# [2.2.1]Heterobicyclic Bromovinyl Sulfones for Thiol-Triggered Strategies in Linker Chemistry: Aza- vs Oxa-Norbornadienic Systems

**DOI:** 10.1021/acs.bioconjchem.5c00371

**Published:** 2025-08-22

**Authors:** Marina Carranza, Ana T. Carmona, Celia Maya, Enrique Gil de Montes, Aldrin V. Vasco, Gonçalo J. L. Bernardes, Antonio J. Moreno-Vargas

**Affiliations:** † Departamento de Química Orgánica, Facultad de Química, 16778 Universidad de Sevilla, C/Prof. García González, 1, 41012 Sevilla, Spain; ‡ Instituto de Investigaciones Químicas (IIQ), Departamento de Química Inorgánica and Centro de Innovación en Química Avanzada (ORFEO–CINQA), Consejo Superior de Investigaciones, Científicas (CSIC) and Universidad de Sevilla, 41092 Sevilla, Spain; § Yusuf Hamied Department of Chemistry, 2152 University of Cambridge, Lensfield Road, CB2 1EW Cambridge, U.K.; ∥ Translational Chemical Biology Group, Spanish National Cancer Research Centre (CNIO), C/Melchor Fernández Almagro, 3. 28029 Madrid, Spain

## Abstract

Azanorbornadienes (ANDs) containing a bromovinyl sulfone are able to accept a first thiol and, in a further stage, fragment upon reaction with a second thiol. This fragmentation has been studied in a collection of differently substituted ANDs. The substitution pattern of the AND influences the rate of the first thiolation and, specially, the further fragmentation. *N*-pyramidalization of selected ANDs was demonstrated via X-ray diffraction. This structural feature attenuates the resonance effect of N-substituents in the further reactivity of ANDs. A comparison with related oxanorbornadienes is also reported. The installation of a fluorogenic AND onto a single domain Antibody against PD-L1 (PD-L1 sdAb) resulted in a conjugate capable of releasing the corresponding fluorogenic pyrrole in the presence of glutathione (GSH) under physiological conditions. Overall, these scaffolds demonstrate potential to be implemented as new drug delivery systems.

## Graphical abstract

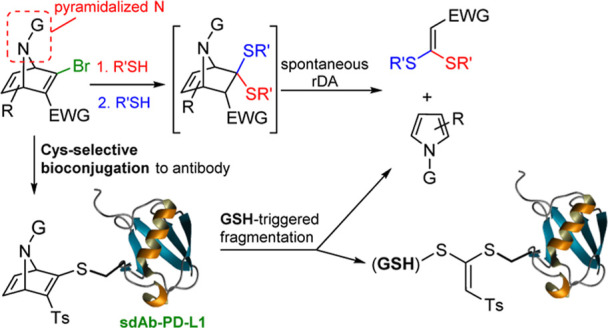

## 1 Introduction

Conjugation of a drug to a targeting vector moiety (e.g., antibody or receptor ligand) is a common strategy for enhancing the selectivity of a cytotoxic compound, ensuring efficacy and avoiding off-target effects.^1^ The resulting conjugate would enrich the desired site due to the action of the vector and release/activate the drug there. This latest aspect highlights the importance of linker chemistry,^2^ as the linker moiety between the vector and the drug should avoid premature drug release and response specifically to a stimulus at the desired site. Several triggering mechanisms can operate for this response. One of the most exploited mechanisms is the thiol-triggered fragmentation of disulfide moieties that has been broadly used in the development of antibody-drug-conjugates (ADCs).^3^ This mechanism takes advantage of the high concentration of tripeptide glutathione (GSH) inside cells (1–10 mM, primarily in the cytoplasm) with respect to the extracellular environment (2–20 μM),^4^ moreover the intracellular [GSH] is even higher in tumor cells.^5^ GSH promotes the reduction of the disulfide moiety of the linker via thiol–disulfide exchange. Although disulfide-based linkers constitute one of the primary linkers employed for drug conjugation,^3^ they are not exempt from problems related with stability of the disulfide-based conjugates in serum: it is problematic to reach a good balance between the stability of the conjugate and the ability to release the payload.^6,7^ Given that GSH has proven to be an effective intracellular trigger for linker fragmentation, the design of novel chemical systems capable of efficiently fragmenting in response to this thiol—through mechanisms other than disulfide bond reduction—remains a significant challenge. The reactivity of electrophilic oxa- and azanorbornadiene (OND and AND) systems toward thiols makes them excellent candidates to be used as thiol-sensitive linkers in targeted drug delivery strategies. (Hetero)­norbornadienes able to spontaneously fragment by accepting one thiol molecule were previously described by Finn and co-workers.^8^ These systems upon fragmentation were able to deliver a pyrrole/furan moiety anchored to a biologically relevant molecule (cargo).^9^ Our group recently reported that AND vinyl and bromovinyl sulfones **1** (Scheme 1) react selectively with the side chain of cysteine **2** via thio-Michael addition (for R = H) or concerted nucleophilic vinylic substitution (S_N_V_σ_, for R = Br) in the presence of other nucleophilic amino acids, being excellent tools for site-selective modification of proteins.^10,11^

**Scheme 1:**
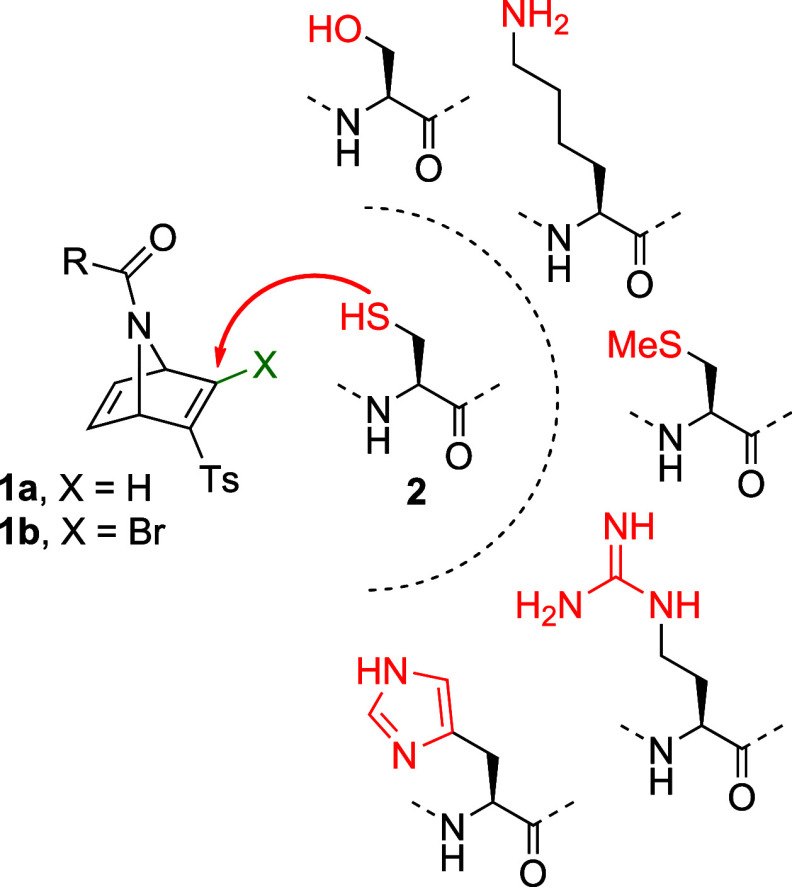
Preferential Reactivity of Azanorbornadienic (bromo)­vinyl Sulfones towards Thiol-Based Nucleophiles

Inspired by these results, we have also explored the thiol-triggered fragmentation of 2-halo-3-tosyl-oxanorbornadienes (Scheme 2, Z=O) as a means to design new thiol-responsive chemical systems.^12^ We have shown that these compounds can sequentially react with two thiol molecules in distinct stages. The first step involves a nucleophilic vinylic substitution, while the second implies a thiol-triggered two-step fragmentation, initiated by a conjugate addition followed by a retro-Diels–Alder (rDA) reaction. In this study, we explore the impact of incorporating a nitrogen atom into the bridge of ANDs on their thiol-promoted fragmentation, enhancing their utility in biomedical applications. The presence of this nitrogen provides an additional site for the attachment of biologically relevant moieties, such as drugs, fluorophores, or affinity probes. We hypothesized that the introduction of an N-substituent could influence both the rate of the initial nucleophilic substitution and the subsequent fragmentation steps, compared to their oxa-analogues. To address this, we first performed a comparative analysis of the thiol-triggered substitution and fragmentation sequences in a series of differently substituted ANDs (and related analogues), using N-acetylcysteamine as a model thiol. Finally, we achieved the Cys-selective labeling of a nanobody with a fluorogenic AND, and evaluated the stability of the resulting bioconjugate in plasma, as well as its capacity to undergo GSH-mediated fragmentation.

**Scheme 2:**
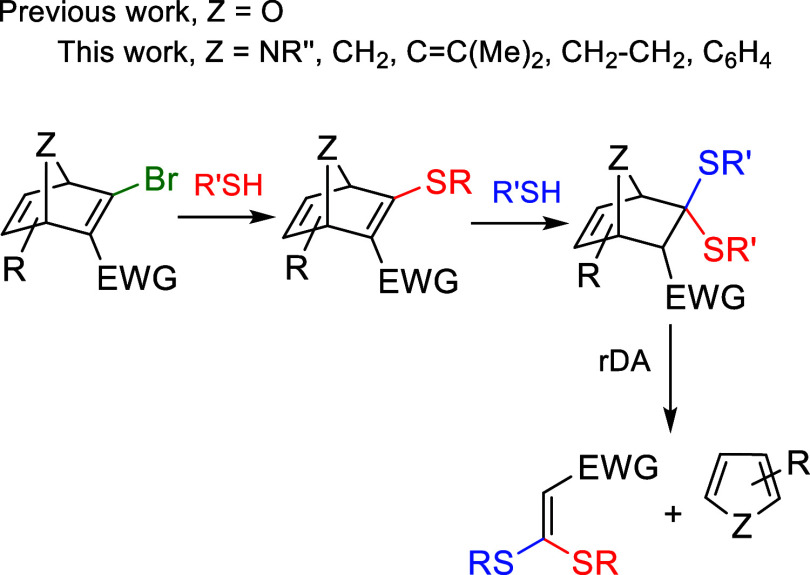
Reaction of (hetero)­norbornadienes with Two Thiols at Different Stages

## 2 Results and Discussion

### 2.1 Synthesis and Structural Studies (Pyramidalization) of 2-bromo­(aza)­norbornadienic Systems

To investigate how structural modifications influence the reactivity of (aza)­norbornadienes toward thiols, we prepared a series of AND derivatives incorporating different N-bridge substituents, electron-withdrawing groups at C-3 and substitution patterns on the bicyclic scaffold. Thus, we carried out the synthesis of 2-bromo-(aza)­norbornadienes **4a**-**18a** and (carba)­analogues **19a**-**22a** (Scheme 3).^13^

**Scheme 3:**
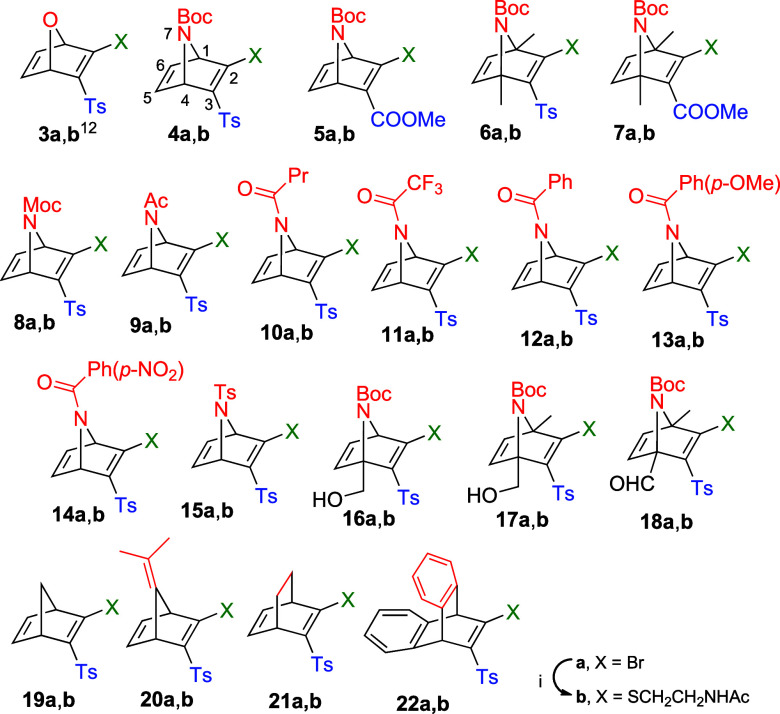
(Hetero)­norbornadienic and Related Systems for This Study^a^

a Reaction conditions: (i) *N*-acetylcysteamine, DMF-Phosphate buffer pH 8.0.

These compounds were synthesized via Diels–Alder (DA) reactions between the appropriate cyclic dienes and electron-deficient alkynes (see Supporting Information for details). DA reactions involving pyrroles required higher temperatures than those with furans, consistent with the lower dienic character of pyrroles due to their significant aromatic stabilization. Introduction of electron-withdrawing groups on the nitrogen atom (e.g., via amidation, alkoxycarbonylation, or sulfonylation) is a well-established strategy to reduce aromaticity by delocalizing the nitrogen lone pair, thereby enhancing their reactivity in DA cycloadditions.^14^ However, this electronic modulation must be carefully balanced, as overly electron-deficient pyrroles become inefficient dienes in normal electron-demand DA reactions. The Boc group offers an optimal balance of electron-withdrawing character, and most N-substituted ANDs were obtained from AND **4a** through a deprotection/acylation sequence (see Supporting Information).

Amides of bicyclic 7-azanorbornanes are known to exhibit intrinsic nitrogen pyramidalization,^15^ which reduces the electron delocalization from the nitrogen lone pair (nN) to the amide π*CO orbital. Motivated by this, we decided to investigate the extent of *N*-pyramidalization in selected N-substituted 7-azanorbornadienes as a potential factor influencing their reactivity toward nucleophiles. Crystals of ANDs **4a**, **6a**, **8a**, **12a**, **15a**, and **23a** (Table 1) were obtained and analyzed by single-crystal X-ray diffraction. The pyramidalization of the bridging nitrogen correlates directly with the angular parameters α and θ (Table 1). Additionally, deviation from planarity at the nitrogen center can result from torsion around the N-substituent bond, quantified by the twist angle τ in amides or carbamates, which corresponds to the rotation about the N–(C=O) bond.^16^ For an ideal sp^2^-hybridized nitrogen, the angles α, θ, and τ would be 180°, 360°, and 0°, respectively.

**Table 1:**
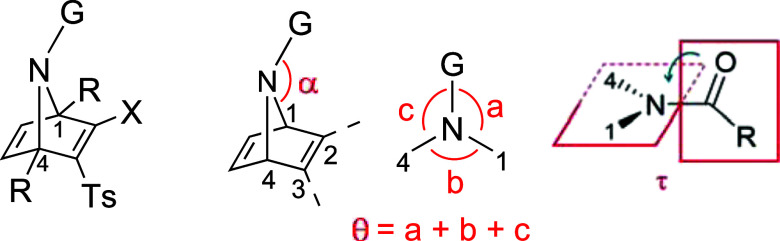
Selected Angular Parameters^a^ from Crystal Structural Data of ANDs **4a**, **6a**, **8a**, **12a**, **15a** and **23a**

AND	G	X	R	α (deg)	θ (deg)	τ (deg)
4a^b^	Boc	Br	H	136.7	333.9	12
				134.8	331.7	4
				136.4	333.5	6.5
6a	Boc	Br	Me	145.2	342.9	1.3
8a	Moc	Br	H	135.2	332.2	1.7
12a	Bz	Br	H	141.3	338.4	15.1
15a^b^	Ts	Br	H	142.1	339.4	
				142.0	339.4	
23a^c^	Boc	H	H	136.5	333.8	9.2

a Angular parameters related with pyramidalization (α,θ) and twist (τ).

b Two/three molecules are involved in the unit cell.

c Compound previously synthesized by us, see ref 10.

The crystallographic data for carbamates **4a**, **6a**, **8a**, **23a**, amide **12a**, and sulfonamide **15a** reveal significant pyramidalization of the nitrogen atom in these compounds. In acyclic systems, the nitrogen atom in *N*-sulfonylamides typically exhibit greater pyramidalization than the corresponding in *N*-acyl amides.^17–19^ This behavior is also observed in N-substituted pyrrolidines and pyrrolines. For instance, *N*-(*p*-nitrobenzoyl)-3-pyrroline and *N*-benzoylpyrrolidine are essentially planar, whereas *N*-tosylpyrrolidine and *N*-tosyl-3-pyrroline show remarkable pyramidalization.^17,18^ In contrast, our azanorbornadienic compounds display pronounced nitrogen pyramidalization regardless of the functional group (carbamate, amide, or sulfonamide), with carbamates **4a**, **8a**, and **23a** exhibiting slightly higher pyramidalization than amide **12a** and sulfonamide **15a**. Substitution with methyl groups at C1 and C4 of the bicyclic scaffold reduces pyramidalization (**4a** vs. **6a**), whereas bromine substitution does not significantly affect the pyramidalization degree (**4a** vs. **23a**). Due to N-pyramidalization, a *syn*-tilt of the *N*-substituent relative to the C2 and C3 substituents of the azanorbornadienic core is observed in all cases (Figure S1). Moreover, a notable twist around the N–C­(=O) bond is evident in **4a**, **23a** and, particularly, in amide **12a**.

### 2.2 Reactivity of Azanorbornadienic Bromovinyl Sulfones toward Thiol Nucleophiles

The nucleophilic vinylic substitution reaction of a set of bromovinyl bicyclic systems toward thiol nucleophiles was explored. The reaction of **4a**-**22a** with *N*-acetylcysteamine under buffered (pH 8.0) aqueous/DMF conditions originated the expected [2.2.1/2]­bicyclic thiovinyl derivatives (**4b**-**22b**) in a fast (less than 30 min, Scheme 3) and clean reaction (no byproducts were identified). Then, competition reactions by pairing selected (hetero)­norbornadienic systems against *N*-acetylcysteamine in DMF-phosphate buffer were accomplished (Table 2). The previously studied oxanorbornadiene (OND) **3a** ^12^ was chosen as reference for comparative studies in some of the competition experiments.

**Table 2:**
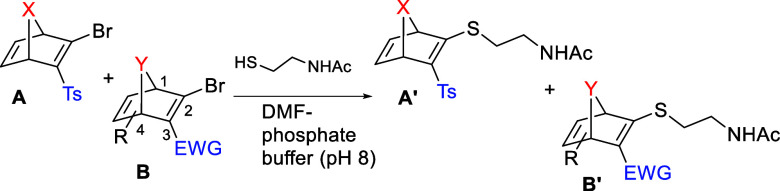
Competition Experiments: Reaction of a Mixture of Two Bromo-(Hetero)­norbornadienes (Br-HNDs) with *N*-Acetylcysteamine^a^

entry	Br-HNDs (A/B)	structural variation	% Conversion into A′/B′
1	**3a**:**4a**	X/Y: O/NBoc	61:39
2	**3a**:**9a**	X/Y: O/NAc	37:63
3	**4a**:**8a**	X/Y: NBoc/NMoc	40:60
4	**4a**:**10a**	X/Y: NBoc/NC(=O)Pr	28:72
5	**4a**:**11a**	X/Y: NBoc/NC(=O)CF_3_	0:100
6	**4a**:**12a**	X/Y: NBoc/NBz	27:73
7	**13a**:**14a**	X/Y: NC(=O)PMP/PNP	36:64
8	**13a**:**15a**	X/Y: NC(=O)PMP/NTs	0:100
9	**3a**:**19a**	X/Y: O/CH_2_	66:34
10	**3a**:**20a**	X/Y: O/C(=CMe_2_)	82:18
11	**3a**:**21a**	X/Y: O/CH_2_CH_2_	73:27
12	**3a**:**22a**	X/Y: O/-CH=CH-	92:8
13	**6a**:**7a**	EWG: Ts/COOMe	35:65

a Reaction conditions: To an equimolar solution of (hetero)­norbornadienes **A**/**B** (50 mM each, 1.0 equiv. each) and *N*-acetylcysteamine (0.9 equiv) in DMF, a similar volume of phosphate buffer solution (pH 8.0, 50 mM) was added. The reaction mixture was stirred for 30 min at room temperature. After workup, the ratio **A**′:**B**′ was analyzed by ^1^H NMR of the unreacted starting material. PMP: *p*-methoxyphenyl; PNP: *p*-nitrophenyl.

The electronegativity of the atom or group in the bridge was found to significantly influence the electrophilic character of these (hetero)­norbornadienes. Specifically, OND **3a** exhibited higher reactivity compared to its *N*-Boc azanorbornadiene analogue **4a**, as well as norbornadienes **19a** and **20a** (entries 1, 9, 10). Among the N-substituted derivatives, *N*-acyl compounds demonstrated greater reactivity than *N*-Boc carbamates (entries 4–6), with the trifluoroacetamide derivative **11a** showing a remarkable reactivity (entry 5). The reduced reactivity of **4a** may be attributed to steric hindrance from the bulky Boc group, as evidenced by increased reactivity upon substitution with the less hindered Moc group (**4a** vs. **8a**, entry 3). This effect could be rationalized by the *syn*-tilting of the *N*-substituent relative to the C2 and C3 substituents of the bicyclic framework. Within the series **13a**–**15a**, which share similar steric profiles, thiol reactivity follows the trend **15a** > **14a** > **13a** (entries 7 and 8). As we have demonstrated, our ANDs exhibit significant *N*-pyramidalization regardless of the bridge substituent (carbamate, acyl, or sulfonamide), which increases the predominance of inductive effects over resonance. The sulfonamide substituent in **15a**, being more electronegative than the amide groups in **14a** and **13a**, contributes to the observed reactivity. On the other hand, the presence of a methoxycarbonyl group instead of a tosyl as electron-withdrawing (EWG) group (**6a** vs **7a**, entry 13) accelerated the substitution reaction. Finally, we also observed that the extension of the bridge (from [2.2.1] to [2.2.2]­bicyclic skeleton) decreased the reactivity of the corresponding compounds (entries 11 and 12).

At this stage, we explored the thiol-promoted fragmentation of selected thio-substituted AND systems (Scheme 4). Compounds **4b**-**19b** were reacted with *N*-acetylcysteamine in CD_3_OD in the presence of Et_3_N. For comparative purposes, data from previously studied fragmentations of selected ONDs (**3b**, **24b**–**26b**) were also included.^12^ The reaction time (t) required to achieve 50% conversion of the heteronorbornadienes into the expected heterocycle (furan or pyrrole) and ketene *S*,*S*-acetal products (**27** or **28**) was monitored. This parameter (t) was determined by ^1^H NMR spectroscopy through plotting the % conversion of the (hetero)­norbornadiene versus reaction time. The direct comparison between oxabicyclic and azabicyclic systems (**3b** vs **4b**, **24b** vs **5b**, **25b** vs **6b**, **26b** vs **7b**) shows that the fragmentation of the aza-analogues is in general much faster than for the oxa-derivatives. Unlike ONDs, where the thioketal intermediate was quantitatively and immediately formed (detected by ^1^H NMR), and the rDA was the rate-limiting step,^12^ in the case of ANDs we have found three different situations. In the first one (Scheme 4, ANDs in blue), compounds **4b**, **6b**, **7b** and **17b** were transformed into the corresponding pyrroles without detection of the thioketal intermediate by ^1^H NMR. This fact indicates that the rate-limiting step was the thio-Michael addition, in accordance with the second-order reaction determined for these fragmentations (Figures S15, S17, S18 and S27). The second situation encompasses ANDs **11b** and **15b**, where the rDA of the resulting thioketal intermediate was the rate-limiting step and a first-order reaction kinetic was determined in both cases (Figures S21 and S26). A third situation was observed for several AND derivatives (**5b**, **9b**, **10b**, **12b**–**14b**, and **16b**; ANDs in red, Scheme 4), where a mixture of starting material, thioketal intermediate and final pyrrole derivative are detected by ^1^H NMR during the fragmentation process. This indicates that the rates of conjugate addition (*k*_1_) and retro-Diels–Alder (*k*_2_) reactions are comparable. Compound **18b** (green) showed exceptionally fast fragmentation, precluding its classification into any of the previous situations.

**Scheme 4:**
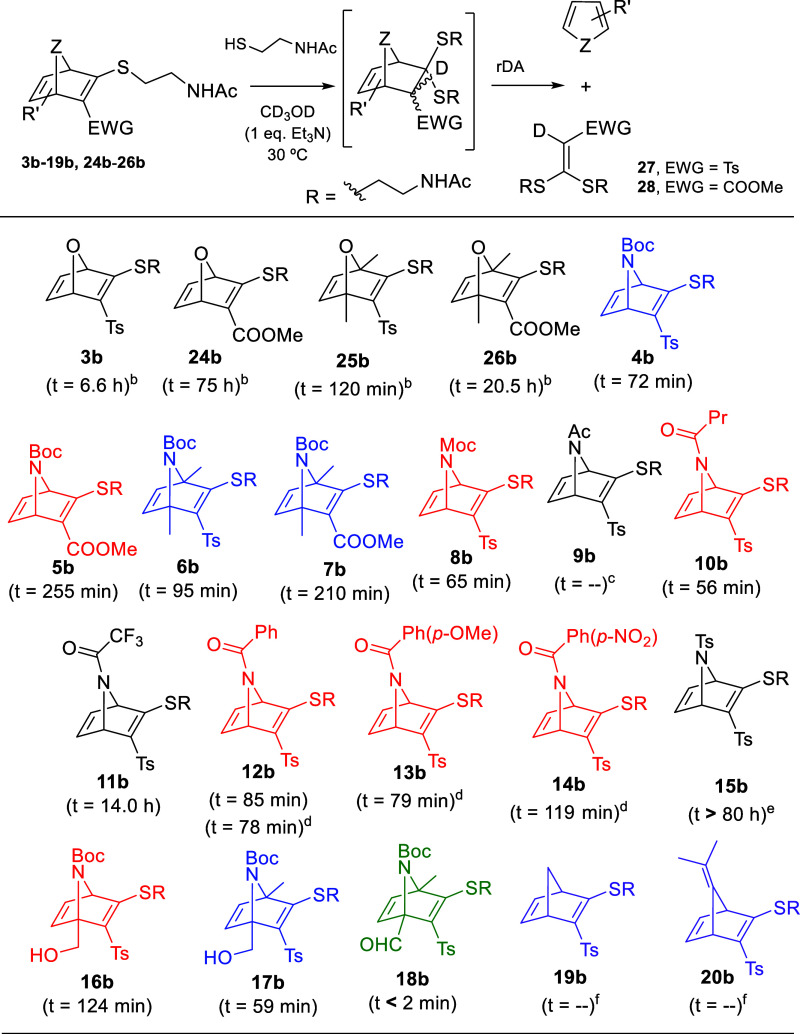
Thiol-Promoted Fragmentation of ANDs and Time (t) Required for the Conversion of (hetero)­norbornadienes Into 50% of the Expected Heterocycle (Furan or Pyrrole)^a^

a Reaction conditions: To a solution of thio-substituted (hetero)­norbornadiene in CD_3_OD (70 mM), 1.1 equiv of *N*-acetylcysteamine in CD_3_OD and Et_3_N (1.1 equiv) were added. The mixture was heated at 30 °C and ^1^H NMR spectra were registered at different intervals. % Conversion of heteronorbornadienes into the expected heterocycle (furan or pyrrole) and ketene *S*,*S*-acetal vs time was plotted. ^b^Data from ref 12. ^c^The resulting pyrrole was not stable under reaction conditions. ^d^This experiment was accomplished in DMSO-*d*_6_ (**13b** and **14b** were not soluble in CD_3_OD). ^e^Thio-Michael addition (3.8 h for completion) is slow although much faster than rDA. ^f^Starting material mostly remains after 24 h. In blue (2nd order kinetics): only starting material + final product is detected; in black (1st order kinetics): only intermediate + final product is detected; in red: intermediate + starting material + final product is detected in the experiment; in green: the transformation was so fast that the identification of the species in solution was not possible during the experiment; in lilac: fragmentation products were not observed.

The reactivity of compounds **4b**, **6b**, **7b**, and **17b**, all of them in situation 1 (thio-Michael addition as rate-limiting step), can be attributed to two main factors: (1) steric hindrance from the N-Boc group, whose *syn*-tilt relative to C2/C3 may interfere with nucleophilic attack; and (2) the lower electronegativity of the carbamate moiety compared to the bridge oxygen in ONDs, which reduces the electrophilicity of the azabicyclic system in the thio-Michael addition. AND **11b** behaved similarly to ONDs, with rapid and quantitative formation of the thioketal intermediate as confirmed by ^1^H NMR, followed by a slow rDA fragmentation (*t* = 13.5 h). In the case of **15b** (*t* > 80 h) both, the thio-Michael addition and the rDA were slow, with the rDA being markedly slower than the initial addition.^20^ Both compounds, **11b** and **15b** present a notably slow fragmentation kinetic. In both cases, the rate-limiting step corresponds to the retro-Diels–Alder (rDA) reaction, reflecting the low dienic character of the resulting pyrrole which would difficult both, the direct and the retro Diels–Alder reaction. Concerning amides **10b** and **12b**-**14b** no significant differences in *t* (78–119 min) were observed, despite the different electron-withdrawing character of the acyl group of the nitrogen. Although the type of substitution of the N atom should influence both the conjugate addition and the rDA reaction, the notable pyramidalization of the N in the azanorbornadienic skeleton avoids, in part, the delocalization of the lone electron pair on the N by resonance effect, and therefore, reduces the expected electronic effect of the amide substituents.

The introduction of substituents at C1 and/or C4 of ANDs did not significantly affect the fragmentation rate (compounds **6b**, **16b**, and **17b** vs **4b**). In contrast, the incorporation of a formyl group at the bridgehead carbon (compound **18b**) led to a dramatic increase in the fragmentation rate (*t* < 2 min). This could be due to the electronic repulsion between nearby CHO and Ts groups in the thioketal intermediate, resulting in destabilization that would be the driving force for the extremely fast rDA reaction. The influence of the electron-withdrawing group at the thiovinyl moiety (C3) was also examined. Replacing the tosyl group with an ester proved to be detrimental to the fragmentation process (**5b** vs **4b**, **7b** vs **6b**), likely due to the superior thio-Michael acceptor ability of vinyl sulfones over acrylates.^21^ In the case of carba-bicyclic systems **19b** and **20b**, no thio-Michael addition was observed even after 24 h.

#### 2.2.1 Azabicyclic Bromovinyl Sulfones for Protein Labeling. GSH-Triggered Degradation of the Bioconjugate

To evaluate the feasibility of our ANDs as cleavable linkers for drug delivery system design, we conjugated dansylated AND **29** to a PD-L1 single-domain antibody (sdAb), following the same experimental protocol previously optimized for the bioconjugation of ubiquitin with a related bromo-AND.^11^ We monitored the fragmentation of the resulting bioconjugate **A** in the presence of GSH at various time points (Scheme 5). The fluorophore in **29** was incorporated through a glycine spacer as *N*-acyl ANDs showed higher fragmentation rates than their *N*-sulfonyl analogues (see Scheme 4, compounds **15b** and **10b**).

**Scheme 5:**
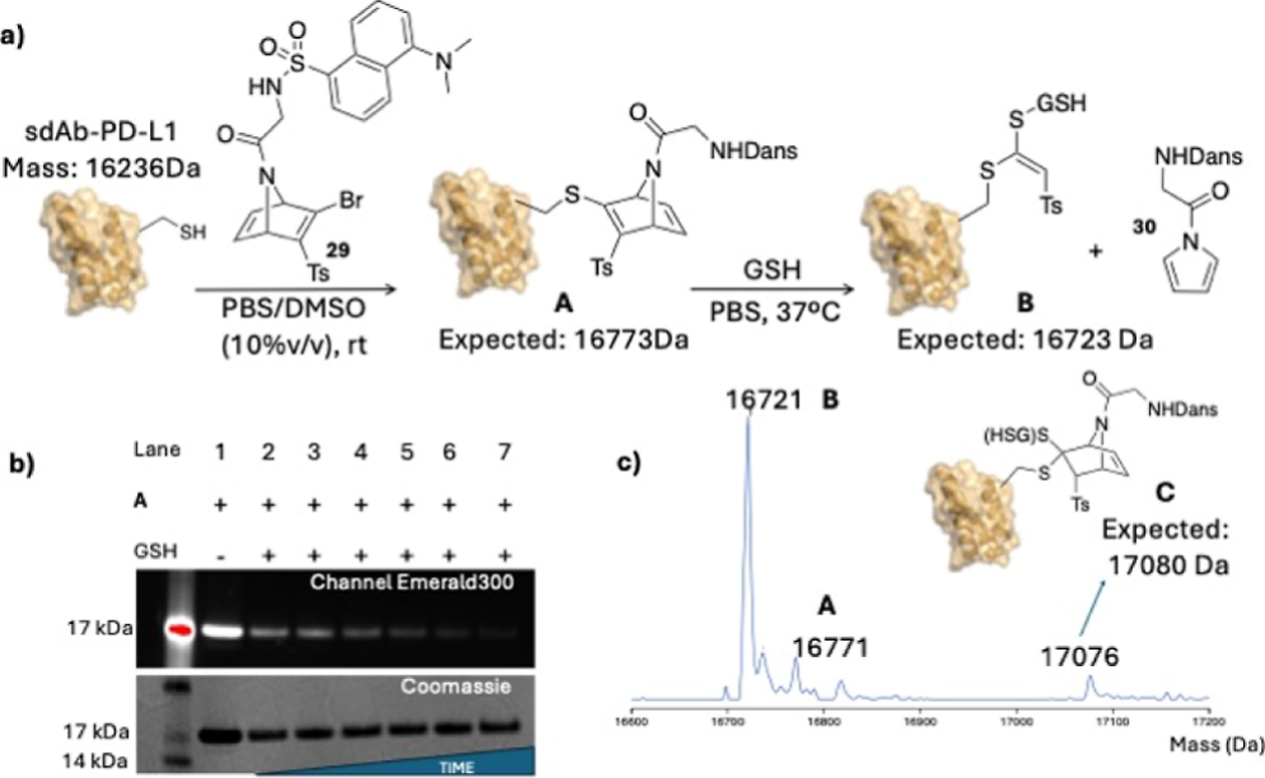
(a) GSH-Promoted Fragmentation Reaction between Bioconjugate **A** and GSH; (b) Bioconjugate **A** Incubated with GSH at different Times on a NuPAGE SDS Gel. Lane 1 Bioconjugate **A**; Lanes 2 to 7: **A** + GSH after 5, 15, 30, 45, 60, 120, and 240 min Incubation at 37 °C; (c) MS Trace of the Reaction Crude between GSH and Bioconjugate **A** after 240 min at 37 °C

Bioconjugate **A** presented attenuated fluorescence compared to that shown by dansyl pyrrole **30**, presumably due to photoinduced electron transfer (PET) effect between the conjugated double bond and the fluorophore. This effect was also observed for a model of bioconjugate **A** (Supporting Information, Figure S29). Since this PET is not completely quenching dansyl fluorescence, residual emission can still be observed in bioconjugate **A** (Scheme 5b, lane 1) and, therefore, can be monitored over time as an indicative of pyrrole release when the bioconjugate is incubated in GSH solution (Scheme 5b, lanes 2 to 7). As shown in Scheme 5b, dansyl residual fluorescence quickly decreases over time and after 240 min, emission was virtually undetectable- consistent with the conversion of **A** into **B**. LCMS studies carried out on the reaction mixture after 4 h incubation (Scheme 5c) confirmed the formation of product **B** as the main species identified (expected 16,723 Da, found 16,721 Da). Interestingly, intermediate **C** (GSH-adduct) was also detected by MS (expected 17,080, found 17,076). However, its concentration in the sample must be very low as almost no fluorescence was observed at that time.

Finally, we decided to investigate the stability of bioconjugate **A** in the presence of other biologically relevant thiols, selecting albumin as a representative example. Albumin is the most abundant circulating protein in blood and its potential interaction with AND-derived conjugate may result in an undesirable cargo release thereby, limiting the utility of AND scaffolds as new cleavable linkers. For that reason, bioconjugate **A** was incubated in both Human Serum Albumin (HAS) and human plasma at 37 °C for 4 days and the reactivity of the conjugate was assessed as it was previously described for GSH (Supporting Information, Figure S34). To our delight, no fluorescence decrease was observed over the curse of the incubation, which may indicate that cysteine 34 of the albumin is unable to attack the thiovinyl sulfone function on **A** -most likely due to steric hindrance- thus confirming not only the stability of the bioconjugate in plasma, but also the applicability of these ANDs as cleavable linkers.

## 3 Conclusions

ANDs incorporating a bromovinyl sulfone moiety exhibit a unique sequential reactivity, undergoing initial thiol–Michael addition followed by a second thiol-triggered fragmentation of the bicyclic scaffold. This dual-thiol responsiveness makes them promising candidates as cleavable linkers for targeted drug delivery, where the first thiol could mimic a targeting vector and the second be supplied by an intracellular trigger such GSH. Unlike previously reported ANDs and ONDs, which accept only one thiol, these systems support a two-step, stimulus-responsive fragmentation. Structural effects play a key role in modulating this reactivity. The pyramidalization of the N-bridge —confirmed in selected cases— partially attenuates the resonance contribution of N-substituents, resulting in generally consistent fragmentation rates across most ANDs studied (*t* = 56–255 min). However, strong electron-withdrawing substituents such as sulfonamides can significantly reduce reactivity (**15b**, *t* > 80 h), making this linkage unsuitable for N-decoration with biologically relevant fragments. In contrast, amide and carbamate linkages proved more effective for such purposes. The steric consequences of nitrogen pyramidalization probably contribute to the distinct fragmentation behavior observed in ANDs compared to previously studied ONDs. Specifically, the initial thiol–Michael addition tends to proceed more rapidly in ONDs; nevertheless, the overall fragmentation process is generally faster in AND analogues. Finally, conjugation of AND **29** to a single-domain antibody (sdAb) produced a plasma-stable bioconjugate that underwent efficient GSH-triggered cleavage. Taken together, these results highlight the utility of AND-based scaffolds as tunable, cleavable linkers with potential applications in drug delivery and targeted bioconjugation.

## 4 Experimental Procedures

### 4.1 General Methods

^1^H- and ^13^C NMR spectra were recorded with a Bruker AVIII300, NEO300, NEO400 and NEO500 spectrometer for solutions in CDCl_3_, CD_3_OD, (CD_3_)_2_CO, (CD_3_)_2_SO, and C_6_D_6_. δ are given in ppm and *J* in Hz. Chemical shifts are calibrated using residual solvent signals. All the assignments were confirmed by 2D spectra (COSY and HSCQ). High resolution mass spectra were recorded on a Q-Exactive-quadrupole mass spectrometer. TLC was performed on silica gel 60 F_254_ (Merck), with detection by UV light charring with KMnO_4_, ninhydrin, or with reagent [(NH_4_)_6_MoO_4_, Ce­(SO_4_)_2_, H_2_SO_4_, H_2_O]. Purification by silica gel chromatography was carried out using either hand-packed glass columns (Silica gel 60 Merck, 40–60 and 63–200 μm) or Puriflash XS520 Plus Interchim system with prepacked cartridges.

### 4.2 General Procedure for the Preparation of Bromo-ANDs **3a**–**8a**, **15a**–**17a**

To a solution of activated alkyne **S1** or **S2** (x mmol) in toluene (2 mL/mmol), the corresponding commercial or synthetic pyrrole derivative **S3**–**S8** (z mmol) was added, and the reaction mixture was stirred at 50–90 °C. After the reaction was completed, the solvent was evaporated, and the resulting residue was purified by a chromatography column on silica gel to give the corresponding bromo-AND.

#### 4.2.1 General Procedure for the Synthesis of Thio-(hetero)­Norbornadienes **4b**–**20b**.^22^

A solution of the corresponding bromo-AND (*x* mmol) in DMF or MeCN (10 mL/mmol), a solution of *N*-acetylcysteamine (*z* mmol) in DMF or MeCN (5 mL/mmol) and phosphate buffer solution (pH 8.0, 50 mM, 10 mL/mmol), were added simultaneously and the mixture was stirred at r.t. for 30 min. Then, solvents were evaporated, and the residue was dissolved in EtOAc and washed with water. The organic phase was dried with Na_2_SO_4_ anh., filtered and concentrated. Purification by silica gel column chromatography afforded the corresponding thio-AND.

#### 4.2.2 (rac) *tert*-Butyl-2-((2-acetamidoethyl)­thio)-3-tosyl-7-azabicyclo­[2.2.1]­hepta-2,5-diene-7-carboxylate (**4b**)

Starting from **4a** (412 mg, 0.95 mmol) and *N*-acetylcysteamine (95 mg, 0.80 mmol) in MeCN following the general procedure, afforded after chromatographic purification (EtOAc/CyHex 2:1 → 10:1) compound **4b** (258 mg, 0.55 mmol, 69%) as a brown oil. ^1^H NMR (300 MHz, CDCl_3_, δ ppm): 7.76 (d, 2H, *J* = 7.9 Hz, Ar–H), 7.32 (d, 2H, *J* = 7.7 Hz, Ar–H), 6.82 (br s, 2H, H-5, H-6), 6.36 (br s, 1H, NH), 5.54 (br s, 1H, H-1 or H-4), 5.31 (br s, 1H, H-1 or H-4), 3.55–3.51 (m, 2H, H-2′), 3.19–3.09 (m, 2H, H-1′), 2.42 (s, 3H, CH_3_ of Ts), 1.98 (s, 3H, CH_3_CO), 1.28 (s, 9H, CH_3_ of Boc). ^13^C NMR (75 MHz, CDCl_3_, δ ppm): 170.7, 166.9, 154.2, 144.6, 142.6, 141.1, 138.2, 137.2, 129.9, 127.3, 81.9, 70.8, 69.4, 40.3, 32.2, 27.3, 23.1, 21.6. HRMS (ESI) *m*/*z*; found, 487.1330 calcd for C_22_H_28_N_2_NaO_5_S_2_ [M + Na]^+^: 487.1337.

#### 4.2.3 (rac) 7-(*tert*-Butyl)-2-methyl-3-((2-acetamidoethyl)­thio)-7-azabicyclo­[2.2.1]­hepta-2,5-diene-2,7-dicarboxylate (**5b**)

Starting from **5a** (250 mg, 0.76 mmol) and *N*-acetylcysteamine (75 mg, 0.63 mmol) in DMF following the general procedure, afforded after chromatographic purification (EtOAc/CyHex 1:1 → EtOAc) compound **5b** (159 mg, 0.43 mmol, 68%) as a yellow oil. ^1^H NMR (300 MHz, CD_3_OD, δ ppm): 7.08 (s, 2H, H-5, H-6), 5.74–5.64 (m, 1H, H-1 or H-4), 5.41 (ap q, *J* = 1.6 Hz, H-1 or H-4), 3.72 (s, 3H, COOCH_3_), 3.54–3.29 (m, 2H, H-2′), 3.19–3.09 (m, 2H, H-1′), 1.96 (s, 3H, COCH_3_), 1.40 (s, 9H, CH_3_ of Boc). ^13^C NMR (75 MHz, CD_3_OD, δ ppm): 172.3, 164.3, 142.9, 139.9, 81.2, 69.7, 68.3, 67.6, 60.2, 50.8, 40.4, 30.7, 27.0, 21.1. HRMS (ESI) *m*/*z*; found, 391.1289 calcd for C_17_H_24_N_2_NaO_5_S [M + Na]^+^: 391.1281.

#### 4.2.4 (rac) *tert*-Butyl-2-((2-acetamidoethyl)­thio)-1,4-dimethyl-3-tosyl-7-azabicyclo­[2.2.1]­hepta-2,5-diene-7-carboxylate (**6b**)

Starting from **6a** (206 mg, 0.85 mmol) and *N*-acetylcysteamine (84 mg, 0.71 mmol) in MeCN following the general procedure, afforded after chromatographic purification (EtOAc/CyHex 10:1) compound **6b** (152 mg, 0.31 mmol, 44%) as a brown oil. ^1^H NMR (300 MHz, CD_3_OD, δ ppm): 7.76 (d, 2H, *J* = 8.4 Hz, Ar–H), 7.42 (d, 2H, *J* = 8.0 Hz, Ar–H), 6.62 (d, 1H, *J* = 5.4 Hz, H-5 or H-6), 6.55 (d, 1H, *J* = 5.2 Hz, H-5 or H-6), 3.26–3.19 (m, 2H, H-2′), 3.14–3.11 (m, 2H, H-1′), 2.44 (s, 3H, CH_3_ of Ts), 1.98 (s, 3H, CH_3_CO), 1.94 (s, 3H, CH_3_), 1.92 (s, 3H, CH_3_), 1.31 (s, 9H, CH_3_ of Boc). ^13^C NMR (75 MHz, CD_3_OD, δ ppm): 173.3 (C=O), 170.9 (C=O), 156.4, 153.6 (C-2, C-3), 148.5 (C-5 or C-6), 146.3 (C_q_Ar), 146.2 (C-5 or C-6), 139.3 (C_q_Ar), 131.0, 128.7 (CH-Ar), 83.0, 82.8, 79.7 (C-1, C-4, C_q_ of Boc), 40.8 (C-2′), 34.4 (C-1′), 28.4 (CH_3_ of Boc), 22.3 (CH_3_CO), 21.6 (CH_3_ of Ts), 18.5 (CH_3_), 18.0 (CH_3_). HRMS (ESI) *m*/*z*; found, 515.1642 calcd for C_24_H_32_N_2_NaO_5_S_2_ [M + Na]^+^: 515.1650.

#### 4.2.5 (rac) 7-(*tert*-Butyl)-2-methyl-3-((2-acetamidoethyl)­thio)-1,4-dimethyl-7-azabicyclo­[2.2.1]­hepta-2,5-diene-2,7-dicarboxylate (**7b**)

Starting from **7a** (190 mg, 0.53 mmol) and *N*-acetylcysteamine (58 mg, 0.48 mmol) in DMF following the general procedure, afforded after chromatographic purification (EtOAc/CyHex 10:1 → EtOAc) compound **7b** (85 mg, 0.21 mmol, 44%) as a dark brown oil. ^1^H NMR (300 MHz, CD_3_OD, δ ppm): 6.72 (d, 1H, *J* = 5.8 Hz, H-5 or H-6), 6.67 (d, 1H, *J* = 5.3 Hz, H-5 or H-6), 3.78 (s, 3H, COOCH_3_), 3.25–3.19 (m, 2H, H-2′), 3.09–3.01 (m, 2H, H-1′), 1.94 (s, 3H, CH_3_CO), 1.93 (s, 3H, CH_3_), 1.89 (s, 3H, CH_3_), 1.42 (s, 9H, CH_3_ of Boc). ^13^C NMR (75 MHz, CD_3_OD, δ ppm): 171.9 (C=O), 165.9 (C=O), 165.7 (C=O), 155.2 (C-2 or C-3), 147.4, 145.2 (C-5, C-6), 141.3 (C-2 or C-3), 80.9 (C_q_ of Boc), 79.7, 77.6 (C-1, C-4), 50.6 (COOCH_3_), 38.4 (C-2′), 31.8 (C-1′), 27.2 (CH_3_ of Boc), 21.1 (CH_3_CO), 16.8 (CH_3_), 15.4 (CH_3_). HRMS (ESI) *m*/*z*; found, 419.1610 calcd for C_19_H_28_N_2_NaO_5_S [M + Na]^+^: 419.1617.

#### 4.2.6 (rac) Methyl-2-((2-acetamidoethyl)­thio)-3-Tosyl-7-azabicyclo[2.2.1]­hepta-2,5-diene-7-carboxylate (**8b**)

Starting from **8a** (61 mg, 0.16 mmol) and *N*-acetylcysteamine (17 mg, 0.15 mmol) in MeCN following the general procedure, afforded after chromatographic purification (EtOAc) compound **8b** (40 mg, 0.094 mmol, 63%) as a brown solid. ^1^H NMR (300 MHz, DMSO-*d*_6_, 353 K, δ ppm): 7.88 (br s, 1H, NH), 7.69 (d, 2H, *J* = 8.4 Hz, Ar–H), 8.16 (d, 2H, *J* = 8.1 Hz, Ar–H), 7.00 (ddd, 1H, *J* = 5.3, 2.8, 0.7 Hz, H-5 or H-6), 6.93 (ddd, 1H, *J* = 5.6, 2.3, 0.5 Hz, H-5 or H-6), 5.72 (t, 1H, *J* = 2.2 Hz, H-1 or H-4), 5.30 (t, 1H, *J* = 2.2 Hz, H-1 or H-4), 3.43 (s, 3H, COOCH_3_), 3.40–3.09 (m, 4H, H-1′, H-2′), 2.42 (s, 3H, CH_3_ of Ts), 1.85 (s, 3H, CH_3_CO). ^13^C NMR (75 MHz, DMSO-*d*_6_, 353 K, δ ppm): 169.1 (C=O), 154.0 (C=O), 143.8 (C-2 or C-3), 142.0 (C-5 or C-6), 138.8 (C_q_Ar), 138.4 (C-5 or C-6), 136.8 (C-2 or C-3), 133.5 (C_q_Ar), 129.3 (CH-Ar), 70.2, 68.2 (C-1, C-4), 52.2 (COOCH_3_), 40.2–38.0 (C-1′ or C-2′, under solvent signal), 30.9 (C-1′ or C-2′), 21.9 (CH_3_ of Ts), 20.5 (CH_3_CO). HRMS (ESI) *m*/*z*; found, 445.0868 calcd for C_19_H_22_N_2_NaO_5_S_2_ [M + Na]^+^: 445.0859.

#### 4.2.7 N-(2-(((rac)-7-Acetyl-3-Tosyl-7-azabicyclo­[2.2.1]­hepta-2,5-dien-2-yl)­thio)­ethyl)­acetamide (**9b**)

Starting from **9a** (135 mg, 0.37 mmol) and *N*-acetylcysteamine (37 mg, 0.31 mmol) in MeCN following the general procedure, afforded after chromatographic purification (EtOAc/CyHex 10:1 → EtOAc) compound **9b** (77 mg, 0.19 mmol, 61%) as a brown oil. ^1^H NMR (300 MHz, CD_3_OD, δ ppm, major rotamer): 7.76 (d, 2H, *J* = 8.3 Hz, Ar–H), 7.45–7.39 (m, 2H, Ar–H), 7.05–6.88 (m, 2H, H-5, H-6), 6.01 (t, 1H, *J* = 2.3 Hz, H-1 or H-4), 5.58 (t, 1H, *J* = 2.5 Hz, H-1 or H-4), 3.56–3.35 (m, 2H, H-2′) 3.26–3.11 (m, 2H, H-1′), 2.44 (s, 3H, CH_3_ of Ts), 2.15, (s, 3H, CH_3_CO), 1.96 (s, 3H, CH_3_CO). ^13^C NMR (75 MHz, CD_3_OD, δ ppm, major rotamer): 172.5 (C=O), 169.3 (C=O), 166.6, 145.2 (C-2, C-3), 142.5 (C-5 or C-6), 140.3 (C_q_Ar), 138.1 (C-5 or C-6), 136.8 (C_q_Ar), 129.7 (CH-Ar), 126.9 (CH-Ar), 69.9, 68.1 (C-1, C-4), 40.6 (C-2′), 31.1 (C-1′), 21.11 (CH_3_ of Ts), 21.07 (CH_3_CO), 19.7 (CH_3_CO). HRMS (ESI) *m*/*z*; found, 429.0905 calcd for C_19_H_22_N_2_NaO_4_S_2_ [M + Na]^+^: 429.0919.

#### 4.2.8 N-(2-(((rac)-7-Butyryl-3-Tosyl-7-azabicyclo­[2.2.1]­hepta-2,5-dien-2-yl)­thio)­ethyl)­acetamide (**10b**)

Starting from **10a** (195 mg, 0.49 mmol) and *N*-acetylcysteamine (53 mg, 0.45 mmol) in MeCN following the general procedure, afforded after chromatographic purification (EtOAc → EtOAc/Acetone 10:1) compound **10b** (96 mg, 0.22 mmol, 49%) as a yellow oil. ^1^H NMR (300 MHz, CD_3_OD, δ ppm, major rotamer): 7.78 (d, 2H, *J* = 8.4 Hz, Ar–H), 7.47–7.41 (m, 2H, Ar–H), 7.07–6.93 (m, 2H, H-5, H-6), 6.04 (t, 1H, *J* = 2.3 Hz, H-1 or H-4), 5.63 (t, 1H, *J* = 2.2 Hz, H-1 or H-4), 3.61–3.36 (m, 2H, H-2′), 3.28–3.11 (m, 2H, H-1′), 2.46 (s, 3H, CH_3_ of Ts), 2.26–2.03 (m, 2H, CH_2_–CH_2_–CH_3_), 1.99 (s, 3H, CH_3_CO), 1.59–1.27 (m, 2H, CH_2_–CH_2_–CH_3_), 0.90–0.79 (m, 3H, CH_2_–CH_2_–CH_3_). ^13^C NMR (75 MHz, CD_3_OD, δ ppm, major rotamer): 172.3 (C=O), 169.4 (C=O), 145.2, 143.4 (C-2, C-3), 142.6 (C-5 or C-6), 139.2 (C_q_Ar), 138.2 (C-5 or C-6), 137.0 (C_q_Ar), 129.8 (CH-Ar), 126.9 (CH-Ar), 68.0, 67.8 (C-1, C-4), 40.0 (C-2′), 34.8 (CH_2_–CH_2_–CH_3_) 30.5 (C-1′), 21.1 (CH_3_CO), 20.2 (CH_3_ of Ts), 17.9 (CH_2_–CH_2_–CH_3_), 12.5 (CH_2_–CH_2_–CH_3_). HRMS (ESI) *m*/*z*; found, 457.1221 calcd for C_21_H_26_N_2_NaO_4_S_2_ [M + Na]^+^: 457.1232.

#### 4.2.9 N-(2-(((rac)-3-Tosyl-7-(2,2,2-Trifluoroacetyl)-7-azabicyclo­[2.2.1]­hepta-2,5-dien-2-yl)­thio)­ethyl)­acetamide (**11b**)

Starting from **11a** (75 mg, 0.23 mmol) and *N*-acetylcysteamine (25 mg, 0.21 mmol) in MeCN following the general procedure, afforded after chromatographic purification (EtOAC/CyHex 10:1 → EtOAc) compound **11b** (25 mg, 0.07 mmol, 33%) as a yellow oil. ^1^H NMR (300 MHz, CD_3_OD, δ ppm, mixture of rotamers): δ 7.65 (d, 2H, *J* = 8.5 Hz, Ar–H), 7.32 (d, 2H, *J* = 7.9 Hz, Ar–H), 7.01 (dd, 1H, *J* = 5.2, 2.9 Hz, H-5 or H-6), 6.91 (dd, 1H, *J* = 5.6, 2.4 Hz, H-5 or H-6), 6.14–6.11 (m, 1H, H-1 or H-4), 5.64 (br s, 1H, H-1 or H-4), 3.45–3.22 (m, 4H, H-1′ or H-2′), 2.34 (s, 3H, CH_3_ of Ts), 1.86 (s, 3H, CH_3_CO). ^13^C NMR (75 MHz, CD_3_OD, δ ppm, mixture of rotamers): δ 172.4, 167.6, 165.3, 143.8, 139.0, 138.4, 136.5, 129.8, 127.0, 67.9, 65.8, 40.0, 31.1, 21.0, 20.2. HRMS (ESI) *m*/*z*; found, 483.0631 calcd for C_19_H_19_N_2_F_3_NaO_4_S_2_ [M + Na]^+^: 483.0631.

#### 4.2.10 N-(2-(((rac)-7-Benzoyl-3-Tosyl-7-azabicyclo­[2.2.1]­hepta-2,5-dien-2-yl)­thio)­ethyl)­acetamide (**12b**)

Starting from **12a** (175 mg, 0.41 mmol) and *N*-acetylcysteamine (44 mg, 0.37 mmol) in MeCN following the general procedure, afforded after chromatographic purification (EtOAC/CyHex 10:1 → EtOAc) compound **12b** (116 mg, 0.25 mmol, 68%) as a yellow solid. ^1^H NMR (300 MHz, DMSO-*d*_6_, δ ppm, mixture of rotamers): 7.58–7.26 (m, 9H, Ar–H), 7.18–7.01 (m, 2H, H-5, H-6), 6.07 (br s, 1H, H-1 or H-4), 5.36 (br s, 1H, H-1 or H-4), 3.23–2.87 (m, 4H, H-1′, H-2′), 2.38 (s, 3H, CH_3_ of Ts), 1.90 (s, 3H, CH_3_CO). ^13^C NMR (75 MHz, DMSO-*d*_6_, δ ppm, mixture of rotamers): 170.2, 168.7, 144.8, 143.5, 139.5, 137.1, 132.2, 130.4, 130.2, 129.0, 128.9, 128.3, 127.8, 126.8, 71.0, 69.4, 60.2, 38.7, 31.8, 23.0, 21.6. HRMS (ESI) *m*/*z*; found, 491.1075 calcd for C_24_H_24_N_2_NaO_4_S_2_ [M + Na]^+^: 491.1070.

#### 4.2.11 N-(2-(((rac)-7-(4-Methoxybenzoyl)-3-Tosyl-7-azabicyclo­[2.2.1]­hepta-2,5-dien-2-yl)­thio)­ethyl)­acetamide (**13b**)

Starting from **13a** (187 mg, 0.43 mmol) and *N*-acetylcysteamine (47 mg, 0.39 mmol) in MeCN following the general procedure, afforded after chromatographic purification (EtOAc/CyHex 10:1 → EtOAc) compound **13b** (93 mg, 0.19 mmol, 49%) as a pale yellow solid. ^1^H NMR (300 MHz, DMSO-*d*_6_, δ ppm, mixture of rotamers): 7.58–7.50 (m, 2H, Ar–H), 7.40–7.26 (m, 4H, Ar–H), 7.18–7.10 (m, 2H, Ar–H), 7.00–6.94 (m, 2H, H-5, H-6), 6.06 (br s, 1H, H-1 or H-4), 5.45 (br s, 1H, H-1 or H-4), 3.88 (s, 3H, OCH_3_), 3.58–3.20 (m, 4H, H-1′, H-2′), 2.43 (s, 3H, CH_3_ of Ts), 1.91 (s, 3H, CH_3_CO). ^13^C NMR (75 MHz, DMSO-*d*_6_, δ ppm, mixture of rotamers): 170.2 (C=O), 162.4 (C=O), 144.7, 143.5, (C-2, C-3), 139.3, 137.1, 130.5, 130.4, 126.8 (CH-Ar), 114.2 (C-5, C-6), 71.5, 69.7 (C-1, C-4), 55.9 (OCH_3_), 40.0, 31.8 (C-1′, C-2′), 23.O (CH_3_ of Ts), 21.6 (CH_3_CO). HRMS (ESI) *m*/*z*; found, 521.1166 calcd for C_25_H_26_N_2_NaO_5_S_2_ [M + Na]^+^: 521.1175.

#### 4.2.12 N-(2-(((rac)-7-(4-Nitrobenzoyl)-3-Tosyl-7-azabicyclo­[2.2.1]­hepta-2,5-dien-2-yl)­thio)­ethyl)­acetamide (**14b**)

Starting from **14a** (165 mg, 0.37 mmol) and *N*-acetylcysteamine (40 mg, 0.34 mmol) in MeCN following the general procedure affored **14b** (71 mg, 0.14 mmol, 41%) as a pale-yellow solid after precipitation with AcOEt. ^1^H NMR (300 MHz, DMSO-*d*_6_, δ ppm, mixture of rotamers): 8.27–8.12 (m, 2H, Ar–H), 7.51 (m, 5H, Ar–H), 7.24–7.16 (m, 4H, Ar–H, H-5, H-6), 6.16 (br s, 1H, H-1 or H-4), 5.37 (br s, 1H, H-1 or H-4), 3.49–3.09 (m, 4H, H-1′, H-2′), 2.36 (s, 3H, CH_3_ of Ts), 1.87 (s, 3H, CH_3_CO). ^13^C NMR (75 MHz, DMSO-*d*_6_, δ ppm, mixture of rotamers): 170.2 (C=O), 166.9 (C=O), 149.4, 144.9 (C-5, C-6), 143.4, 138.2, 138.0, 137.6, 137.1, (C_q_Ar), 130.5, 129.7, 126.9, 124.2 (CH-Ar), 70.6, 69.1 (C-1, C-4), 40.0, 31.8 (C-1′, C-2′), 23.0 (CH_3_ of Ts), 21.5 (CH_3_CO). HRMS (ESI) *m*/*z*; found, 536.0913 calcd for C_24_H_23_N_3_NaO_6_S_2_ [M + Na]^+^: 536.0920.

#### 4.2.13 N-(2-(((rac)-3,7-Ditosyl-7-azabicyclo­[2.2.1]­hepta-2,5-dien-2-yl)­thio)­ethyl)­acetamide (**15b**)

Starting from **15a** (80 mg, 0.17 mmol) and *N*-acetylcysteamine (18 mg, 0.15 mmol) in MeCN following the general procedure, afforded after chromatographic purification (EtOAc/CyHex 10:1 →EtOAc) compound **15b** (51 mg, 0.098 mmol, 65%) as a yellow solid. ^1^H NMR (300 MHz, CD_3_OD, δ ppm): 7.69–7.61 (m, 4H, Ar–H), 7.43–7.34 (m, 4H, Ar–H), 6.84 (dd, 1H, *J* = 5.5, 3.2 Hz, H-5 or H-6), 6.70 (dd, 1H, *J* = 5.5, 2.6 Hz, H-5 or H-6), 5.75 (t, 1H, *J* = 2.4 Hz, H-1 or H-4), 5.30 (t, 1H, *J* = 2.5 Hz, H-1 or H-4), 3.28–3.09 (m, 4H, H-1′, H-2′), 2.44 (s, 6H, CH_3_ of Ts), 2.04 (s, 3H, CH_3_CO). ^13^C NMR (75 MHz, CD_3_OD, δ ppm): 165.6 (C=O), 145.0, 144.5 (C-2, C-3), 143.1, 138.7 (C-5, C-6), 137.6, 134.7 (C_q_Ar), 129.7, 129.6 (CH-Ar), 129.4 (C_q_Ar), 128.3 (CH-Ar), 128.2 (C_q_Ar), 127.0 (C_q_Ar), 71.4, 68.5 (C-1, C-4), 40.2, 30.8 (C-1′, C-2′), 21.1 (CH_3_CO), 20.1 (CH_3_ of Ts). HRMS (ESI) *m*/*z*; found, 541.0903 calcd for C_24_H_26_N_2_NaO_5_S_3_ [M + Na]^+^: 541.0896.

#### 4.2.14 *tert*-Butyl (rac)-3-((2-acetamidoethyl)­thio)-1-(hydroxymethyl)-2-Tosyl-7-azabicyclo[2.2.1]­hepta-2,5-diene-7-carboxylate (**16b**)

Starting from **16a** (240 mg, 0.53 mmol) and *N*-acetylcysteamine (57 mg, 0.48 mmol) in MeCN following the general procedure, afforded after chromatographic purification (EtOAc/Acetone 15:1) compound **16b** (71 mg, 0.14 mmol, 29%) as a yellow solid. ^1^H NMR (300 MHz, CD_3_OD, δ ppm): 7.62 (d, 2H, *J* = 8.4 Hz, Ar–H), 7.28 (d, 2H, *J* = 8.2 Hz, Ar–H), 6.79 (dd, 1H, *J* = 5.5, 3.0 Hz, H-5), 6.72 (d, 1H, *J* = 5.4 Hz, H-6), 5.70 (d, 1H, *J* = 2.9 Hz, H-4), 4.40–4.24 (m, 2H, CH_2_), 3.46–3.28 (m, 2H, H-2′), 3.11–3.02 (m, 2H, H-1′), 2.32 (s, 3H, CH_3_ of Ts), 1.87 (s, 3H, CH_3_CO), 1.25 (s, 9H, CH_3_ of Boc). ^13^C NMR (75 MHz, CD_3_OD, δ ppm): 172.3 (C=O), 153.9 (C=O) 145.7 (C-6), 144.8 (C-2 or C-3), 137.8 (C-5), 136.6 (C_q_Ar), 129.5, 126.6 (CH-Ar), 82.1 (C-1), 81.9 (C_q_Boc), 70.8 (C-4), 58.5 (CH_2_), 42.6 (C-2′), 40.2 (C-1′), 26.9 (CH_3_ of Boc), 21.1 (CH_3_ of Ts), 20.2 (CH_3_CO). HRMS (ESI) *m*/*z*; found, 517.1425 calcd for C_23_H_30_N_2_NaO_6_S_2_ [M + Na]^+^: 517.1437.

#### 4.2.15 *tert*-Butyl (rac)-2-((2-acetamidoethyl)­thio)-4-(hydroxymethyl)-1-methyl-3-Tosyl-7-azabicyclo[2.2.1]­hepta-2,5-diene-7-carboxylate (**17b**)

Starting from **17a** (200 mg, 0.43 mmol) and *N*-acetylcysteamine (46 mg, 0.39 mmol) in MeCN following the general procedure, afforded after chromatographic purification with EtOAc compound **17b** (70 mg, 0.14 mmol, 36%) as a brown oil. ^1^H NMR (300 MHz, CD_3_OD, δ ppm): 7.69 (d, 2H, *J* = 8.2 Hz, Ar–H), 7.30 (d, 2H, *J* = 8.3 Hz, Ar–H), 6.81 (d, 1H, *J* = 5.4 Hz, H-5 or H-6), 6.54 (d, 1H, *J* = 5.8 Hz, H-5 or H-6), 4.46–4.34 (m, 2H, CH_2_), 3.06–2.86 (m, 4H, H-1′, H-2′), 2.33 (s, 3H, CH_3_ of Ts), 1.91 (s, 3H, CH_3_), 1.83 (s, 3H, CH_3_CO), 1.27 (CH_3_ of Boc). ^13^C NMR (75 MHz, CD_3_OD, δ ppm): 171.9, 170.4 (C=O), 154.7, 152.2 (C-2, C-3), 145.0 (C_q_Ar), 144.8, 144.1 (C-5, C-6), 137.9 (C_q_Ar), 129.4, 127.2 (CH-Ar), 83.2, 82.1 (C-1, C-4), 82.0 (C_q_Boc), 59.8 (CH_2_), 39.4, 32.5 (C-1′, C-2′), 27.0 (CH_3_ of Boc), 21.2 (CH_3_ of Ts), 20.2 (CH_3_CO), 13.1 (CH_3_). HRMS (ESI) *m*/*z*; found, 531.1578 calcd for C_24_H_32_N_2_NaO_6_S_2_ [M + Na]^+^: 531.1594.

#### 4.2.16 *tert*-Butyl (rac)-2-((2-acetamidoethyl)­thio)-4-formyl-1-methyl-3-tosyl-7-azabicyclo[2.2.1]­hepta-2,5-diene-7-carboxylate (**18b**)

Starting from **18a** (130 mg, 0.28 mmol) and *N*-acetylcysteamine (30 mg, 0.25 mmol) in MeCN following the general procedure, afforded after chromatographic purification with EtOAc compound **18b** (56 mg, 0.11 mmol, 44%) as a brown oil. ^1^H NMR (300 MHz, CD_3_OD, δ ppm): 9.92 (s, 1H, CHO), 7.60 (d, 2H, *J* = 8.6 H, Ar–H), 7.33 (d, 2H, *J* = 8.2 Hz, Ar–H), 6.97 (d, 1H, *J* = 5.2 Hz, H-6), 6.62 (dd, 1H, *J* = 5.6, 1.0 Hz, H-5), 3.07–3.00 (m, 4H, H-1′, H-2′), 2.34 (s, 3H, CH_3_ of Ts), 1.89 (s, 3H, CH_3_), 1.85 (s, 3H, CH_3_CO), 1.28 (s, 9H, CH_3_ of Boc). ^13^C NMR (75 MHz, CD_3_OD, δ ppm): 190.8 (CHO), 172.0 (C=O), 168.2 (C=O), 154.3, 150.5 (C-2, C-3), 145.5 (C_q_Ar), 144.5 (C-5), 140.8 (C-6), 136.9 (C_q_Ar), 129.7, 127.3 (CH-Ar), 83.9, 83.4 (C-1, C-4), 39.3, 33.2 (C-1′, C-2′), 26.9 (CH_3_ of Boc), 21.1 (CH_3_ of Ts), 20.2 (CH_3_CO), 15.3 (CH_3_). HRMS (ESI) *m*/*z*; found, 529.1428 calcd for C_24_H_30_N_2_NaO_6_S_2_ [M + Na]^+^: 529.1437.

#### 4.2.17 N-(2-(((1*R*,4*S*)-3-tosylbicyclo­[2.2.1]­hepta-2,5-dien-2-yl)­thio)­ethyl)­acetamide (**19b**)

Starting from **19a** (400 mg, 1.22 mmol) and *N*-acetylcysteamine (121 mg, 1.02 mmol) in MeCN following the general procedure, afforded after chromatographic purification with EtOAc compound **19b** (140 mg, 0.39 mmol, 38%) as a brown oil. ^1^H NMR (300 MHz, CD_3_OD, δ ppm): 7.70 (d, 2H, *J* = 8.4 Hz, Ar–H), 7.39 (d, 2H, *J* = 8.4 Hz, Ar–H), 6.67–6.62 (m, 1H, H-5 or H-6), 6.58–6.53 (m, 1H, H-5 or H-6), 4.26 (br s, 1H, H-1 or H-4), 3.81 (br s, 1H, H-1 or H-4), 3.39–3.27 (m, 2H, H-1′ or H-2′), 3.17–3.05 (m, 2H, H-1′ or H-2′), 2.44 (s, 3H, CH_3_ of Ts), 2.10 (dt, 1H, *J* = 6.7, 1.4 Hz, CH_2_), 2.00 (dt, 1H, *J* = 6.8, 1.9 Hz, CH_2_), 1.96 (s, 3H, CH_3_CO). ^13^C NMR (75 MHz, CD_3_OD, δ ppm): 172.1, 166.3, 144.4, 142.2, 139.1, 138.0, 137.2, 129.4, 126.7, 68.3, 55.8, 52.8, 40.3, 30.7, 21.1, 20.2. HRMS (ESI) *m*/*z*; found, 386.0850 calcd for C_18_H_21_NNaO_3_S_2_ [M + Na]^+^: 386.0855.

### 4.3 General Procedure for Competition Experiments

To a solution of compound A (1.2 equiv) and B (1.2 equiv) in DMF (20 mL/mmol), phosphate buffer solution (pH 8.0, 50 mM, 25 mL/mmol) and a solution of *N*-acetylcysteamine (1.0 equiv) in DMF (4 mL/mmol) were added. The mixture was stirred at r.t. for 30 min and then, solvents were evaporated, and the residue was dissolved in EtOAc and washed with water. The aqueous phase was extracted with EtOAc (x3) and the combined organic phases were dried (Na_2_SO_4_), filtered and concentrated. Purification was performed by silica gel column chromatography (DCM/MeOH 100:1 → 15:1). % Conversion A/B into A'/B' was determined by ^1^H NMR of the starting material fraction recovered from the chromatographic purification.

#### 4.3.1 N-(2-((rac)-2-Bromo-3-Tosyl-7-azabicyclo­[2.2.1]­hepta-2,5-dien-7-yl)-2-oxoethyl)-5-(dimethylamino)­naphthalene-1-sulfonamide (**29**)

To a solution of **4a** (100 mg, 0.23 mmol) in DCM (4 mL), TFA (0.5 mL, 7 mmol) was added. The reaction mixture was stirred at r.t. for 30 min and then, the solvent was removed to afford crude unprotected AND. To a cooled (0 °C) solution of this compound (70 mg, 0.21 mmol) in anh. DCM (2 mL), dansylglycine chloride (190 mg, 0.53 mmol) in anh. DCM (5 mL) and Et_3_N (75 μL, 0.53 mmol) were added under Ar. The reaction mixture was stirred at r.t for 1.5 h. and then it was diluted with DCM and washed with H_2_O. The organic phase was separated, dried, filtered and concentrated. The resulting crude was purified by column chromatography on silica gel (EtOAc/Cy 1:1) to afford **29** (42 mg, 0.068 mmol, 30% three steps, pale yellow solid). ^1^H NMR (300 MHz, CDCl_3_, 298 K, δ ppm, mixture of rotamers): δ 8.56 (d, 1H, *J* = 8.7 Hz, Ar–H), 8.25 (d, 1H, *J* = 8.1 Hz, Ar–H), 8.18 (d, 1H, *J* = 7.1 Hz, Ar–H), 7.73 (d, 2H, *J* = 8.1 Hz, Ar–H), 7.60–7.47 (m, 2H, Ar–H), 7.20 (d, 1H, *J* = 7.2 Hz, Ar–H), 7.00–6.80 (m, 2H, H-5, H-6), 5.68–5.45 (m, 1H, H-1 or H-4), 5.42–5.31 (m, 1H, H-1 or H-4), 3.56 (dd, 1H, *J* = 15.8, 5.3 Hz, CH_2_), 3.35 (dd, 1H, *J* = 16.0, 3.6 Hz, CH_2_), 2.89 (s, 6H, N­(CH_3_)_2_), 2.43 (s, 3H, CH_3_ of Ts). ^13^C NMR (75 MHz, CDCl_3_, 298 K, δ ppm, mixture of rotamers): δ 163.5 (C=O), 150.2, 146.0.145.7, 144.5 (C_q_ Ar), 142.6, 140.4 (C-5, C-6), 135.2, 133.9, (C_q_ Ar), 130.8, 130.4 (CH-Ar), 129.6 (C_q_ Ar), 129.4, 128.6, 127.8, 123.2, 118.9, 115.5 (CH-Ar), 72.7, 66.7 (C-1, C-4), 45.5 (N­(CH_3_)_2_), 44.2 (CH_2_), 21.8 (CH_3_ of Ts). HRESIMS *m*/*z*; found, 616.0571 calcd for C_27_H_27_^79^BrN_3_O_5_S_2_ [M + H]^+^, 616.0570; found, 618.0548 calcd for C_27_H_27_^81^BrN_3_O_5_S_2_ [M + H]^+^, 618.0549.

### 4.4 Protein Bioconjugation

#### 4.4.1 Synthesis of Bioconjugate **A**

500 μL protein solution in PBS (pH 7.4) (concentration 74 μM) was reduced for 20 min at 37 °C using 5 equiv of TCEP. After checking completion of the reduction step by LC–MS, 36.5 μL of DMSO were added followed by a solution of compound **29** in DMSO (18.5 μL, 10 mM, 5eq) and the mixture was incubated at rt for 1 h. After accomplishing full conversion (as determined by LC–MS analysis), bioconjugate **A** was purified by size exclusion chromatography using a 10/300 Superdex Increase 75 GL column (Cytiva, Little Chalfont, UK) and PBS (pH 7.4) as an elution buffer. Calculated mass 16,773 Da, Observed 16,769 Da.

#### 4.4.2 GSH Stability Assay

Four μL of a 10 mM glutathione (GSH) solution (33.1 mg GSH in 1.08 mL PBS, adjusted to pH 7.4) was added at room temperature to 96 μL of bioconjugate **A** (4 μM in PBS) and mixed thoroughly by pipetting up and down, resulting in a final GSH concentration of 400 μM. The mixture was incubated at 37 °C. Aliquots were taken at 5, 15, 30, 60, 120, and 240 min, and immediately flash-frozen in liquid nitrogen. Samples were analyzed by SDS–PAGE to observe the decrease in fluorescence associated with pyrrole loss (Figure S32, ProQ Emerald 300 channel). The final time point (240 min) was also analyzed by LC–MS (Figure S33), confirming the presence of bioconjugate **B** (expected: 16,723 Da; found: 16,721 Da) and intermediate bioconjugate **C** (expected: 17,080 Da; found: 17,076 Da).

#### 4.4.3 Plasma and HSA Stability Studies

The stability of a sample of conjugate **A** was tested in two separate experiments upon incubation with human plasma (Sigma-Aldrich, Cat. H4522) or human serum albumin (HSA, Sigma-Aldrich Cat. A3782). In short, 10 μL of a 15 μM solution of the conjugate in PBS were added to 40 μL of either human plasma or HSA (50 mg/mL) at room temperature and mixed thoroughly by pipetting up and down. The resulting mixture were incubated at 37 °C. Time points were taken at 0, 1, 2,3 and 4 days, and immediately flash frozen in liquid N_2_. Samples were analyzed by SDS–PAGE (Figure S33).

## Supplementary Material



## Notes

ORTEP of compounds **4a**, **6a**, **8a**, **12a**, **15a** and **23a**; synthesis of starting materials; details for the synthesis and characterization data of **4a**-**22a**; details for competition and fragmentation experiments; bioconjugation and stability studies; ^1^H- and ^13^C NMR spectra for new compounds (PDF)

## References

[1] Qian L., Lin X., Gao X., Khan R. U., Liao J. Y., Du S., Ge J., Zeng S., Yao S. Q., Yao S. Q. (2023). The Dawn of a New Era: Targeting
the “Undruggables” with Antibody-Based Therapeutics. Chem. Rev..

[2] Yang X., Pan Z., Choudhury M. R., Yuan Z., Anifowose A., Yu B., Wang W., Wang B. (2020). Making smart drugs smarter: The importance
of linker chemistry in targeted drug delivery. Med. Res. Rev..

[3] Bargh J. D., Isidro-Llobet A., Parker J. S., Spring D. R. (2019). Cleavable linkers
in antibody–drug conjugates. Chem. Soc.
Rev..

[4] Forman H. J., Zhang H., Rinna A. (2009). Glutathione:
Overview of its protective
roles, measurement, and biosynthesis. Mol. Aspects
Med..

[5] Russo A., DeGraff W., Friedman N., Mitchell J. B. (1986). Selective modulation
of glutathione levels in human normal versus tumor cells and subsequent
differential response to chemotherapy drugs. Cancer Res..

[6] Pillow T. H., Sadowsky J. D., Zhang D., Yu S. – F., Del Rosario G., Xu K., He J., Bhakta S., Ohri R., Kozak K. R., Ha E., Junutula J. R., Flygare J. A. (2017). Decoupling stability and release
in disulfide bondswith
antibody-small molecule conjugates. Chem. Sci..

[7] Danial M., Postma A. (2017). Disulfide Conjugation Chemistry: A Mixed Blessing for
Therapeutic Drug delivery?. Ther. Delivery.

[8] Hong V., Kislukhin A. A., Finn M. G. (2009). Thiol-Selective
Fluorogenic Probes for Labeling and Release. J. Am. Chem. Soc..

[9] Kislukhin A. A., Higginson C. J., Hong V. P., Finn M. G. (2012). Degradable
Conjugates from Oxanorbornadiene Reagents. J.
Am. Chem. Soc..

[10] Gil de Montes E., Jiménez-Moreno E., Oliveira B. L., Navo C. D., Cal P. M. S. D., Jiménez-Osés G., Moreno-Vargas A. J., Robina I., Bernardes G. J. L. (2019). Azabicyclic Vinyl Sulfones for Residue-Specific
Dual Protein Labelling. Chem. Sci..

[11] Gil de Montes E., Istrate A., Navo C. D., Jiménez-Moreno E., Hoyt E. A., Corzana F., Robina I., Jiménez-Osés G., Moreno-Vargas A. J., Bernardes G. J. L. (2020). Stable Pyrrole-Linked Bioconjugates
through Tetrazine-Triggered Azanorbornadiene Fragmentation. Angew. Chem., Int. Ed..

[12] Carranza M., Carmona A. T., Navo C. D., Robina I., Fratta S., Newburn C., Jiménez-Osés G., Moreno-Vargas A. J. (2023). Experimental
and Theoretical Analysis of the Thiol-Promoted Fragmentation of 2-Halo-3-tosyl-oxanorbornadienes. Org. Lett..

[13] Moreno-Clavijo E., Moreno-Vargas A. J., Carmona A. T., Robina I. (2013). Strain-Promoted Retro-Dieckmann-Type
Condensation on [2.2.2]- and [2.2.1]­Bicyclic Systems: A Fragmentation
Reaction for the Preparation of Functionalized Heterocycles and Carbocycles. Org. Biomol. Chem..

[14] Chen Z., Trudell M. L. (1996). Chemistry of 7-Azabicyclo[2.2.1]­hepta-2,5-dienes,
7-Azabicyclo[2.2.1]­hept-2-enes
and 7-Azabicyclo[2.2.1]­heptanes. Chem. Rev..

[15] Otani Y., Nagae O., Naruse Y., Inagaki S., Ohno M., Yamaguchi K., Yamamoto G., Uchiyama M., Ohwada T. (2003). An Evaluation
of Amide Group Planarity in 7-Azabicyclo[2.2.1]­heptane Amides. Low
Amide Bond Rotation Barrier in Solution. J.
Am. Chem. Soc..

[16] The twist angle |τ| (Table 1) was calculated as the absolute value of the mean of two torsion angles in N-acyl derivatives, ω_1_(RCNC_1_) and ω_2_(OCNC_4_) (|τ| = 1/2|ω_1_+ω_2_|).

[17] Ohwada T., Okamoto I., Shudo K. (1998). On the Planarity of
Amide Nitrogen.
Intrinsic Pyramidal Nitrogen of N-Acyl-7-Azabicyclo[2.2.1]­heptanes. Tetrahedron Lett..

[18] Ohwada T., Okamoto I., Shudo K., Yamaguchi K. (1998). Intrinsic
Pyramidal Nitrogen of N-Sulfonylamides. Tetrahedron
Lett..

[19] Blahun O. P., Rozhenko A. B., Rusanov E., Zhersh S., Tolmachev A. A., Volochnyuk D. M., Grygorenko O. O. (2020). Twisting and Turning the Sulfonamide
Bond: A Synthetic, Quantum Chemical, and Crystallographic Study. J. Org. Chem..

[20] Thioketal intermediate from AND **15b** was formed very slowly, taking 3.8 h for complete formation, and the further rDA fragmentation of the corresponding thioketal intermediate was even slower (48% of fragmentation after 80 h).

[21] Chatani S., Nair D. P., Bowman C. N. (2013). Relative Reactivity and Selectivity
of Vinyl Sulfones and Acrylates Towards the Thiol–Michael Addition
Reaction and Polymerization. Polym. Chem..

[22] García-Domínguez J., Carranza M., Jansons E., Carmona A. T., Robina I., Moreno-Vargas A. J. (2023). Transferring Substituents from Alkynes to Furans and
Pyrroles through Heteronorbornadienes as Intermediates: Synthesis
of β-Substituted Pyrroles/Furans. J. Org.
Chem..

