# Supplementary material for: [2.2.1]Heterobicyclic Bromovinyl Sulfones for Thiol-Triggered Strategies in Linker Chemistry: Aza- vs Oxa-Norbornadienic Systems
Source: Bioconjug Chem. 2025 Aug 22;36(9):2079–89. doi: 10.1021/acs.bioconjchem.5c00371 (PMC12447399; doi:10.1021/acs.bioconjchem.5c00371)
Supplement: Supplementary file 1 [file bc5c00371_si_001.pdf]

SUPPORTING INFORMATION

[2.2.1]Heterobicyclic bromovinyl sulfones for thiol-triggered strategies in linker chemistry: aza- *vs* oxa-norbornadienic systems

Marina Carranza,[a] Ana T. Carmona,[a,*] Celia Maya,[b] Enrique Gil de Montes,[c] Aldrin V. Vasco,[c] Gonçalo J. L. Bernardes[c,d] and Antonio J. Moreno-Vargas[a,*]

[a] *Departamento de Química Orgánica, Facultad de Química, Universidad de Sevilla, Sevilla, 41012, Spain.*

[b] *Instituto de Investigaciones Químicas (IIQ), Departamento de Química Inorgánica and Centro de Innovación en Química Avanzada (ORFEO−CINQA), Consejo Superior de Investigaciones, Científicas (CSIC) and Universidad de Sevilla, 41092 Sevilla, Spain.*

[c] *Yusuf Hamied Department of Chemistry, University of Cambridge, Lensfield Road, CB2 1EW Cambridge, UK.*

[d] *Translational Chemical Biology Group, Spanish National Cancer Research Centre (CNIO), C/ Melchor Fernández Almagro, 3. 28029 Madrid, Spain.*

TABLE OF CONTENTS

1. Crystal structures of compounds **4a**, **6a**, **8a**, **12a**, **15a** and **23a** S2

2. Synthesis of starting material **S1-S8** S6

3. Diels-Alder (DA) reaction for the preparation of halo-ANDs **4a-8a, 15a-22a** S7

4. Synthesis of AND **18a** S11

5. Synthesis of bromo-ANDs **9a-14a** S11

6. Competition experiments S15

7. Studies of the fragmentation of thio-ANDs via ^{1}H-NMR S25

8. Synthesis of dansyl-derivatives. Fluorescence emission plots S43

9. Bioconjugation studies S45

10. ^{1}H and ^{13}C-NMR spectra for new compounds S50

11. References S116

1. Crystal structures of compounds **4a**, **6a**, **8a**, **12a**, **15a** and **23a**.

Crystallographic details. Low-temperature diffraction data were collected on a D8 Quest APEX-III single crystal diffractometer with a Photon III detector and a IµS 3.0 microfocus X-ray source (**4a**, **6a**, **8a**, **12a**, **15a** and **23a**) at the Instituto de Investigaciones Químicas, Sevilla. Data were collected by means of ω and ϕ scans using monochromatic radiation λ(Mo Kα1) = 0.71073 Å. The diffraction images collected were processed and scaled using APEX-III software. Using Olex2[1], all structures were solved with SHELXT and were refined against F^2 on all data by full-matrix least squares with SHELXL.[2] All non-hydrogen atoms were refined anisotropically. Hydrogen atoms were included in the model at geometrically calculated positions and refined using a riding model. The isotropic displacement parameters of all hydrogen atoms were fixed to 1.2 times the U value of the atoms to which they are linked (1.5 times for methyl groups).

A summary of the fundamental crystal and refinement data are given in Tables S1 and S2. Atomic coordinates, anisotropic displacement parameters and bond lengths and angles can be found in the cif files, which have been deposited in the Cambridge Crystallographic Data Centre with no. 2393736-2393741. These data can be obtained free of charge from The Cambridge Crystallographic Data Centre via www.ccdc.cam.ac.uk/data_request/cif.

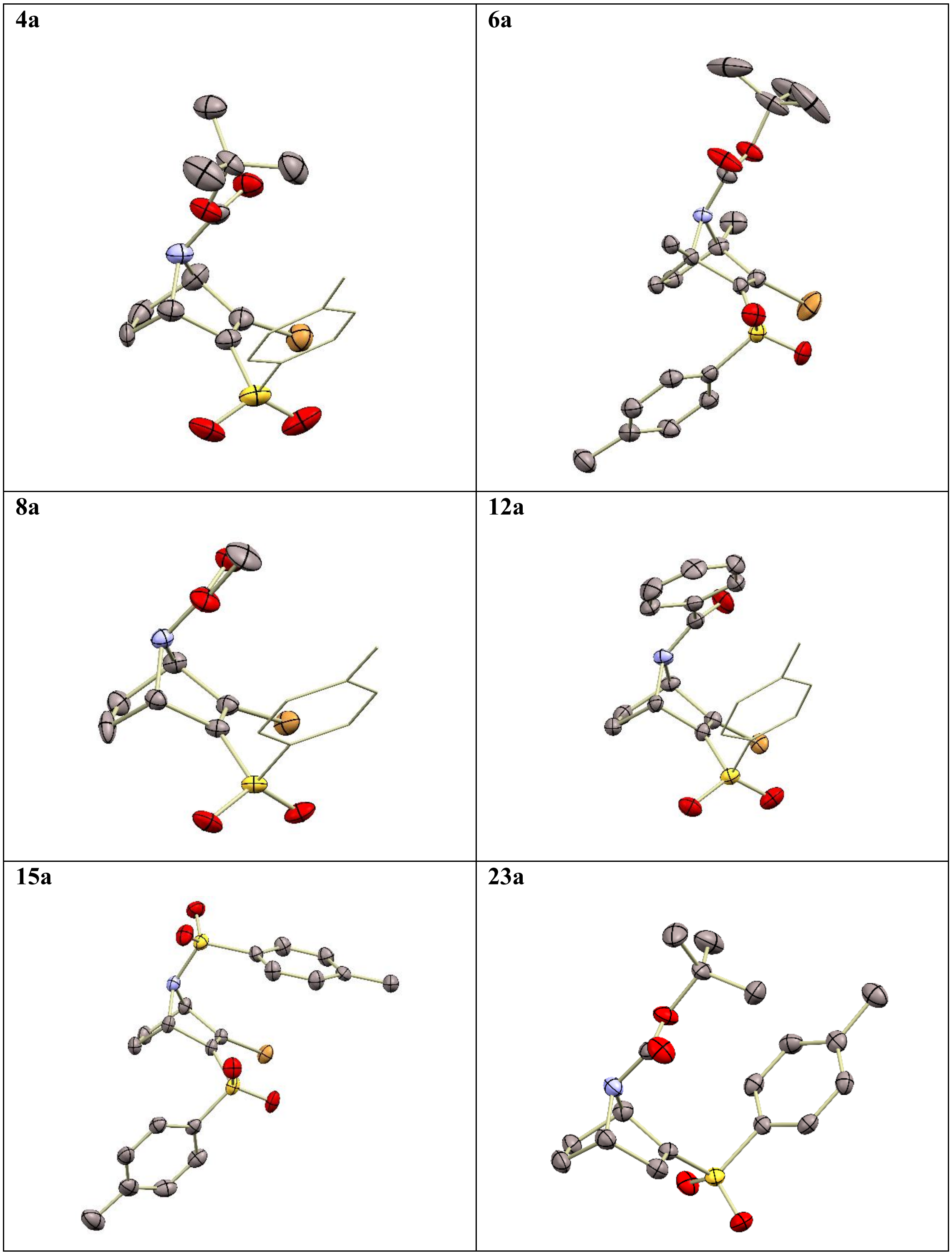

Figure S1. ORTEP of compounds **4a**, **6a**, **8a**, **12a**, **15a** and **23a**. For the sake of clarity hydrogen atoms are excluded, while some fragments are represented in wireframe format and thermal ellipsoids are set at 50% of probability.

Table S1. Crystal data and structure refinement for compounds **4a**, **6a** and **8a**.

	**4a**	**6a**	**8a**
formula	$C_{18}H_{20}BrNO_4S$	$C_{20}H_{24}BrNO_4S$	$C_{30}H_{28}Br_2N_2O_8S_2$
Fw	426.32	454.37	768.48
cryst.size, mm	0.21 × 0.18× 0.15	0.24 × 0.21 × 0.14	0.43 × 0.2 × 0.1
crystal system	Triclinic	Monoclinic	Monoclinic
space group	*P-1*	$P2_1/c$	$P2_1/c$
a, Å	9.7152(6)	11.9267(5)	8.5251(3)
b, Å	11.7987(8)	12.3552(5)	11.8396(4)
c, Å	25.0137(18)	14.3881(6)	15.7778(6)
α, deg	93.649(2)	90	90
β, deg	95.661(2)	96.173(2)	90.8450(10)
γ, deg	90.865(2)	90	90
V, $Å^3$	2846.8(3)	2107.89(15)	1592.34(10)
T, K	193	193	193
Z	6	4	2
ρ_{calc}, g cm^{-3}	1.492	1.432	1.603
μ, mm^{-1} (MoKα)	2.298	2.073	2.729
F(000)	1308	936	776
absorption corrections	multi-scan, 0.62 – 0.75	multi-scan, 0.59 – 0.75	multi-scan, 0.57 – 0.75
ϑ range, deg	2.30 – 21.33	4.36 – 58.00	2.39 – 30.53
no. of rflns measd	97943	132614	64907
R_{int}	0.1796	0.0986	0.0745
no. of rflns unique	12444	5588	3470
no. of params / restraints	688 / 0	266 / 6	205 / 0
R_1 ($I > 2\sigma(I)$) [a]	0.0595	0.0358	0.0460
R_1 (all data)	0.1210	0.0537	0.0524
wR_2 ($I > 2\sigma(I)$)	0.1170	0.0823	0.1147
wR_2 (all data)	0.1399	0.0920	0.1182
Diff.Fourier.peaks min/max, e$Å^{-3}$	-0.73 / 1.29	-0.75 / 0.31	-0.58 / 2.97
CCDC number	2393736	2393737	2393738

Table S2. Crystal data and structure refinement for compounds **12a**, **15a** and **23a**.

	**12a**	**15a**	**23a**
formula	$C_{20}H_{16}BrNO_3S$	$C_{20}H_{18}BrNO_4S_2$	$C_{18}H_{21}NO_4S$
Fw	430.31	480.38	347.42
cryst.size, mm	0.26 × 0.24 × 0.23	0.52 × 0.13 × 0.11	0.25 × 0.23 × 0.15
crystal system	Monoclinic	Monoclinic	Orthorhombic
space group	$P2_1/c$	$P2_1/c$	$P2_12_12_1$
a, Å	7.9400(6)	12.6336(11)	11.0299(11)
b, Å	11.7666(10)	8.0088(7)	11.4625(11)
c, Å	19.3821(15)	40.107(3)	13.8043(14)
α, deg	90	90	90
β, deg	90.588(3)	94.210(3)	90
γ, deg	90	90	90
V, Å^3	1810.7(2)	4047.1(6)	1745.3(3)
T, K	193	193	193
Z	4	8	4
ρ_{calc}, g cm^{-3}	1.578	1.577	1.322
μ, mm^{-1} (MoKα)	2.405	2.264	0.207
F(000)	872	1952	736
absorption corrections	multi-scan, 0.58 – 0.75	multi-scan, 0.55 – 0.75	multi-scan, 0.64 – 0.75
ϑ range, deg	4.204 – 53.996	4.074 – 57.996	4.618 – 51.988
no. of rflns measd	64215	95331	41663
R_{int}	0.0721	0.0533	0.1419
no. of rflns unique	3919	10742	3394
no. of params / restraints	236 / 0	509 / 0	221 / 0
R_1 ($I > 2\sigma(I)$) [a]	0.0936	0.0301	0.0433
R_1 (all data)	0.0968	0.0360	0.0590
wR_2 ($I > 2\sigma(I)$)	0.2442	0.0730	0.0832
wR_2 (all data)	0.2453	0.0754	0.0893
Diff.Fourier.peaks min/max, eÅ^{-3}	-2.25 / 2.04	-0.46 / 0.40	-0.27 / 0.18
CCDC number	2393739	2393740	2393741

2. **Synthesis of starting material S1-S8**

2.1 Synthesis of activated alkynes

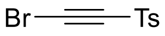

S1

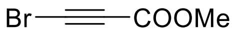

S2

- Alkyne **S1** was prepared following a procedure previously described by our research group[3] and previously characterized.[4]
- Alkyne **S2** was synthesized from commercially available methyl propiolate, following standard protocol previously reported.[5]

2.2 Synthesis of pyrrole derivatives

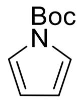

S3

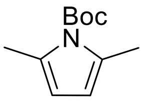

S4

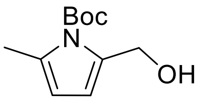

S5

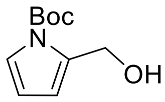

S6

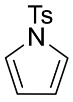

S7

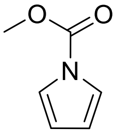

S8

- Pyrroles **S3, S7** and **S8** were available from commercial sources.
- Pyrrole **S4** was synthesized following a procedure previously described by our research group.[6]
- Pyrrole **S6** was synthesized following standard protocol previously reported.[7]

- Synthesis of *tert*-butyl 2-(hydroxymethyl)-5-methyl-1H-pyrrole-1-carboxylate (**S5**)

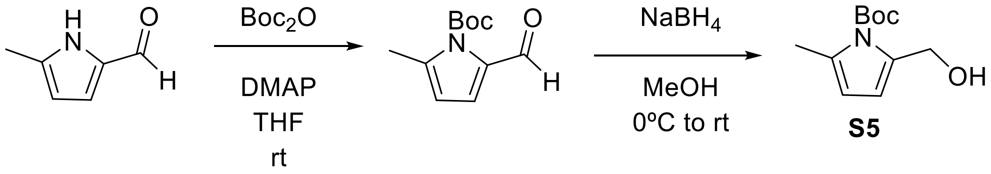

To a solution of 5-methylpyrrol-1H-pyrrole-2-carboxaldehyde (1.0 g, 9.2 mmol) in THF (20 mL), DMAP (56 mg, 0.46 mmol) and Boc_2O (2.4 g, 11.0 mmol) were added, and the reaction mixture was stirred at room temperature for 30 minutes. After the reaction was completed, the mixture was washed with saturated aqueous solution of $NaHCO_3$ (20 mL). The aqueous phase was extracted with EtOAc (3 x 50 mL), and the combined organic phases were dried with Na_2SO_4 anh., filtered and evaporated. The resulting compound was dissolved in MeOH (12 mL), cooled to 0ºC, and $NaBH_4$ (346 mg, 9.16 mmol) was added. The reaction was stirred

at room temperature for 30 minutes and then, it was quenched with cold water (15 mL) and extracted with Et_2O (3 x 30 mL). The organic phase was washed with saturated aqueous solution of $NaHCO_3$, dried with Na_2SO_4 anh., filtered and evaporated. The resulting crude was purified by chromatography column on silica gel (EtOAc: CyHex 1:5) to obtain compound **S5** (1.20 g, 5.68 mmol, 62%) as a light brown oil. ^{1}H NMR ($CDCl_3$, 300 MHz, δ ppm): 6.07 (d, 1H, *J*= 3.0 Hz, H-3), 5.87-5.84 (m, 1H, H-4), 4.60 (d, 2H, *J*= 7.0 Hz, CH_2), 3.55 (t, 1H, *J*=7.0 Hz, OH), 2.39 (d, 3H, *J*=1.0 Hz, CH_3), 1.64 (s, 9H, CH_3 of Boc). ^{13}C NMR ($CDCl_3$, 75 MHz, δ ppm): 151.1 (C=O), 135.2, 132.4 (C-3, C-4), 112.5, 111.0 (C-2, C-5), 84.7 (C_q of Boc), 58.4 (CH_2), 28.2 (CH_3 of Boc), 16.8 (CH_3). HRMS (ESI) m/z: found, 234.1101; calcd. for $C_{11}H_{17}NNaO_3$ $[M+Na]^+$: 234.1101.

3. Diels-Alder (DA) reaction for the preparation of bromo-HNDs 4a-8a, 15a-22a.

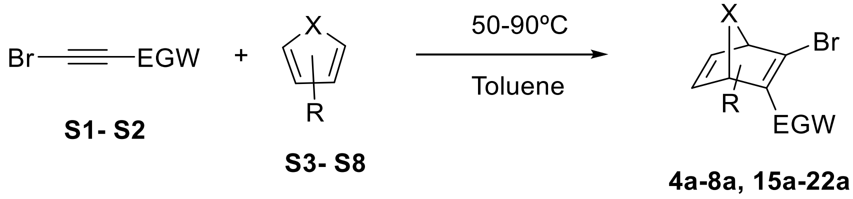

- Bromo-HNDs **4a**, **19a**-**22a** have been previously described in our group.[8]
- Bromo-AND **5a** was synthesized following the procedure previously reported.[9]

General procedure for the preparation of bromo-ANDs via DA reaction

To a solution of activated alkyne **S1** or **S2** (x mmol) in toluene (2 mL/mmol), the corresponding commercial or synthetic pyrrole derivative **S3**-**S8** (z mmol) was added, and the reaction mixture was stirred at 50-90 °C. After the reaction was completed, the solvent was evaporated, and the resulting residue was purified by a chromatography column on silica gel to give the corresponding bromo-AND. When two regioisomers were expected, NOE experiments were performed for structure elucidation.

(*rac*)-*tert*-Butyl-2-bromo-1,4-dimethyl-3-tosyl-7-azabicyclo[2.2.1]hepta-2,5-diene-7-carboxylate (6a)

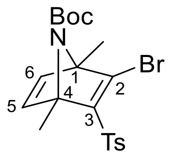

Reaction of pyrrole **S4** (226 mg, 1.16 mmol) and alkyne **S1** (100 mg, 0.39 mmol) following the general procedure (55 °C, 24 h), afforded after chromatographic purification (EtOAc:CyHex 1:10) compound **6a** (102 mg, 0.22 mmol, 56%) as a yellow liquid. The compound was recrystallized (2% EtOAc in Hexane). ^{1}H NMR (300 MHz, $CDCl_3$, δ ppm): δ 7.76 (d, 2H, *J*= 8.3 Hz, Ar-H), 7.31 (d, 2H, *J*= 8.1 Hz, Ar-H), 6.56 (s, 2H, H-5, H-6), 2.43 (s, 3H, CH_3 of Ts), 1.95 (s, 3H, CH_3), 1.88 (s, 3H, CH_3), 1.29 (s, 9H, CH_3 of Boc). ^{13}C NMR (75.4 MHz, $CDCl_3$, δ ppm): δ 154.9 (C=O of Boc), 154.3, 151.5 (C-2, C-3), 148.1, 145.2 (C-5, C-6), 144.9, 137.2 (C_qAr), 129.8, 127.7 (CH-Ar), 81.8, 80.2 (C-1, C-4), 79.4 (C_q of Boc), 28.0 (CH_3 of Boc), 21.7 (CH_3 of Ts), 17.5, 16.7 (CH_3). HRMS (ESI) m/z: found, 476.0497; calcd. for $C_{20}H_{24}{}^{79}BrNNaO_4S$ $[M+Na]^+$: 476.0507; m/z: found, 478.0474; calcd. for $C_{20}H_{24}{}^{81}BrNNaO_4S$ $[M+Na]^+$: 478.0487.

(*rac*)-7-(*tert*-Butyl)-2-methyl-3-bromo-1,4-dimethyl-7-azabicyclo[2.2.1]hepta-2,5-diene-2,7-dicarboxylate (7a)

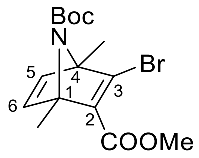

Reaction of pyrrole **S4** (300 mg, 1.54 mmol) and alkyne **S2** (1.2 g, 7.68 mmol) following the general procedure (70 °C, 14 h), afforded after chromatographic purification (EtOAc:CyHex 1:8) compound **7a** (316 mg, 0.88 mmol, 57%) as a brown oil. ^{1}H NMR (300 MHz, $CDCl_3$, δ ppm): δ 6.75 (d, 1H, *J*= 5.5 Hz, H-5 or H-6), 6.66 (d, 1H, *J*= 5.7 Hz, H-5 or H-6), 3.79 (s, 3H, CH_3 of COOMe), 2.02 (s, 3H, CH_3), 1.89 (s, 3H, CH_3), 1.41 (s, 9H, CH_3 of Boc). ^{13}C NMR (75.4 MHz, $CDCl_3$, δ ppm): δ 164.0 (C=O of COOMe), 154.6 (C=O of Boc), 153.6 (C-2 or C-3), 148.1, 146.1 (C-5, C-6), 146.0 (C-2 or C-3), 81.4 (C-1 or C-4), 79.3 (C_q of Boc), 78.5 (C-1 or C-4), 51.6 (CH_3 of COOMe), 28.3 (CH_3 of Boc), 17.6, 17.0 (CH_3). HRMS (ESI) m/z: found, 380.0467; calcd. for $C_{15}H_{20}{}^{79}BrNNaO_4$ $[M+Na]^+$: 380.0473; m/z: found, 382.0446; calcd. for $C_{15}H_{20}{}^{81}BrNNaO_4$ $[M+Na]^+$: 382.0447.

(*rac*)-Methyl 2-bromo-3-tosyl-7-azabicyclo[2.2.1]hepta-2,5-diene-7-carboxylate (8a)

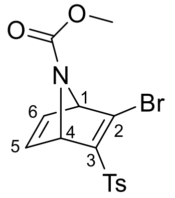

Reaction of pyrrole **S8** (1.20 g, 9.65 mmol) and alkyne **S1** (500 mg, 1.93 mmol) following the general procedure (80 °C, 15 h), afforded after chromatographic purification (EtOAc: CyHex 1:5) compound **8a** (375 mg, 0.98 mmol, 51%) as a white solid. The compound was recrystallized in 2% EtOAc in Hexane. ^{1}H NMR (300 MHz, $CDCl_3$, δ ppm): 7.81 (d, 2H, *J*=8.4 Hz, Ar-H), 7.38 (d, 2H, *J*=8.3 Hz, Ar-H), 7.03-6.96 (m, 2H, H-5, H-6), 5.45-5.43 (m, 1H, H-1 or H-4), 5.23 (br.s, 1H, H-1 or H-4), 3.55 (br.s, 3H, CH_3 of COOMe), 2.47 (s, 3H, CH_3 of Ts). ^{13}C NMR (75 MHz, $CDCl_3$, δ ppm): 154.7 (C=O), 145.3 (C-5 or C-6), 143.0 (C_q), 139.8 (C-5 or C-6), 136.2 (C_q), 130.0, 127.8 (CH-Ar), 75.3, 69.3 (C-1, C-4), 53.2 (COO$\underline{C}H_3$ of COOMe), 21.7 (CH_3 of Ts). HRMS (ESI) m/z: found, 405.9705; calcd. for $C_{15}H_{14}{}^{79}BrNNaO_4S$ [M+Na]$^+$: 405.9719; m/z: found, 407.9683; calcd. for $C_{15}H_{14}{}^{81}BrNNaO_4S$ [M+Na]$^+$: 407.9666.

(*rac*)-2-Bromo-3,7-ditosyl-7-azabicyclo [2.2.1]hepta-2,5-diene (**15a**)

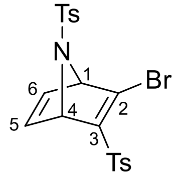

Reaction of pyrrole **S7** (853 mg, 3.86 mmol) and alkyne **S1** (200 mg, 0.77 mmol) following the general procedure (90 °C, 6 h), afforded after chromatographic purification (EtOAc:CyHex 1:4) compound **15a** (159 mg, 0.31 mmol, 40%) as a yellow solid. ^{1}H NMR (300 MHz, $CDCl_3$, δ ppm): 7.66 (d, 2H, *J*= 8.2 Hz, Ar-H), 7.60 (d, 2H, *J*=8.3 Hz, Ar-H), 7.34-7.30 (m, 4H, Ar-H), 6.87 (ddd, 1H, *J*=5.9, 2.8, 0.8 Hz, H-5 or H-6), 6.80 (ddd, 1H, *J*= 5.2, 2.7, 0.6 Hz, H-5 or H-6), 5.32 (td, 1H, *J*=2.5, 0.7 Hz, H-1 or H-4), 5.08 (t, 1H, *J*= 2.7 Hz, H-1 or H-4), 2.44 (s, 3H, CH_3 of Ts), 2.43 (s, 3H, CH_3 of Ts). ^{13}C NMR (75.4 MHz, $CDCl_3$, δ ppm): 149.2, 145.5, 144.7, 143.8, 143.6, 140.0, 135.2, 134.2, 130.3, 130.1, 128.6, 127.9, 75.8, 69.9, 21.8, 21.7. HRMS (ESI) m/z: found, 501.9748; calcd. for $C_{20}H_{18}{}^{79}BrNNaO_4S_2$ [M+Na]$^+$: 501.9758; m/z: found, 503.9725; calcd. for $C_{20}H_{18}{}^{81}BrNNaO_4S_2$ [M+Na]$^+$: 507.9732.

(*rac*)-*tert*-Butyl 3-bromo-1-(hydroxymethyl)-2-tosyl-7-azabicyclo[2.2.1]hepta-2,5-diene-7-carboxylate (16a)

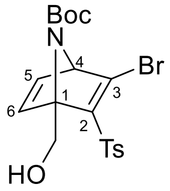

Reaction of pyrrole **S6** (250 mg, 1.27 mmol) and alkyne **S1** (493 mg, 1.90 mmol) following the general procedure (80 °C, 2 h), afforded after chromatographic purification (Et_2O: CyHex 1:2) compound **16a** (178 mg, 0.39 mmol, 31%, regioisomer ratio 7:1) as a brown oil. ^{1}H NMR (300 MHz, $CDCl_3$, δ ppm, data for major regioisomer): 7.79 (d, 2H, *J*=8.3 Hz, Ar-H), 7.32 (d, 2H, *J*=8.1 Hz, Ar-H), 7.06 (d, 1H, *J*=5.6 Hz, H-6), 6.96 (dd, 1H, *J*=5.0, 2.9 Hz, H-5), 5.12 (d, 1H, *J*= 5.1 Hz, H-4), 4.58 (dd, 1H, *J*= 12.0, 6.7 Hz, CH_2), 4.40 (dd, 1H, *J*=13.5, 6.4 Hz, 3.52 (br. s, 1H, OH), 2.44 (s, 3H, CH_3 of Ts), 1.33 (s, 9H, CH_3 of Boc). ^{13}C NMR (75.4 MHz, $CDCl_3$, δ ppm, major regioisomer): 153.2 (C=O), 146.1 (C-6), 145.2 (C-5), 139.0, 136.9 (C_qAr), 129.9, 127.7 (CH-Ar), 82.7 (C_q of Boc), 77.2 (C-4), 75.6 (C-1), 59.5 (CH_2), 28.0 (CH_3 of Boc), 21.7 (CH_3 of Ts). HRMS (ESI) m/z: found, 478.0279; calcd. for $C_{19}H_{22}{}^{79}BrNNaO_5S$ $[M+Na]^+$: 478.0294; m/z: found, 480.0257; calcd. for $C_{19}H_{22}{}^{81}BrNNaO_5S$ $[M+Na]^+$: 480.0274.

(*rac*)-*tert*-Butyl 3-bromo-1-(hydroxymethyl)-2-tosyl-7-azabicyclo[2.2.1]hepta-2,5-diene-7-carboxylate (17a)

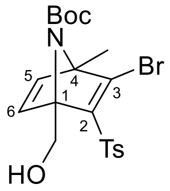

Reaction of pyrrole **S5** (500 mg, 2.36 mmol) and alkyne **S1** (740 mg, 2.84 mmol) following the general procedure (80 °C, 2 h), afforded after chromatographic purification (Et_2O: CyHex 1:2) compound **17a** (228 mg, 0.49 mmol, 21%, regioisomer ratio 5:1) as a brown oil. ^{1}H NMR (300 MHz, $CDCl_3$, δ ppm, data for major regioisomer): 7.78 (dd, 2H, *J*=8.1 Hz, Ar-H), 7.31 (dd, 2H, *J*=8.1 Hz, Ar-H), 7.04 (d, 1H, *J*= 5.4 Hz, H-5), 6.58 (d, 1H, *J*=5.5 Hz, H-6), 4.56 (dd, 1H, *J*=12.3, 7.3 Hz, CH_2), 4.37 (dd, 1H, *J*=12.6, 6.3 Hz, CH_2), 3.29 (ap t, 1H, *J*=8.1 Hz, OH), 2.42 (s, 3H, CH_3 of Ts), 1.87 (s, 3H, CH_3), 1.32 (s, 9H, CH_3 of Boc). ^{13}C NMR (75.4 MHz, $CDCl_3$, δ ppm,

major regioisomer): 153.6, 151.8 (C-2, C-3), 145.4 (C-5), 145.2 (C-6), 145.1, 137.0 (C_qAr), 129.8, 127.6 (CH-Ar), 84.3 (C-1 or C-4), 82.8 (C_q of Boc). 80.2 (C-1 or -4), 60.3 (CH_2), 28.0 (CH_3 of Boc), 21.7 (CH_3 of Ts), 16.7 (CH_3). HRMS (ESI) m/z: found, 492.0443; calcd. for $C_{20}H_{14}{}^{79}BrNNaO_5S$ $[M+Na]^+$: 492.0451; m/z: found, 494.0420; calcd. for $C_{20}H_{14}{}^{79}BrNNaO_5S$ $[M+Na]^+$: 494.0436.

4. **Synthesis of (*rac*)-*tert*-butyl 2-bromo-4-formyl-1-methyl-3-tosyl-7-azabicyclo[2.2.1]hepta-2,5-diene-7-carboxylate (18a)**

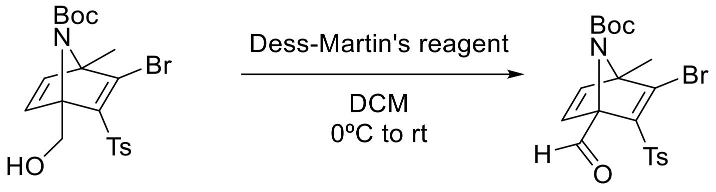

17a **18a**

A solution of compound **17a** (150 mg, 0.32 mmol) in DCM (4 mL) was cooled at 0°C Then, Dess-Martin's reagent was added, and the reaction mixture was stirred at room temperature for 2 hours. Then, the mixture was diluted with DCM and washed sequentially with saturated aqueous solution of $NaHCO_3$/25 M $NaSO_3$, and brine. The organic phase was dried with Na_2SO_4 anh., filtered and the resulting crude was purified by chromatography column on silica gel (EtOAc: CyHex 1:2) to obtain compound **18a** (119 mg, 0.25 mmol, 80%) as a brown oil. ^1H NMR (300 MHz, $CDCl_3$, δ ppm): 9.26 (s, 1H, CHO), 7.60 (d, 2H, J=8.3 Hz, Ar-H), 7.26 (d, 2H, J=8.1 Hz, Ar-H), 6.98 (d, 1H, J=5.0 Hz, H-5), 6.48 (dd, 1H, J= 5.1, 1.0 Hz, H-6), 2.37 (s, 3H, CH_3 of Ts), 1.88 (s, 3H, CH_3), 1.30 (s, 3H, CH_3 of Boc). ^{13}C NMR (75 MHz, $CDCl_3$, δ ppm): 190.0 (CHO), 153.8 (C=O), 152.2. 150.3 (C-2, C-3), 145.5 (C_qAr), 143.8 (C-6), 141.6 (C-5), 135.4 (C_qAr), 130.0, 127.8 (CH-Ar), 84.4, 84.0 (C-1, C-4), 81.9 (C_q of Boc), 28.0 (CH_3 of Boc), 21.8 (CH_3 of Ts), 16.0 (CH_3). HRMS (ESI) m/z: found, 490.0283; calcd. for $C_{20}H_{22}{}^{79}BrNNaO_5S$ $[M+Na]^+$: 490.0294; m/z: found, 492.0262 calcd. for $C_{20}H_{22}{}^{81}BrNNaO_5S$ $[M+Na]^+$: 492.0273.

5. **Synthesis of bromo-ANDs 9a-14a**

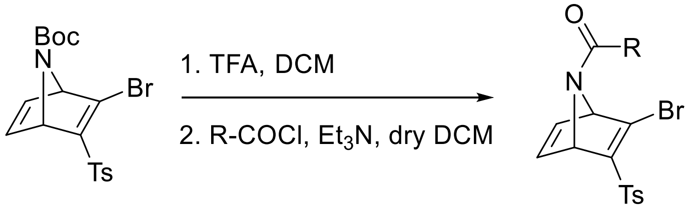

4a **9a-14a**

General procedure

To a solution of **4a** (x mmol, 1 eq.) in DCM (1 mL/mmol), TFA (y mmol, 30 eq.) was added, and the reaction mixture was stirred at r.t. for 30 min. Then, the solvent was co-evaporated with toluene and the crude product was dissolved in dry DCM (2 mL/mmol) under Ar and cooled to 0° C. Then, the corresponding acyl chloride (z mmol, 4 eq.) and triethylamine (t mmol, 6 eq.) were added, and the reaction was stirred at r.t. for 1h. After evaporation, the resulting residue was purified by chromatography column on silica gel to give the corresponding AND.

1-((*rac*)-2-Bromo-3-tosyl-7-azabicyclo[2.2.1]hepta-2,5-dien-7-yl)ethan-1-one (9a)

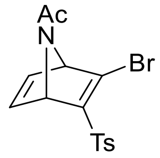

Acidic deprotection of **4a** (206 mmol, 0.48 mmol) and acylation with acetyl chloride following the general procedure afforded after chromatographic purification (EtOAc; CyHex 1:3) compound **9a** (131 mg, 0.36 mmol, 75%) as a brown oil. ^{1}H NMR (300 MHz, $CDCl_3$, δ ppm, major rotamer): 7.80-7.56 (m, 2H, Ar-H), 7.36 (d, 2H, *J*= 8.2 Hz, Ar-H), 7.06-6.93 (m, 2H, H-5, H-6), 5.68 (s, 1H, H-1 or H-4), 5.19 (s, 1H, H-1 or H-4), 2.45 (s, 3H, CH_3 of Ts), 1.78 (s, 3H, CH_3CO). ^{13}C NMR (75 MHz, $CDCl_3$, δ ppm, mixture of rotamers): 167.9, 147.1, 145.6, 144,9, 142.8, 140.5, 139.6, 135.7, 130.11, 127.9, 72.9, 68.8, 21.7, 21.1. HRMS (ESI) m/z: found, 389.9768; calcd. for $C_{15}H_{14}{}^{79}BrNNaO_3S$ $[M+Na]^+$: 389.9768, m/z: found, 391.9746; calcd. for $C_{15}H_{14}{}^{81}BrNNaO_3S$ $[M+Na]^+$: 391.9749.

1-((*rac*)-2-Bromo-3-tosyl-7-azabicyclo[2.2.1]hepta-2,5-dien-7-yl)butan-1-one (10a)

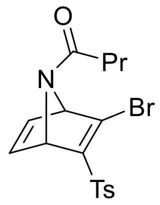

Acidic deprotection of **4a** (300 mmol, 0.70 mmol) and acylation with butanoyl chloride following the general procedure afforded after chromatographic purification (EtOAc:CyHex 1:3) compound **9a** (216 mg, 0.54 mmol, 77%) as a brown solid. ^{1}H NMR (300 MHz, CD_3CN, δ

ppm, mixture of rotamers): 7.82-7.77 (m, 2H, Ar-H), 7.50-7.45 (m, 2H, Ar-H), 7.07-7.05 (m, 1H, H-5 or H-6), 7.00-6.98 (m, 1H, H-5 or H-6), 5.62-5.60 (m, 1H, H-1 or H-4), 5.50-5.46 (m, 1H, H-1 or H-4), 2.47 (s, 3H, CH_3 of Ts), 2.12-2.00 (m, 2H, $\underline{CH_2}$-CH_2-CH_3), 1.54-1.30 (m, 2H, CH_2-$\underline{CH_2}$-CH_3), 0.87-0.78 (m, 3H, CH_2-CH_2-$\underline{CH_3}$). ^{13}C NMR (75 MHz, CD_3CN, δ ppm, mixture of rotamers): 151.3, 148.7, 147.2, 145.4, 144.5, 144.1, 141.2, 141.0, 136.6, 131.4, 131.2, 128.7, 75.6, 74.1, 69.6, 67.7, 36.0, 21.7, 18.6, 18.3, 13.9. HRMS (ESI) m/z: found, 418.0074; calcd. for $C_{17}H_{18}{}^{79}BrNNaO_3S$ $[M+Na]^+$: 418.0083; m/z: found, 420.0052; calcd. for $C_{17}H_{18}{}^{81}BrNNaO_3S$ $[M+Na]^+$: 420.0068.

1-((*rac*)-2-Bromo-3-tosyl-7-azabicyclo[2.2.1]hepta-2,5-dien-7-yl)-2,2,2-trifluoroethan-1-one (11a)

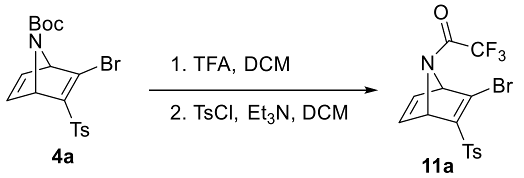

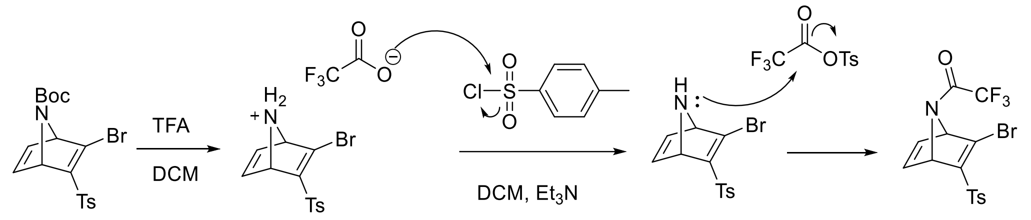

Acidic deprotection of **4a** (250 mmol, 0.58 mmol) and acylation with tosyl chloride following the general procedure afforded after chromatographic purification (EtOAc:CyHex 1:3) compound **11a** (175 mg, 0.41 mmol, 72%) as a brown oil.^{1}H NMR (300 MHz, CD_3OD, δ ppm, mixture of rotamers): 7.83 (d, 2H, *J*=7.9 Hz, Ar-H), 7.49 (d, 2H, *J*=8.0 Hz, Ar-H), 7.25-7.18 (m, 1H, H-5 or H-6), 7.15 (dd, 1H, *J*=5.5, 2.5 Hz, H-5 or H-6), 5.88-5.84 (m, 1H, H-1 or H-4), 5.75-5.70 (m, 1H, H-1 or H-4), 2.47 (s, 3H, CH_3 of Ts). ^{13}C NMR (75 MHz, CD_3OD, δ ppm, mixture of rotamers): δ 146.2, 142.8, 142.6, 139.8, 135.4, 130.1, 127.6, 73.8, 72.5, 67.8, 66.6, 20.3. HRMS (ESI) m/z: found, 443.9483; calcd. for $C_{17}H_{18}{}^{79}BrF_3NNaO_3S$ $[M+Na]^+$: 443.9487; m/z: found, 445.9459; calcd. for $C_{17}H_{18}{}^{81}BrF_3NNaO_3S$ $[M+Na]^+$: 445.9467.

((*rac*)-2-Bromo-3-tosyl-7-azabicyclo[2.2.1]hepta-2,5-dien-7-yl)(phenyl)methanone (12a)

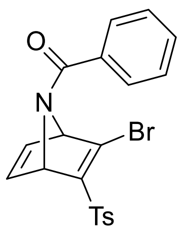

Acidic deprotection of **4a** (380 mmol, 0.89 mmol) and acylation with benzoyl chloride following the general procedure afforded after chromatographic purification (EtOAc:CyHex 1:4) compound **12a** (314 mg, 0.73 mmol, 82%) as a brown oil. The compound was recrystallized in 2% DCM in Hexane. ^{1}H NMR (500 MHz, $CDCl_3$, 253 K, δ ppm, major rotamer): 7.65 (d, 2H, *J*=8.4 Hz, Ar-H), 7.50-7.48 (m, 1H, Ar-H), 7.35 (t, 2H, *J*=7.7 Hz, Ar-H), 7.30-7.25 (m, 4H, Ar-H), 7.21 (dd, 1H, *J*= 5.6, 2.0 Hz, H-5 or H-6), 7.02 (dd, 1H, *J*=5.1, 2.5 Hz, H-5 or H-6), 5.62 (br.s, 1H, H-1 or H-4), 5.44 (br.s, 1H, H-1 or H-4), 2.46 (s, 3H, CH_3 of Ts). ^{13}C NMR (125 MHz, $CDCl_3$, 253 K, δ ppm, major rotamer): 168.5, 149.3, 147.4, 145.5, 142.7, 141.3, 135.5, 132.1, 130.3, 128.7, 128.1, 127.6, 73.3, 71.0, 22.1. HRMS (ESI) m/z: found, 451.9921; calcd. for $C_{20}H_{16}{}^{79}BrNNaO_3S$ [M+Na]$^+$: 451.9926; m/z: found, 453.9899; calcd. for $C_{20}H_{16}{}^{81}BrNNaO_3S$ [M+Na]$^+$: 453.9906.

((*rac*)-2-Bromo-3-tosyl-7-azabicyclo[2.2.1]hepta-2,5-dien-7-yl)(4-methoxyphenyl)methanone (13a)

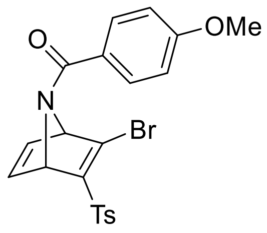

Acidic deprotection of **4a** (300 mmol, 0.70 mmol) and acylation with *p*-methoxybenzoyl chloride following the general procedure afforded after chromatographic purification (EtOAc:CyHex 1:5) compound **13a** (225 mg, 0.52 mmol, 74%) as a dark brown oil. ^{1}H NMR (500 MHz, $CDCl_3$, 253 K, δ ppm, major rotamer): 7.57 (d, 2H, *J*=8.5 Hz, Ar-H), 7.21-7.18 (m, 4H, Ar-H), 7.11 (dd, 1H, *J*=5.2, 2.5 Hz, H-5 or H-6), 6.93 (dd, 1H, *J*=5.6, 2.4 Hz, H-5 or H-6), 6.75-6.72 (m, 2H, Ar-H), 5.48 (br.s, 1H, H-1 or H-4), 5.39 (br.s, 1H, H-1 or H-4), 3.80 (s, 3H, OCH_3), 2.38 (s, 3H, CH_3 of Ts). ^{13}C NMR (125 MHz, $CDCl_3$, 253 K, δ ppm, major rotamer): 169.7, 163.2, 149.9, 148.6, 146.2, 143.6, 142.1, 136.0, 131.1, 131.0, 128.3, 125.0, 114.5, 74.5, 72.2,

55.8, 22.1. HRMS (ESI) m/z: found, 482.0020; calcd. for $C_{21}H_{18}{}^{79}BrNNaO_4S$ $[M+Na]^+$: 482.0032; m/z: found, 483.9998; calcd. for $C_{21}H_{18}{}^{81}BrNNaO_4S$ $[M+Na]^+$: 484.0012.

((*rac*)-2-Bromo-3-tosyl-7-azabicyclo[2.2.1]hepta-2,5-dien-7-yl)(4-nitrophenyl)methanone (14a)

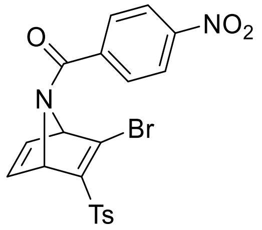

Acidic deprotection of **4a** (300 mmol, 0.70 mmol) and acylation with *p*-nitrobenzoyl chloride following the general procedure afforded after chromatographic purification (EtOAc:CyHex 1:6) compound **14a** (167 mg, 0.37 mmol, 53%) as a pale yellow solid. ^{1}H NMR (500 MHz, $(CD_3)_2CO$, 253 K, δ ppm, major rotamer): 8.18 (d, 2H, *J* = 9.5 Hz, Ar-H), 7.55 (d, 2H, *J* = 8.4 Hz, Ar-H), 7.51 (d, 2H, *J* = 8.7 Hz, Ar-H), 7.30-7.26 (m, 3H, Ar-H, H-5, H-6), 7.21 (dd, 1H, *J* = 5.2, 1.0 Hz, H-1 or H-4), 5.55-5.53 (m, 1H, H-1 or H-4), 2.37 (s, 3H, CH_3 of Ts). ^{13}C NMR (125 MHz, $(CD_3)_2CO$, 253 K, δ ppm, mixture of rotamers): 167.7, 150.2, 149.9, 149.5, 146.6, 144.8, 143.9, 140.7, 140.5, 138.3, 136.7, 136.4, 131.7, 131.1, 130.3, 128.2, 124.6, 74.5, 71.8, 55.1, 22.0. HRMS (ESI) m/z: found, 496.9765; calcd. for $C_{20}H_{15}{}^{79}BrN_2NaO_5S$ $[M+Na]^+$: 496.9777; m/z: found, 498.9743; calcd. for $C_{20}H_{15}{}^{81}BrN_2NaO_5S$ $[M+Na]^+$: 498.9757.

6. Competition experiments

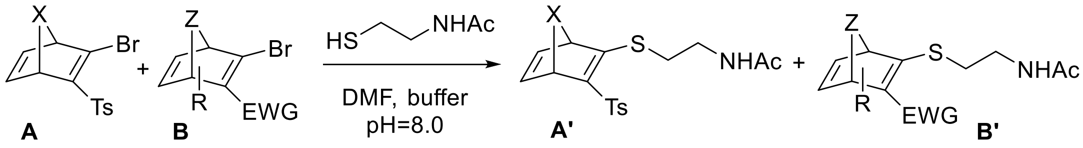

- Competition experiment **3a** *vs* **4a**

Starting from *N*-acetylcysteamine (5.0 mg, 0.042 mmol) following the general procedure. % Conversion of **3a**:**4a** into **3b**:**4b** was 61:39 (determined by ^{1}H NMR).

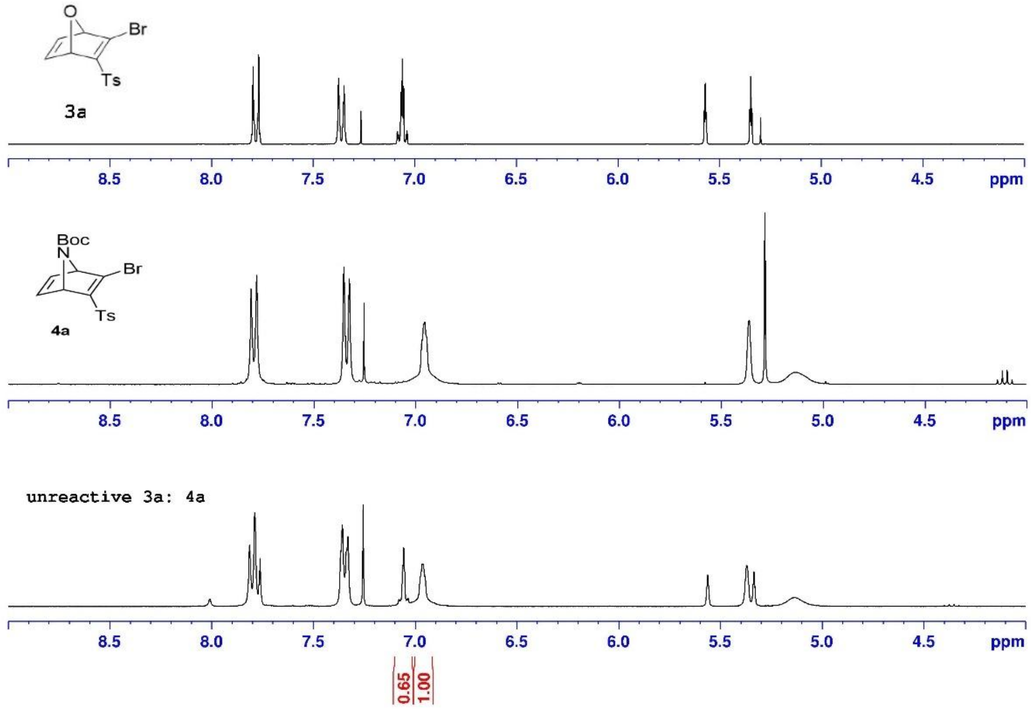

Figure S2. ^{1}H-NMR (300 MHz, $CDCl_3$) of the competition experiment **3a**:**4a** *vs* *N*-acetylcysteamine.

- Competition experiment **3a** *vs* **9a**

Starting from *N*-acetylcysteamine (5.0 mg, 0.042 mmol) following the general procedure. % Conversion of **3a**:**9a** into **3b**:**9b** was 37:63 (determined by ^{1}H NMR).

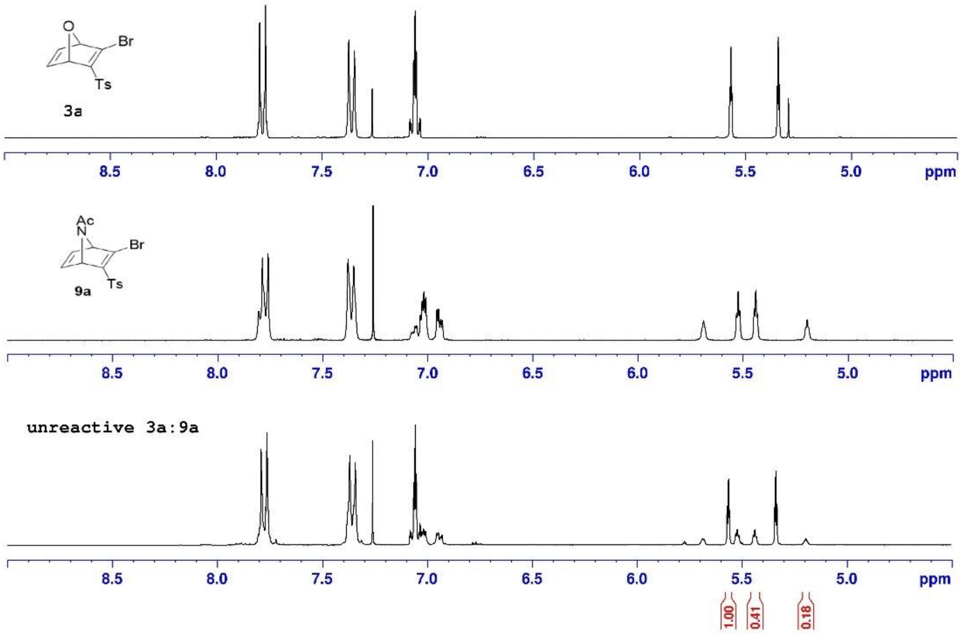

Figure S3. ^{1}H-NMR (300 MHz, $CDCl_3$) of the competition experiment **3a**:**9a** *vs* *N*-acetylcysteamine

- Competition experiment **4a** *vs* **8a**

  Starting from *N*-acetylcysteamine (5.0 mg, 0.042 mmol) following the general procedure. % Conversion of **4a**:**8a** into **4b**:**8b** was 40:60 (determined by ^{1}H NMR).

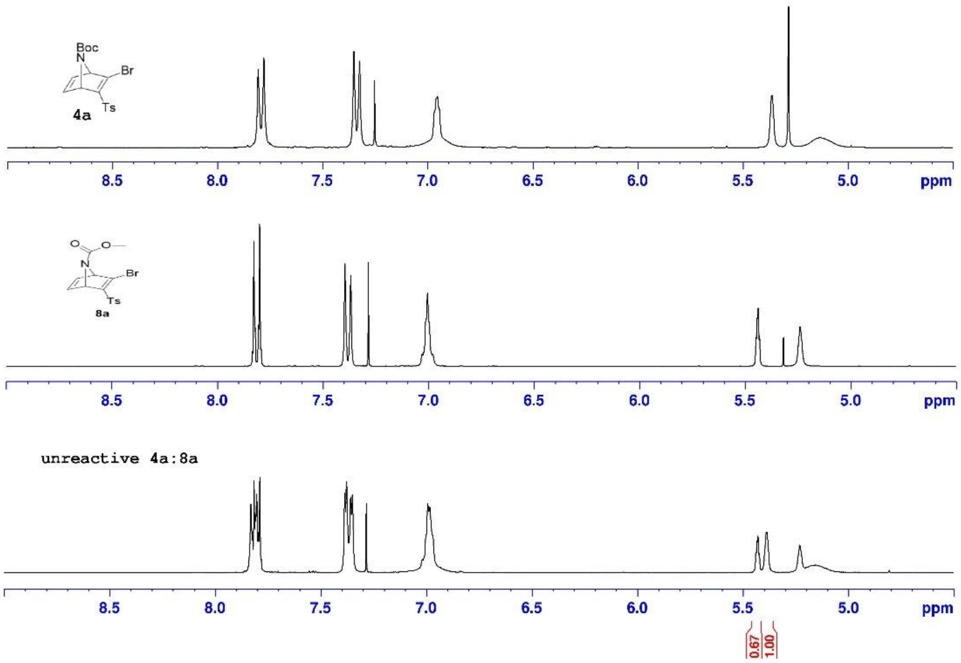

Figure S4. ^{1}H-NMR (300 MHz, $CDCl_3$) of the competition experiment **4a**:**8a** *vs* *N*-acetylcysteamine

- Competition experiment **4a** *vs* **11a**

  Starting from *N*-acetylcysteamine (5.0 mg, 0.042 mmol) following the general procedure. %Conversion of **4a**:**11a** into **4b**:**11b** was 0:100 (determined by ^{1}H NMR).

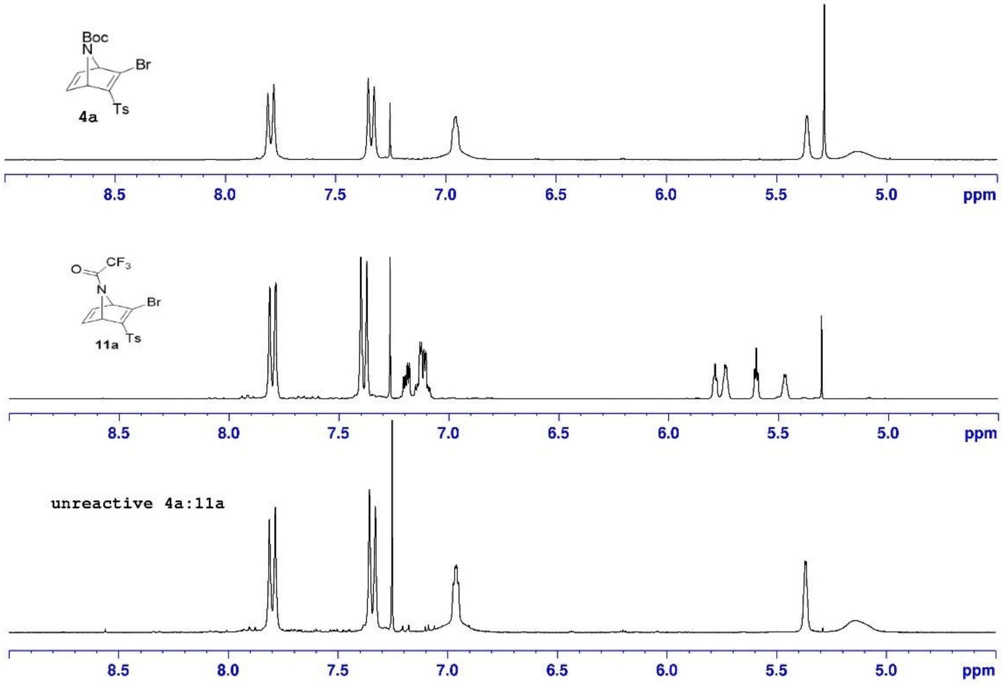

Figure S5. ^{1}H-NMR (300 MHz, $CDCl_3$) of the competition experiment **4a**:**11a** *vs* *N*-acetylcysteamine

- Competition experiment **4a** *vs* **12a**

  Starting from *N*-acetylcysteamine (8.1 mg, 0.068 mmol) following the general procedure. %Conversion of **4a**:**12a** into **4b**:**12b** was 27:73 (determined by ^{1}H NMR).

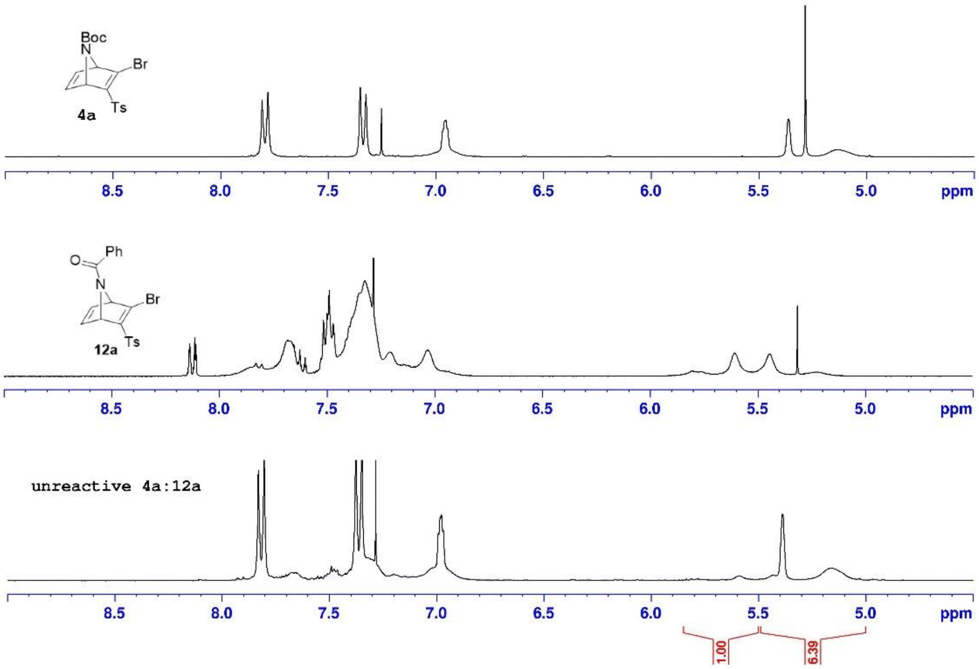

Figure S6. ^{1}H-NMR (300 MHz, $CDCl_3$) of the competition experiment **4a**:**12a** *vs* *N*-acetylcysteamine

- Competition experiment **3a** *vs* **19a**

Starting from *N*-acetylcysteamine (9 mg, 0.08 mmol) following the general procedure. %Conversion of **3a**:**19a** into **3b**:**19b** was 66:34 (determined by ^{1}H NMR).

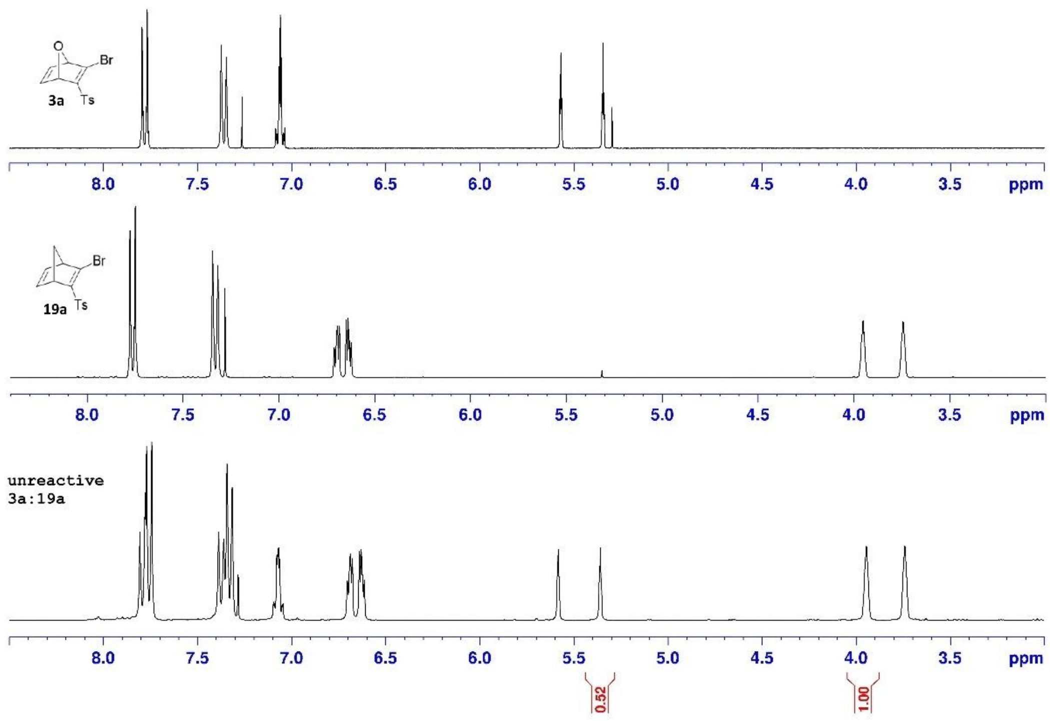

Figure S7. ^{1}H-NMR (300 MHz, $CDCl_3$) of the competition experiment **3a**:**19a** *vs* *N*-acetylcysteamine

- Competition experiment **3a** *vs* **20a**

  Starting from *N*-acetylcysteamine (9 mg, 0.08 mmol) following the general procedure. % Conversion of **3a**:**20a** into **3b**:**20b** was 82:18 (determined by ^{1}H NMR).

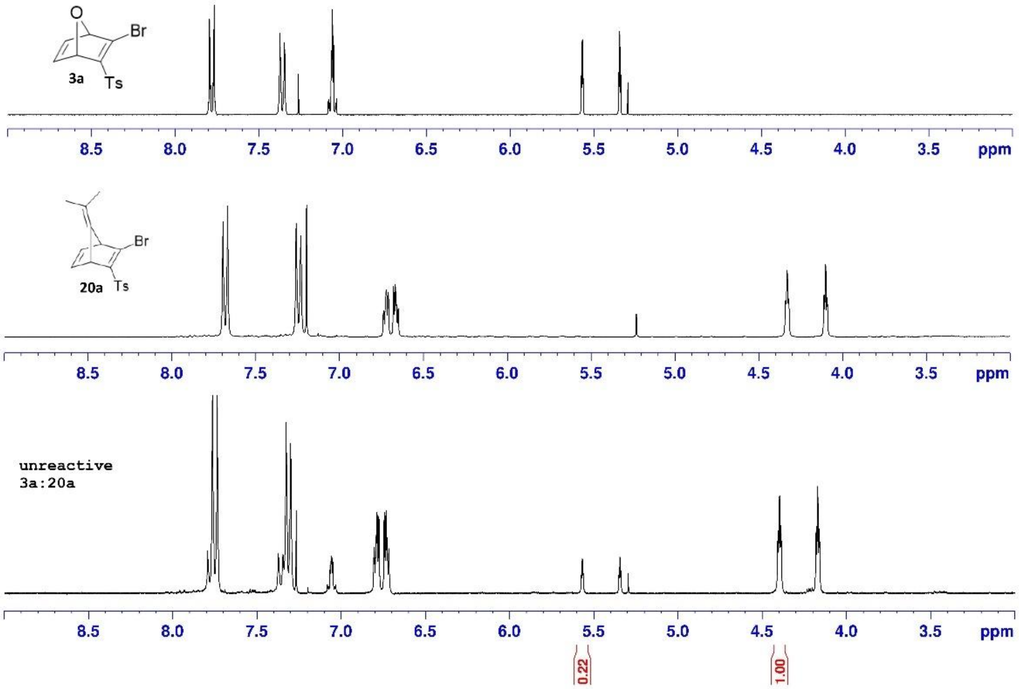

Figure S8. ^{1}H-NMR (300 MHz, $CDCl_3$) of the competition experiment **3a**:**20a** *vs* *N*-acetylcysteamine

- Competition experiment **3a** *vs* **21a**

  Starting from *N*-acetylcysteamine (9.0 mg, 0.08 mmol) following the general procedure. % Conversion of **3a**:**21a** into **3b:21b** was 73:27 (determined by ^{1}H NMR).

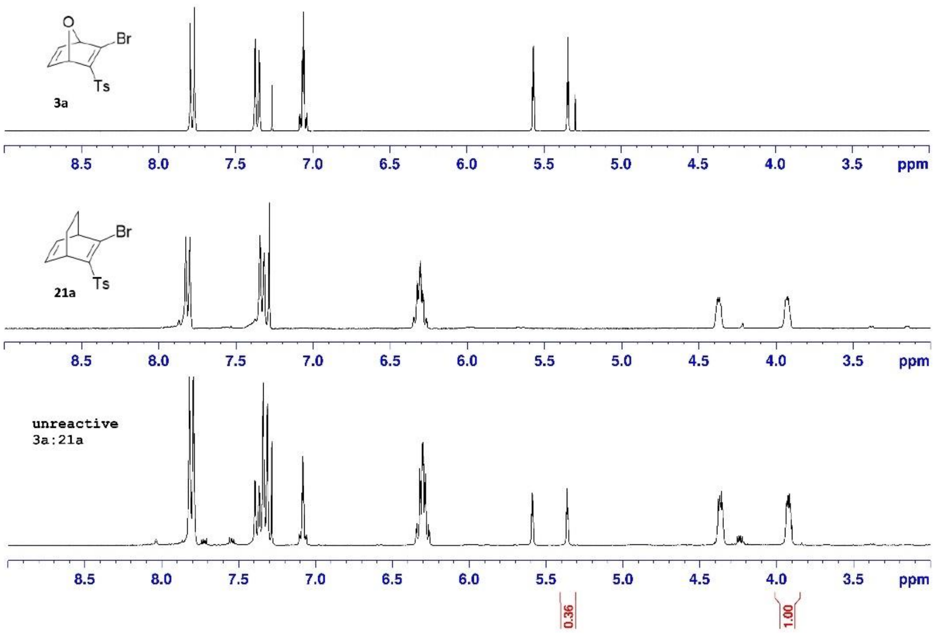

Figure S9. ^{1}H-NMR (300 MHz, $CDCl_3$) of the competition experiment **3a**:**21a** *vs* *N*-acetylcysteamine

- Competition experiment **3a** *vs* **22a**

  Starting from *N*-acetylcysteamine (6.0 mg, 0.05 mmol) following the general procedure. % Conversion of **3a**:**22a** into **3b**:**22b** was 92:8 (determined by ^{1}H NMR).

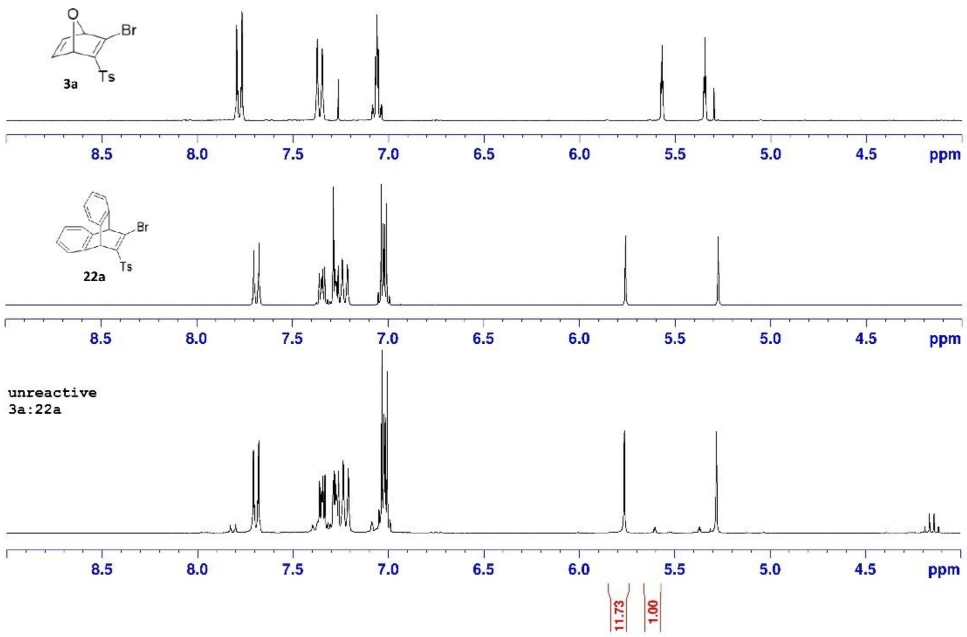

Figure S10. ^{1}H-NMR (300 MHz, $CDCl_3$) of the competition experiment **3a**:**22a** *vs* *N*-acetylcysteamine

- Competition experiment **6a** *vs* **7a**

  Starting from *N*-acetylcysteamine (8.0 mg, 0.07 mmol) following the general procedure. %Conversion of **6a**:**7a** into **6b**:**7b** was 35:65 (determined by ^{1}H NMR).

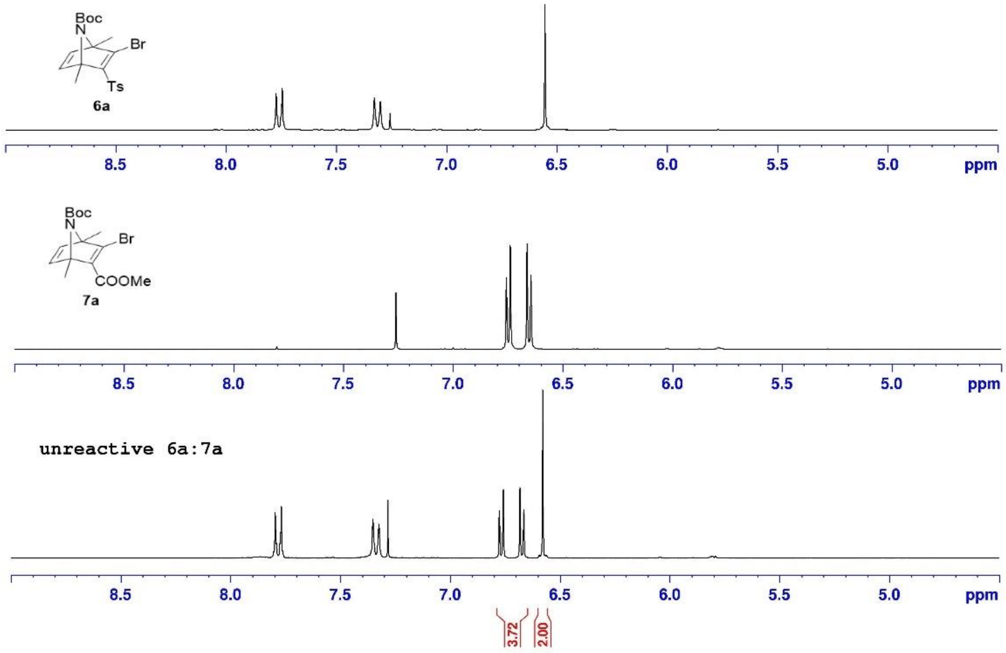

Figure S11. ^{1}H-NMR (300 MHz, $CDCl_3$) of the competition experiment **6a**:**7a** *vs* *N*-acetylcysteamine

7. Studies of the fragmentation of thio-ANDs *via* ^{1}H-NMR.

General procedure: Compound **B'** (0.04 mmol) was dissolved in CD_3OD or DMSO-d_6 (0.5 mL) in a NMR tube. Then, *N*-acetylcysteamine (0.05 mmol) and triethylamine (0.05 mmol) in MeOD or DMSO-d_6 (0.1 mL) were added, and the reaction was left to complete at 30 °C. ^{1}H-NMR spectra were registered at regular intervals.

Representative examples

a) Thioketal intermediate not observed in the ^{1}H NMR monitoring of the fragmentation

- ^{1}H NMR monitoring for the fragmentation of **6b**

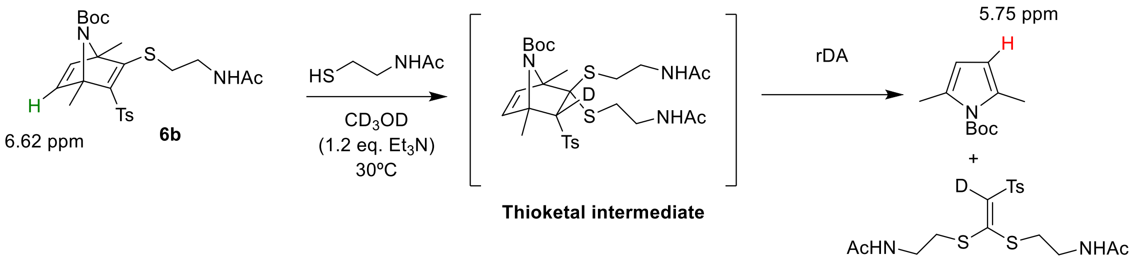

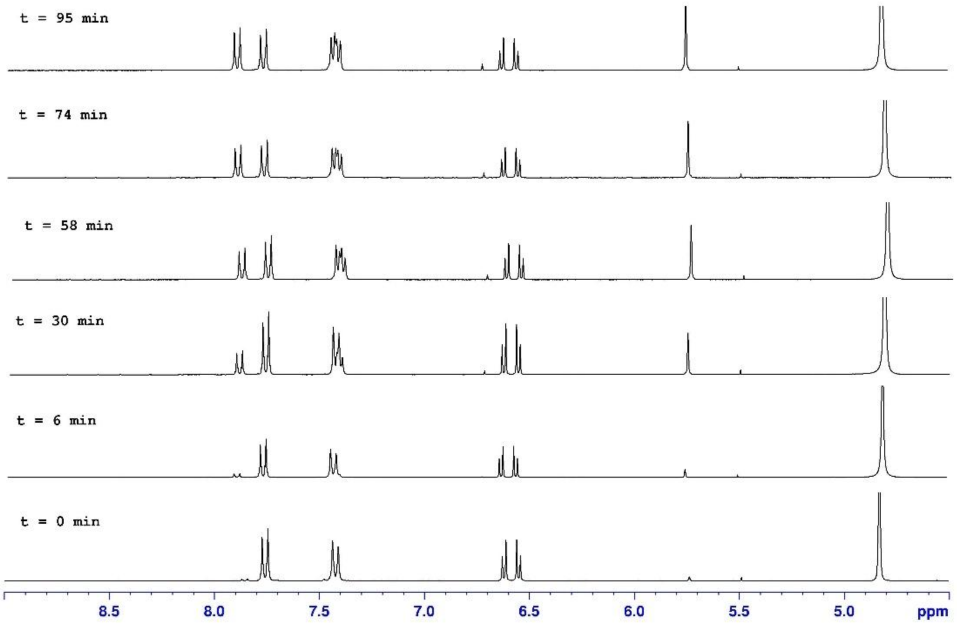

Figure S12. ^{1}H NMR (300 MHz, CD_3OD, 303 K) for the fragmentation of **6b**.

b) Starting thio-AND not observed in the ^{1}H NMR monitoring of the fragmentation

- ^{1}H NMR monitoring for the fragmentation of **11b**

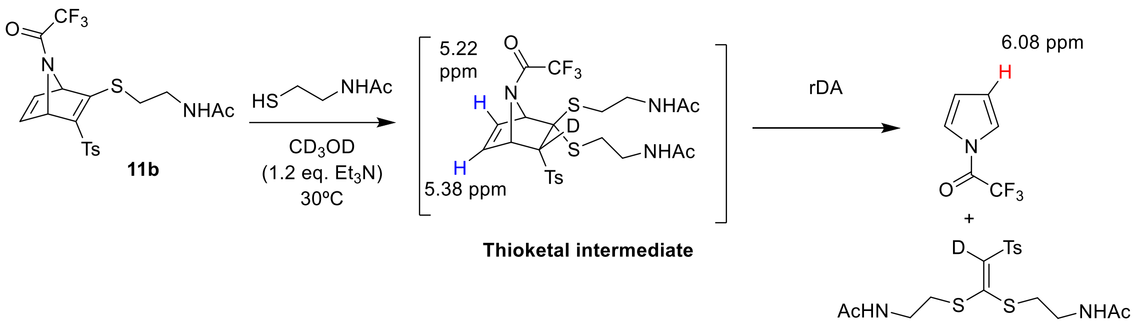

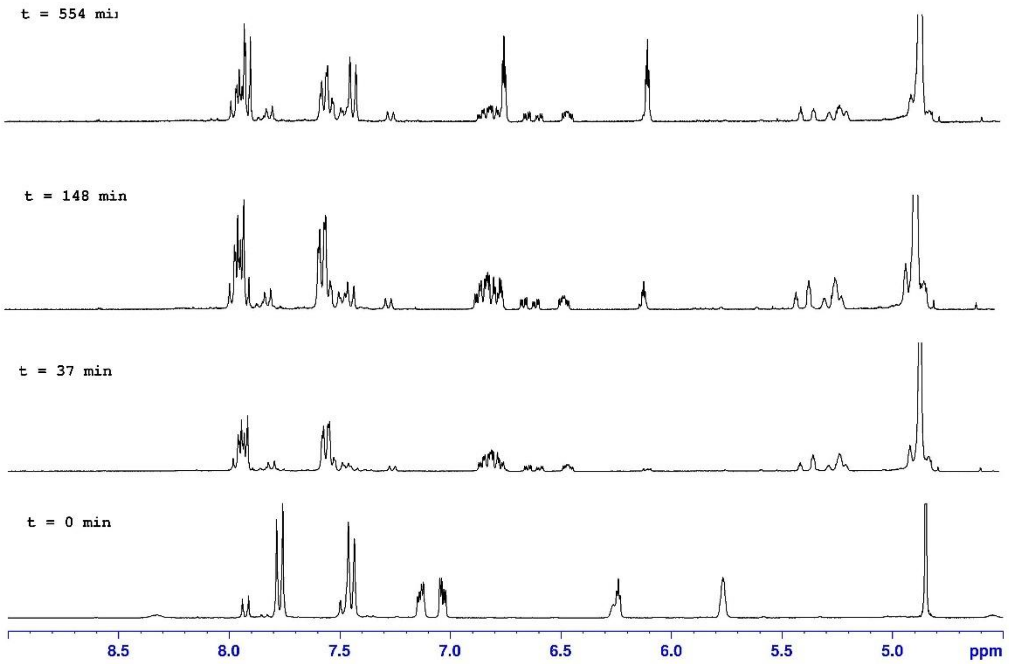

Figure S13. ^{1}H NMR (300 MHz, CD_3OD, 303 K) for the fragmentation of **11b**.

c) Starting thio-AND, thioketal intermediate and final products are observed in the ^{1}H NMR monitoring of the fragmentation

- ^{1}H NMR monitoring for the fragmentation of **14b**

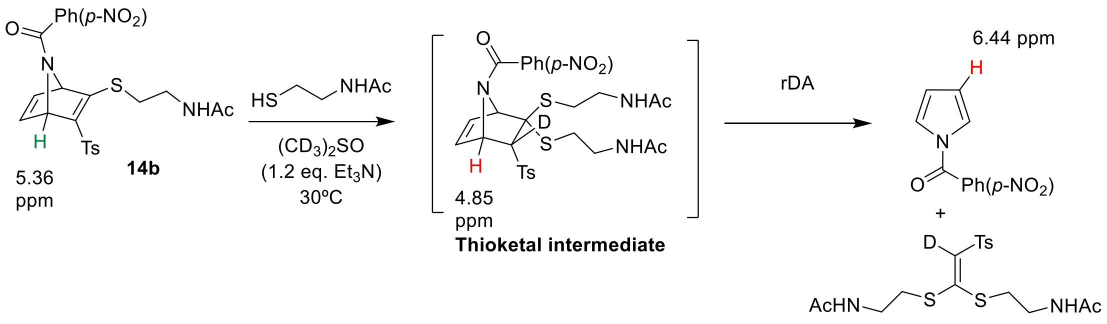

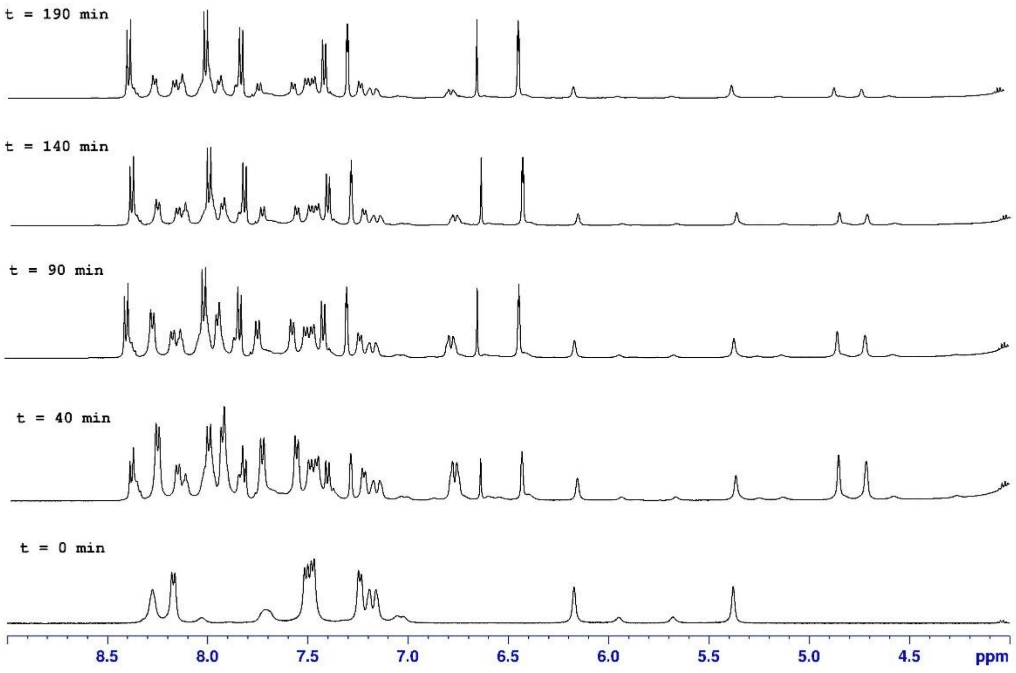

Figure S14. ^{1}H NMR (500 MHz, $(CD_3)_2SO$, 303 K) for the fragmentation of **14b**.

Plots for the % conversion *vs* time in the thiol-promoted fragmentation of ANDs.

- Fragmentation of compound **4b**

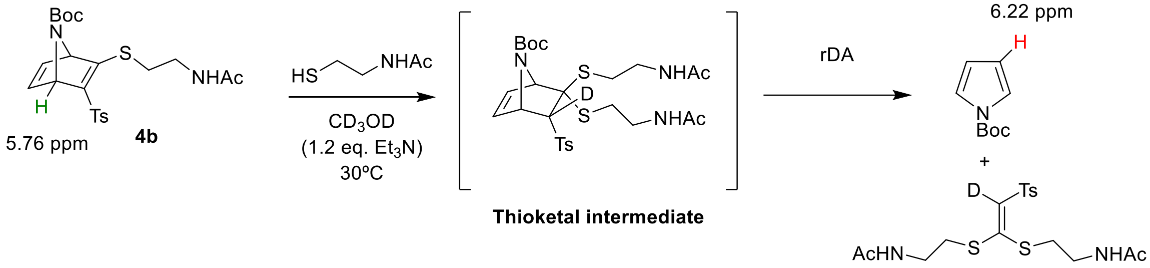

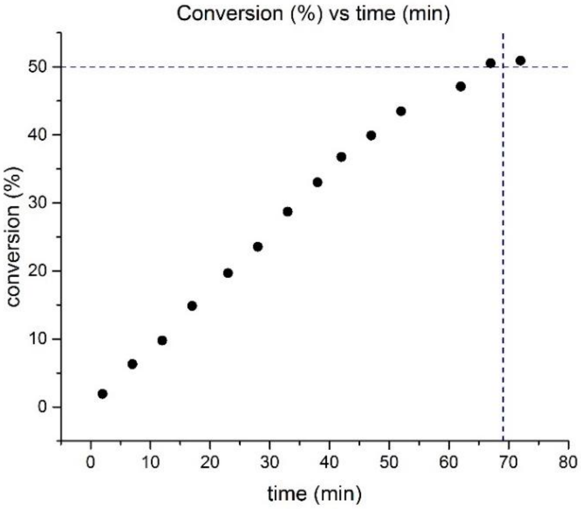

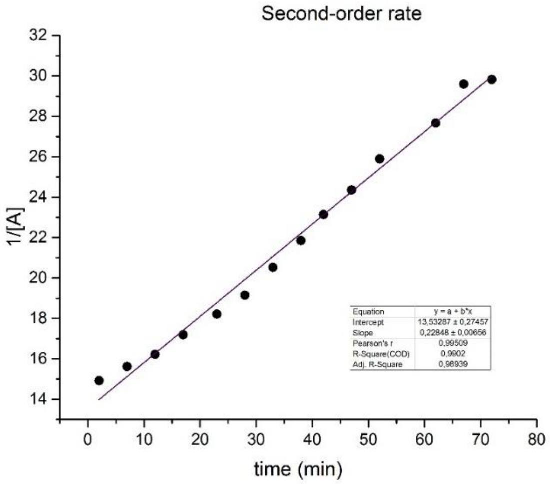

Figure S15. Plot for conversion (%) *vs* time (h) of the fragmentation of **4b**. Plot for second order rate. % Conversion was determined by ^{1}H NMR (300 MHz, CD_3OD, 300 K).

- Fragmentation of compound **5b**

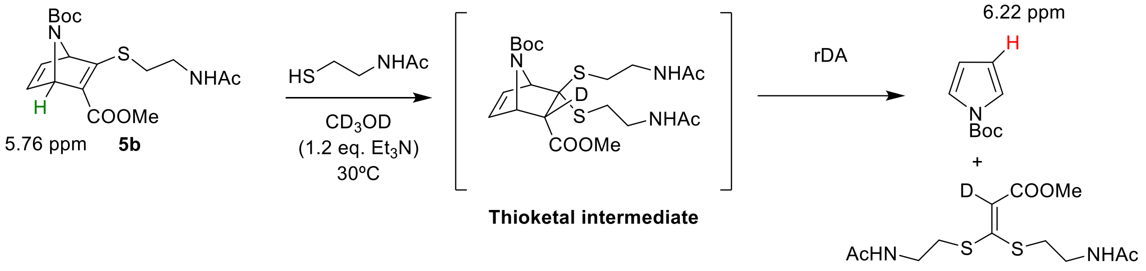

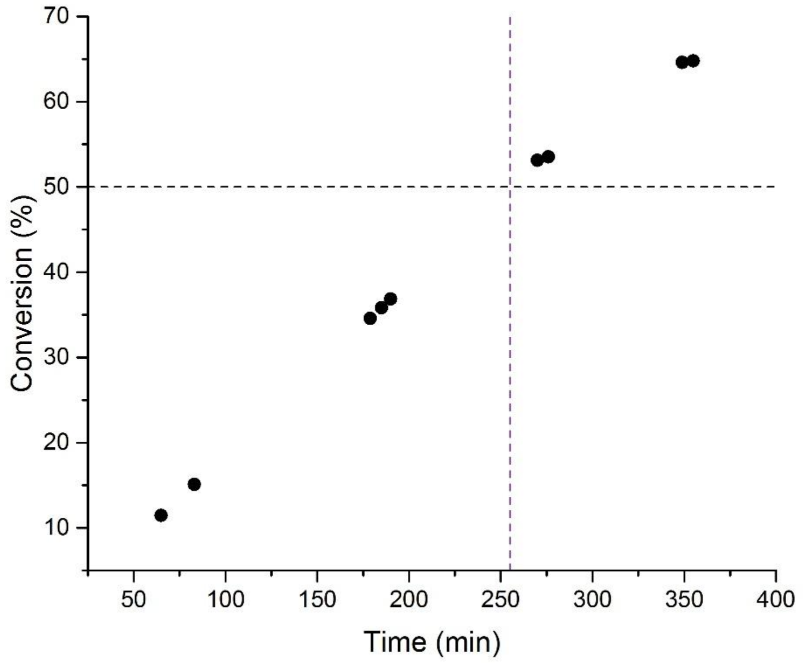

Figure S16. Plot for conversion (%) *vs* time (h) of the fragmentation of **5b**. % Conversion was determined by ^{1}H NMR (300 MHz, CD_3OD, 300 K).

- Fragmentation of compound **6b**

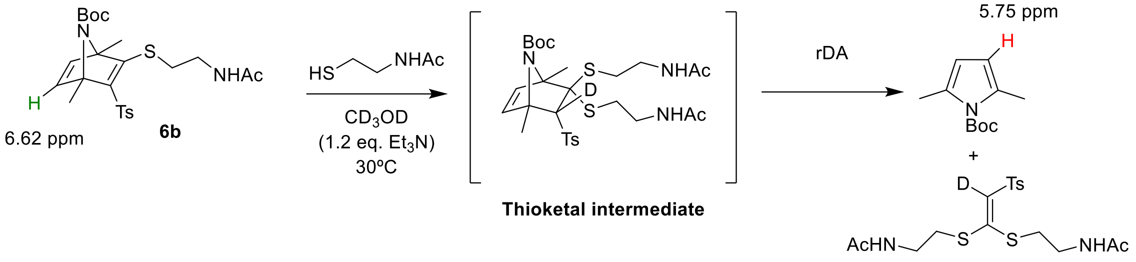

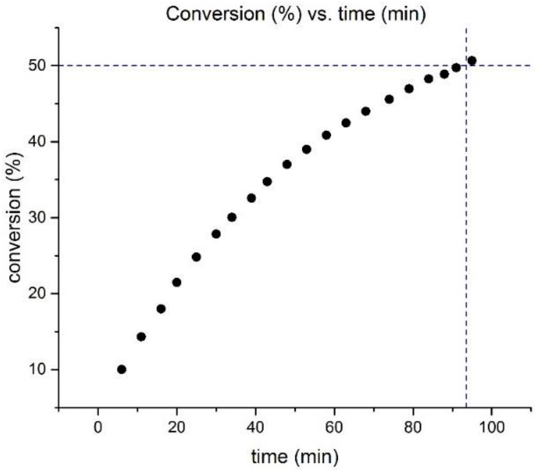

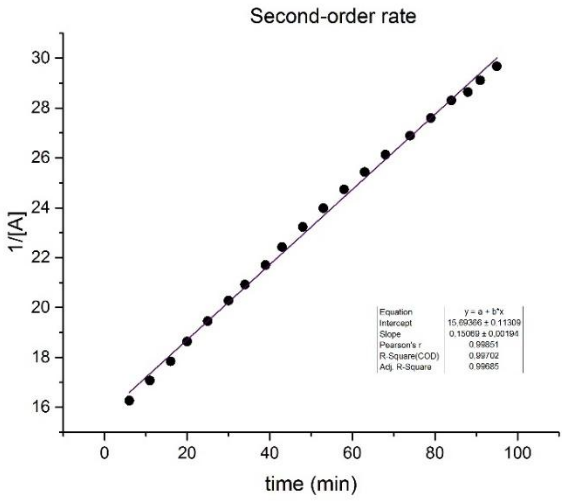

Figure S17. Plot for conversion (%) *vs* time (h) of the fragmentation of **6b**. Plot for second order rate. % Conversion was determined by ^{1}H NMR (300 MHz, CD_3OD, 300 K).

- Fragmentation of compound **7b**

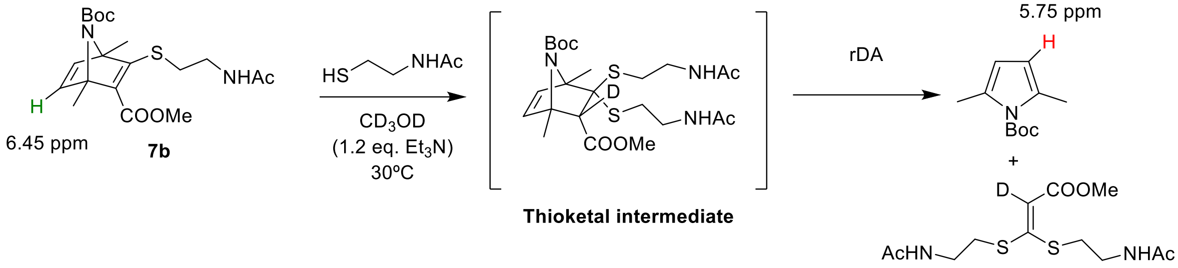

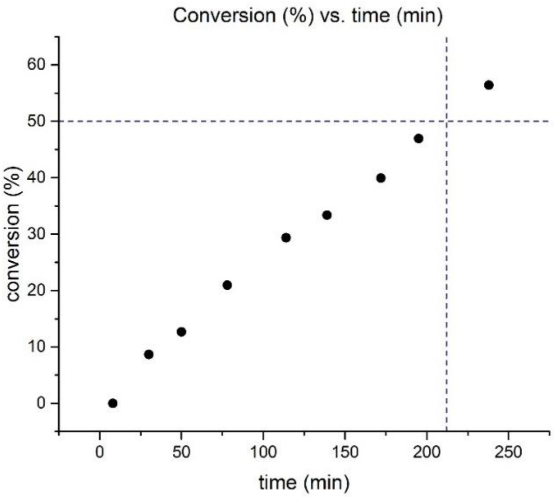

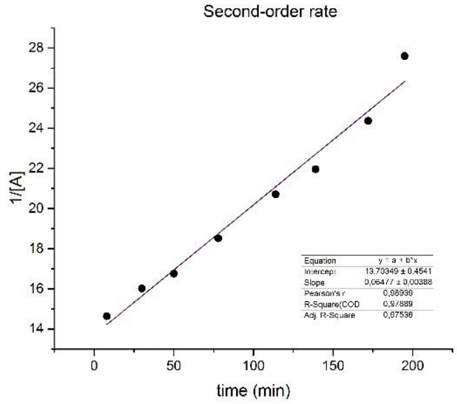

Figure S18. Plot for conversion (%) *vs* time (h) of the fragmentation of **7b**. Plot for second order rate. % Conversion was determined by ^{1}H NMR (300 MHz, CD_3OD, 300 K).

- Fragmentation of compound **8b**

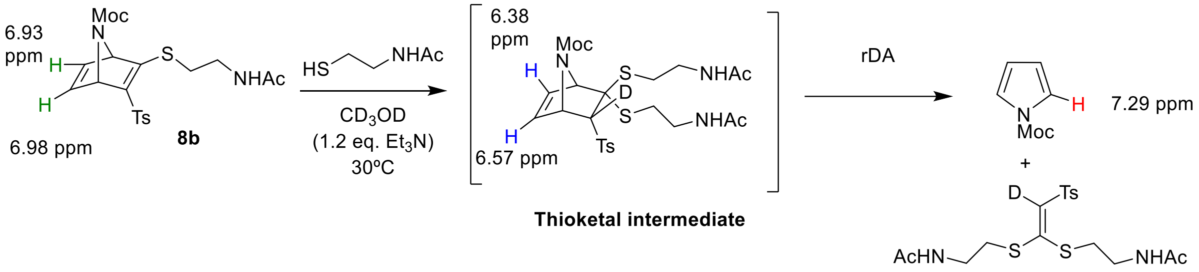

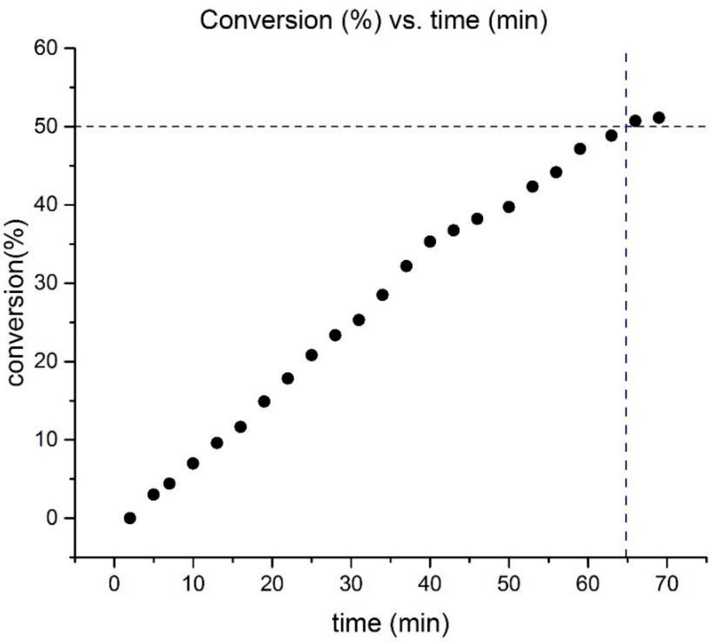

Figure S19. Plot for conversion (%) *vs* time (h) of the fragmentation of **8b**. % Conversion was determined by ^{1}H NMR (300 MHz, CD_3OD, 300 K).

- Fragmentation of compound **10b**

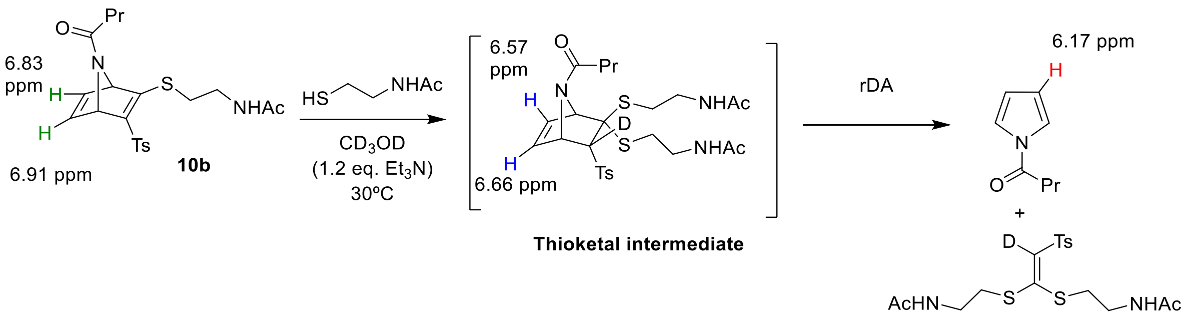

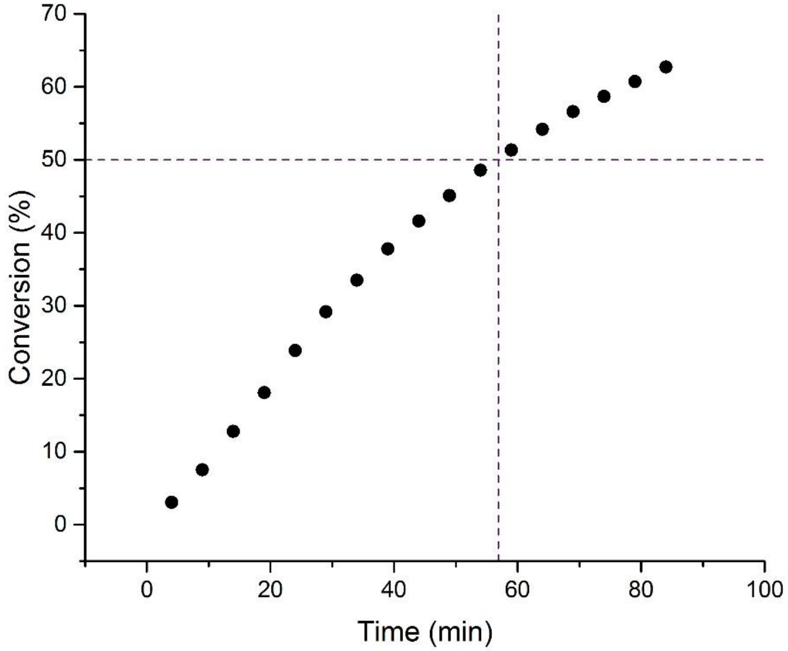

Figure S20. Plot for conversion (%) *vs* time (h) of the fragmentation of **10b**. % Conversion was determined by ^{1}H NMR (400 MHz, CD_3OD, 300 K).

- Fragmentation of compound **11b**

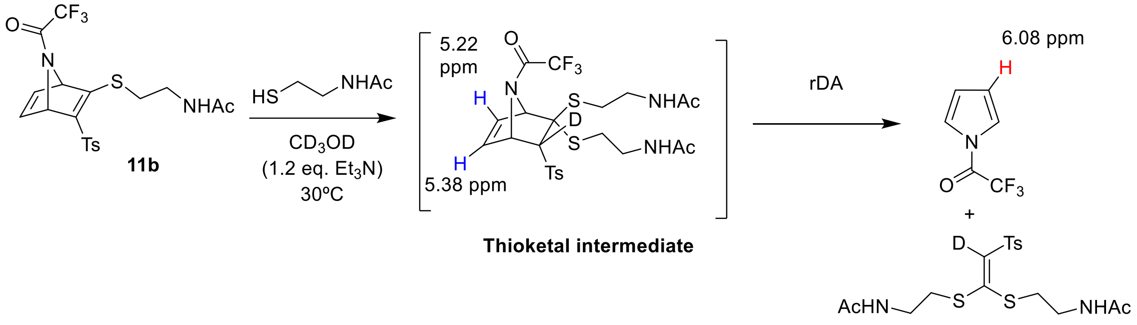

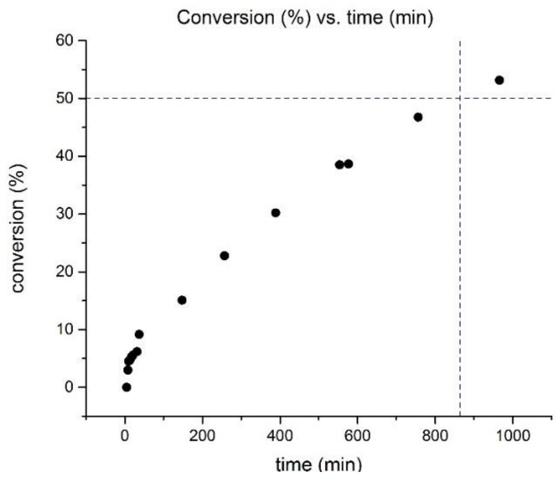

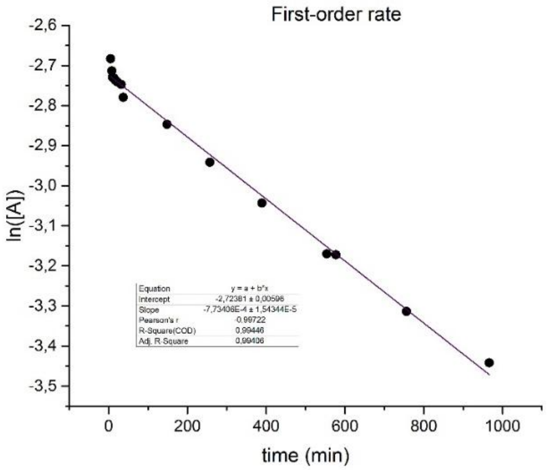

Figure S21. Plot for conversion (%) *vs* time (h) of the fragmentation of **11b**. Plot for first order rate. % Conversion was determined by ^{1}H NMR (300 MHz, CD_3OD, 300 K).

- Fragmentation of compound **12b** in CD_3OD

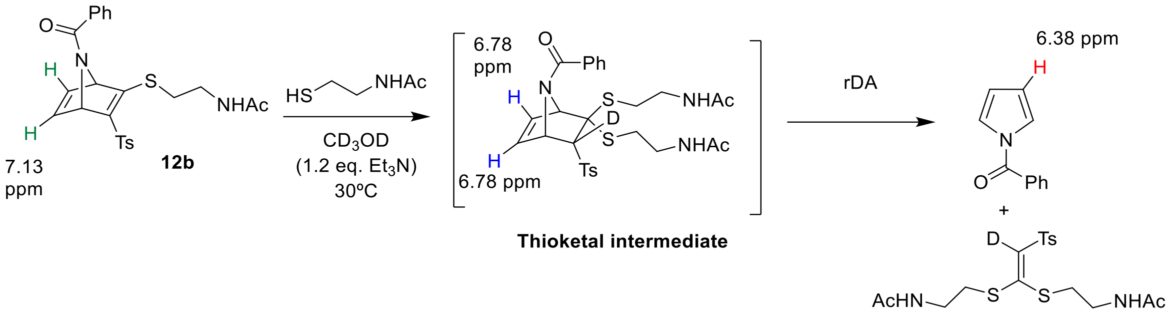

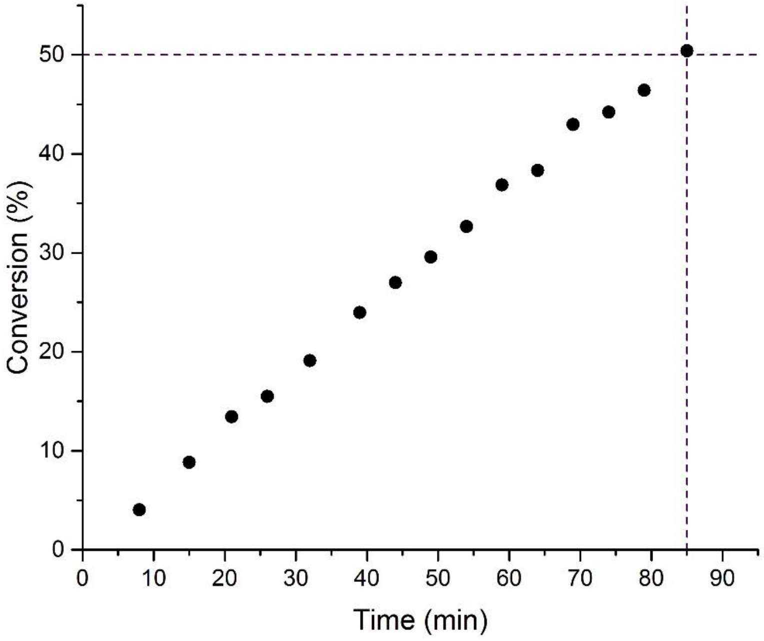

Figure S22. Plot for conversion (%) *vs* time (h) of the fragmentation of **12b**. % Conversion was determined by ^{1}H NMR (400 MHz, CD_3OD, 300 K).

- Fragmentation of compound **12b** in $(CD_3)_2SO$

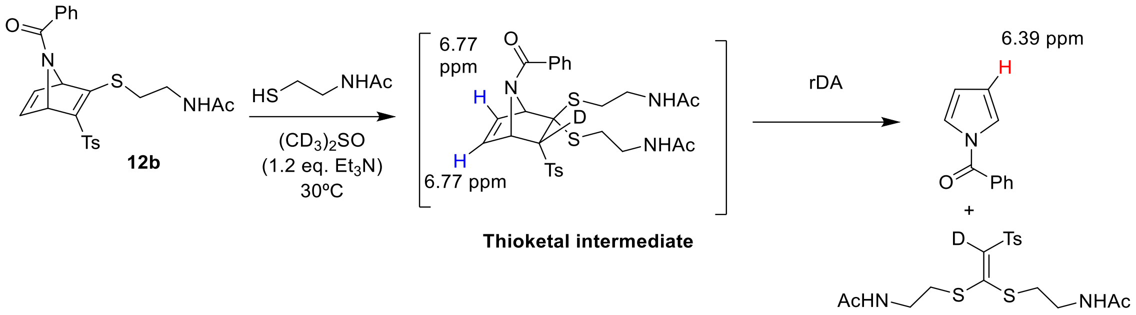

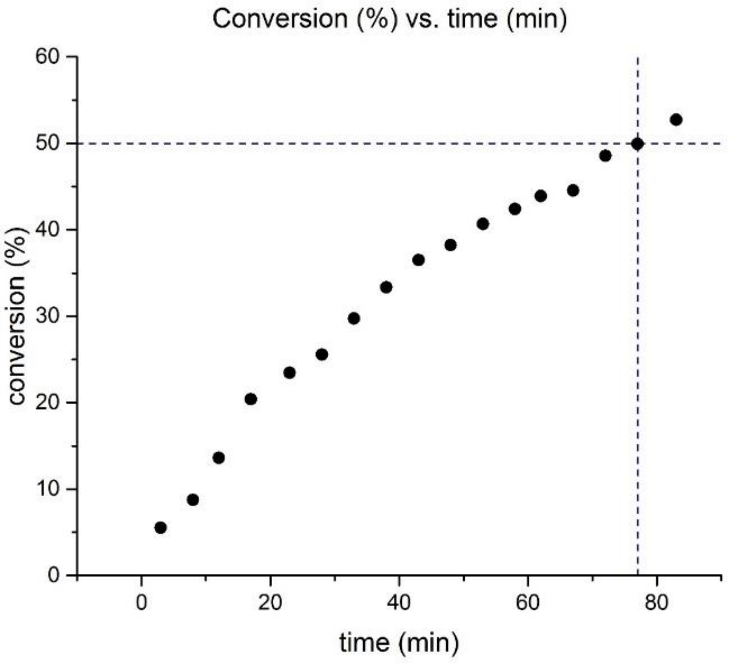

Figure S23. Plot for conversion (%) *vs* time (h) of the fragmentation of **12b**. % Conversion was determined by ^{1}H NMR (300 MHz, $(CD_3)_2SO$, 300 K).

- Fragmentation of compound **13b**

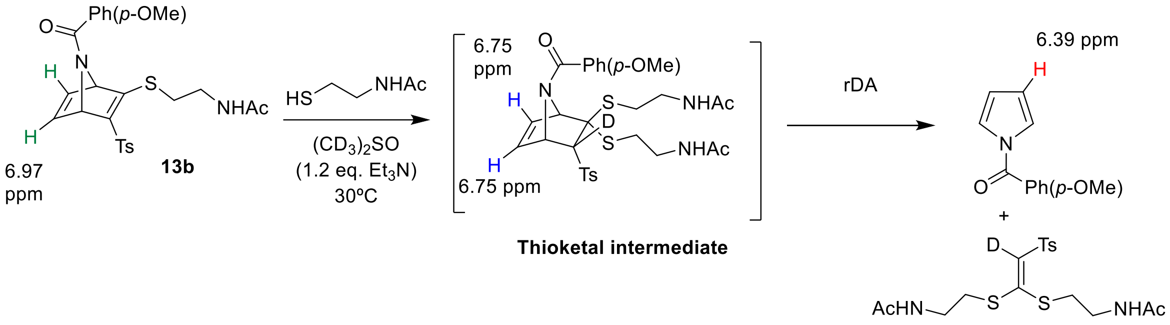

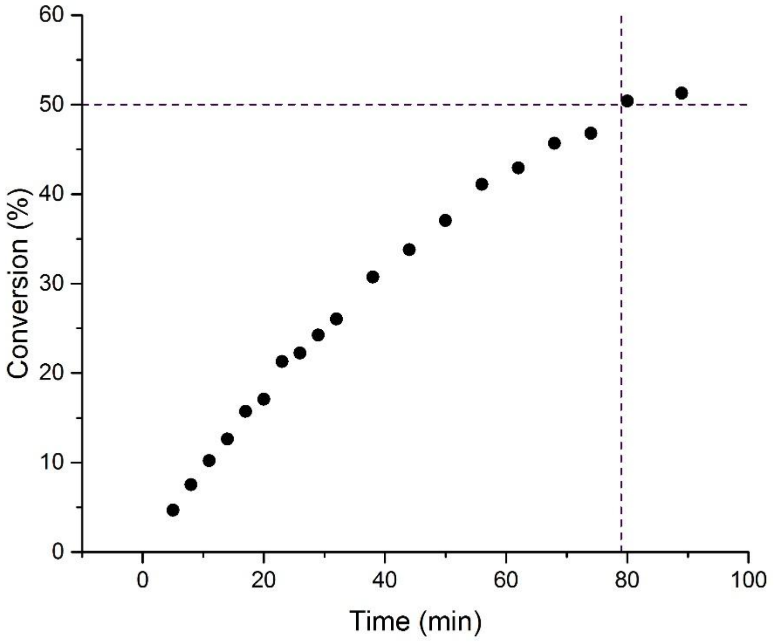

Figure S24. Plot for conversion (%) *vs* time (h) of the fragmentation of **13b**. % Conversion was determined by ^{1}H NMR (400 MHz, $(CD_3)_2SO$, 300 K).

- Fragmentation of compound **14b**

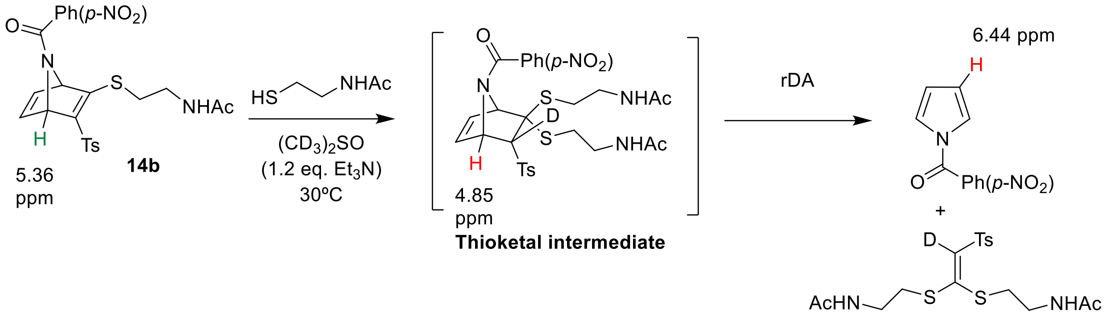

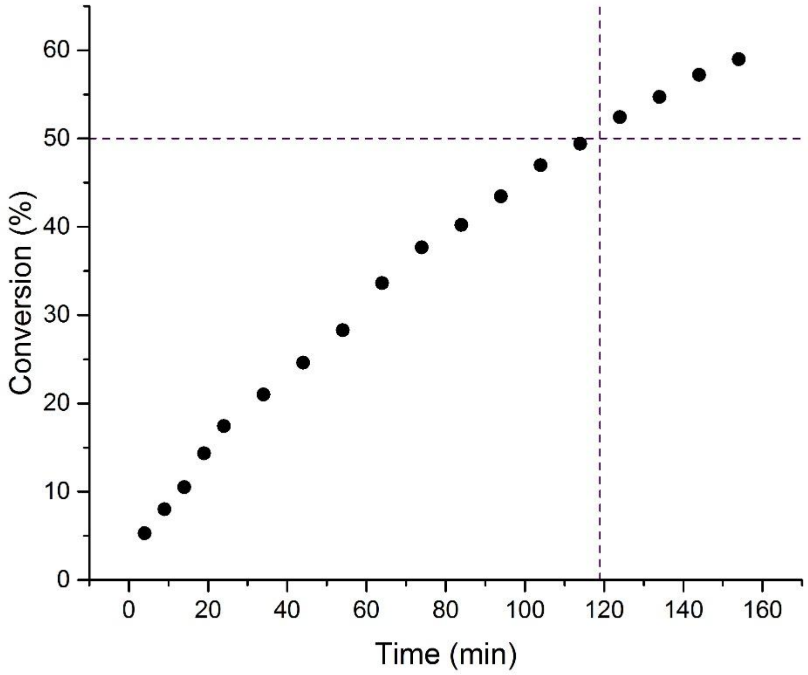

Figure S25. Plot for conversion (%) *vs* time (h) of the fragmentation of **14b**. % Conversion was determined by ^{1}H NMR (300 MHz, $(CD_3)_2SO$, 300 K).

- Fragmentation of compound **15b**

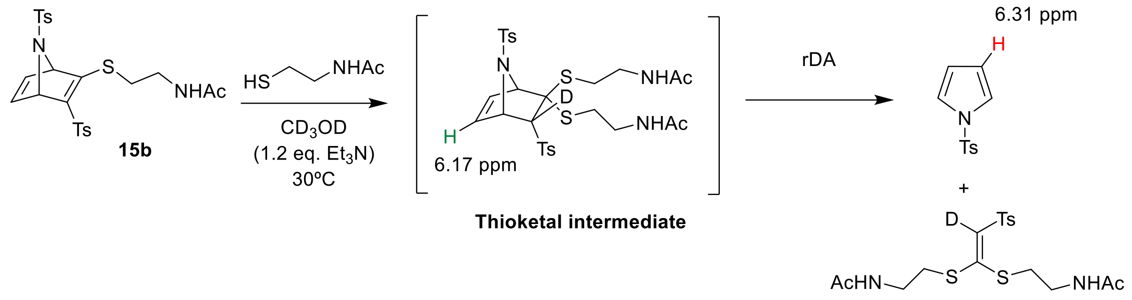

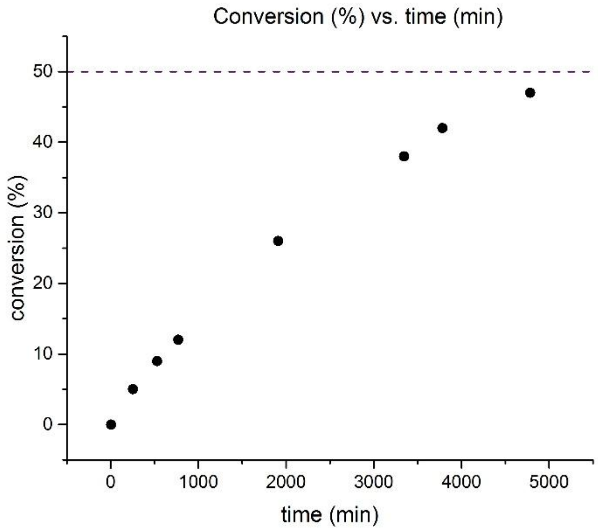

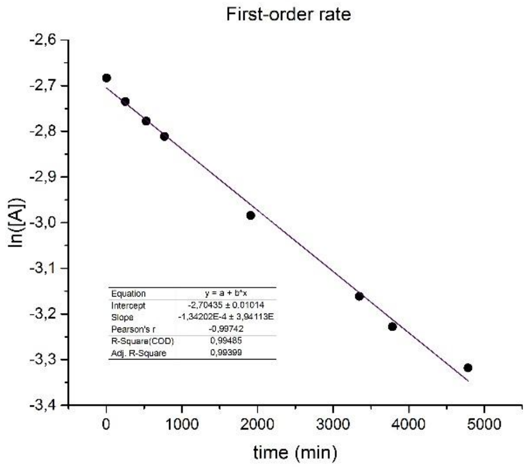

Figure S26. Plot for conversion (%) *vs* time (h) of the fragmentation of **15b**. Plot for first order rate. % Conversion was determined by ^{1}H NMR (300 MHz, CD_3OD, 300 K).

- Fragmentation of compound **16b**

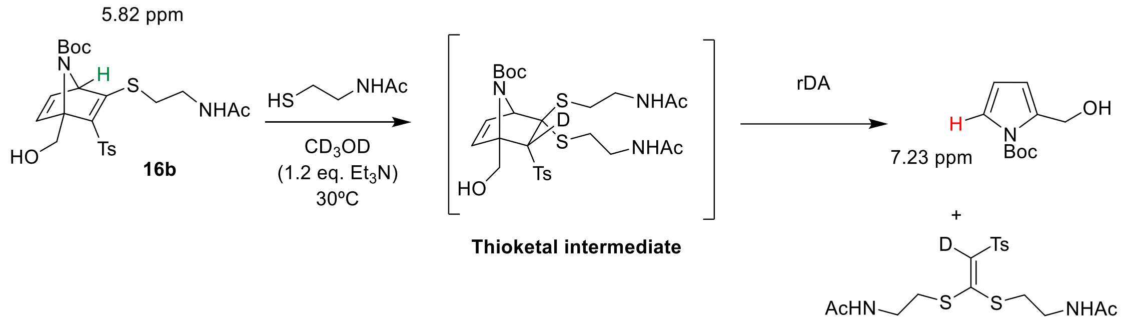

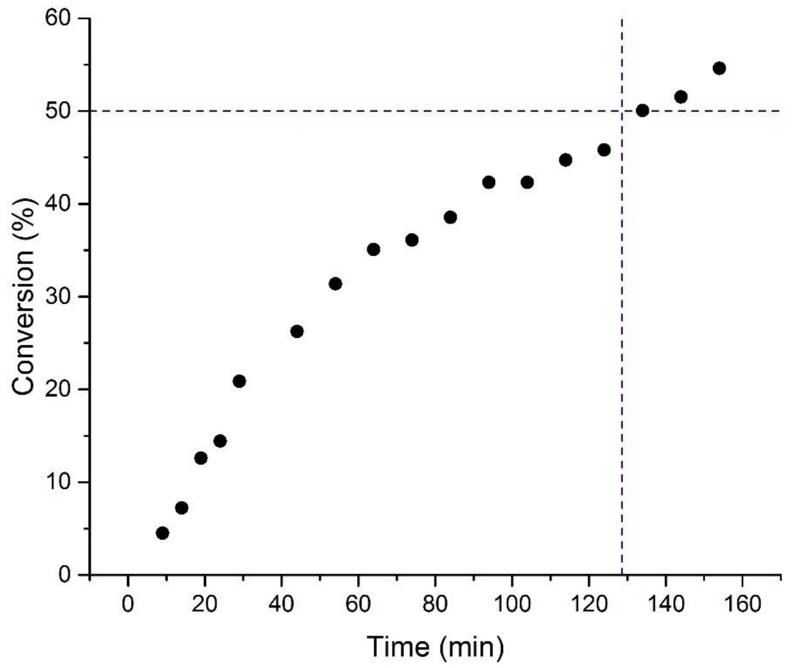

Figure S27. Plot for conversion (%) *vs* time (h) of the fragmentation of **16b**. % Conversion was determined by ^{1}H NMR (400 MHz, CD_3OD, 300 K).

- Fragmentation of compound **17b**

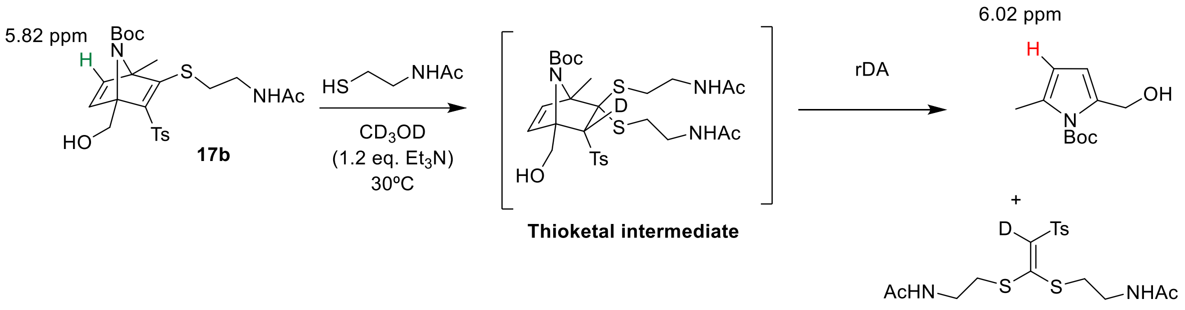

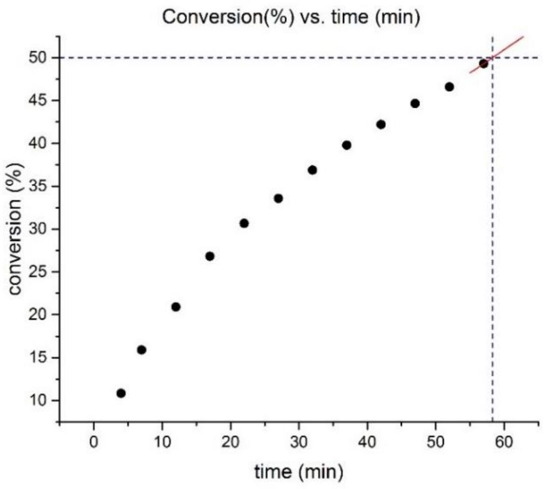

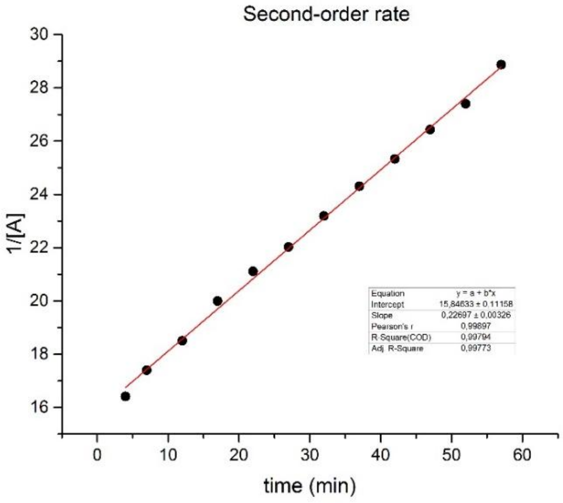

Figure S28. Plot for conversion (%) *vs* time (h) of the fragmentation of **17b**. Plot for second order rate. % Conversion was determined by ^{1}H NMR (300 MHz, CD_3OD, 300 K)

8. Synthesis of dansyl derivatives. Fluorescence emission plots.

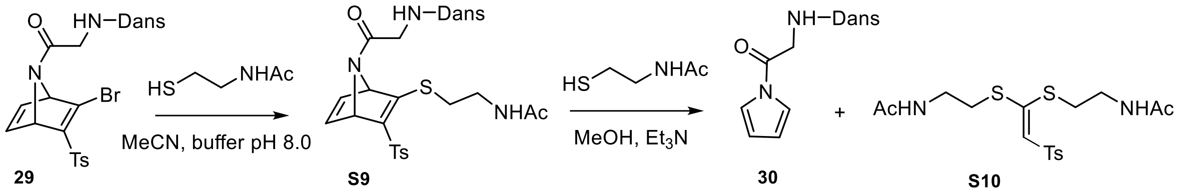

(rac) ***N*****-(2-((7-(((5-(Dimethylamino)naphthalen-1-yl)sulfonyl)glycyl)-3-tosyl-7-azabicyclo[2.2.1]hepta-2,5-dien-2-yl)thio)ethyl)acetamide (S9).** To a solution of **29** (82 mg, 0.13 mmol) in MeCN (2 mL), a solution of N-acetylcysteamine (14 mg, 0.12 mmol) in MeCN (1 mL) and phosphate buffer solution (pH 8.0, 50 mM, 2 mL), were added simultaneously and the mixture was stirred at r.t. for 30 min. Then, solvents were evaporated, and the residue was dissolved in AcOEt and washed with water. The organic phase was dried, filtered and concentrated. Purification by silica gel column chromatography (EtOAc:Cy 10:1→ EtOAc) afforded **S9** (40 mg, 0.061 mmol, 51%, pale yellow solid). ^{1}H NMR (300 MHz, CD_3OD, 298 K, δ ppm, mixture of rotamers): δ 8.57 (d, 1H, *J* = 8.4 Hz, Ar-H), 8.33 (d, 1H, *J* = 8.6 Hz, Ar-H), 8.15 (dd, 1H, *J* = 7.5 Hz, 1.1 Hz, Ar-H), 7.69 (d, 1H, *J* = 8.3 Hz, Ar-H), 7.63-7.54 (m, 2H, Ar-H), 7.41-7.34 (m, 2H, Ar-H), 7.28 (d, 1H, *J* = 7.6 Hz, Ar-H), 6.96-6.67 (m, 2H, H-5, H-6), 5.85 (s, 1H, H-1 or H-4), 5.54 (s, 1H, H-1 or H-4), 3.81-3.51 (m, 2H, H-2'), 3.27-3.02 (m, 2H, H-1'), 2.89 (s, 3H, $N(CH_3)_2$), 2.38 (s, 3H, CH_3 of Ts), 1.94 (s, 3H, $COCH_3$). $^{13}C\{^1H\}$ NMR (75 MHz, CD_3OD, 298 K, δ ppm, mixture of rotamers): δ 173.6, 170.3, 167.2, 164.4, 153.2, 146.6, 144.5, 143.7, 140.4, 139.0, 137.9, 136.8, 131.3, 131.2, 131.1, 1230.1, 129.9, 129.1, 128.2, 124.4, 120.7, 116.7, 69.7, 56.2, 45.8, 41.3, 32.4, 22.5, 22.6. HRESIMS m/z: found, 655.1706; calcd. for $C_{31}H_{35}N_4O_6S_3$ $[M+H]^+$, 655.1732.

5-(Dimethylamino)-N-(2-oxo-2-(1H-pyrrol-1-yl)ethyl)naphthalene-1-sulfonamide (30). To a solution of **S9** (60 mg, 0.092 mmol) in MeOH (1 mL), *N*-acetylcysteamine (17 mg, 0.14 mmol) and Et_3N (20 µL, 0.14 mmol) on MeOH (1 mL) were added, and the mixture of reaction was stirred a r.t. for 3 h. Then, the solvent was evaporated and the crude was purified by column chromatography on silica gel (EtOAc:Cy 10:1 → EtOAc:Acetone 10:1) to afford **30** (17 mg, 0.048 mmol, 52%, yellow solid) and **S10**[10]. Data for **30**: ^{1}H NMR (300 MHz, $CDCl_3$, 298 K, δ ppm): δ 8.55 (ap. d, 1H, *J* = 8.9 Hz, Ar-H), 8.33 (ap. d, 1H, *J* = 8.4 Hz, Ar-H), 8.26 (dd, 1H, *J* = 7.7, 1.8 Hz, Ar-H), 7.66-7.55 (m, 1H, Ar-H), 7.50 (dd, 1H, *J* = 9.5, 6.6 Hz, Ar-H), 7.20 (d, 1H, *J* = 7.6 Hz, Ar-H), 7.11 (br. s, 2H, H-2, H-5), 6.29-6.22 (m, 2H, H-3, H-4), 5.69 (t, 1H, *J* = 4.2 Hz, NH), 4.28 (d, 2H, *J* = 4.9 Hz, CH_2), 2.88 (s, 6H, $N(CH_3)_2$). $^{13}C\{^1H\}$ NMR (75 MHz, $CDCl_3$, 298 K, δ ppm): δ 162.1 (C=O), 152.1, 133.7 (C_q Ar), 131.07 (Ar-H), 130.0 (C_q Ar),

129.66, 129.62 (Ar-H), 128.8 (C_q Ar), 123.0, 118.6, 118.5 (Ar-H), 115.5 (C-2, C-5), 114.3 (C-3, C-4), 45.4 ($N(CH_3)_2$), 45.0 (CH_2). HRESIMS m/z: found, 358.1217; calcd. for $C_{18}H_{20}N_3O_3S$ $[M+H]^+$, 358.1220.

Fluorescence emission plots

Determination of emission curves: Stock solutions (10 mM in DMSO) and subsequent dilutions (200 µM in DMSO) of each compound were prepared. 50 µL of each compound (200 µM) was mixed with 50 µL of buffer (NaPi, 10 mM, pH 7.4 and 8.2) to achieve a final concentration of 100 µM in DMSO/Buffer 1:1. Emission spectrum of each solution was recorded in a Varioskan Lux ThermoFisher Scientific at 37 ºC, at a fixed excitation wavelength (334 nm). Each spectrum was recorded from 400 nm to 750 nm. Each spectrum was measured in triplicate (all these measurements were carried out in a greiner Black 96 well plate).

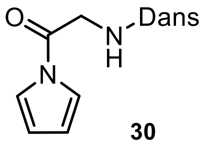

30

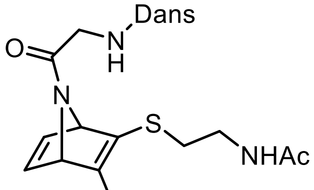

S9

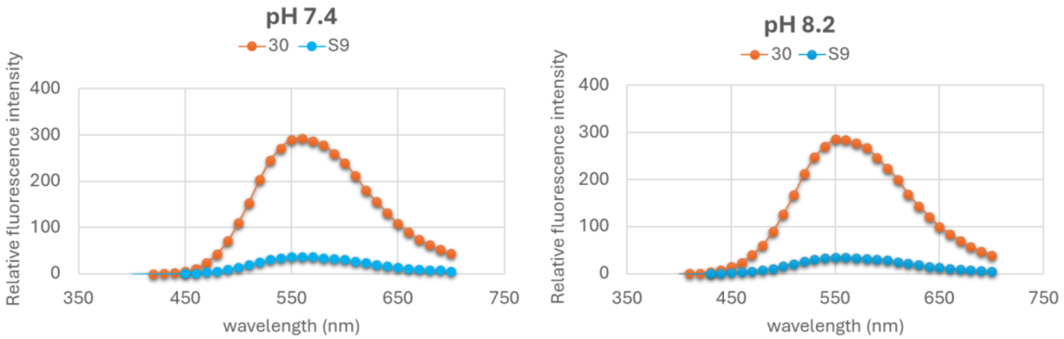

Figure S29. Fluorescence emission plots of compounds **30** and **S9** at pH 7.4 (left) and pH 8.2 (right).

Table S3. Comparative fluorescence **S9** *vs* **30**. Maximum fluorescence determined from the emission curves. (λ_{ex}= 334 nm, λ_{em}= 550 nm)

	**S9**	**30**
**pH 6.4**	53.2	304.8
**pH 7.4**	56.9	292.8
**pH 8.2**	50.8	289.5

9. Protein bioconjugation

General LCMS protocol

LC–MS analysis of protein samples was carried out using a Waters SQD2 in combination with an Acquity UPLC system with an Acquity UPLC BEH300 C4 column (130 Å1.7 μm, 2.1 × 50 mm). The SQD2 mass spectrometer mobile phase consisted of solvent A (99.9% water with 0.1% formic acid), solvent B (99.9% ACN with 0.1% formic acid) and the following gradient was programmed: 5% to 72% B in 6 min, then 72% B for 1.5 min followed by a gradient from 72% to 5% B over 0.25 mins and finally, 95% A for 1.25 mins. The capillary voltage of the electrospray source for the Waters SQD2 mass spectrometer was 3.0 kV with a cone voltage of 30 V and the desolvation gas used was nitrogen, with a flow rate of 800 L h^{-1}. The ion series was obtained through integration of the major peaks of the chromatogram. Following this, the total mass spectra were reconstructed using the MaxEnt1 algorithm on the MassLynx software (v. 4.1), according to manufacturers guidelines.

General protocol for SDS-NuPAGE gel electrophoresis

SDS-NuPAGE gel electrophoresis was carried out using an XCell Sure Lock™ Mini-Cell electrophoresis system from ThermoFisher Scientific. Protein molecular weight were approximated by comparison to a protein marker (SeeBlue™ Pre-stained Protein Standard 3-200KDa from Invitrogen™). Samples were prepared by mixing 11μL of each time point aliquot with 4μL of NuPAGE LDS sample buffer. Sample preparation was done without boiling to maintain integrity of the conjugate. Resulting solutions were then loaded to pre-cast NuPAGE™ Bis-Tris mini protein gel with 4-12% gradient polyacrylamide concentration and subject to electrophoresis at 200V with 1X MES running buffer. The SDS–PAGE gel was visualised by blue Trans UV illumination source (Pro-Q Emerald 300) prior to staining and subsequently Instant Blue™ coomassie stain using a ChemiDoc Imaging System (Bio-Rad)

Protein Expression PD-L1 sdAb

Periplasmic expression of the anti-PD-L1 sdAb was achieved from BL21(DE3) *E. coli* strain using previously established protocols. (Reference https://doi.org/10.1021/jacs.4c03721).

Protein sequence:

MAQVQLVETGGGLVQPGGSLRLSCTASGFTFSMHAMTWYRQAPGKQRELVAVITSHGDRANYTDSVRGRFTIS

RDNTKNMVYLQMNSLKPEDTAVYYCNVPRYDSWGQGTQVTVSSSPSTPPTPSPSTPPCGENLYFQGLEHHHHH

H

Calculated molecular weight: 16239 Da

Theoretical extinction coefficient: 21555 $M^{-1}cm^{-1}$

Characterization of Bioconjugate A

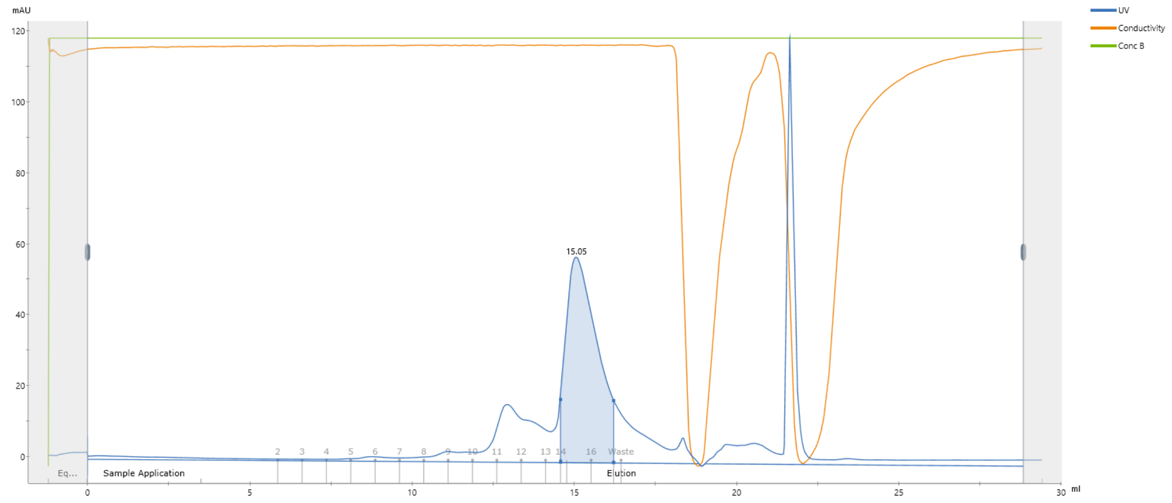

Figure S30. Size Exclusion Chromatogram for crude **bioconjugate A**. Collected peak at 15.05 minutes corresponds to the pure conjugate.

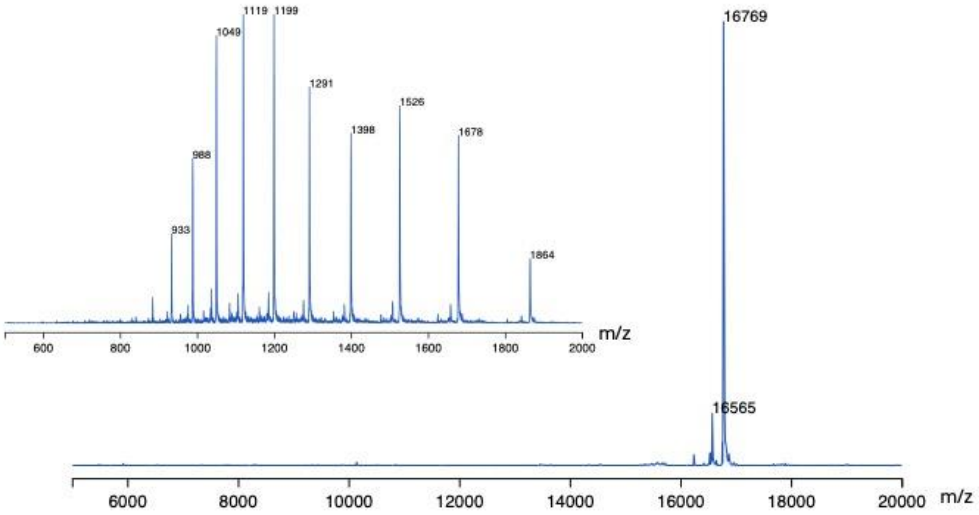

Figure S31. Combined ion series and deconvoluted mass spectrum of Bioconjugate **A**.

GSH stability assay

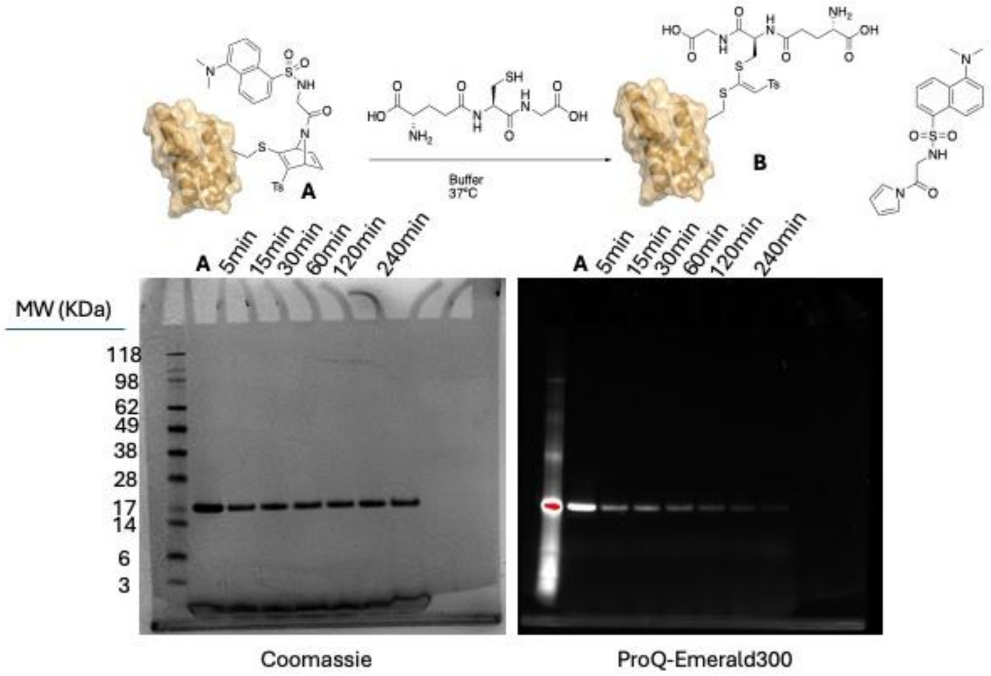

Figure S32. Biconjugate **A** treated with GSH in PBS at 37 ºC. Aliquots were taken at various time points during incubation and analysed by SDS-NUPAGE gel. Left: coomassie stained gel and Right: ProQ-Emerald channel showing residual fluorescence of bioconjugate **A**.

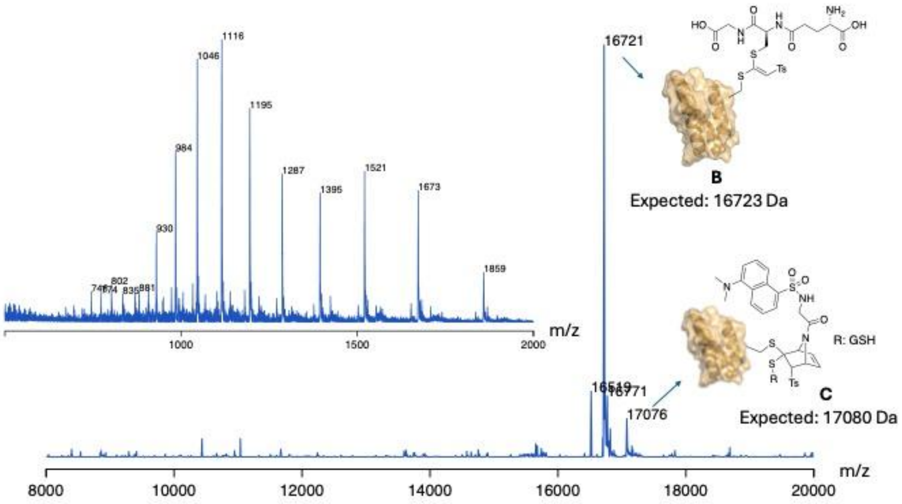

Figure S33. Combined ion series and deconvoluted mass spectrum of the reaction between GSH and bioconjugate **A** after 240 min of incubation at 37 °C.

Plasma and HSA stability studies

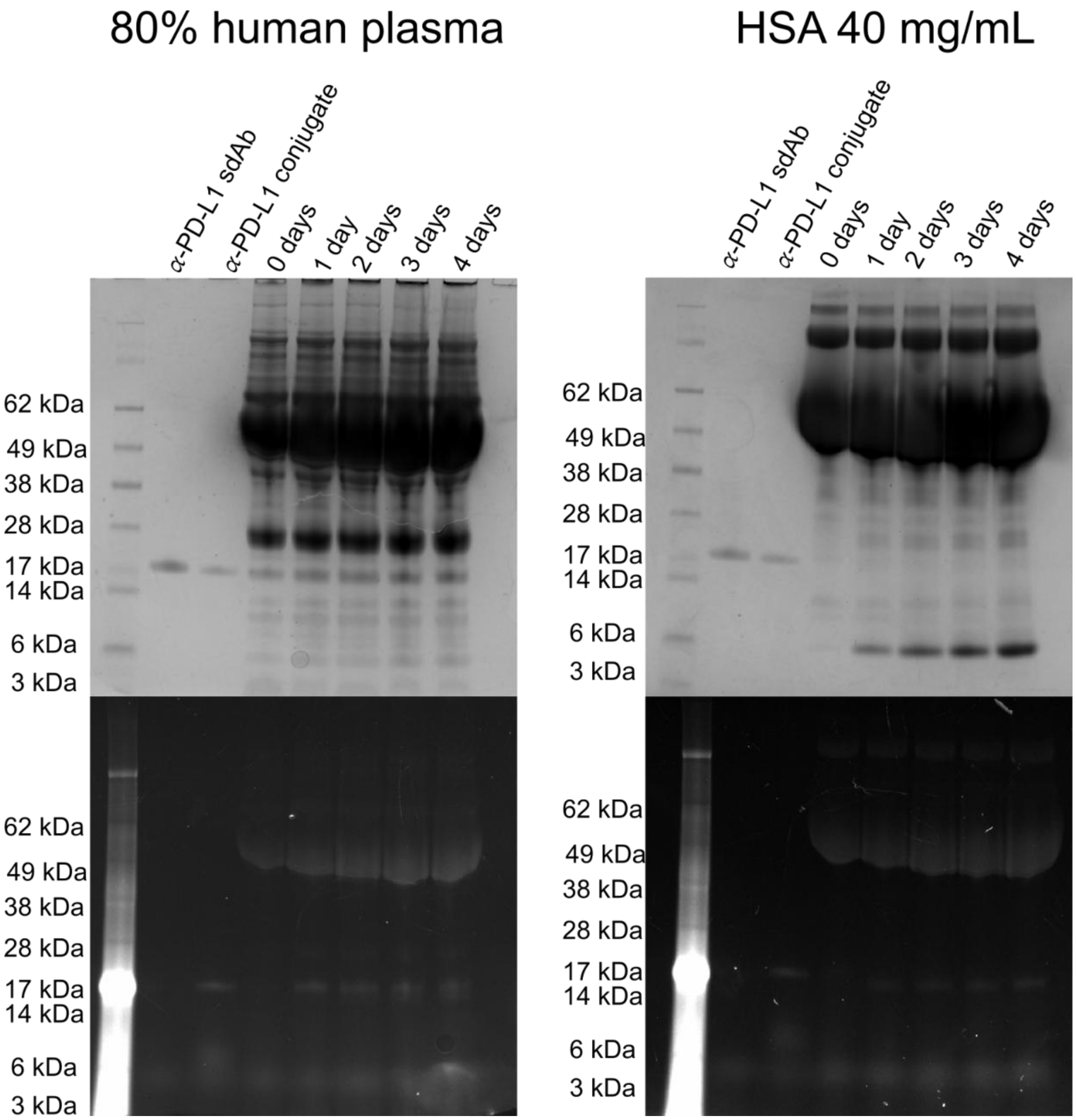

Figure S34. Bioconjugate **A** stability in 80 % human plasma (left) and HSA 40 mg/mL (right). Aliquots were taken at different times and analysed by SDS NUPAGE. Top: coomassie; bottom: ProQ-Emerald channel showing residual fluorescence of bioconjugate **A**.

10. ^{1}H-NMR and ^{13}C-NMR for new compounds

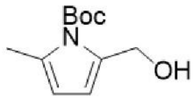

S5

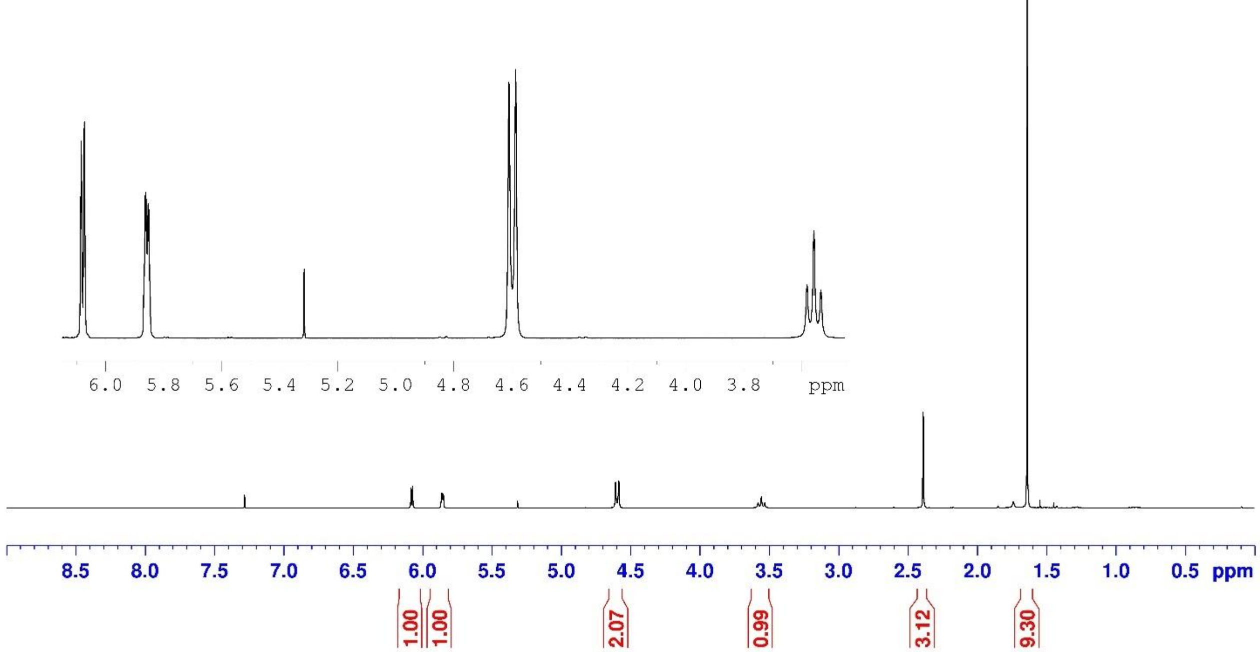

^{1}H-NMR ($CDCl_3$, 300 MHz) of compound **S5**

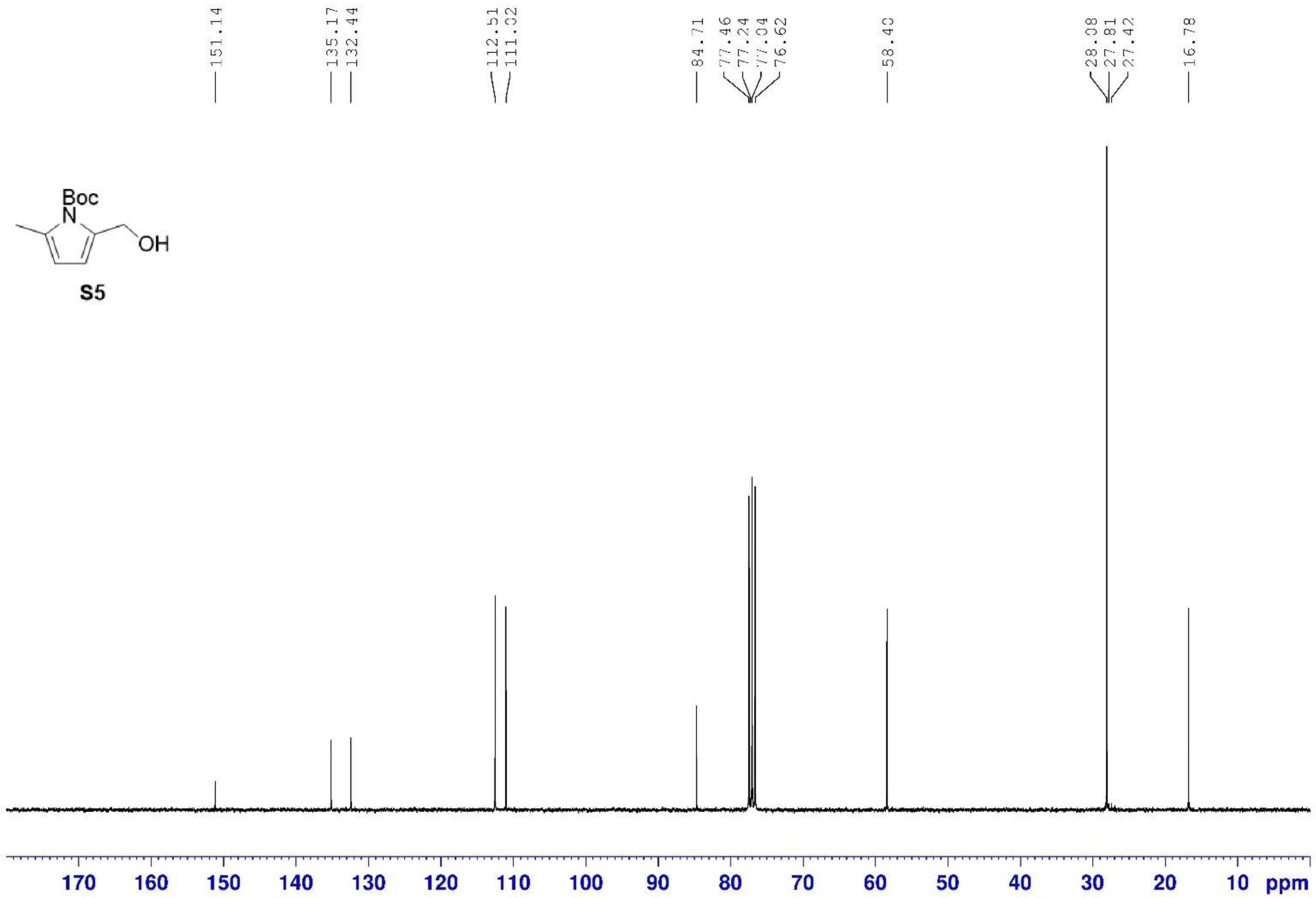

^{13}C-NMR ($CDCl_3$, 75 MHz) of compound **S5**

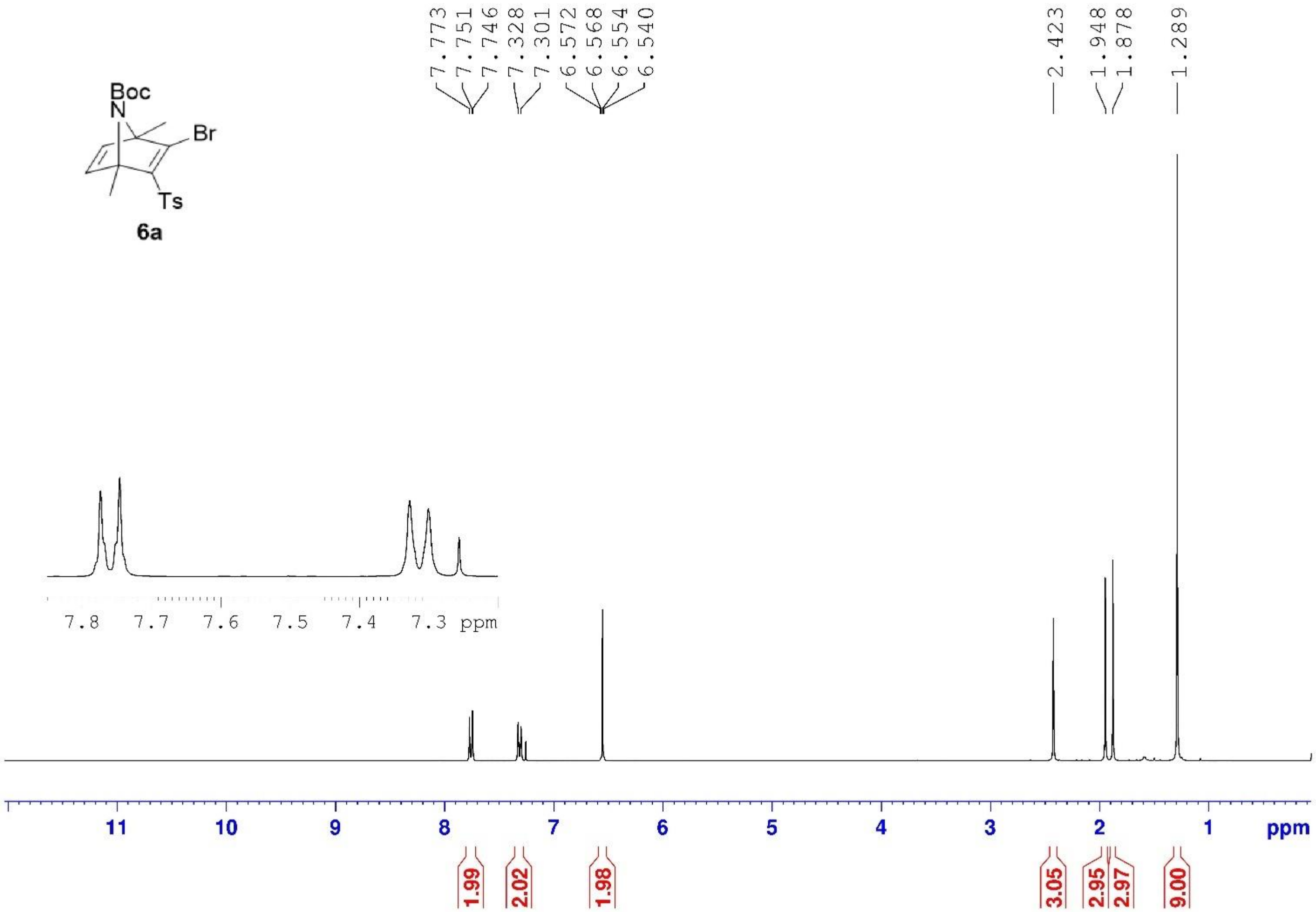

^1H-NMR ($CDCl_3$, 300 MHz) of compound **6a**

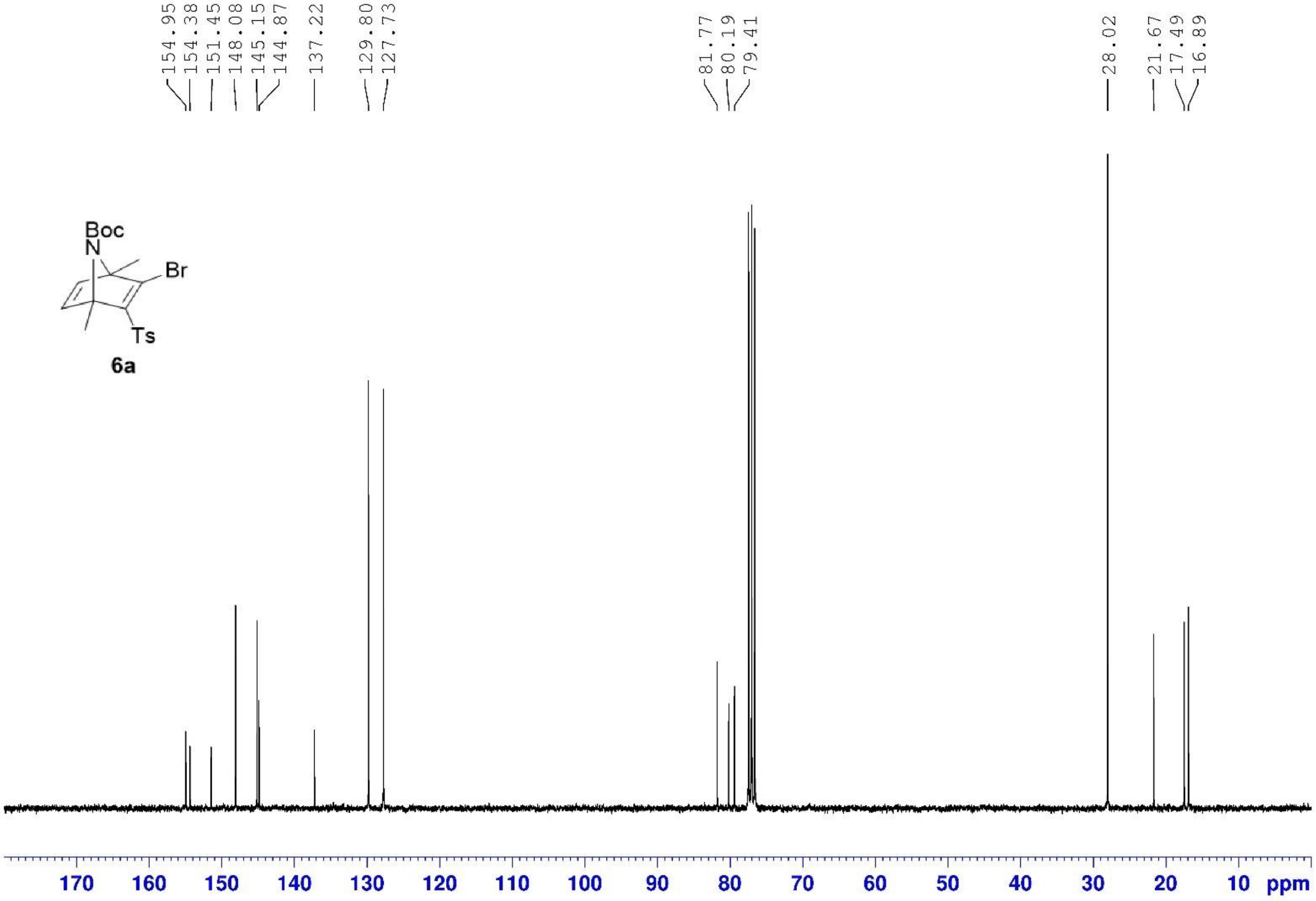

^{13}C-NMR ($CDCl_3$, 75 MHz) of compound **6a**

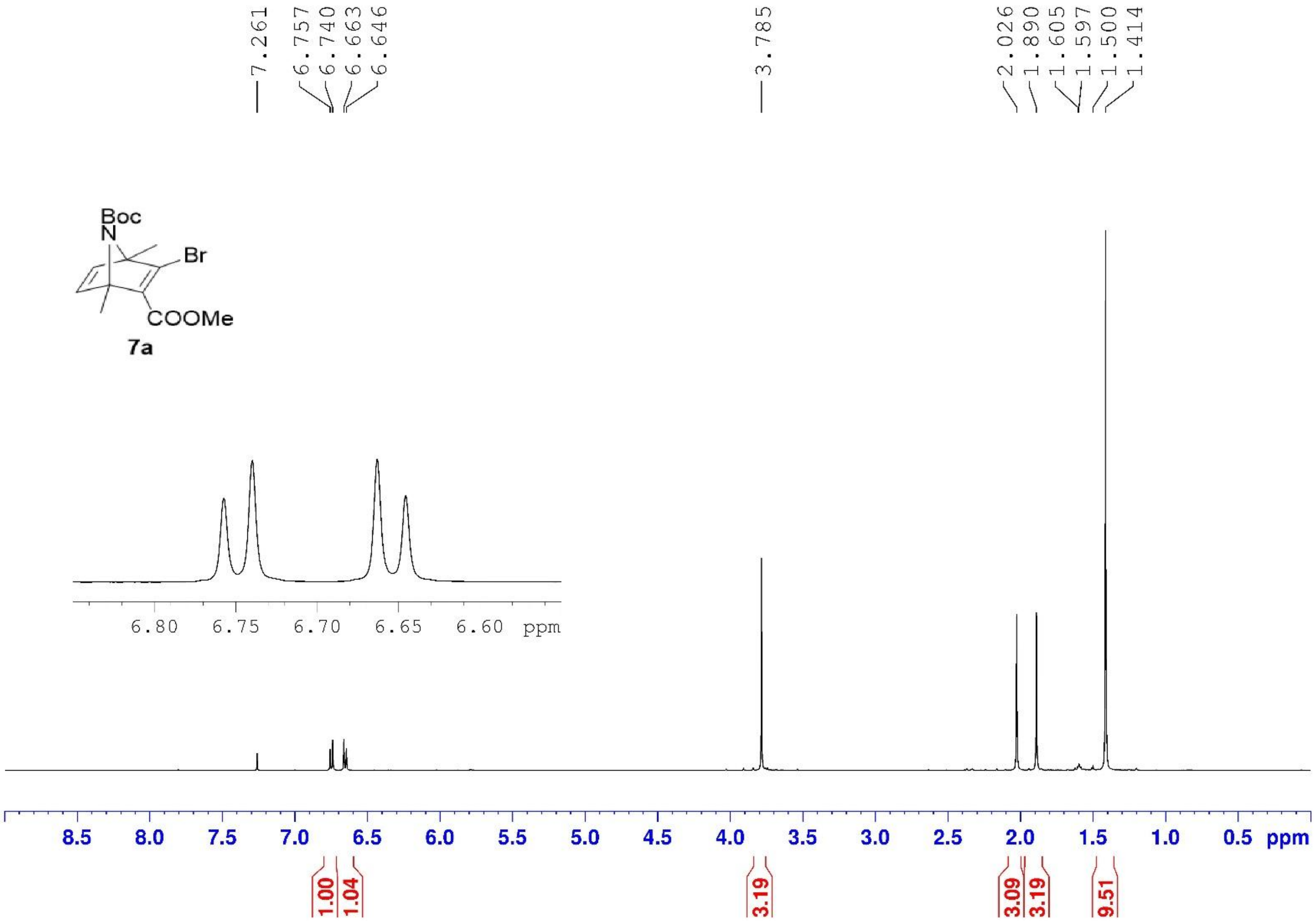

^{1}H-NMR ($CDCl_3$, 300 MHz) of compound **7a**

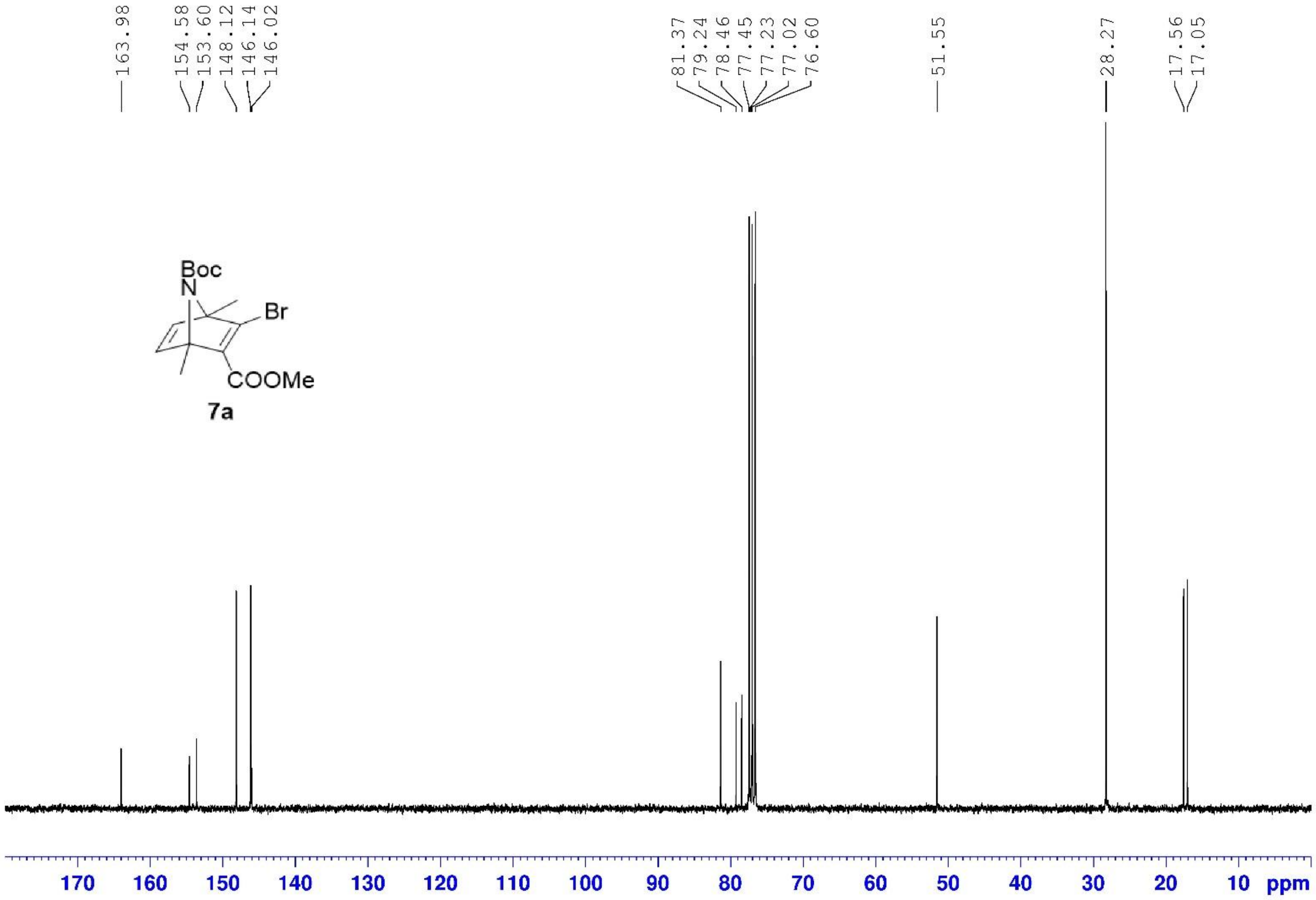

^{13}C-NMR ($CDCl_3$, 75 MHz) of compound **7a**

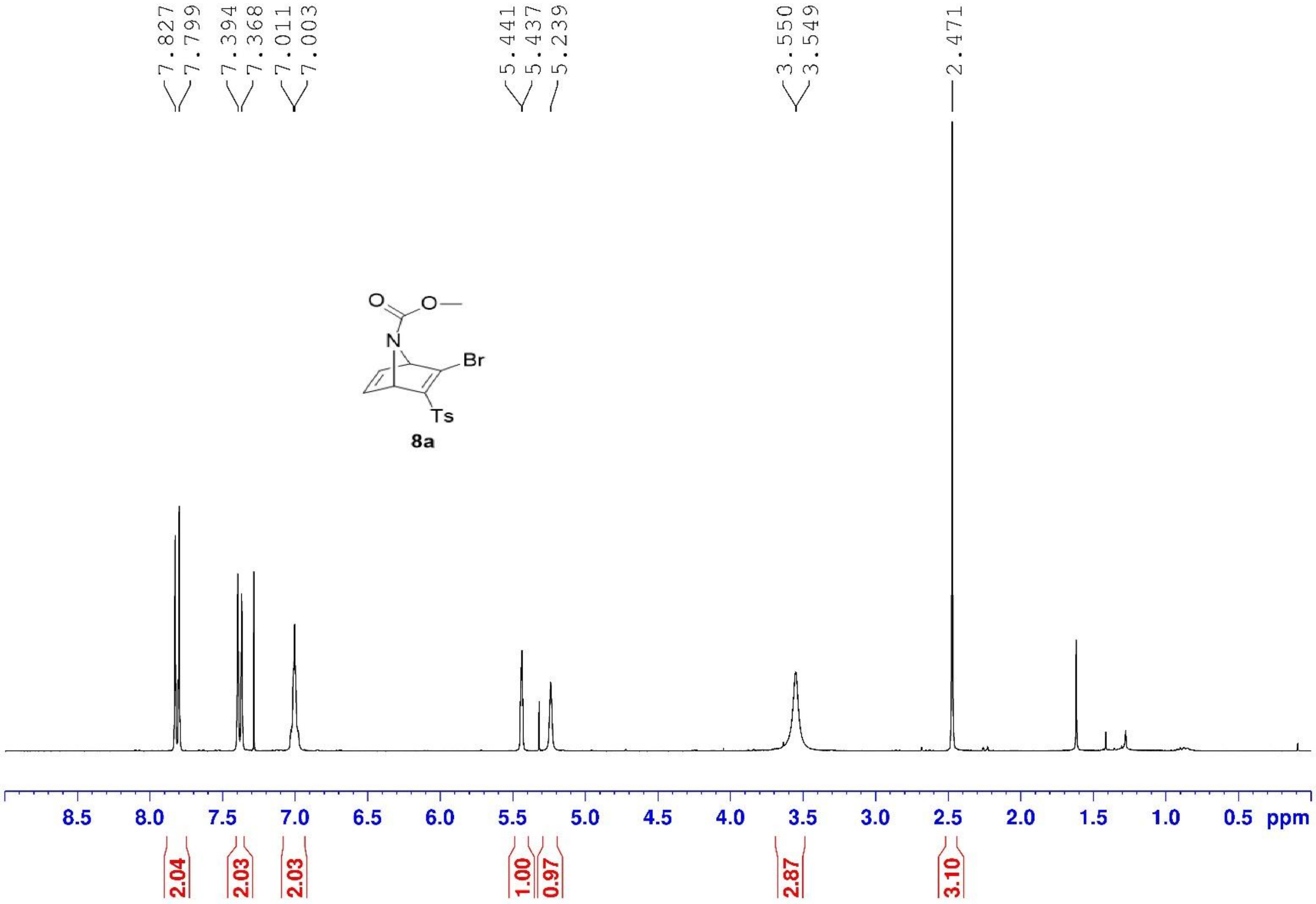

^{1}H-NMR ($CDCl_3$, 300 MHz) of compound **8a**

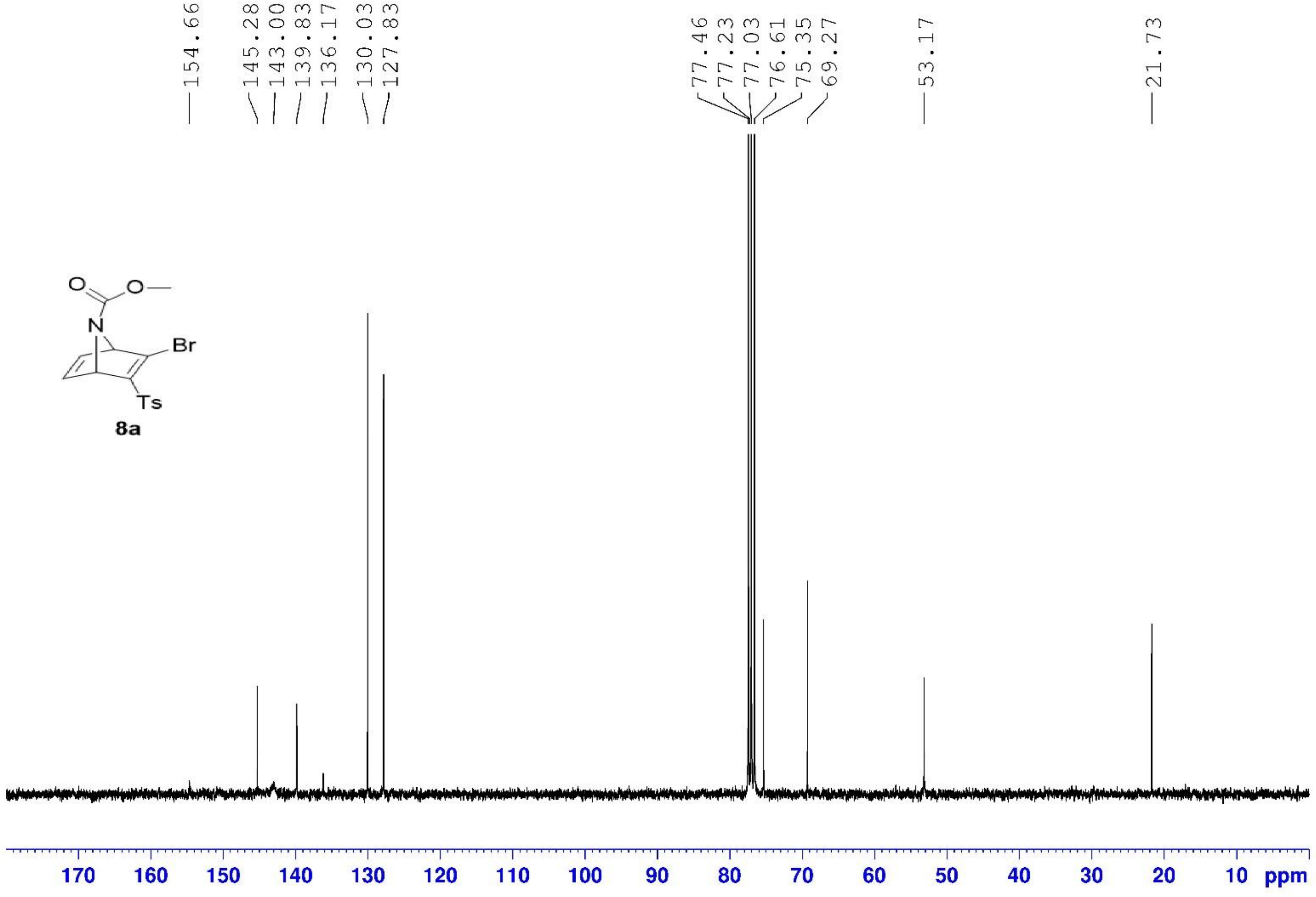

^{13}C-NMR ($CDCl_3$, 75 MHz) of compound **8a**

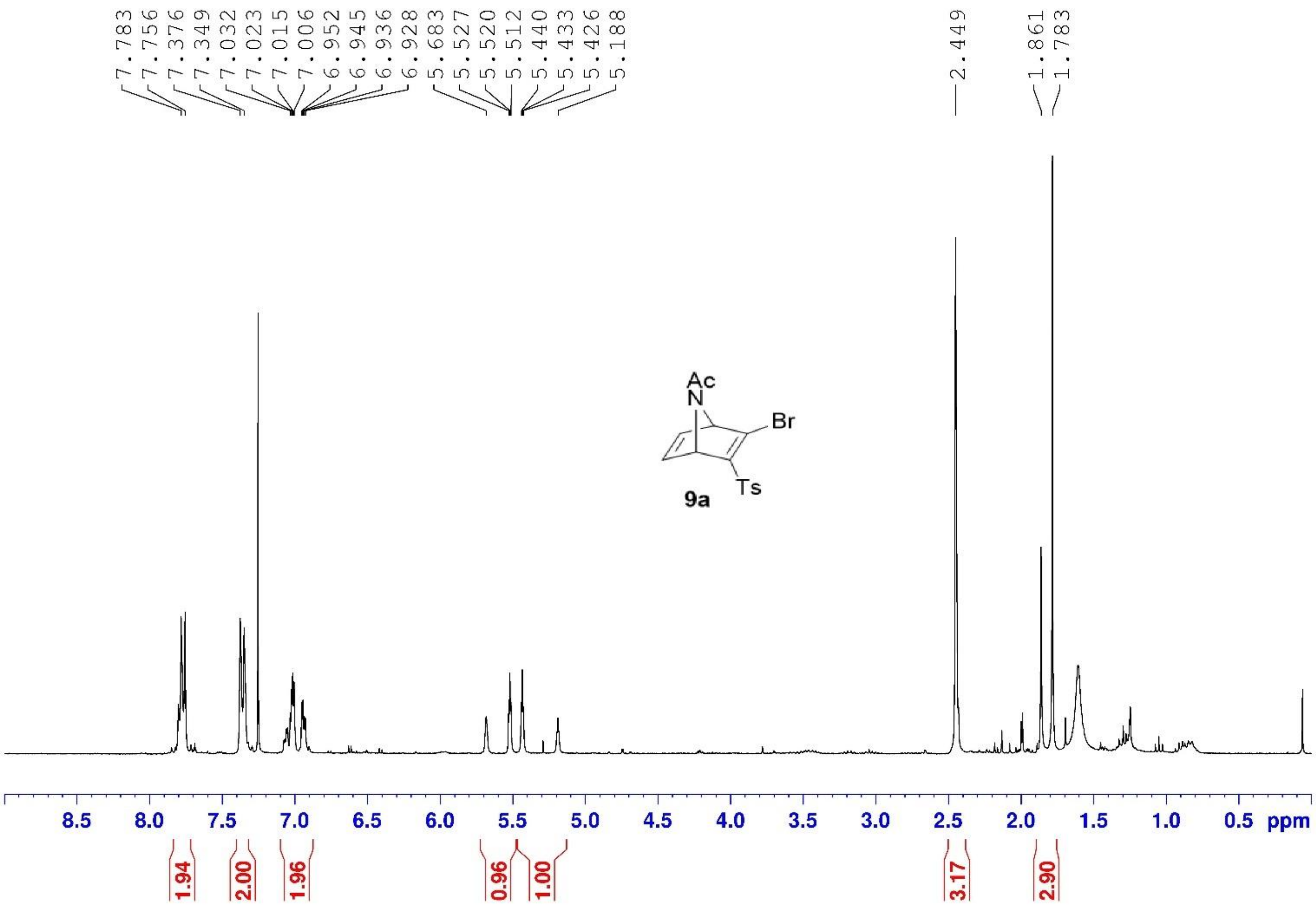

^{1}H-NMR ($CDCl_3$, 300 MHz) of compound **9a**

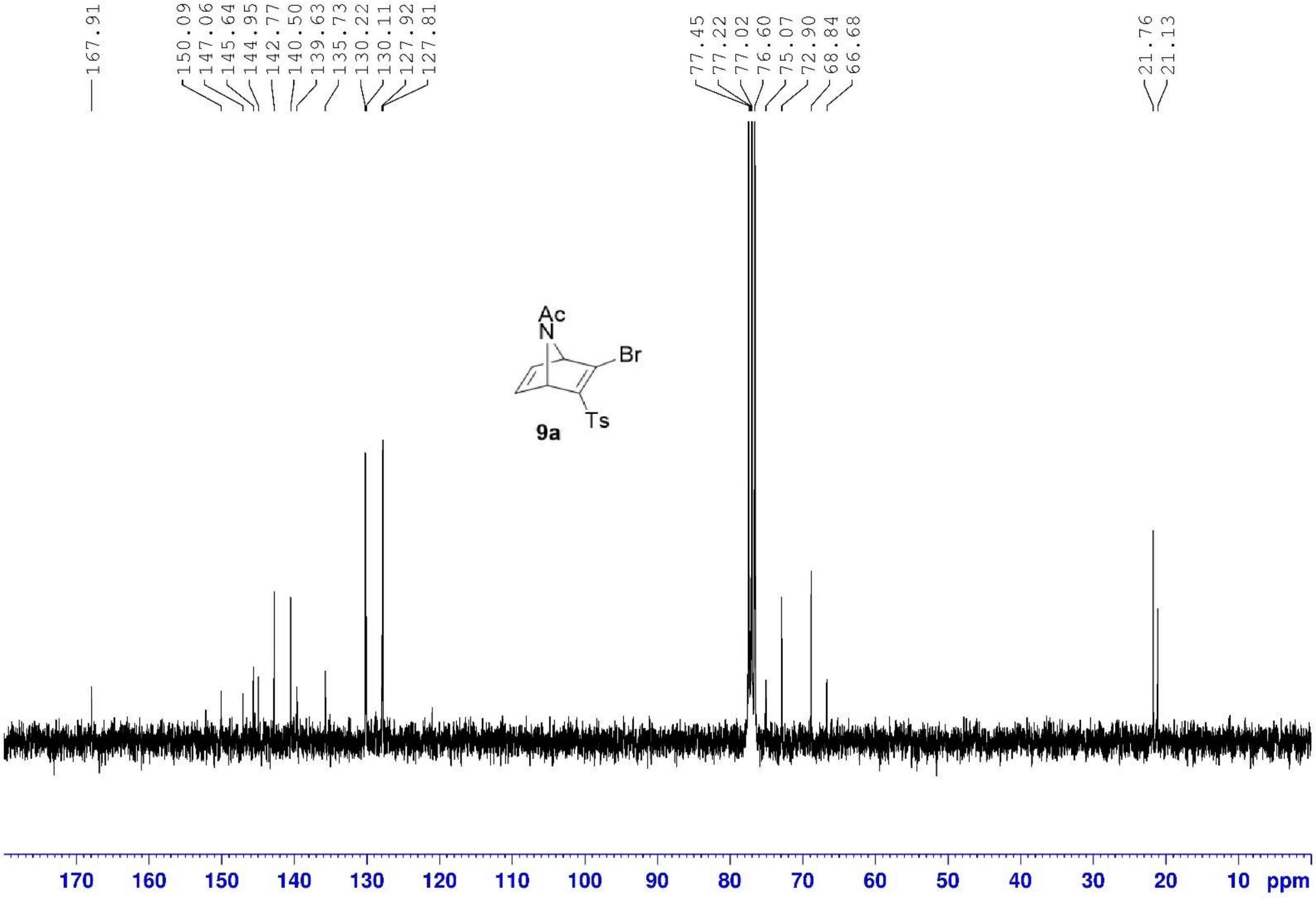

^{13}C-NMR ($CDCl_3$, 75 MHz) of compound **9a**

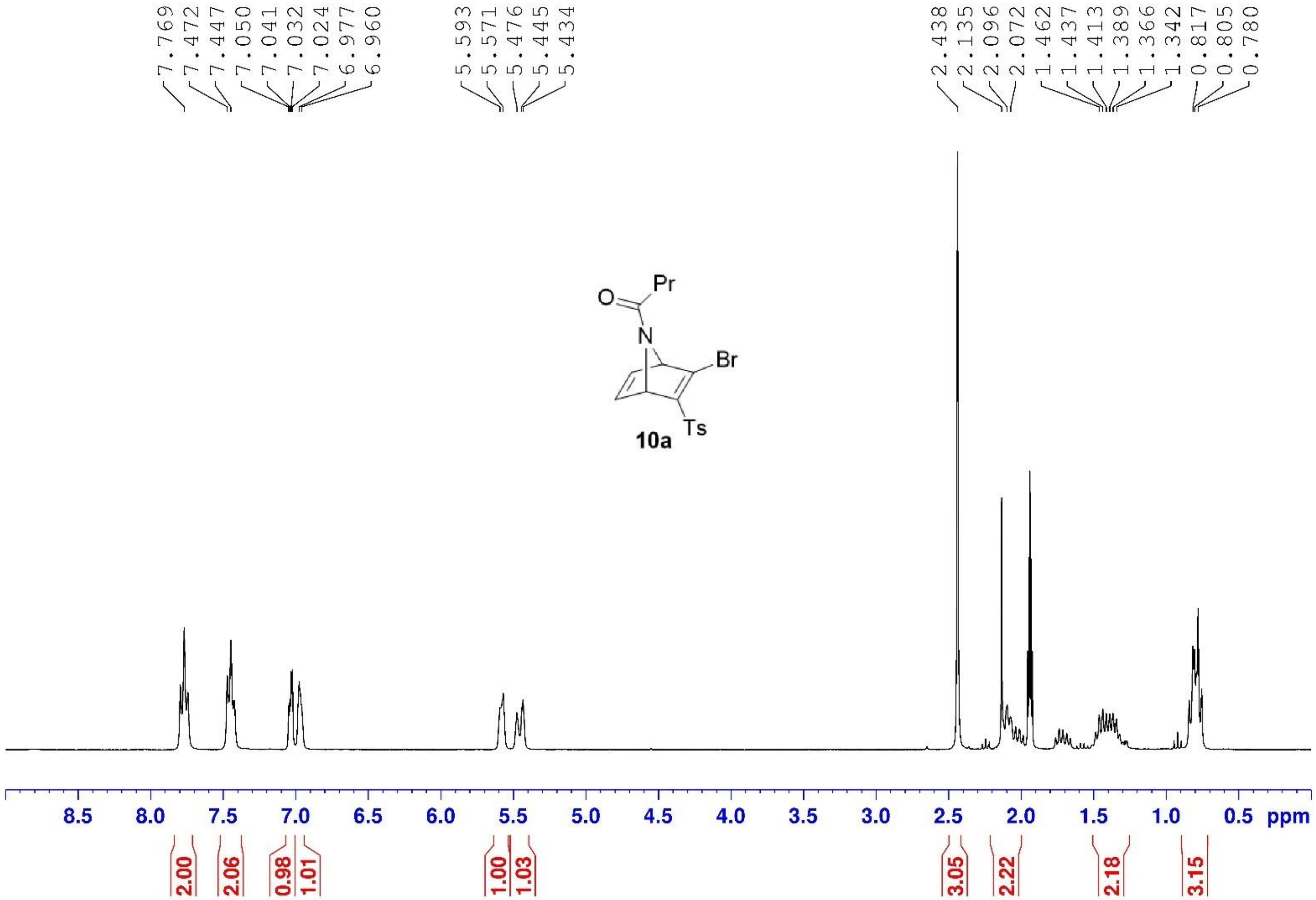

^{1}H-NMR (CD_3CN, 300 MHz) of compound **10a**

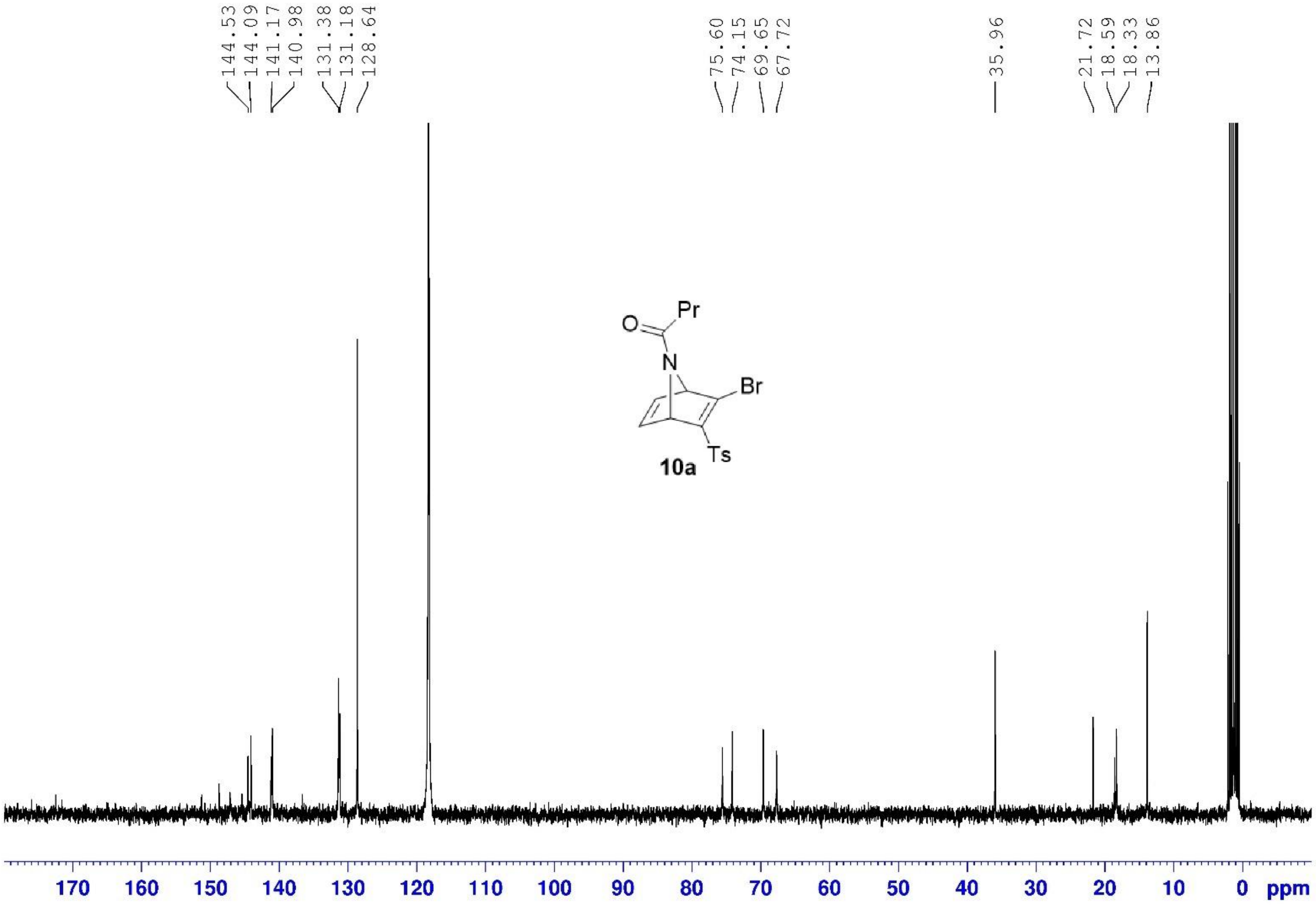

^{13}C-NMR (CD_3CN, 75 MHz) of compound **10a**

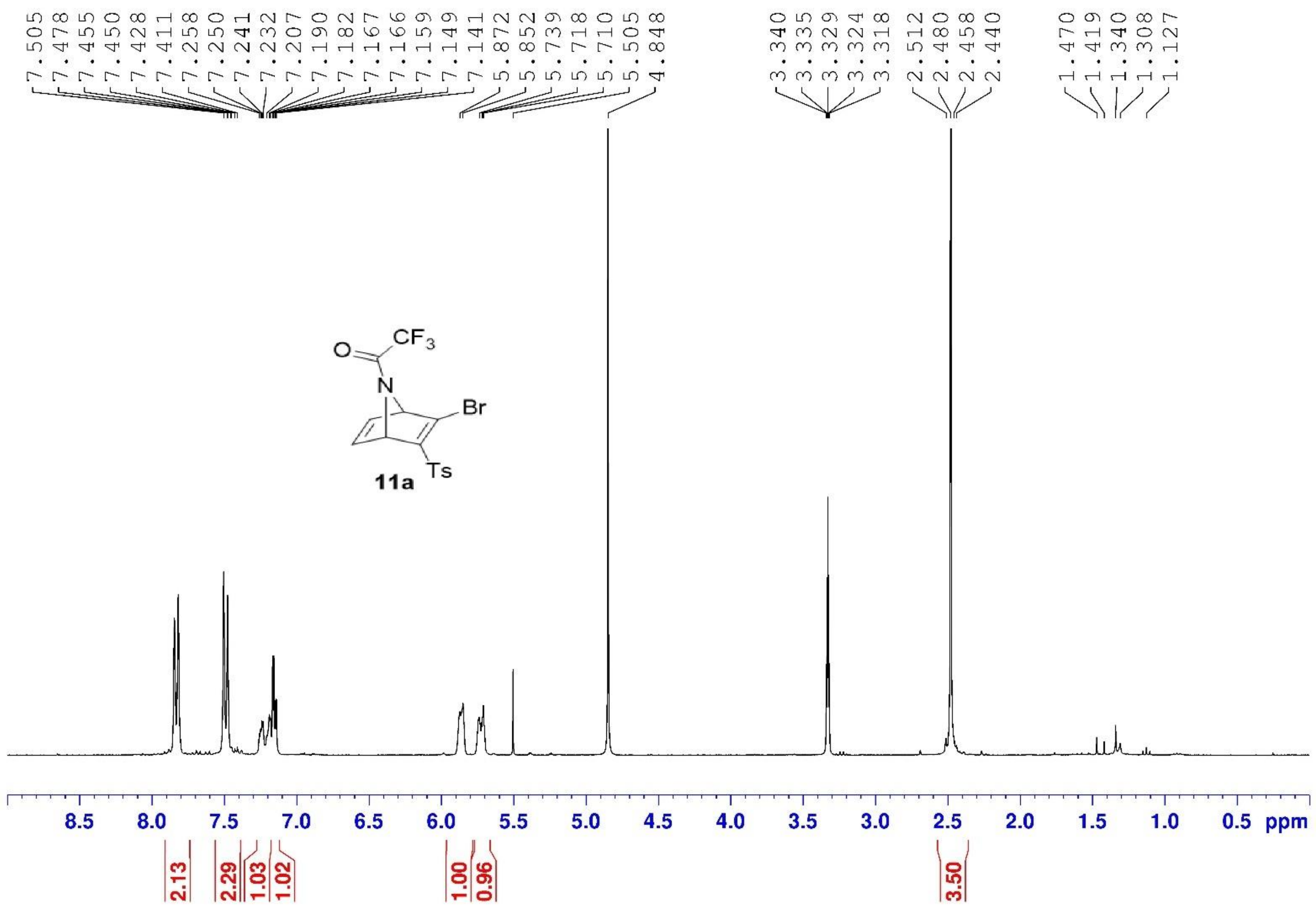

^{1}H-NMR (CD_3OD, 300 MHz) of compound **11a**

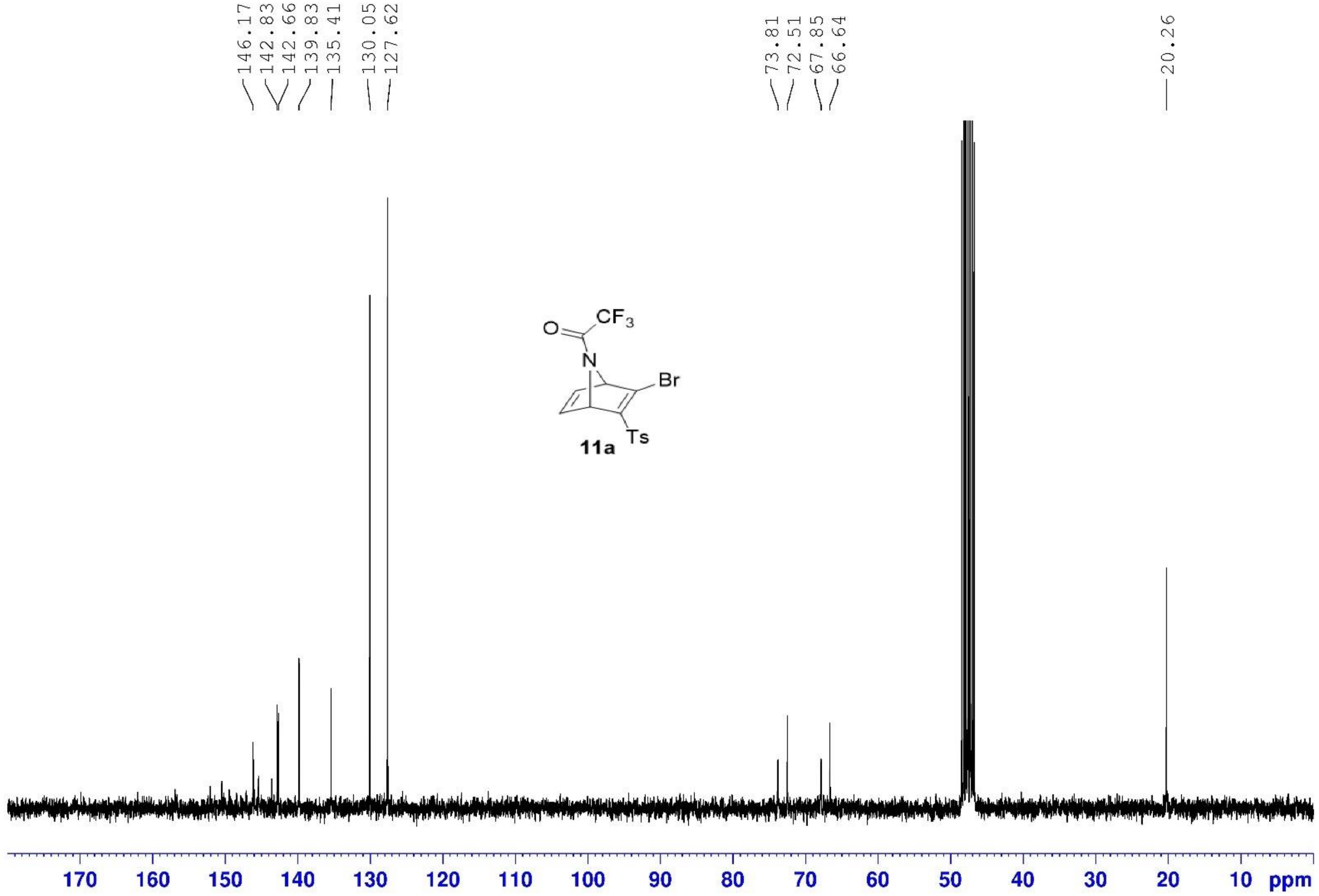

^{13}C-NMR (CD_3OD, 75 MHz) of compound **11a**

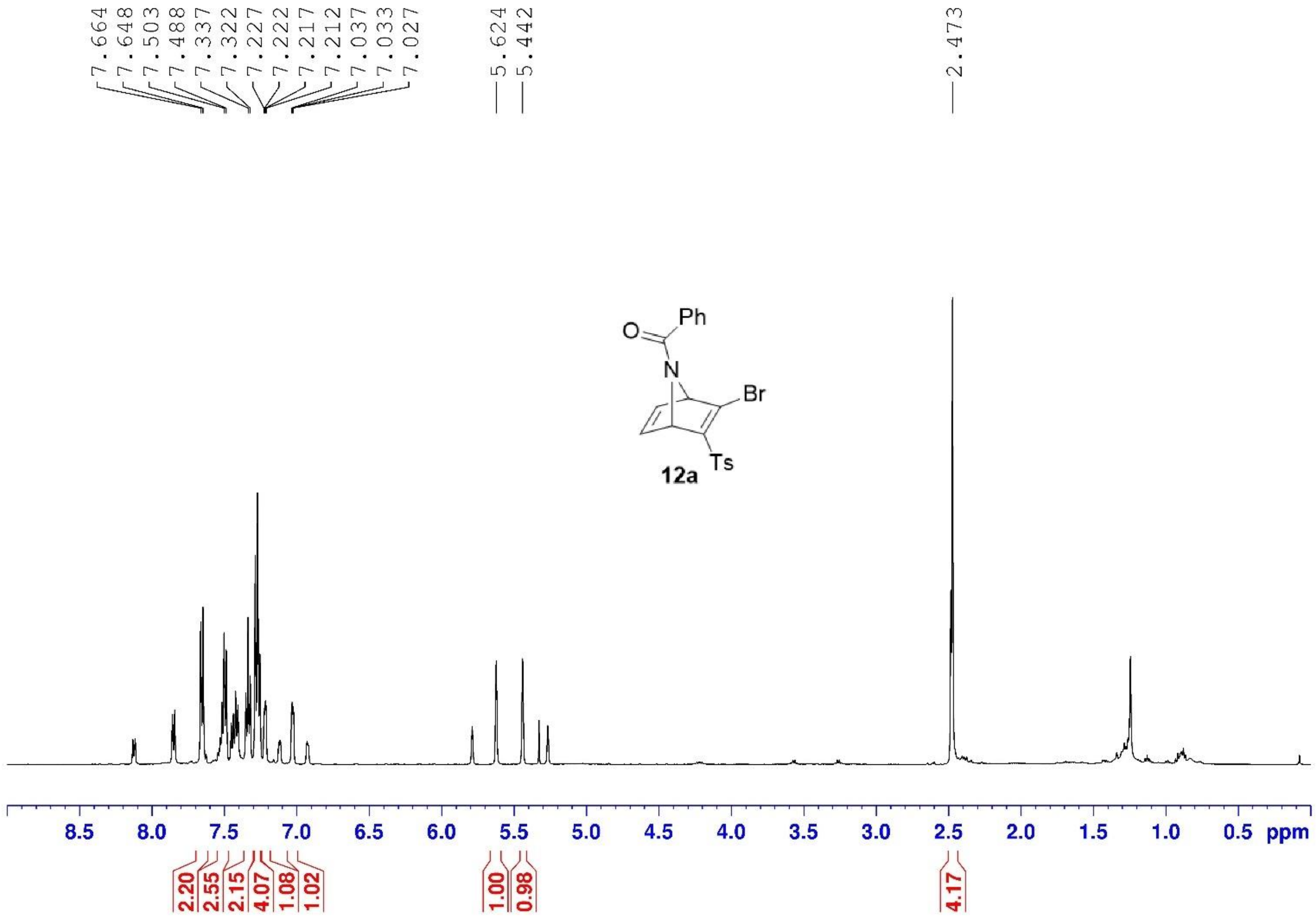

^{1}H-NMR ($CDCl_3$, 500 MHz, 253 K) of compound **12a**

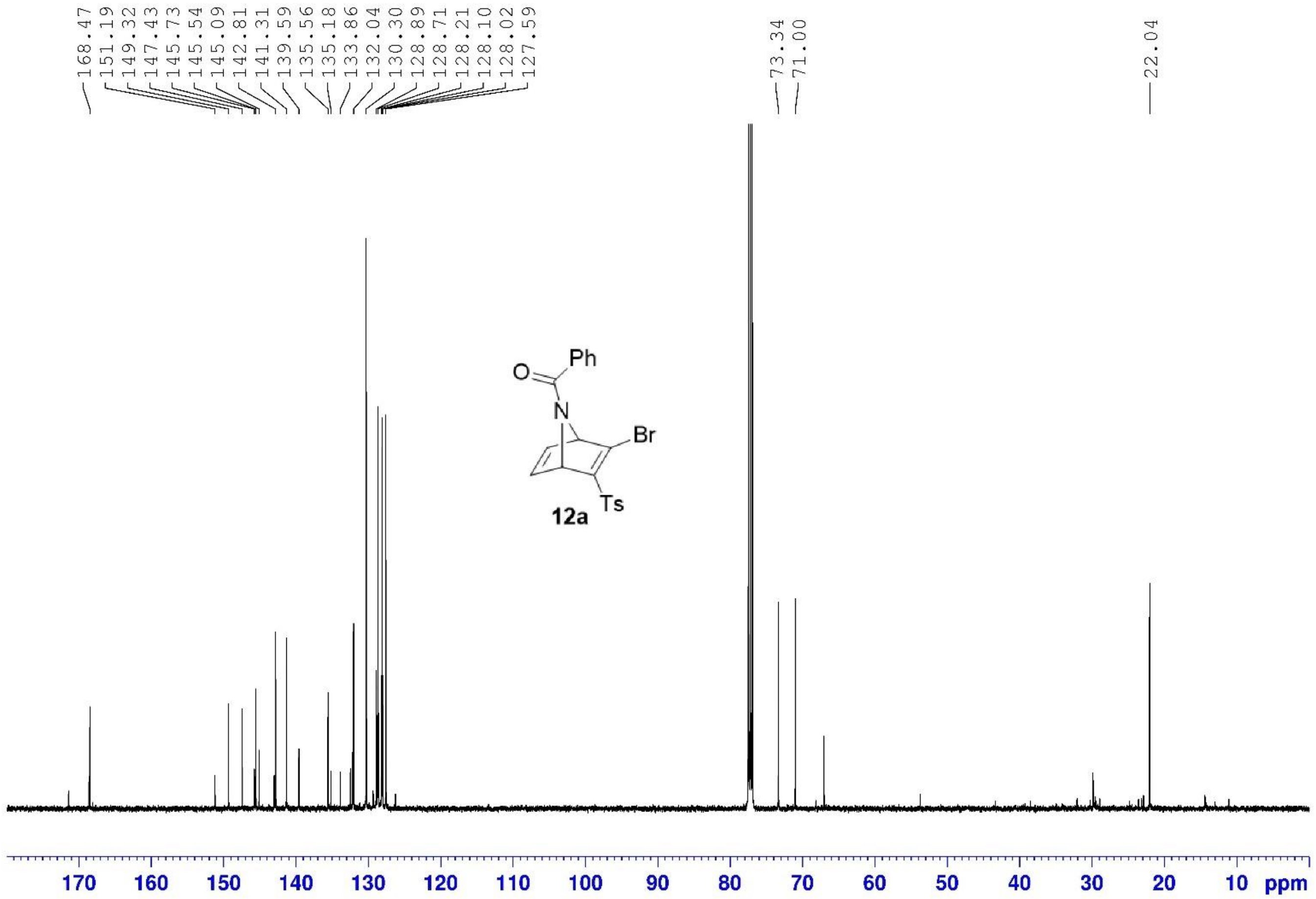

^{13}C-NMR ($CDCl_3$, 125 MHz, 253 K) of compound **12a**

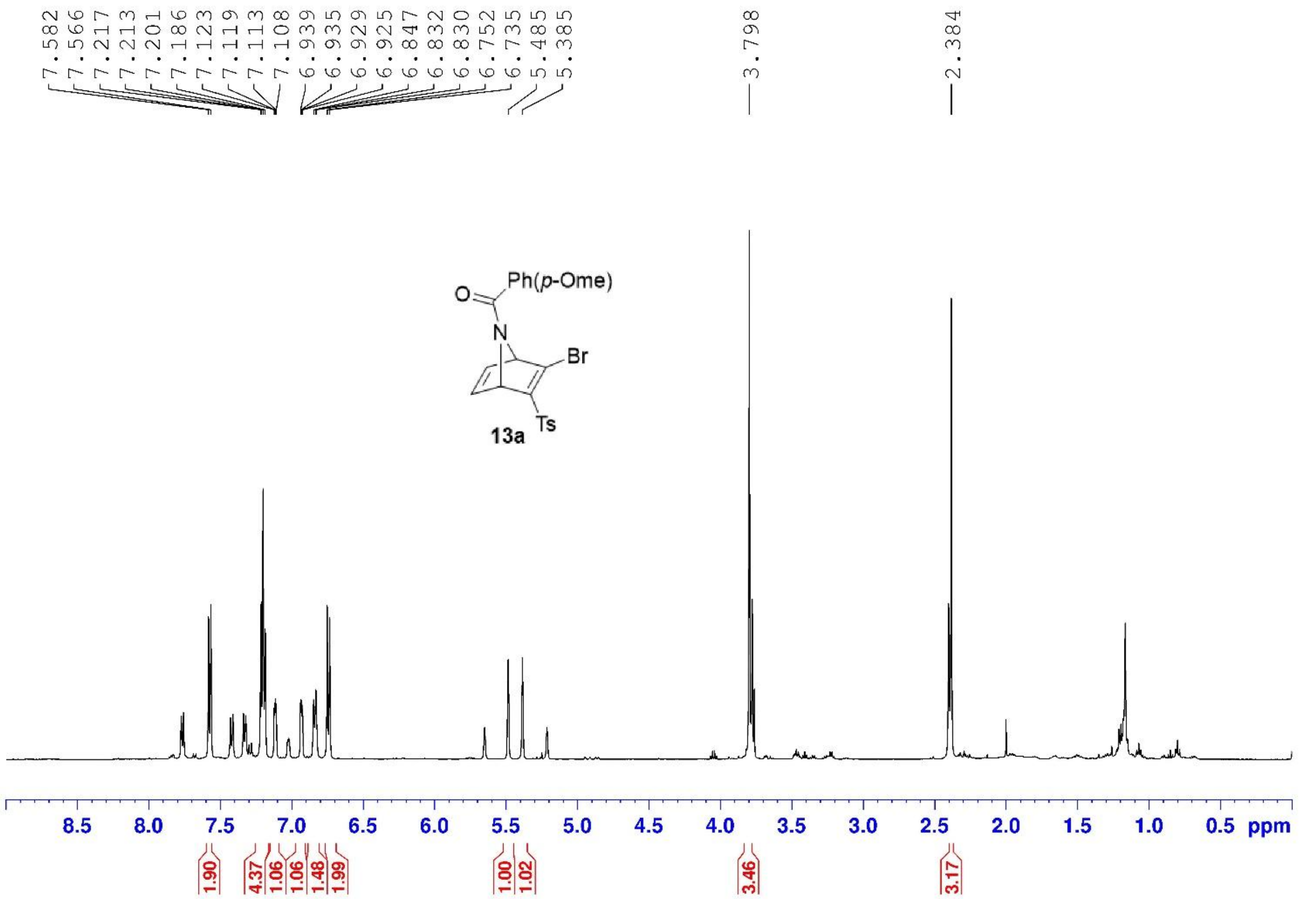

^{1}H-NMR ($CDCl_3$, 500 MHz, 253 K) of compound **13a**

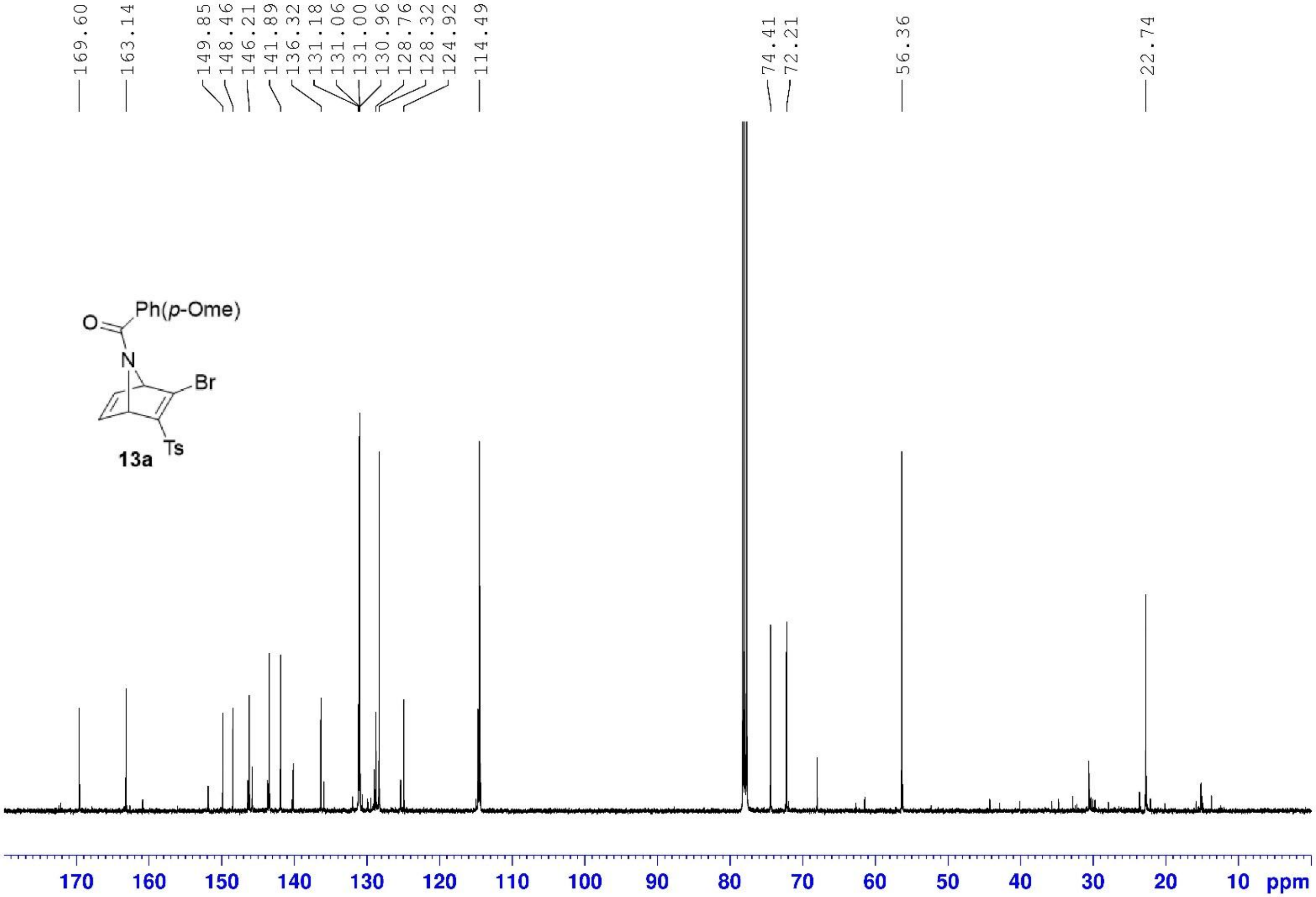

^{13}C-NMR ($CDCl_3$, 125 MHz, 253 K) of compound **13a**

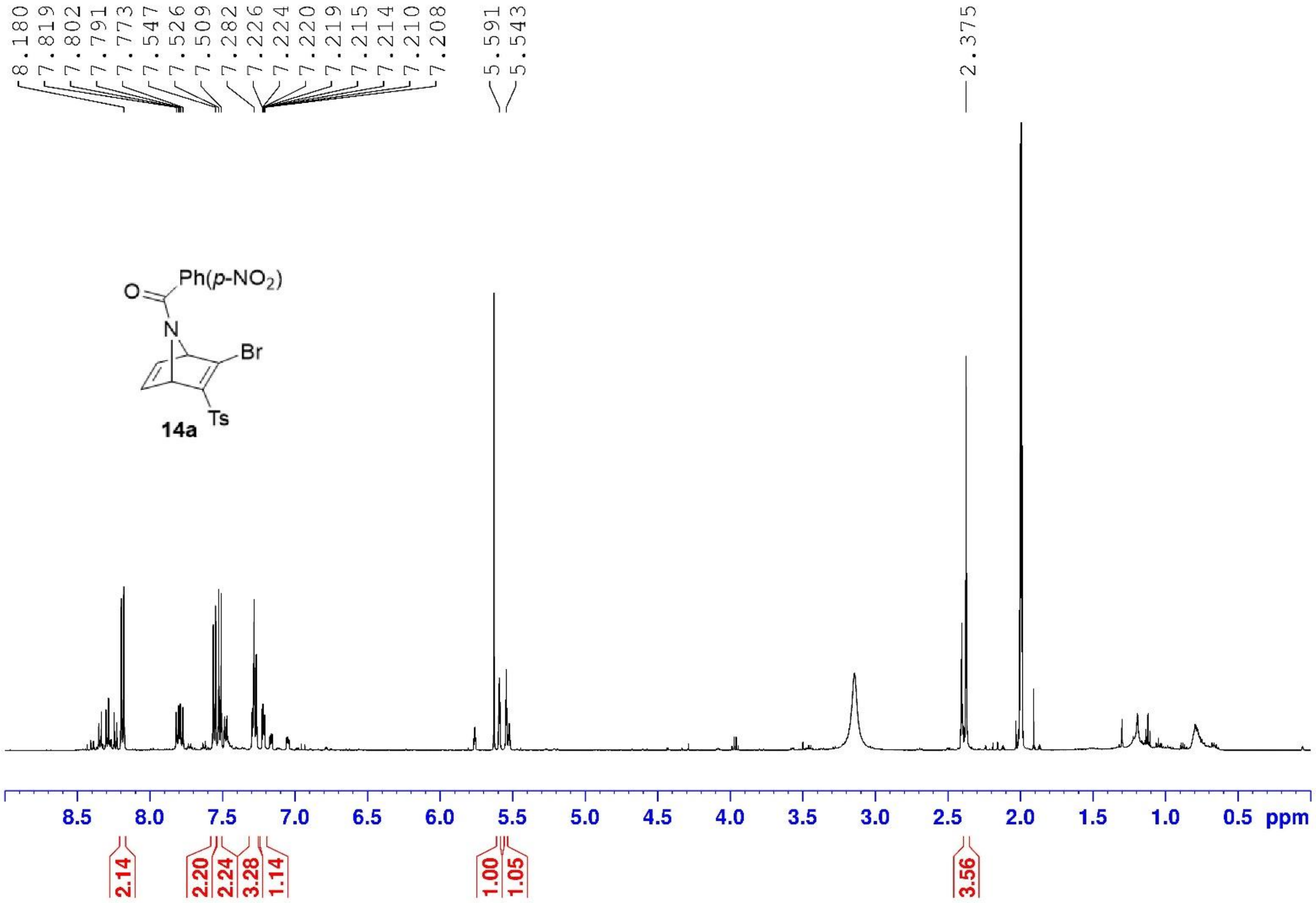

^{1}H-NMR ($(CD_3)CO$, 500 MHz, 253 K) of compound **14a**

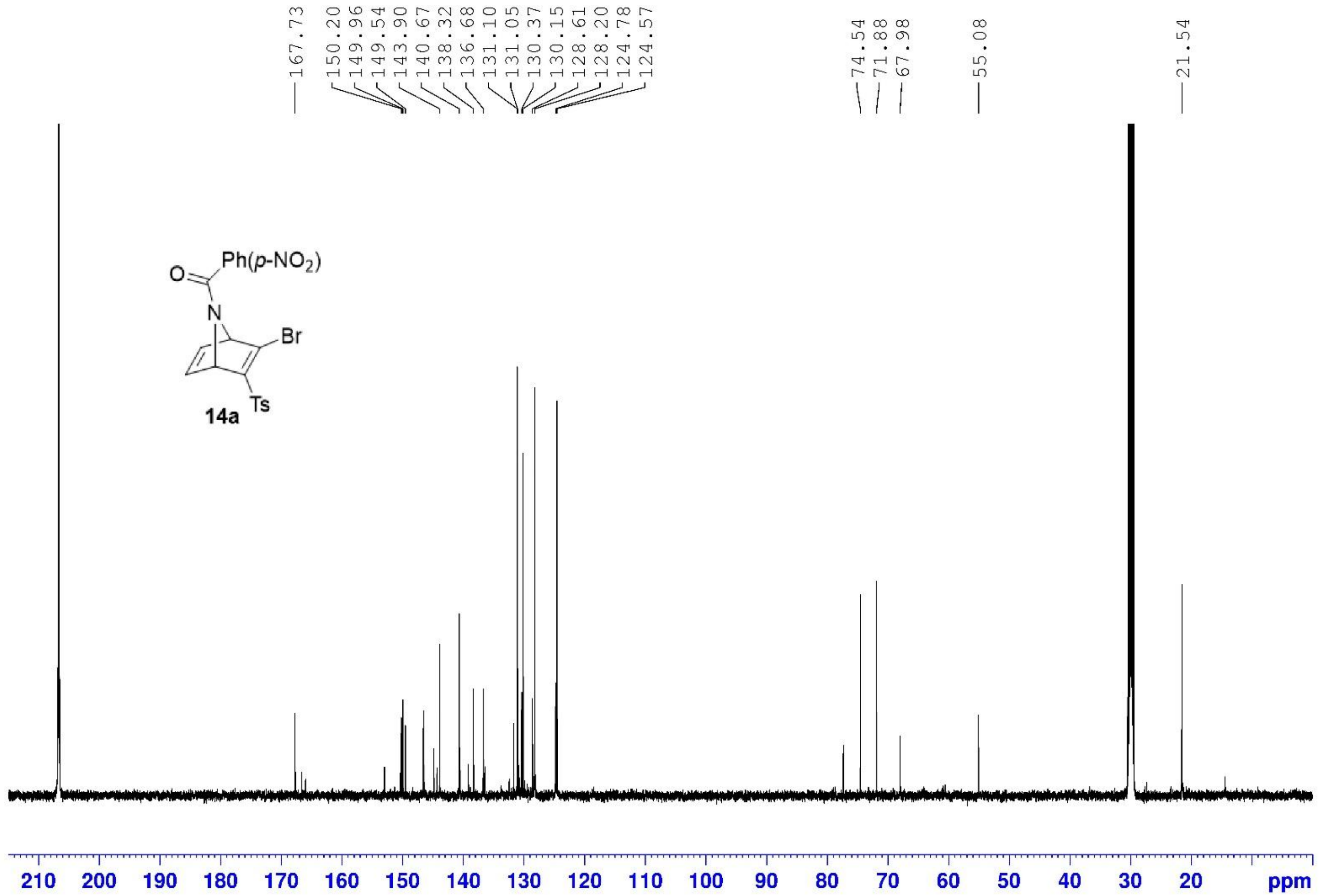

^{13}C-NMR ((CD$_3$)CO, 125 MHz, 253 K) of compound **14a**

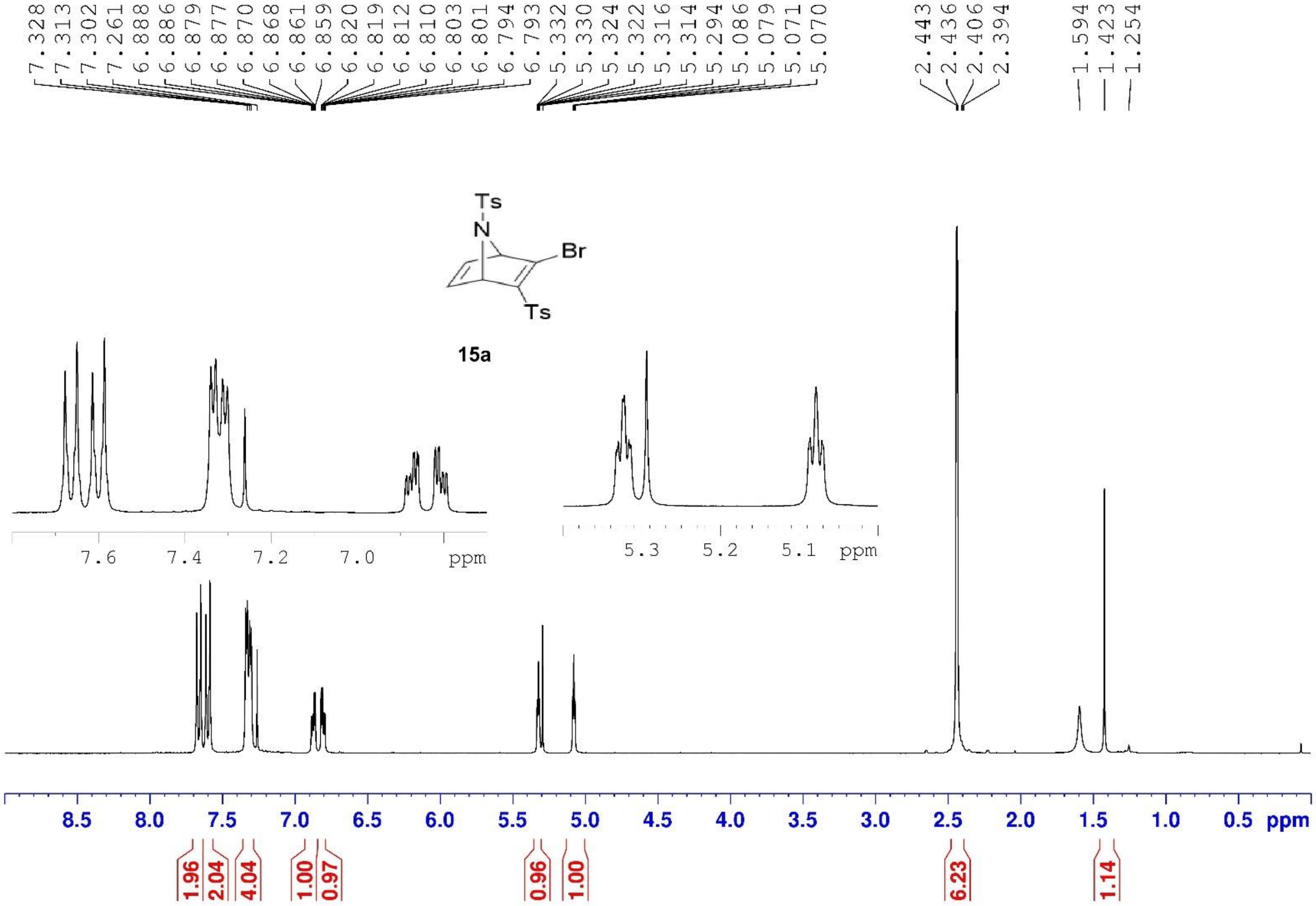

^{1}H-NMR ($CDCl_3$, 300 MHz) of compound **15a**

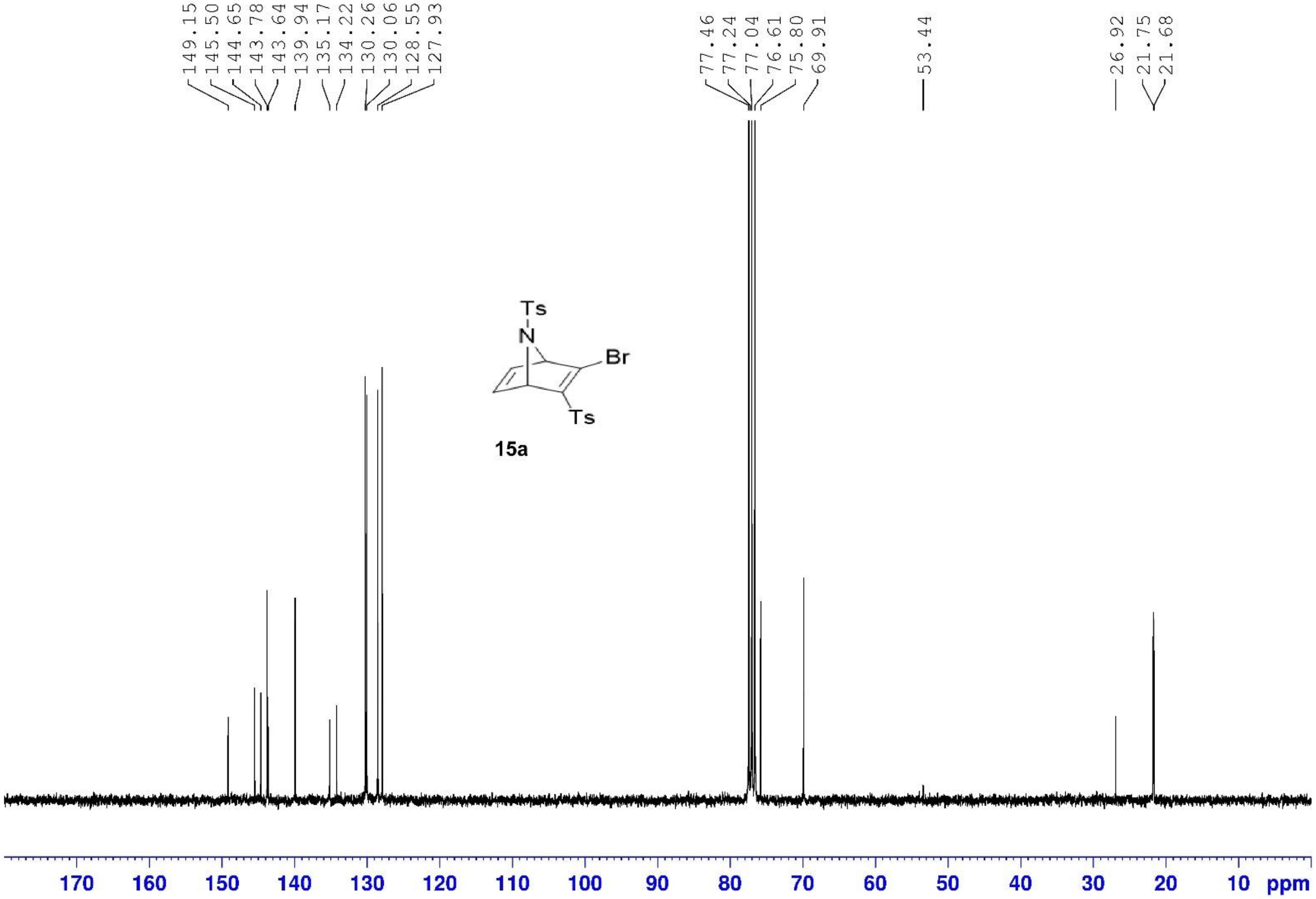

^{13}C-NMR ($CDCl_3$, 75 MHz) of compound **15a**

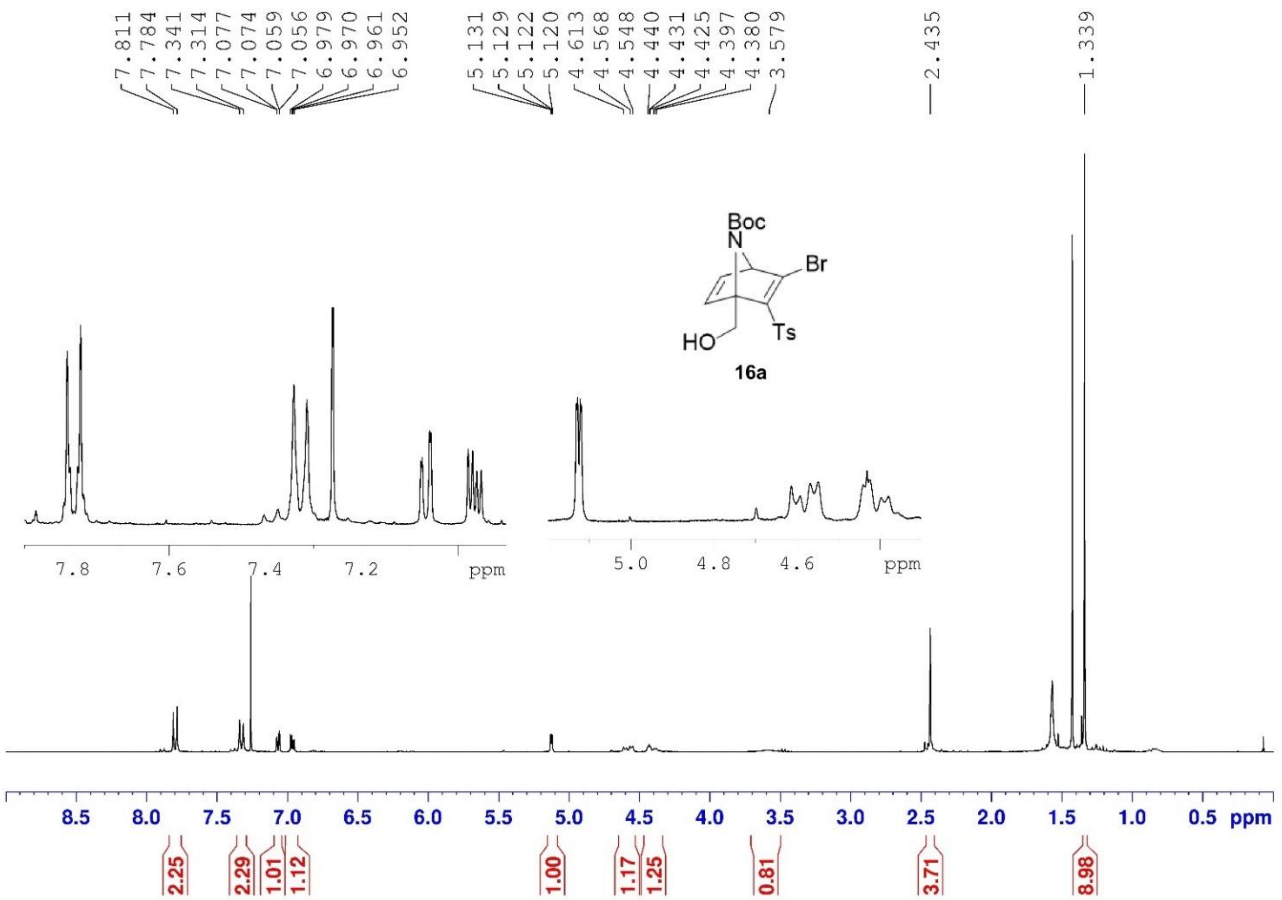

^{1}H-NMR ($CDCl_3$, 300 MHz) of compound **16a**

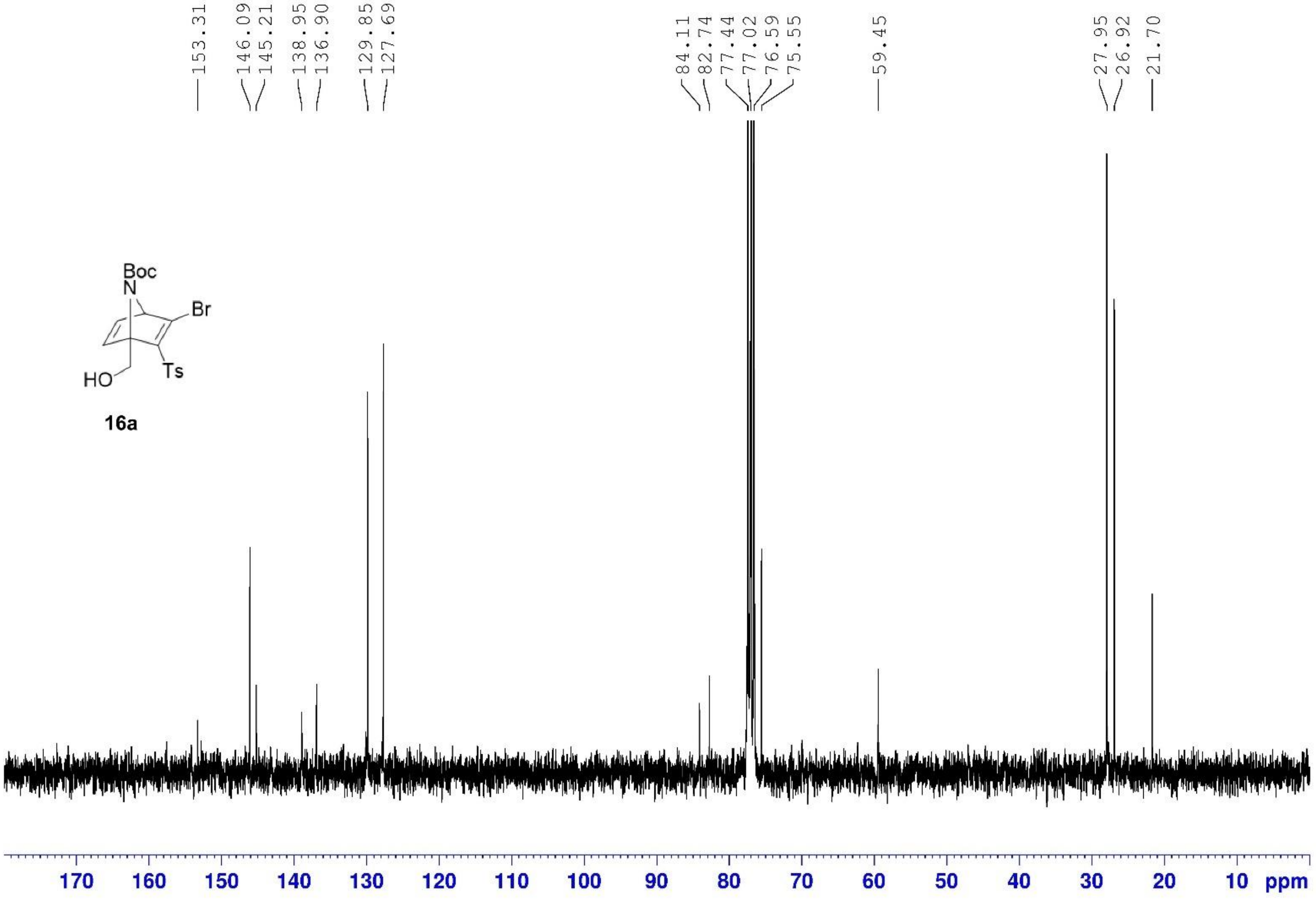

16a

^{13}C-NMR ($CDCl_3$, 75 MHz) of compound **16a**

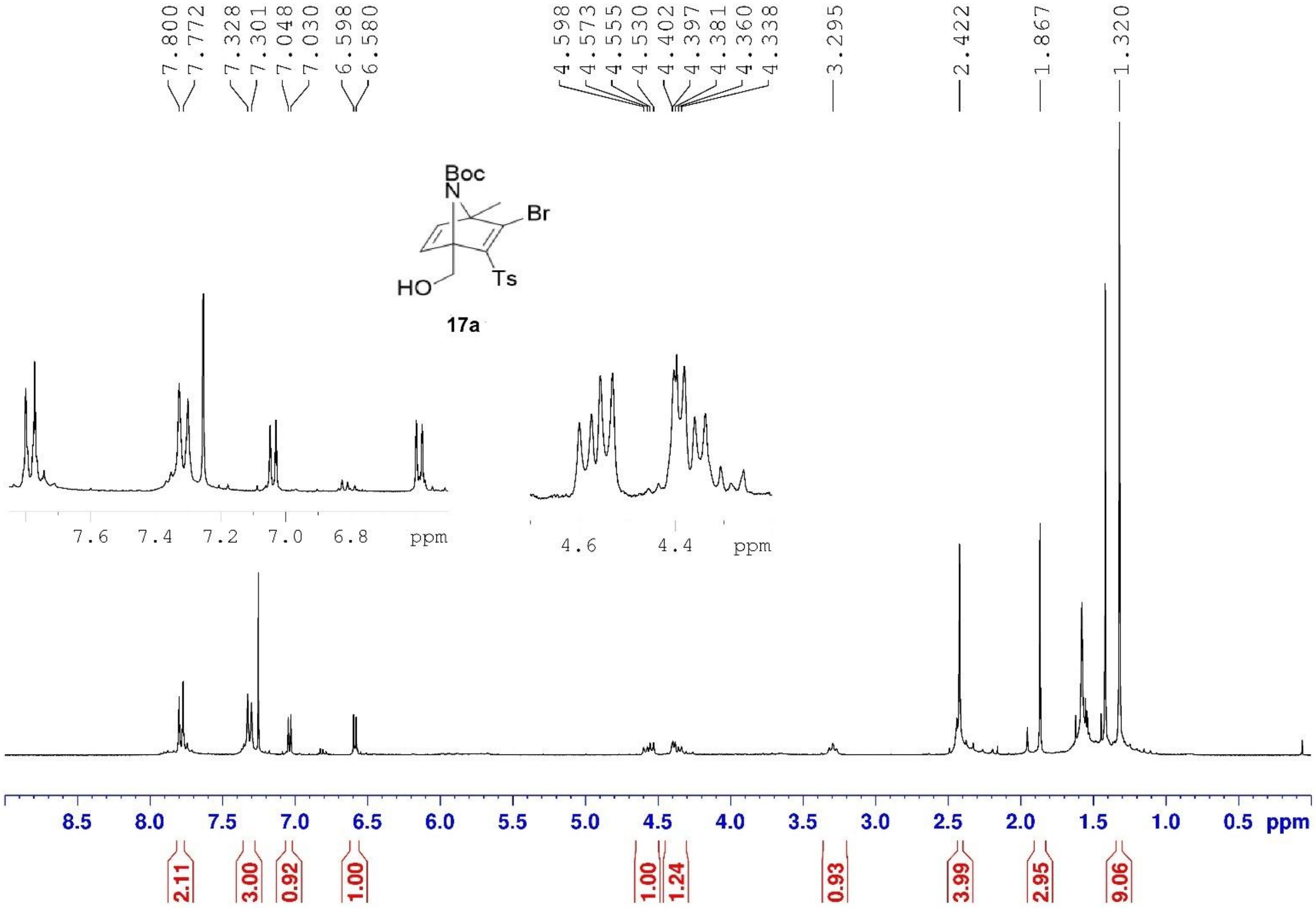

^{1}H-NMR ($CDCl_3$, 300 MHz) of compound **17a**

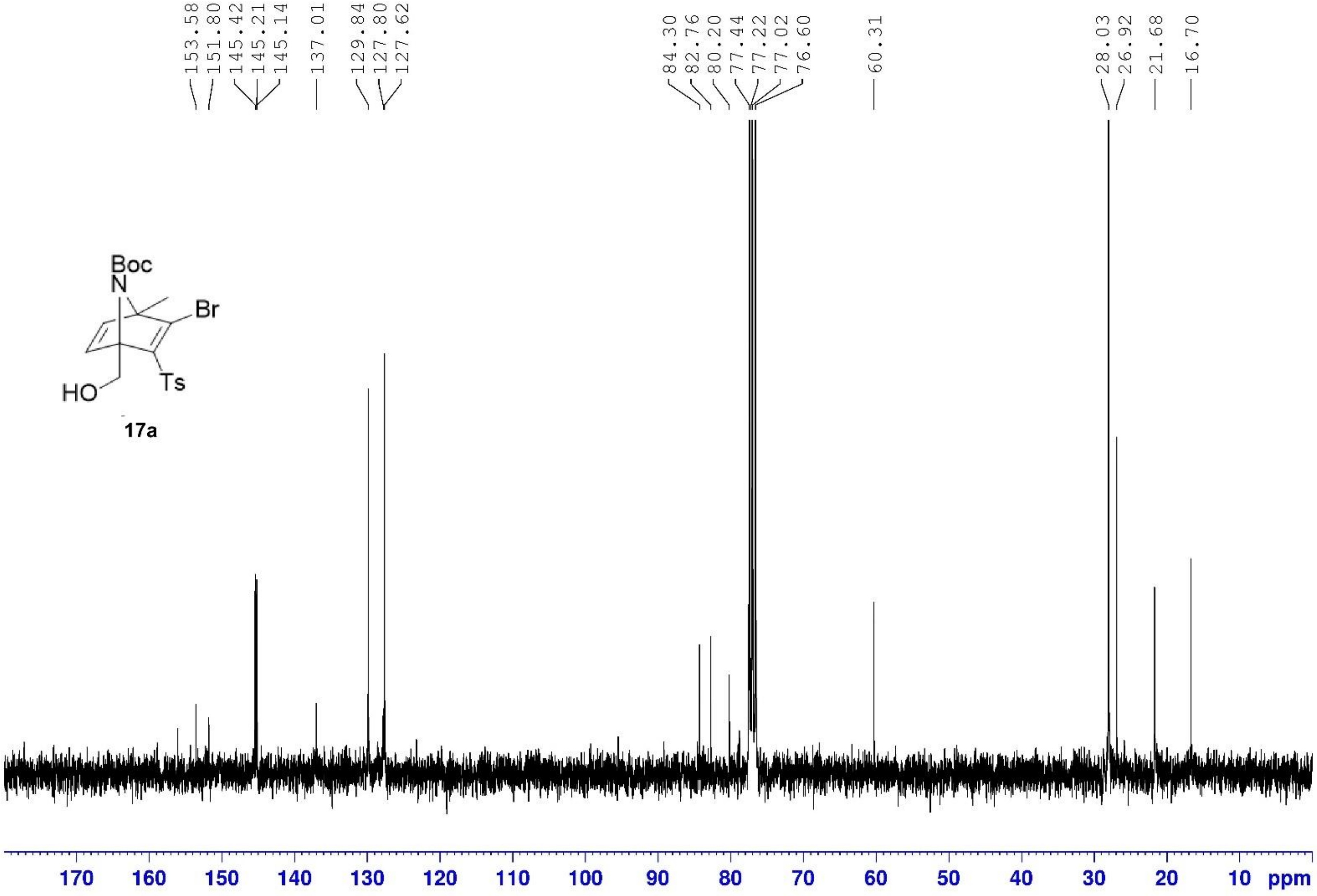

^{13}C-NMR ($CDCl_3$, 75 MHz) of compound **17a**

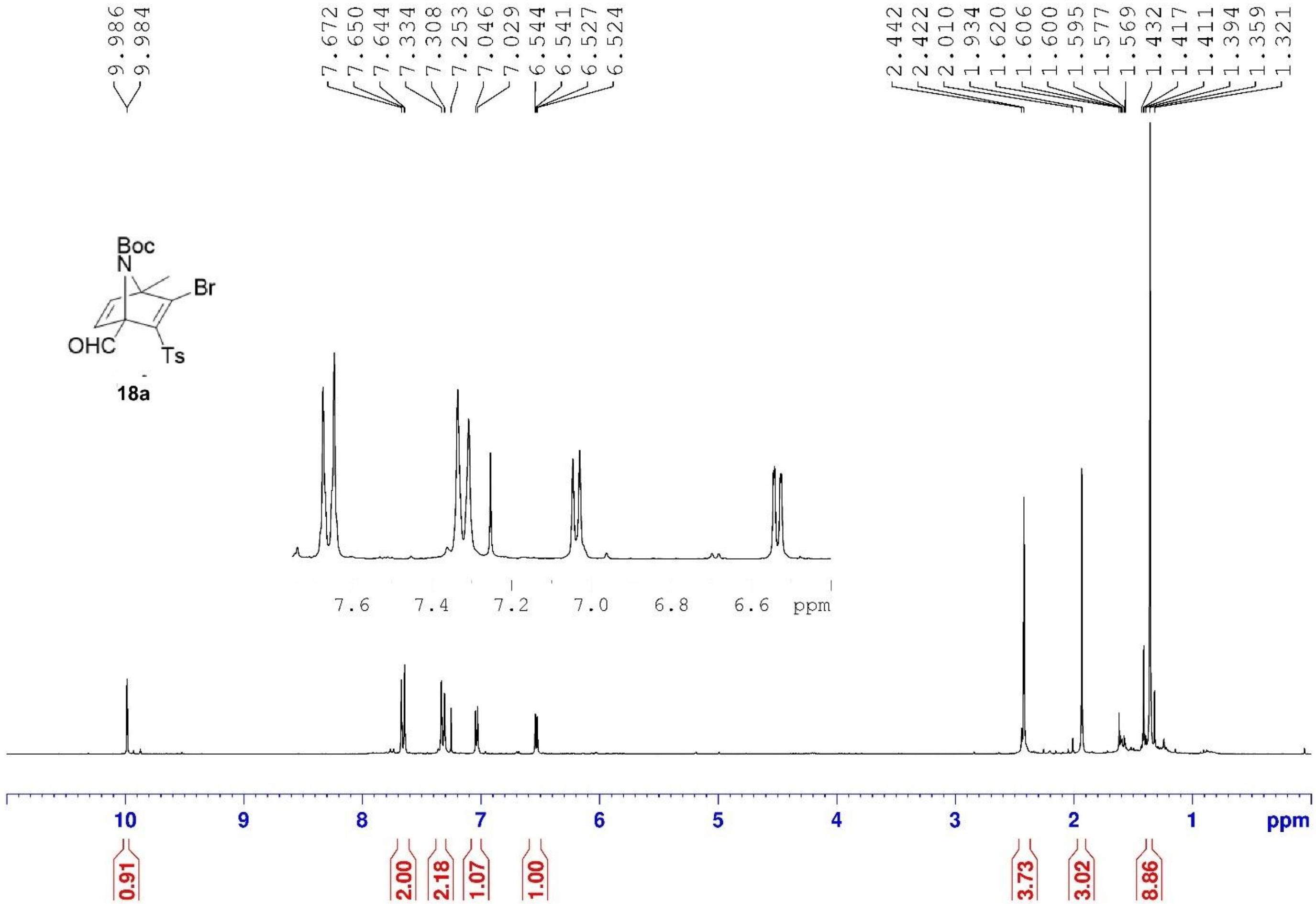

^{1}H-NMR ($CDCl_3$, 300 MHz) of compound **18a**

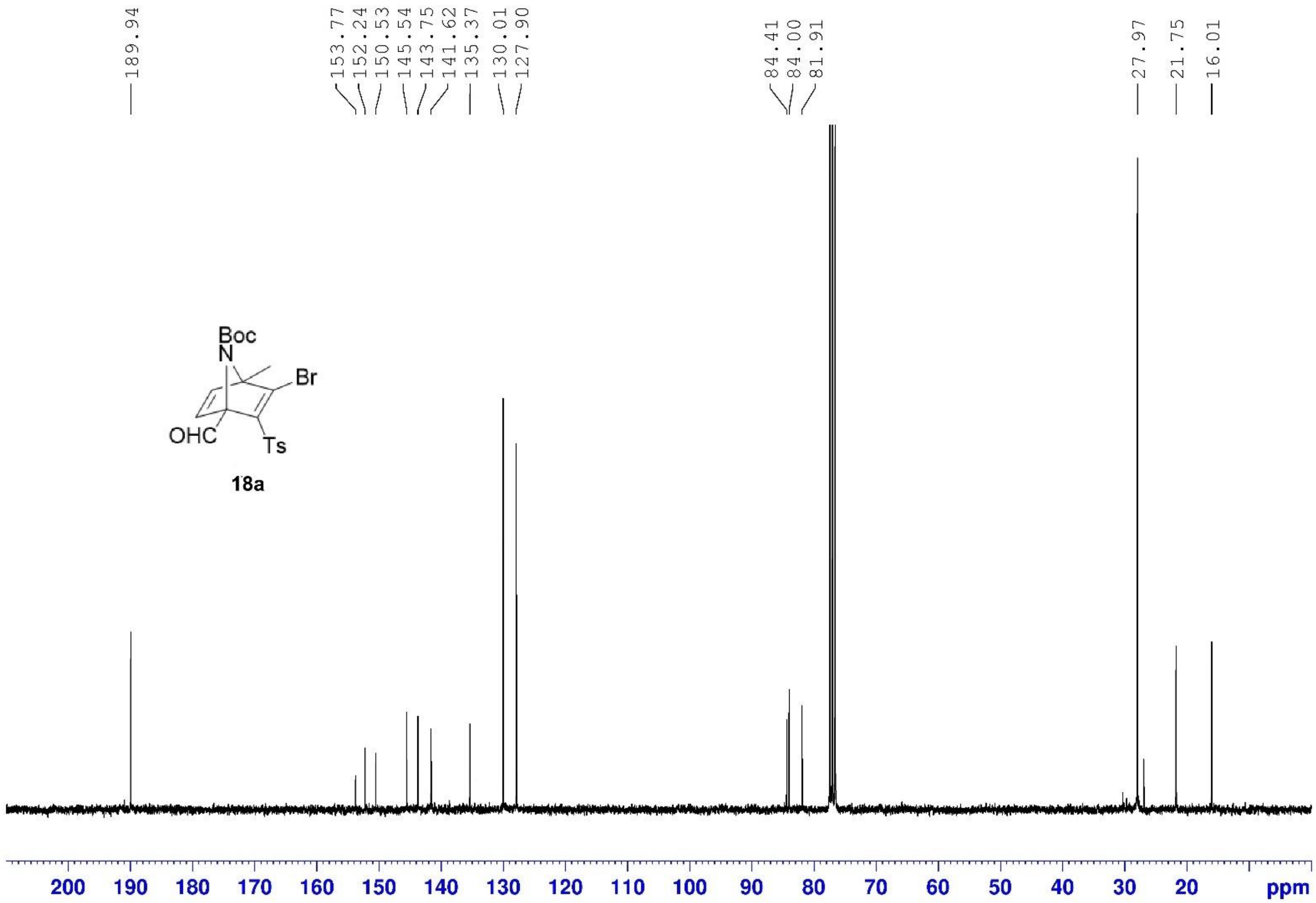

^{13}C-NMR ($CDCl_3$, 75 MHz) of compound **18a**

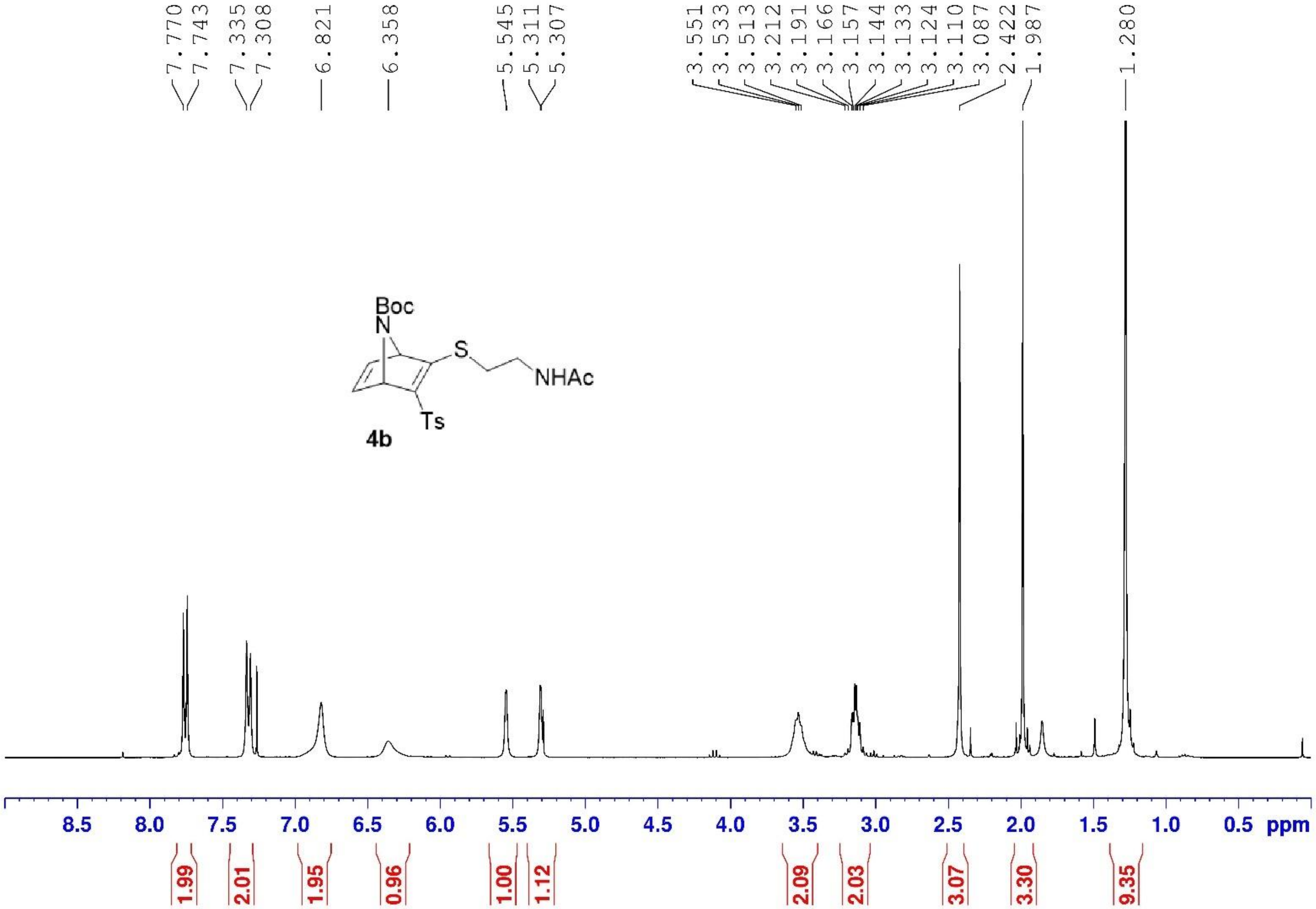

^{1}H-NMR ($CDCl_3$, 300 MHz) of compound **4b**

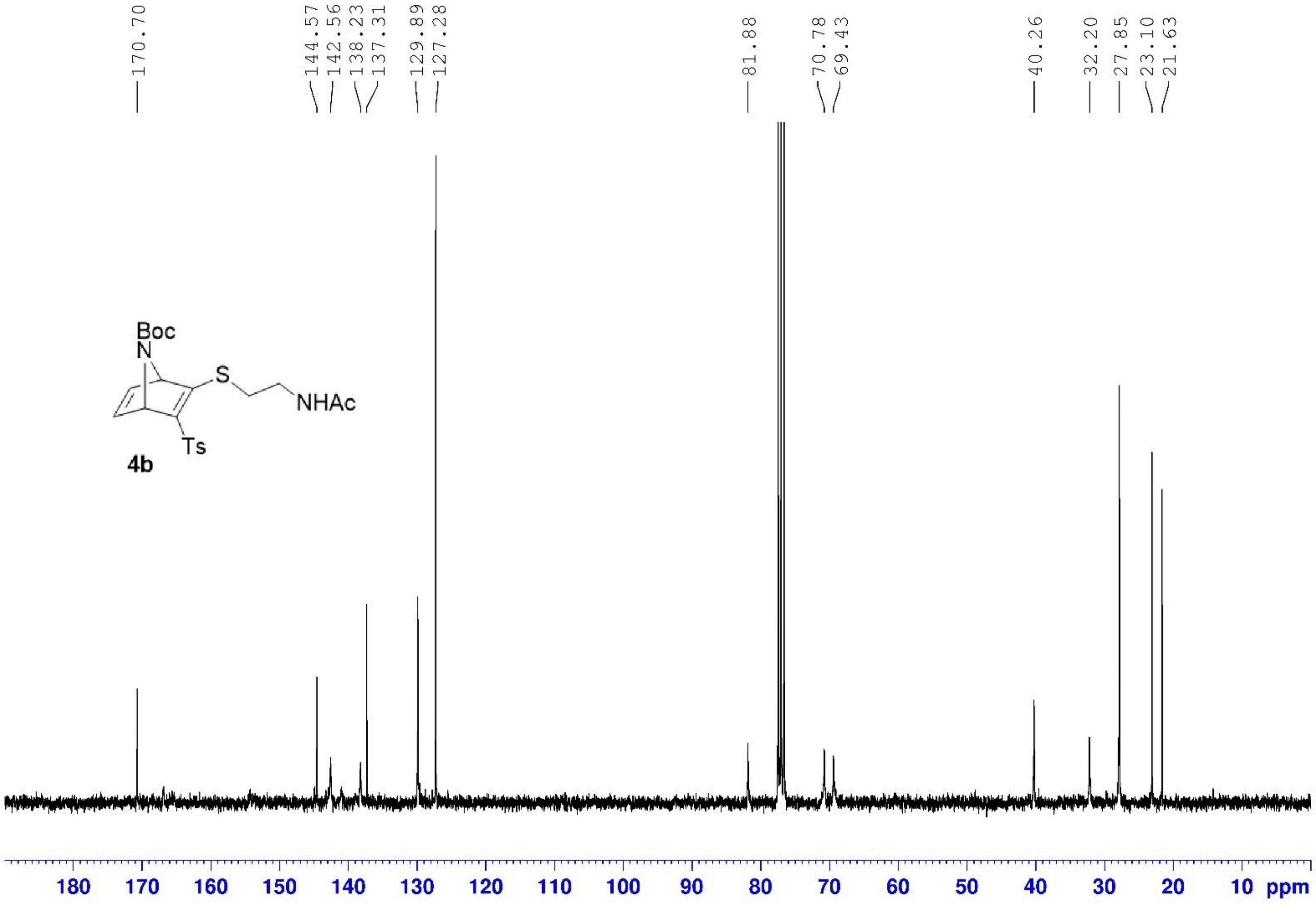

^{13}C-NMR ($CDCl_3$, 75 MHz) of compound **4b**

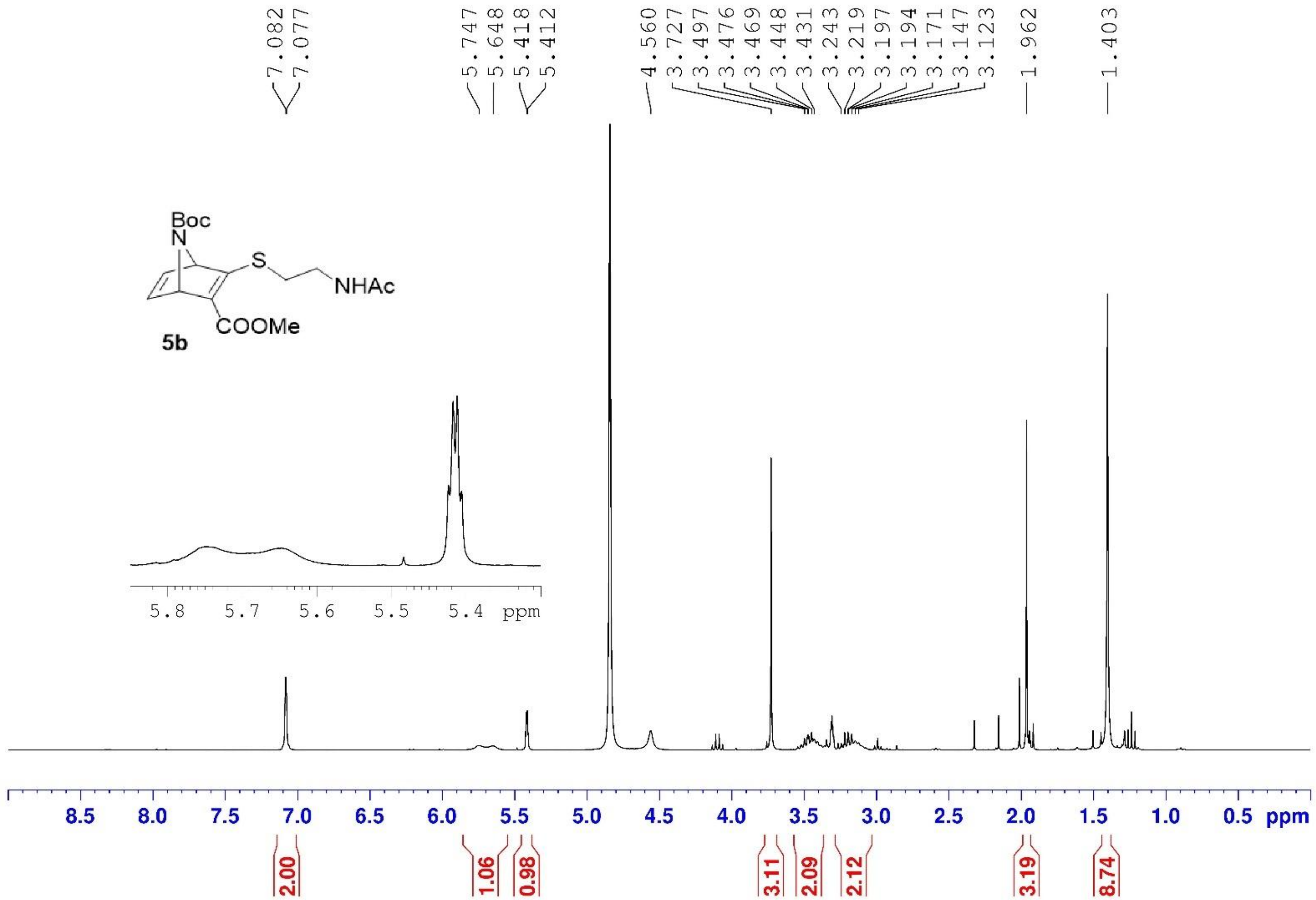

^{1}H-NMR (CD_3OD, 300 MHz) of compound **5b**

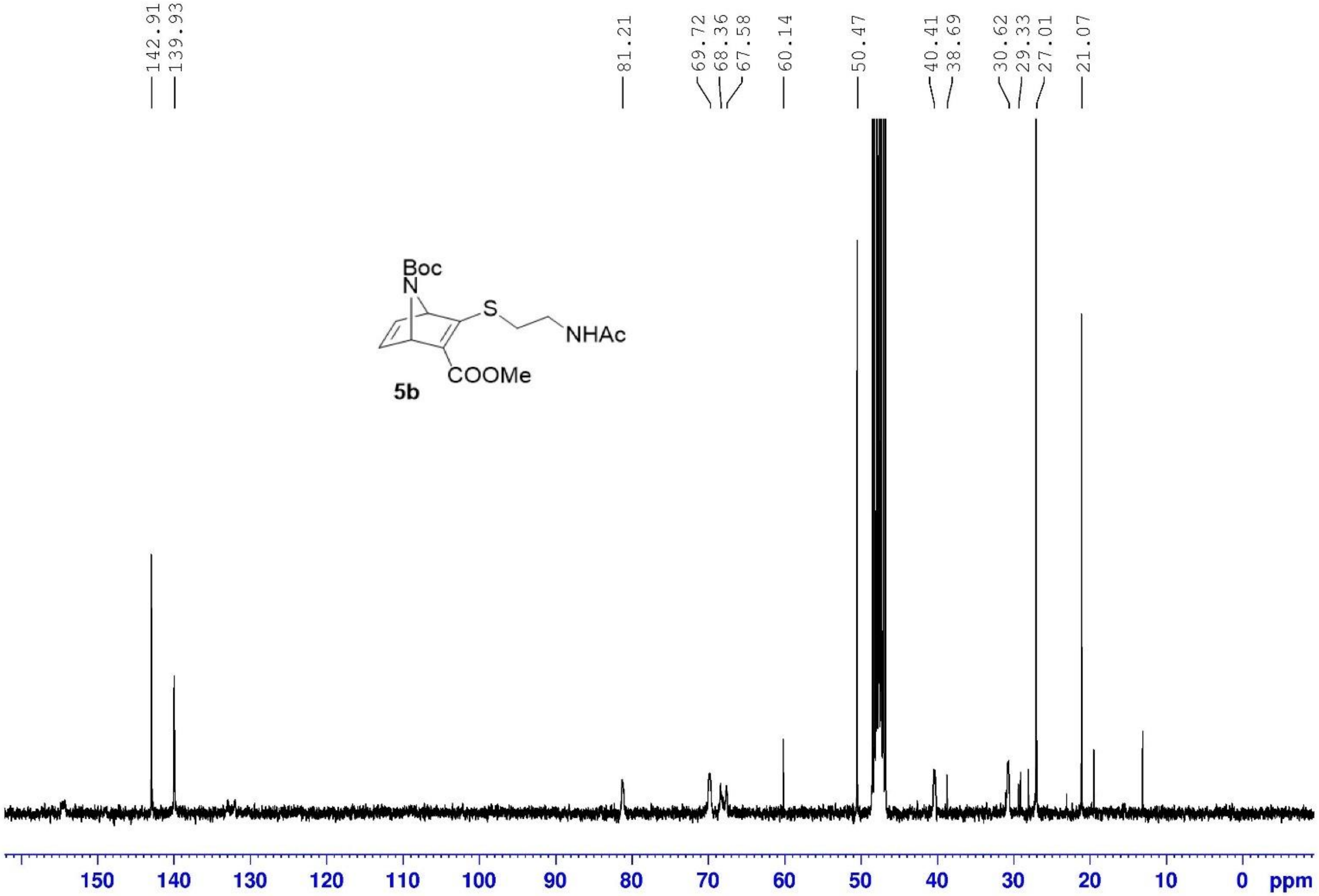

^{13}C-NMR (CD_3OD, 300 MHz) of compound **5b**

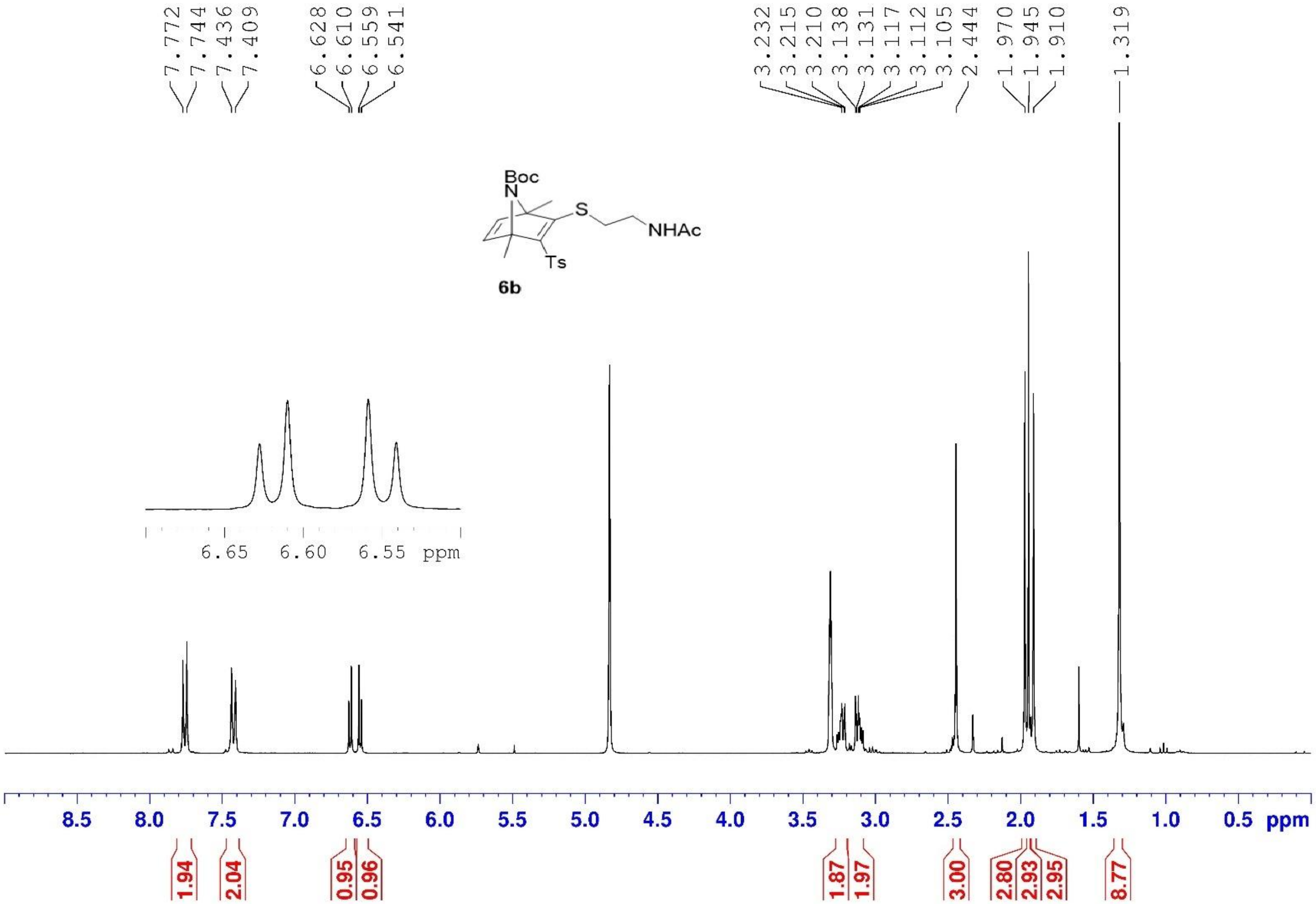

^{1}H-NMR (CD_3OD, 300 MHz) of compound **6b**

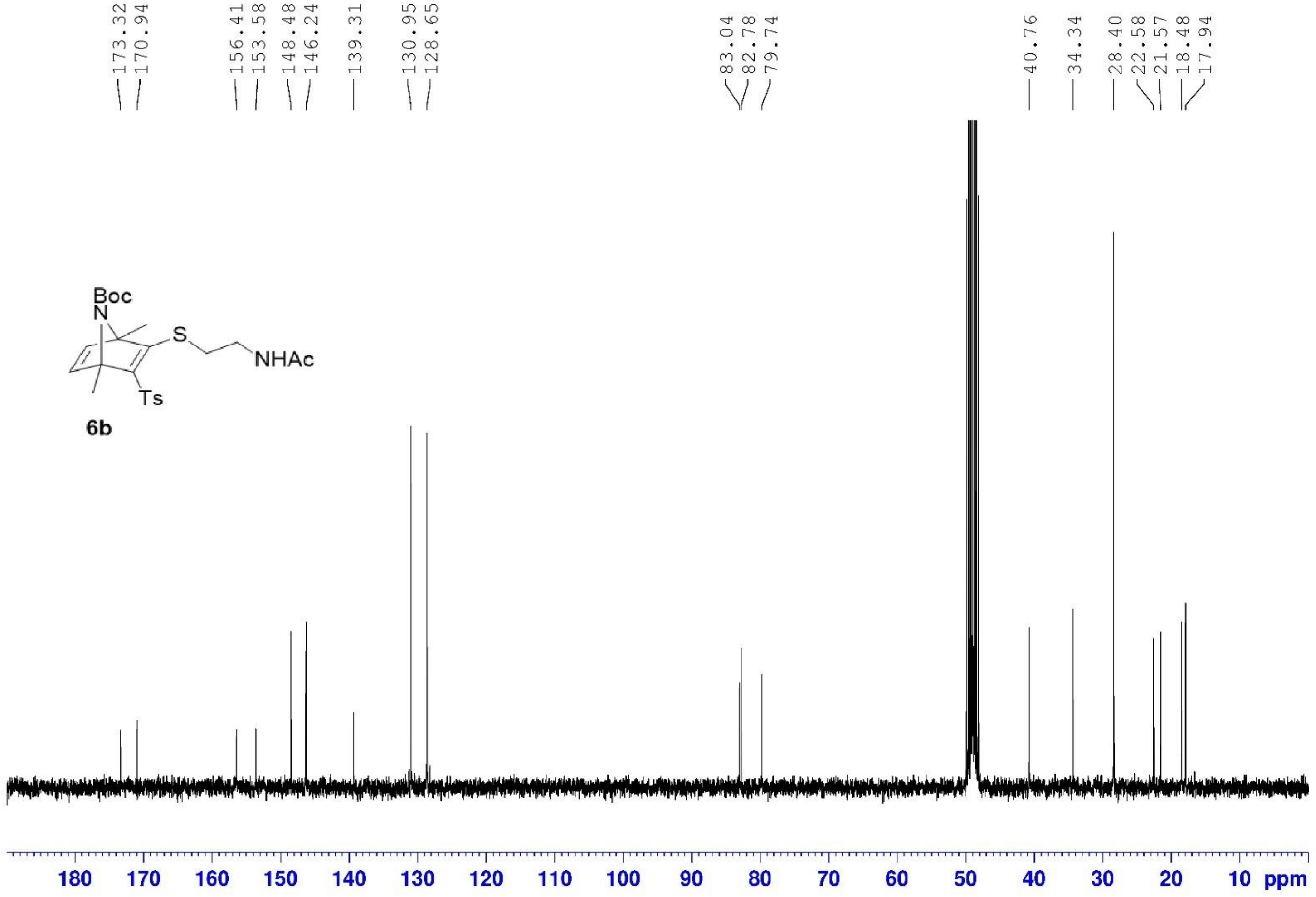

^{13}C-NMR (CD_3OD, 75MHz) of compound **6b**

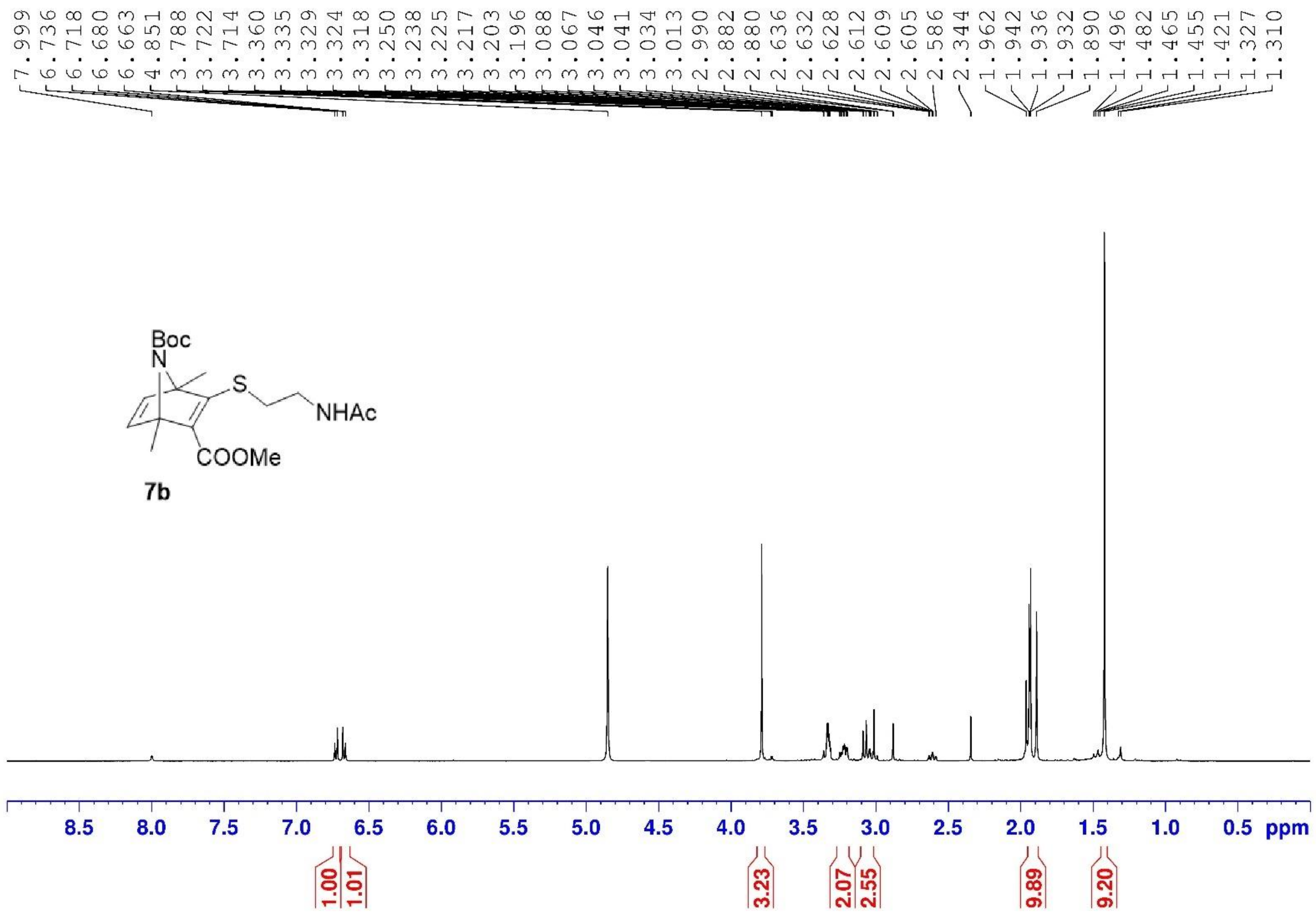

^{1}H-NMR (CD_3OD, 300 MHz) of compound **7b**

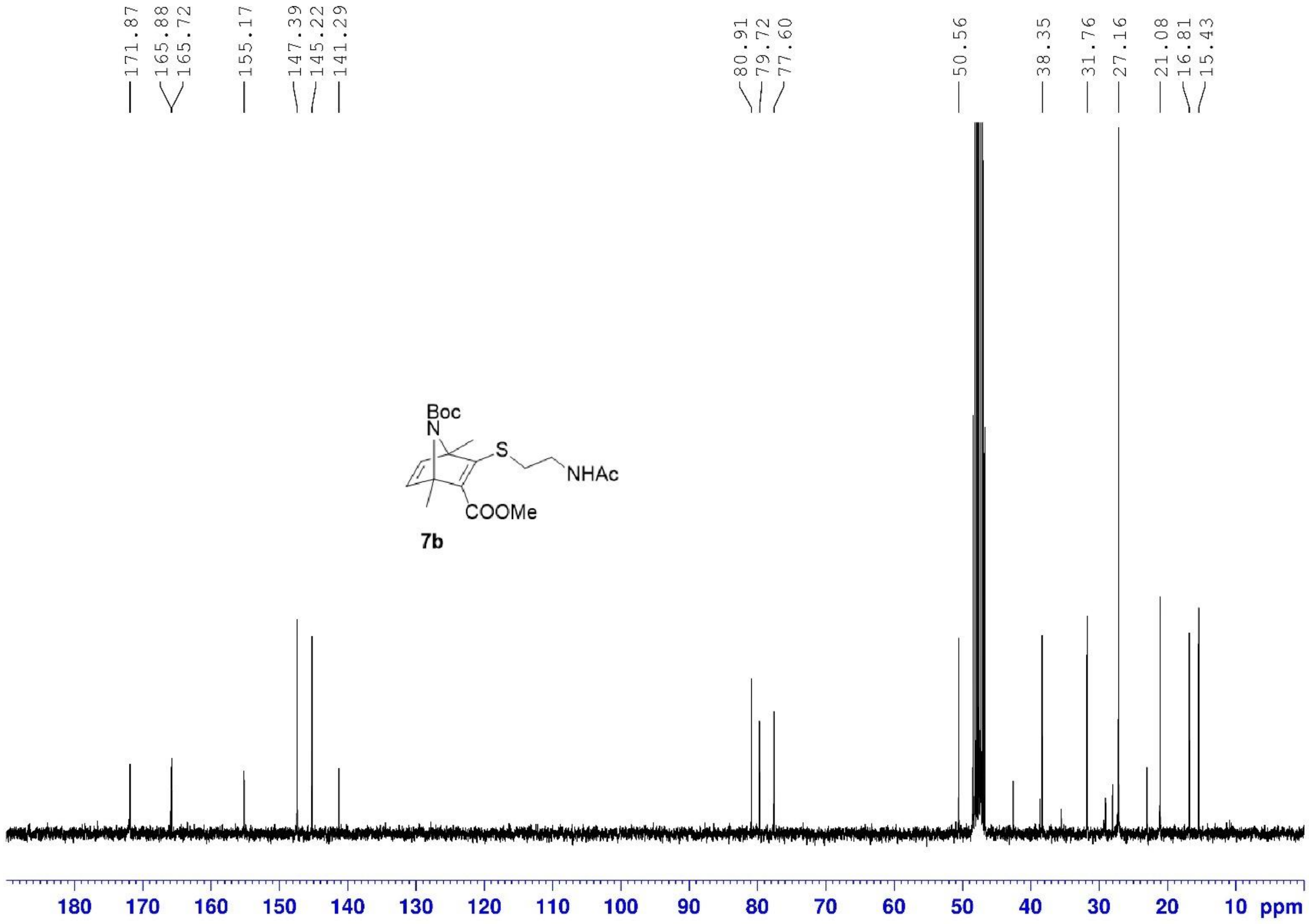

^{13}C-NMR (CD_3OD, 75 MHz) of compound **7b**

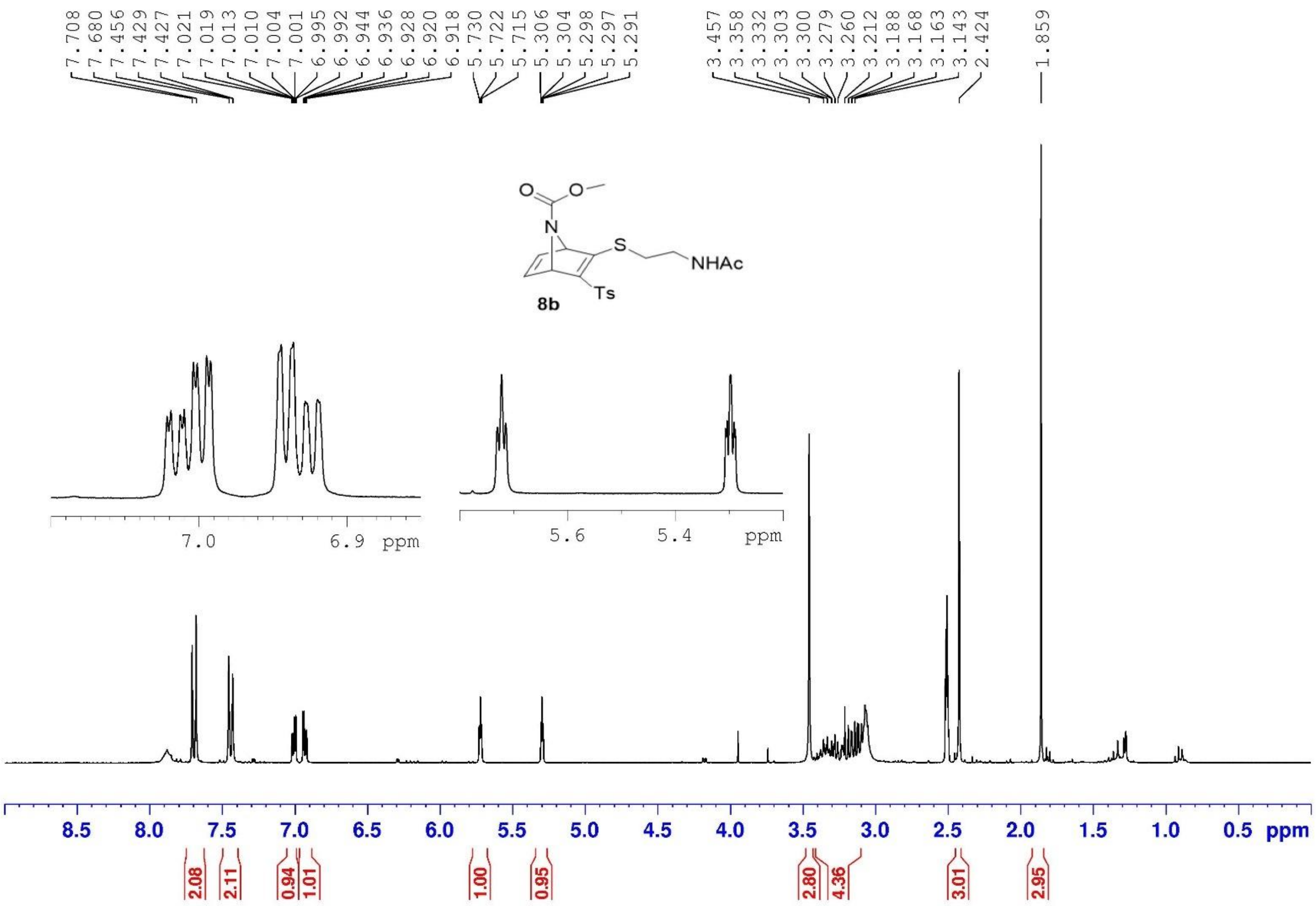

^{1}H-NMR ($(CD_3)_2SO$, 300 MHz, 363 K) of compound **8b**

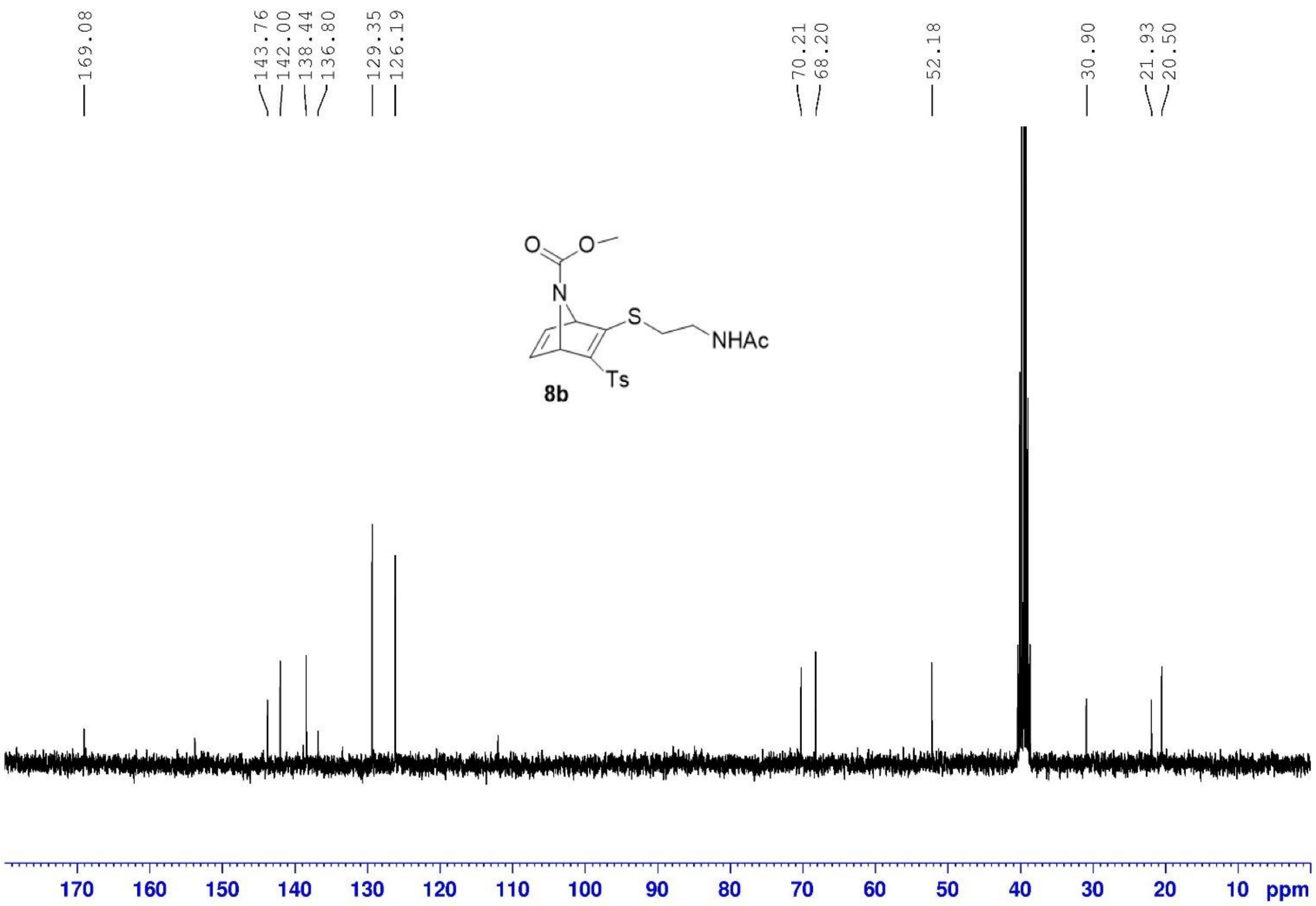

^{13}C-NMR $(CD_3)_2SO$, 75 MHz, 363 K) of compound **8b**

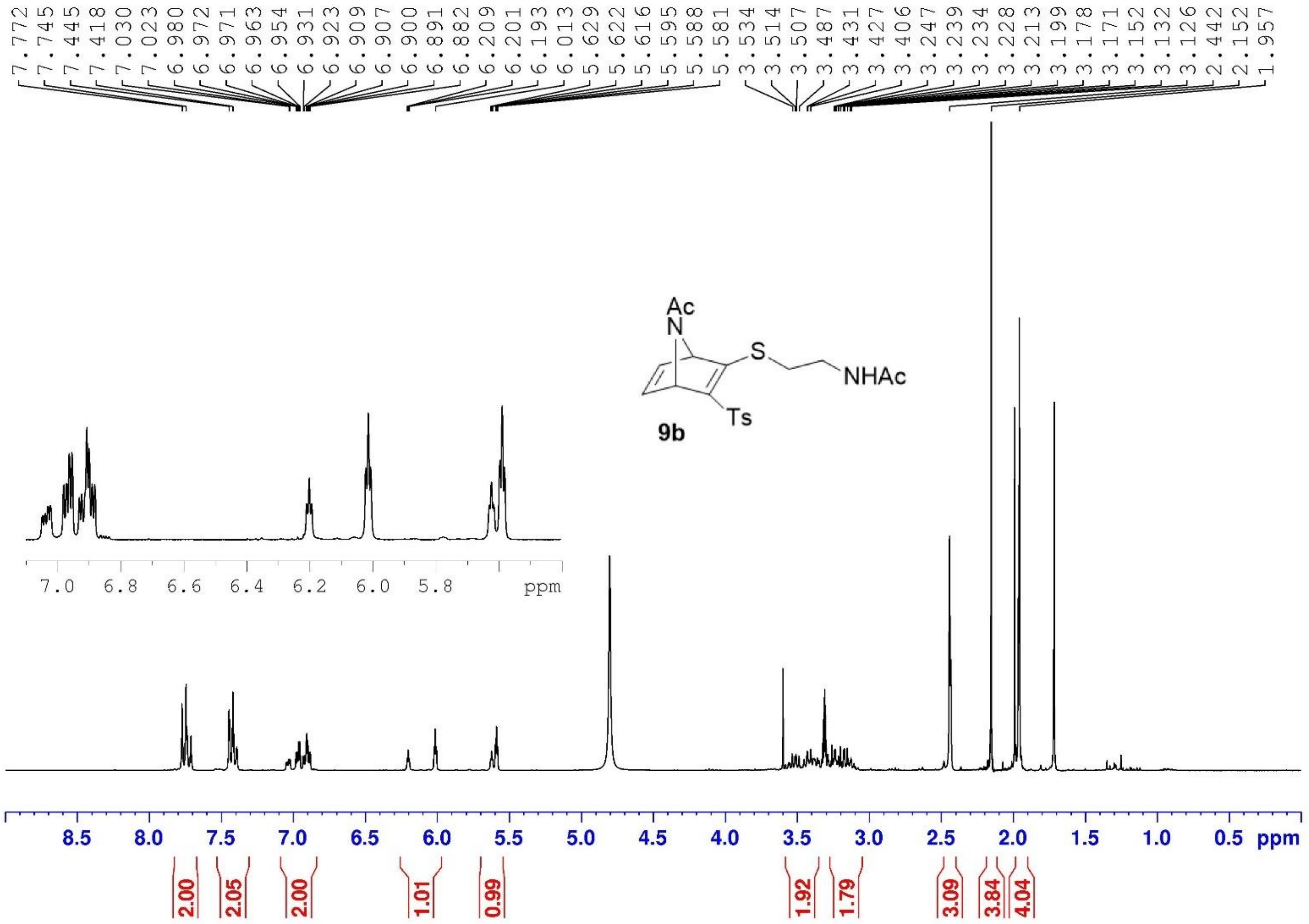

^{1}H-NMR (CD_3OD, 300 MHz) of compound **9b**

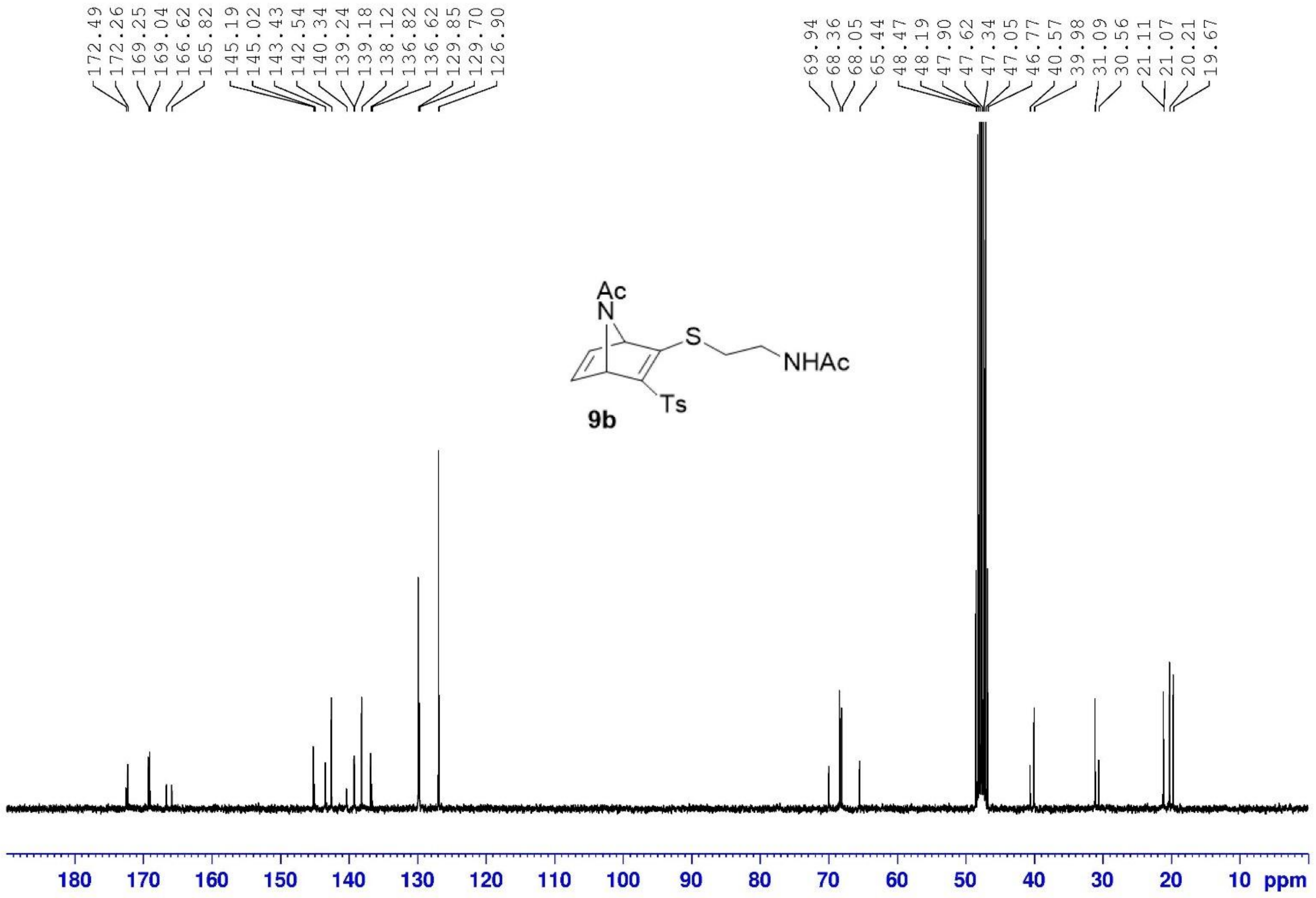

^{13}C-NMR (CD_3OD, 75 MHz) of compound **9b**

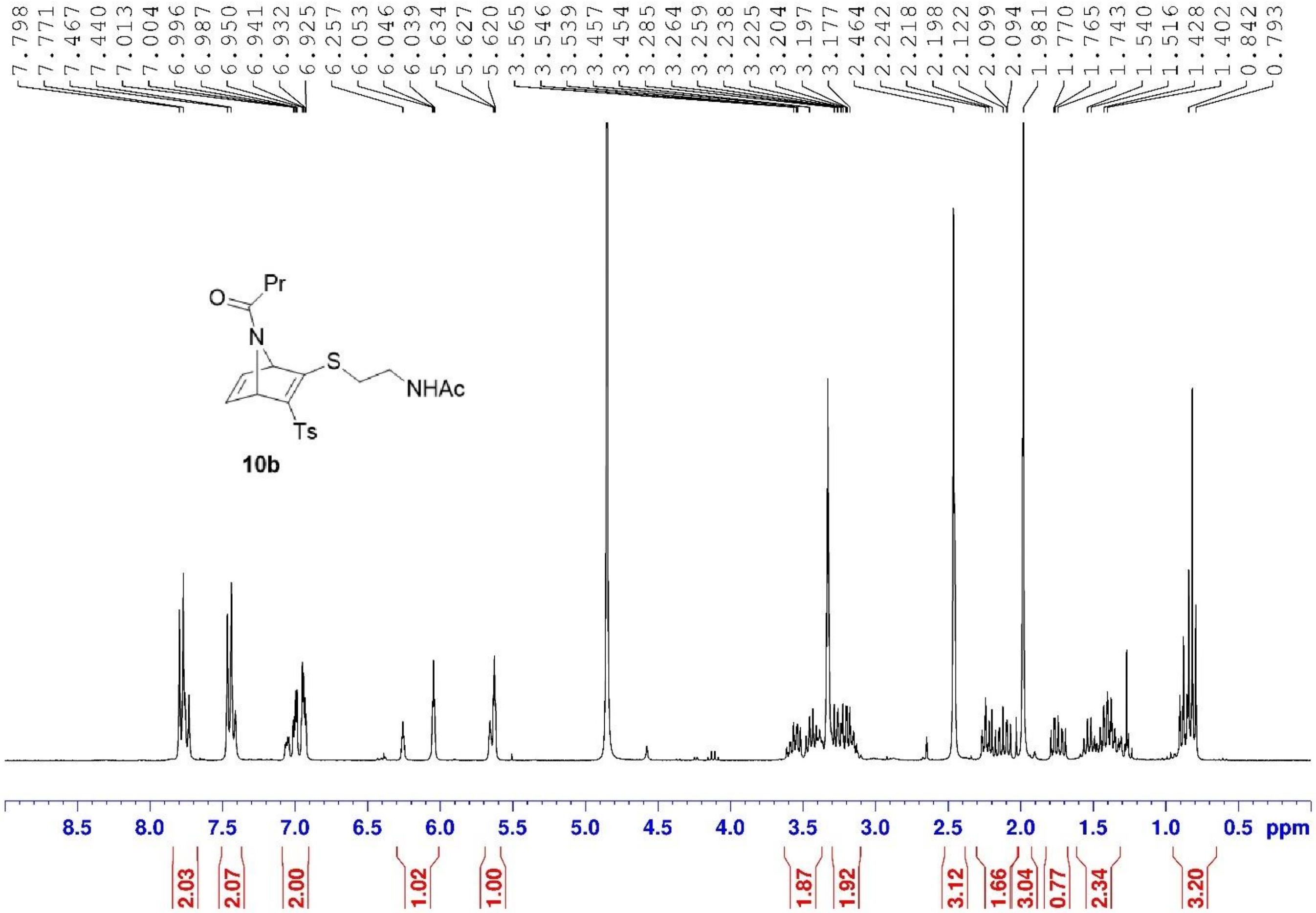

^{1}H-NMR (CD_3OD, 300 MHz) of compound **10b**

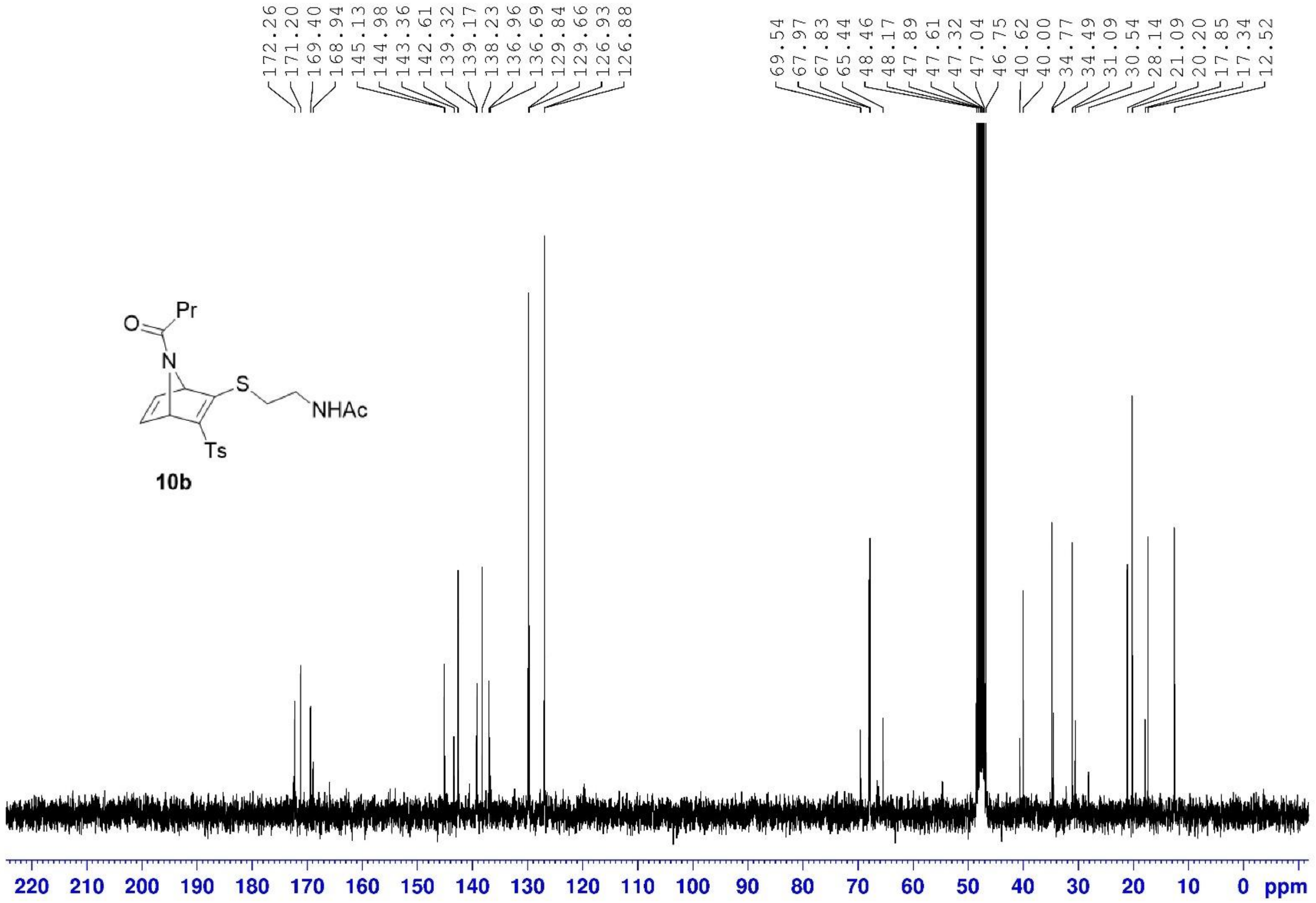

10b

^{13}C-NMR (CD_3OD, 75 MHz) of compound **10b**

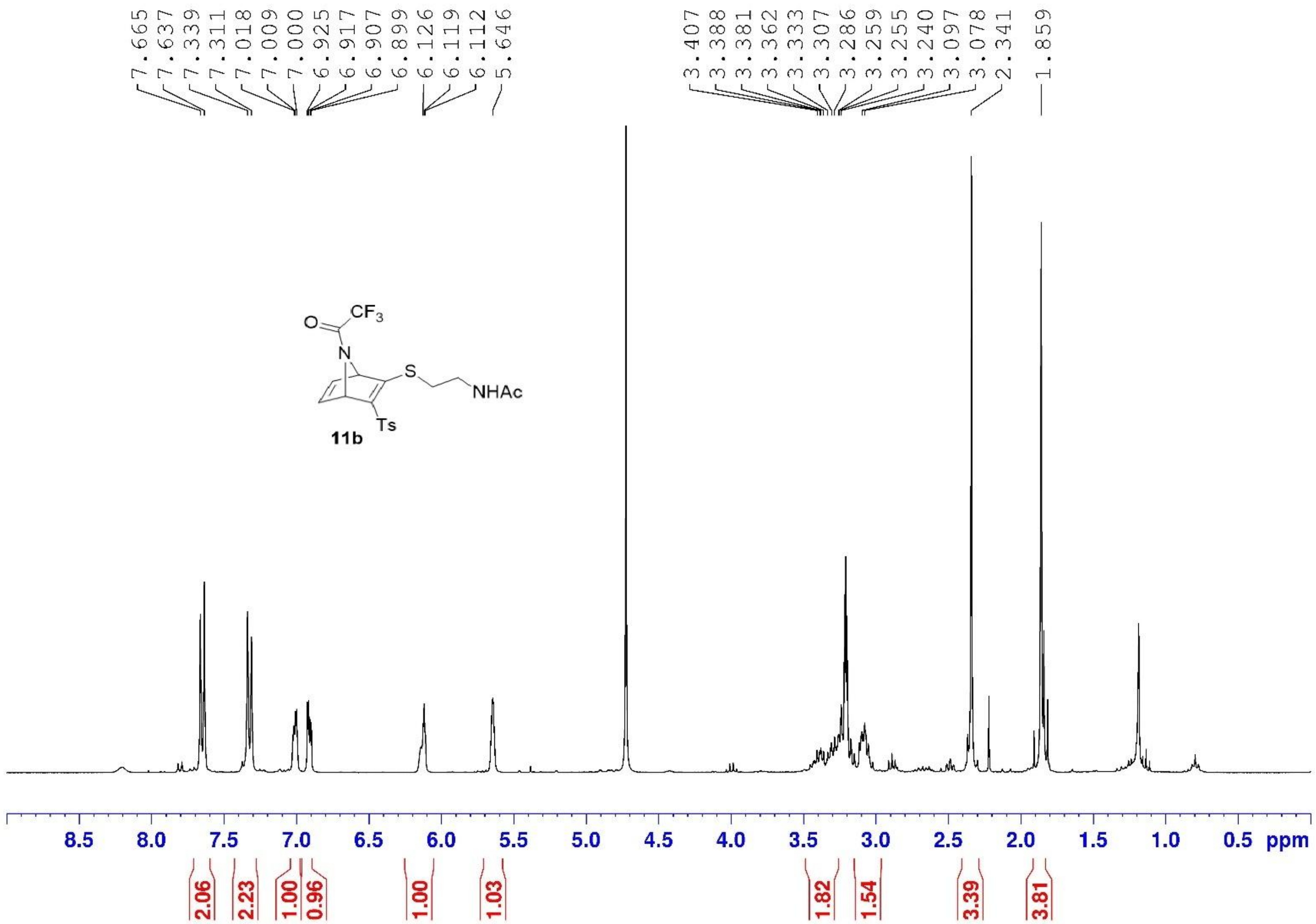

^{1}H-NMR (CD_3OD, 300 MHz) of compound **11b**

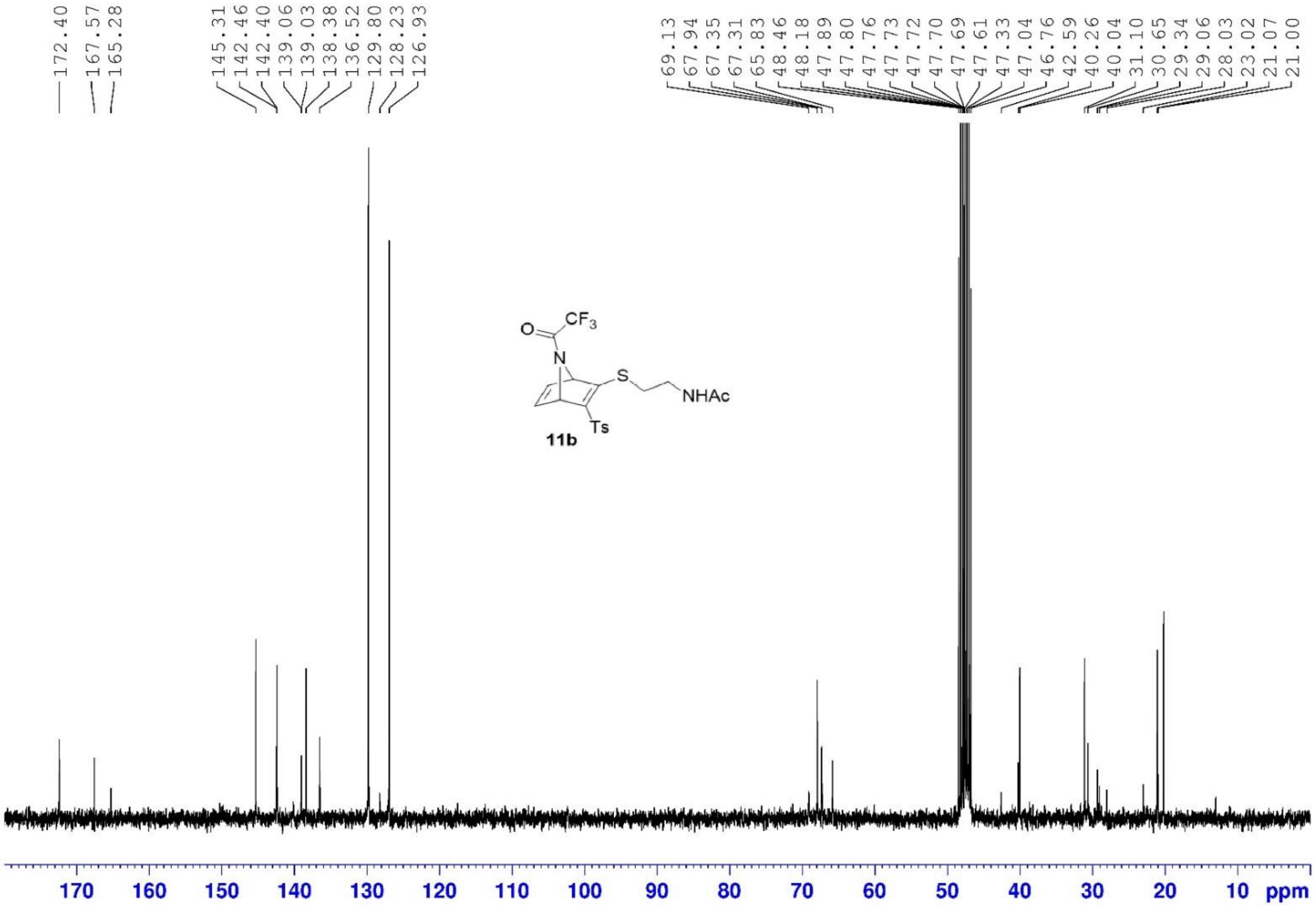

^{13}C-NMR (CD_3OD, 75 MHz) of compound **11b**

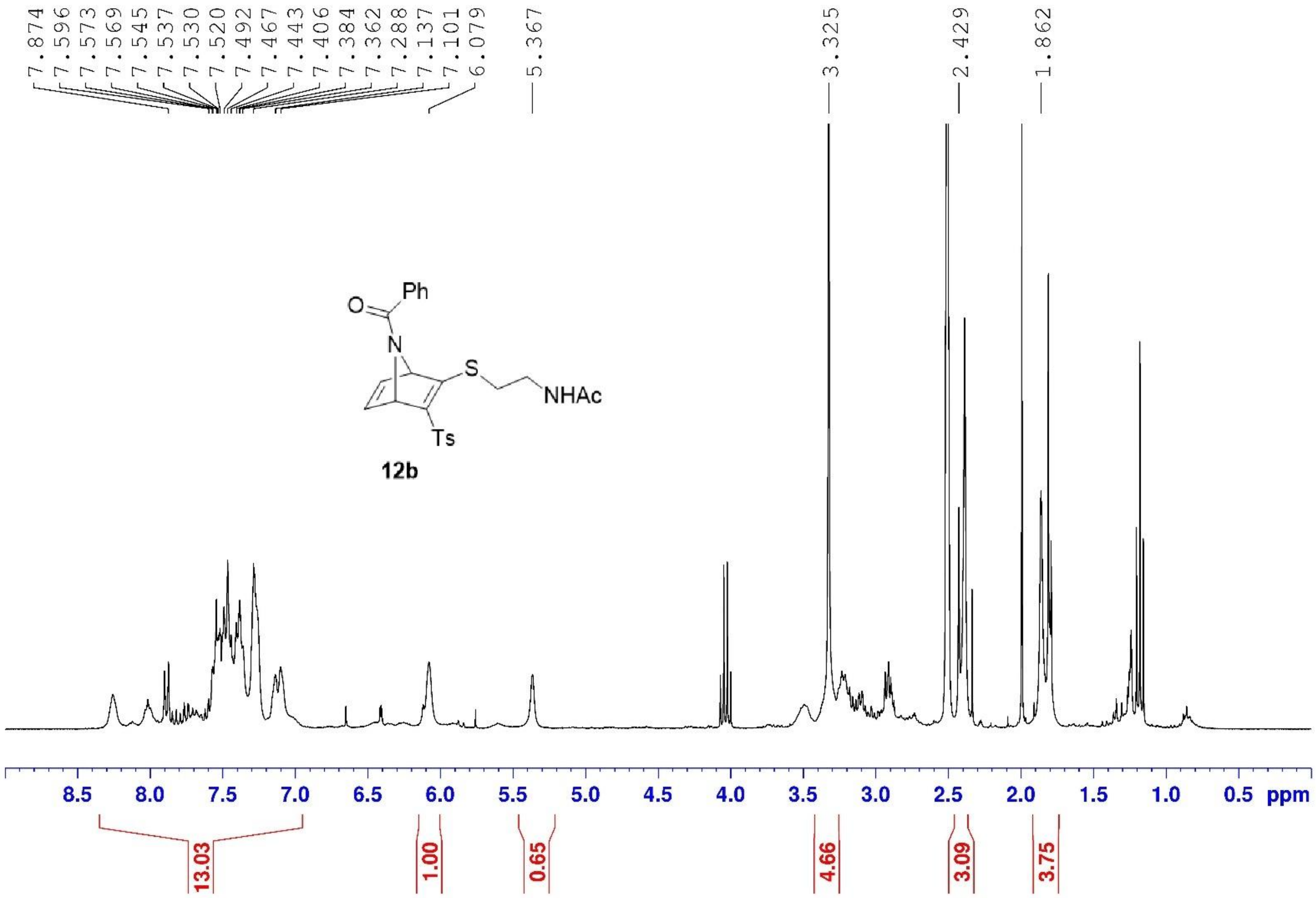

^{1}H-NMR ($(CD_3)_2SO$, 300 MHz) of compound **12b**

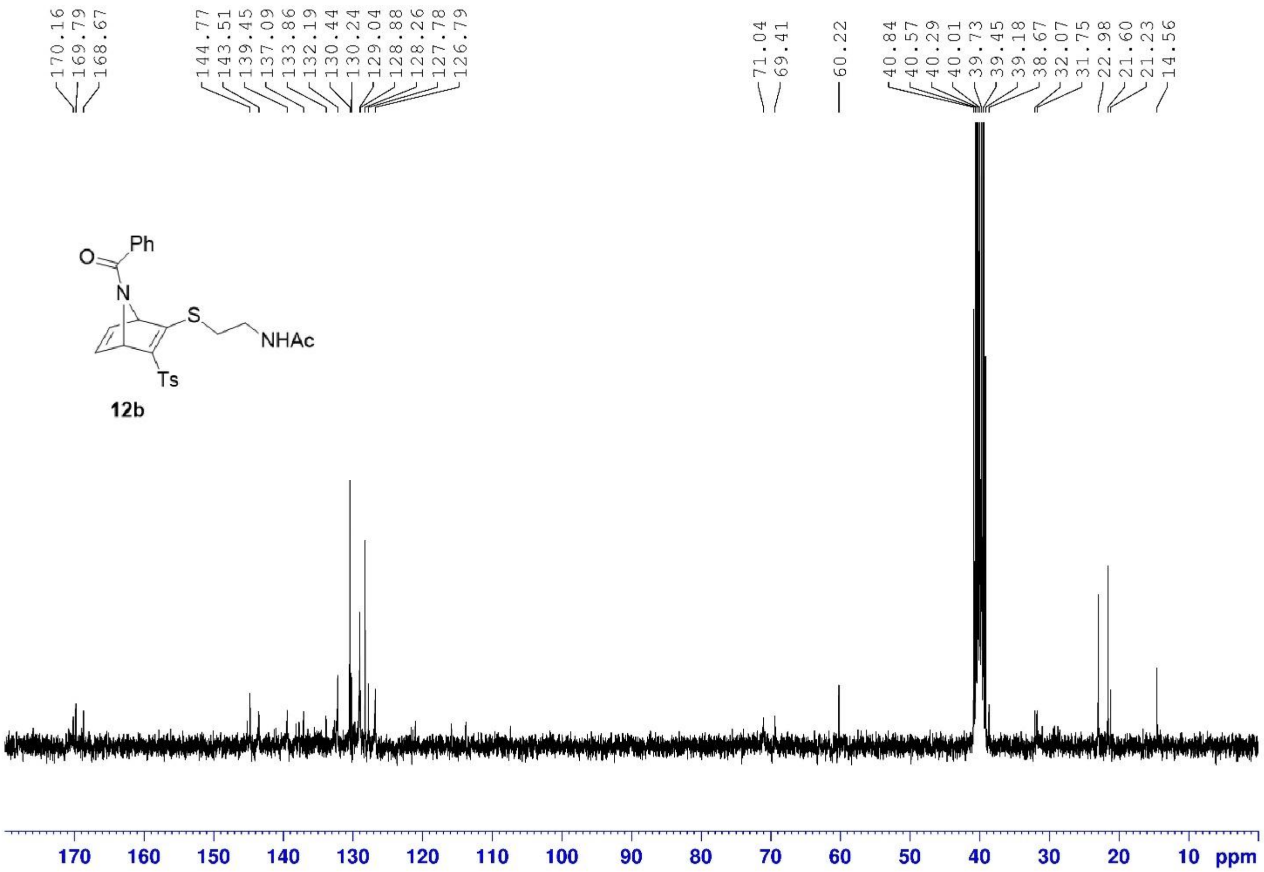

^{13}C-NMR ($(CD_3)_2SO$, 75 MHz) of compound **12b**

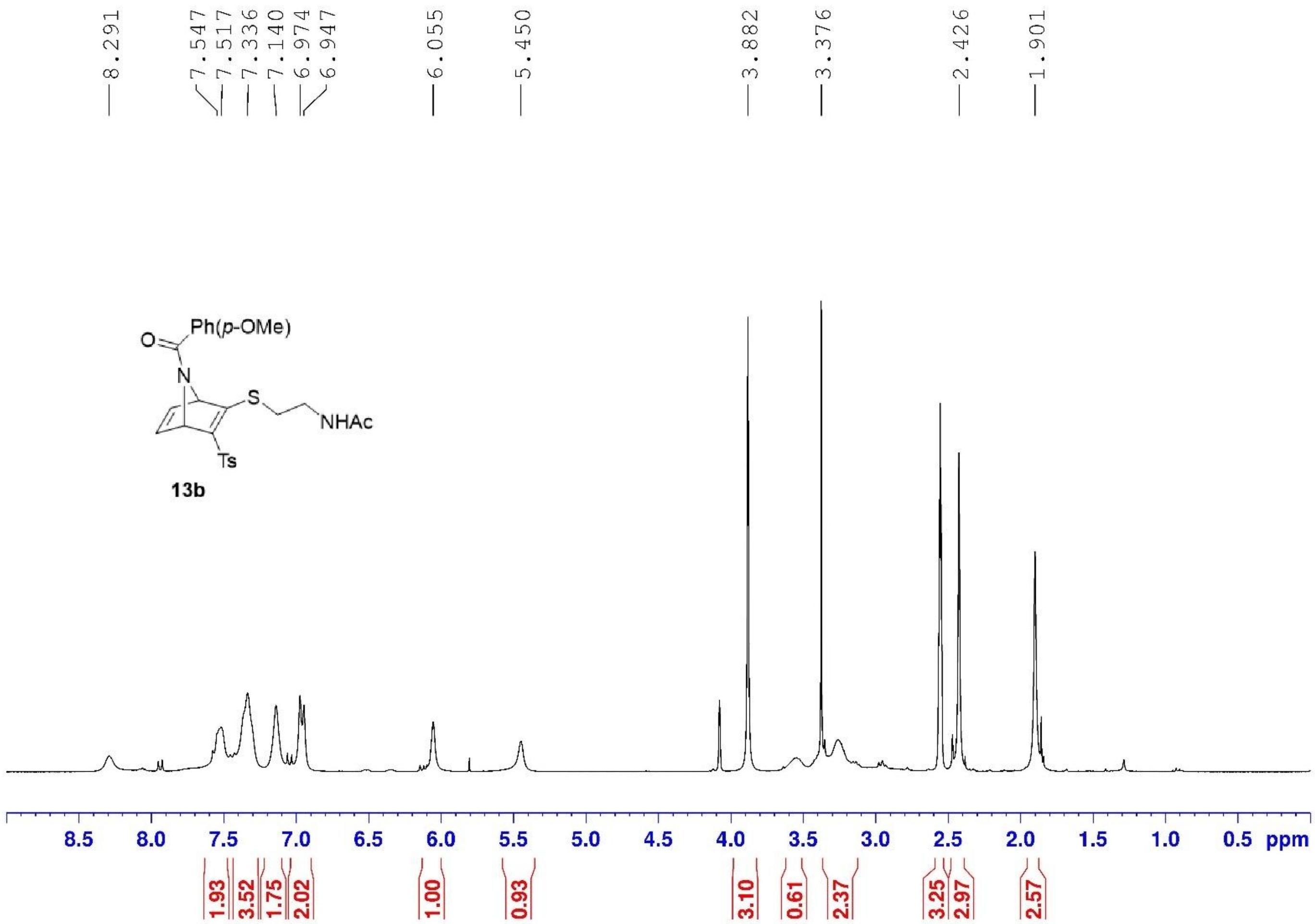

^{1}H-NMR ($(CD_3)_2SO$, 300 MHz) of compound **13b**

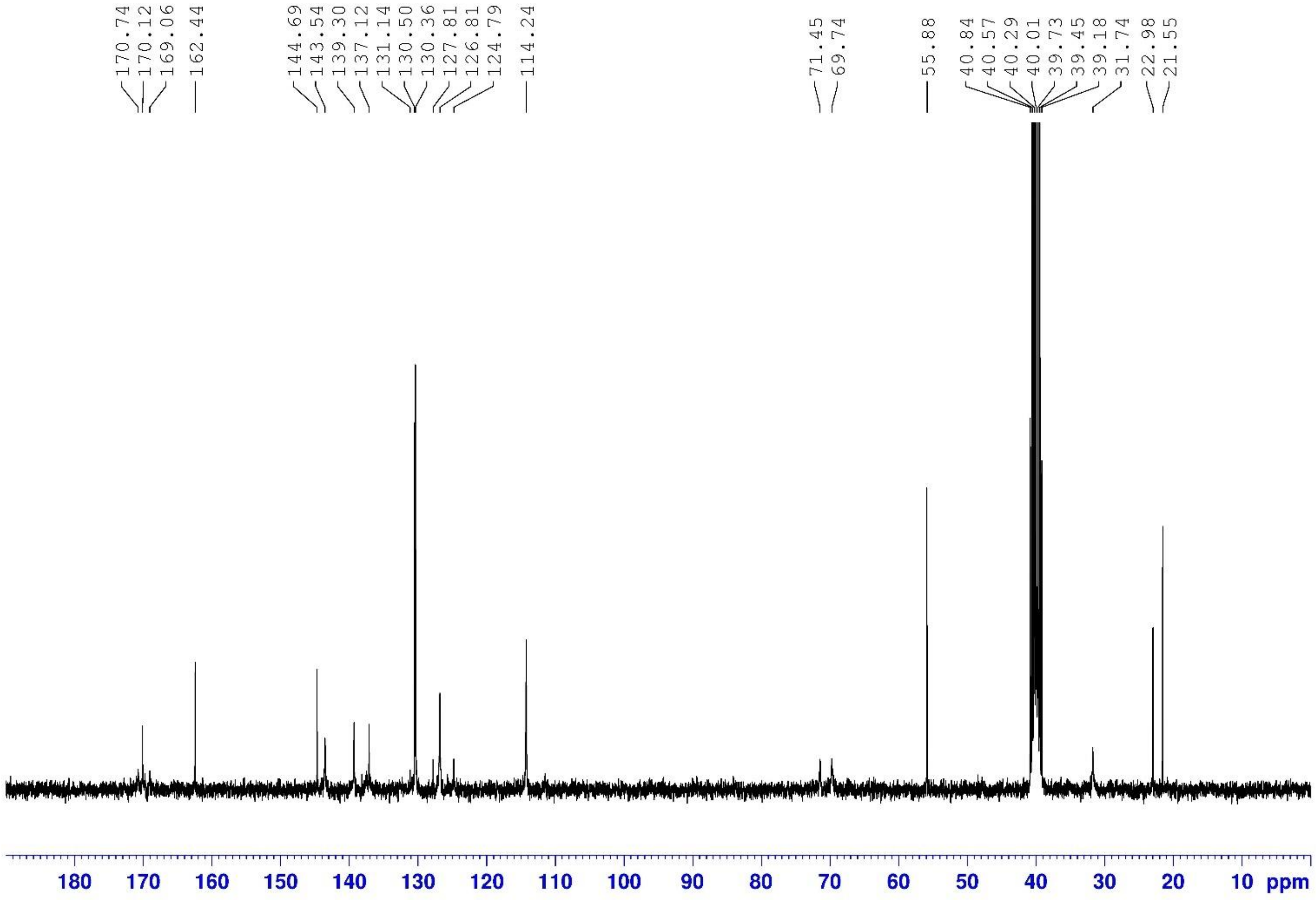

^{13}C-NMR ($(CD_3)_2SO$, 75 MHz) of compound **13b**

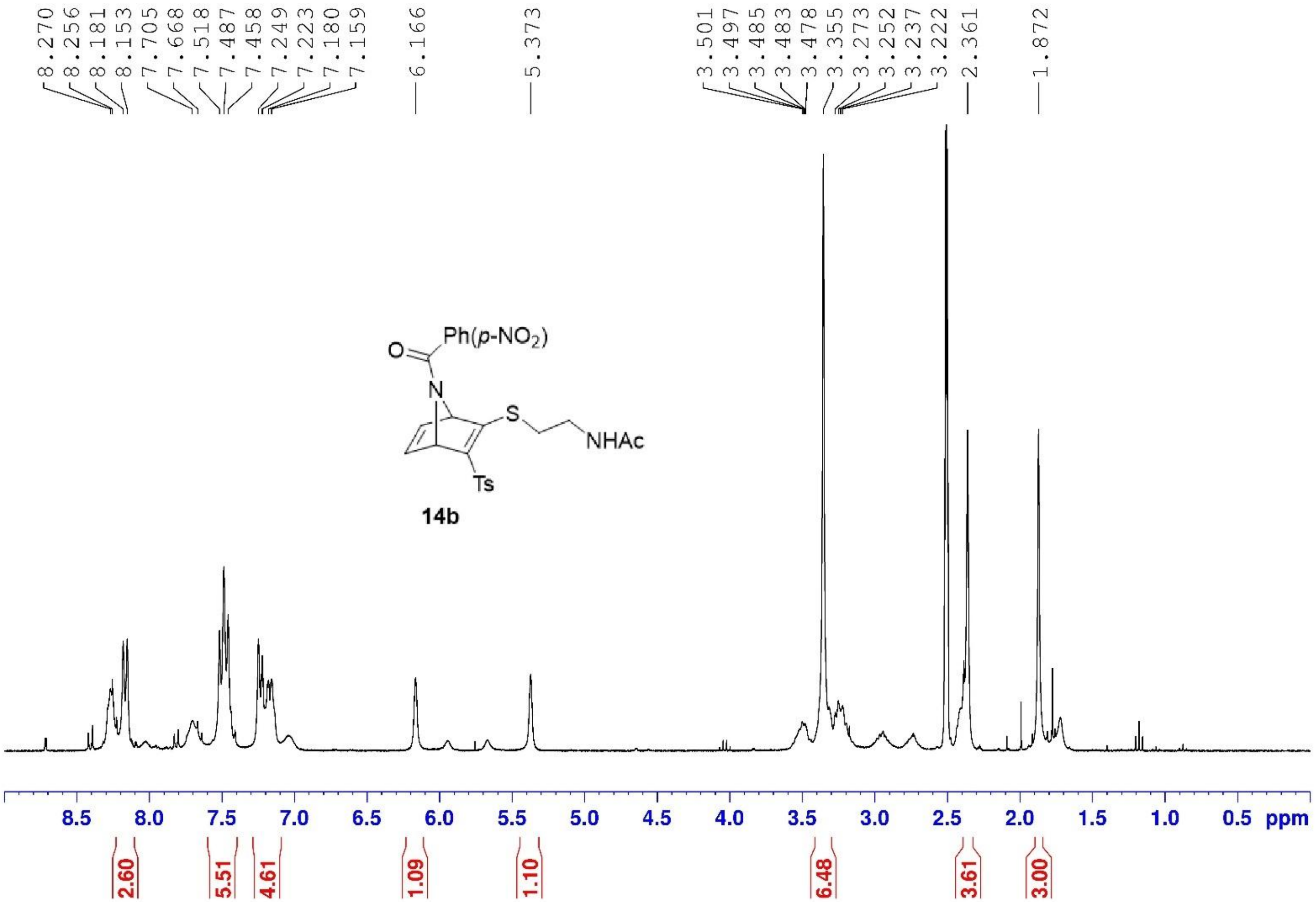

^{1}H-NMR (($(CD_3)_2SO$, 300 MHz) of compound **14b**

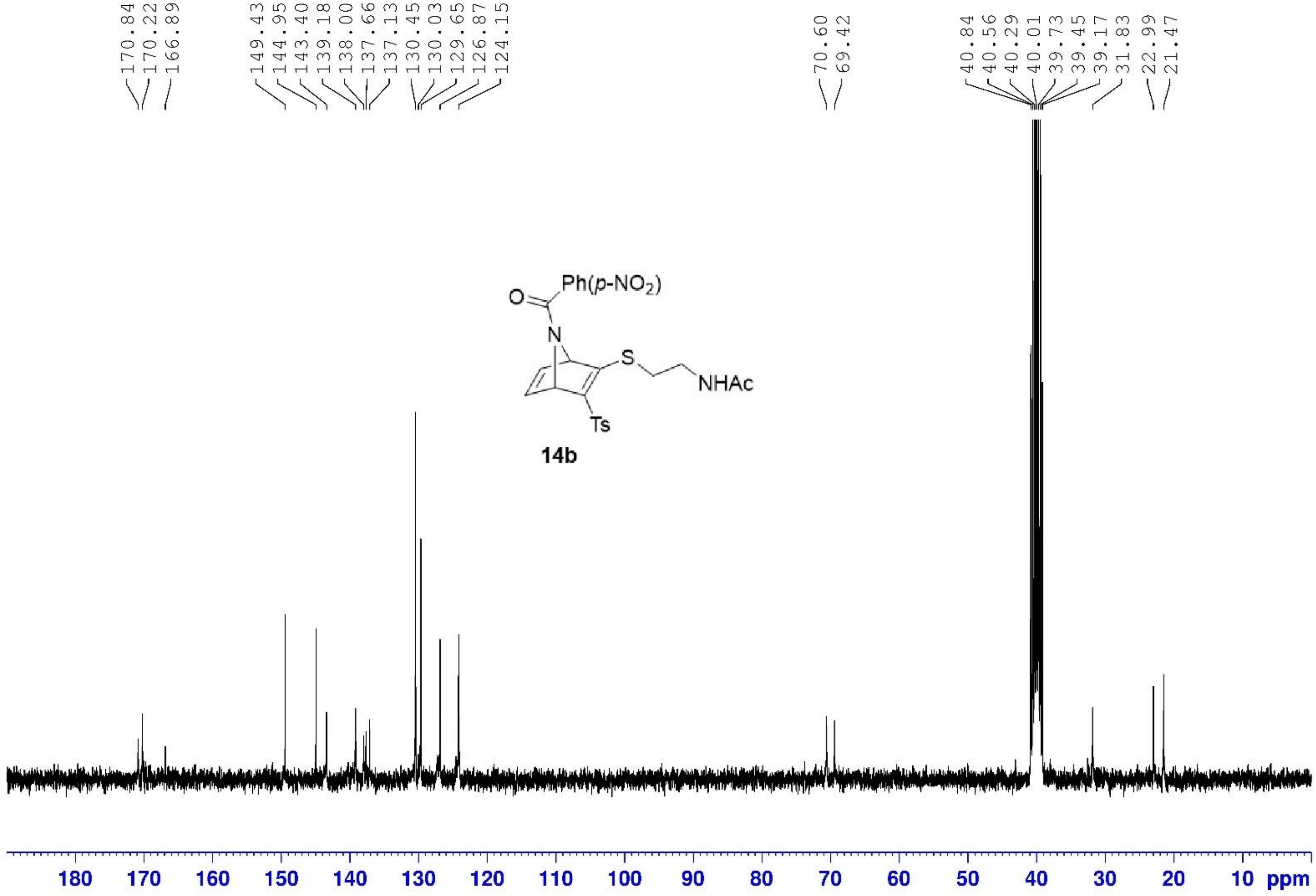

^{13}C-NMR ($(CD_3)_2SO$, 75 MHz) of compound **14b**

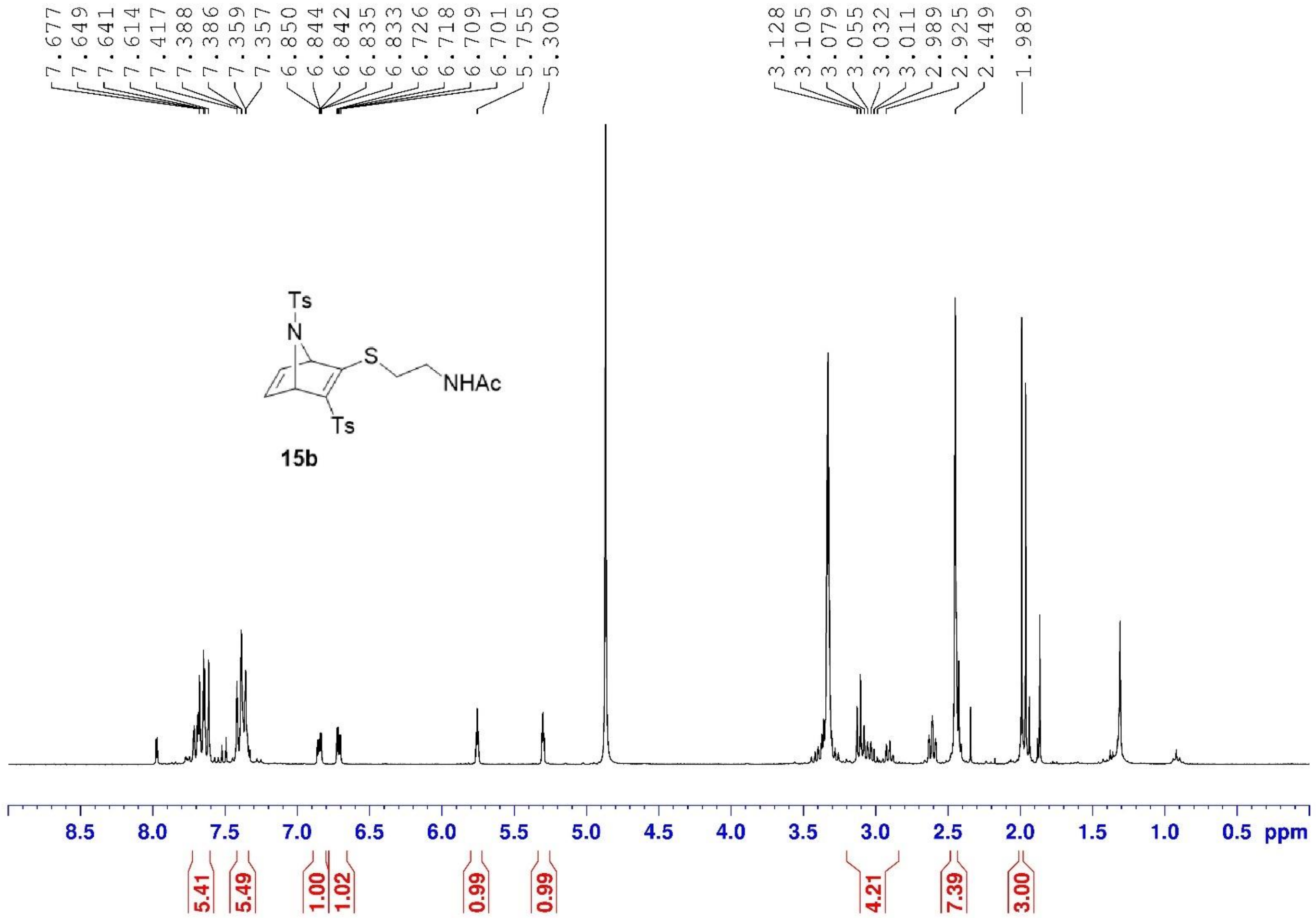

^{1}H-NMR (CD_3OD, 300 MHz) of compound **15b**

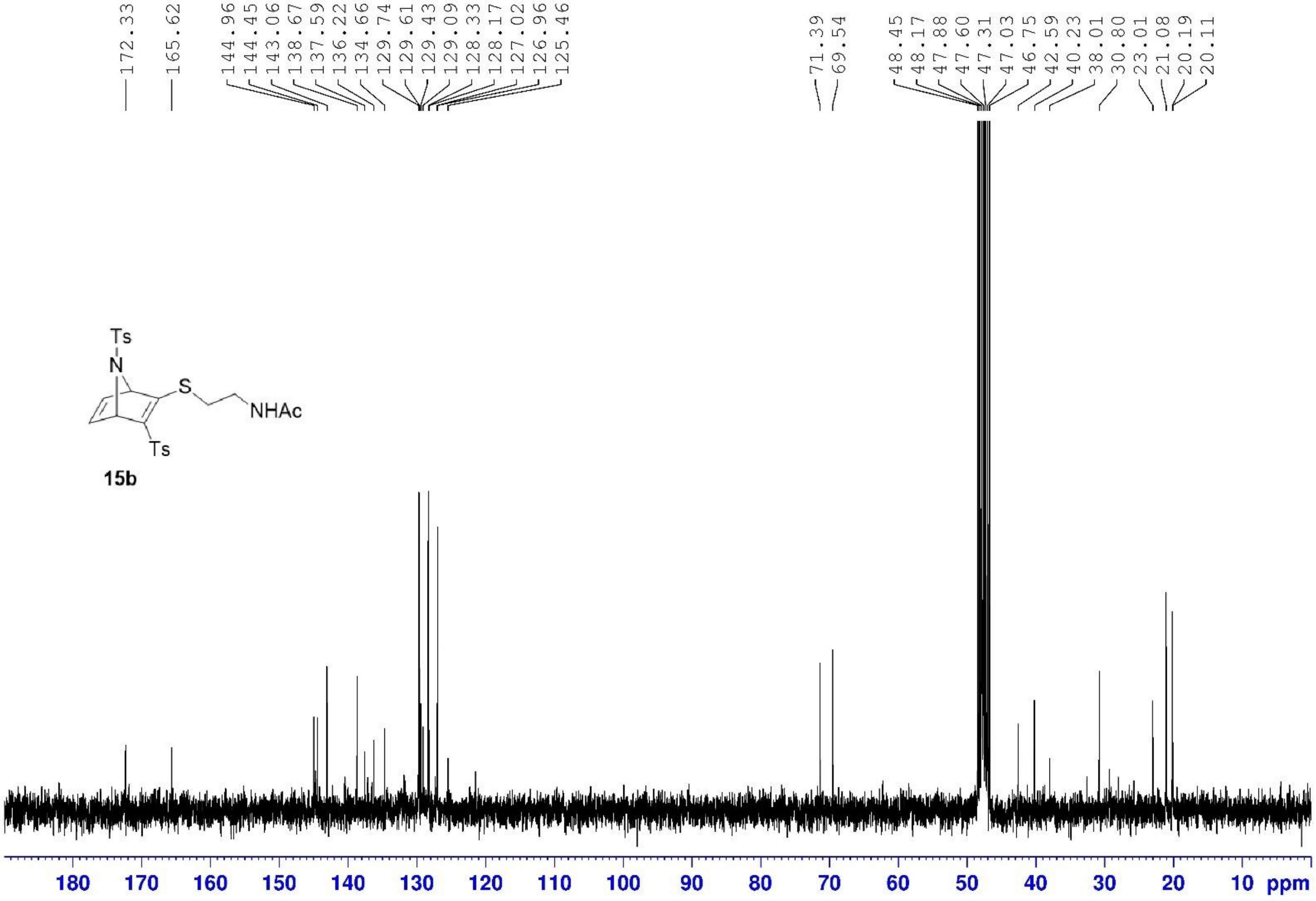

^{13}C-NMR (CD_3OD, 75 MHz) of compound **15b**

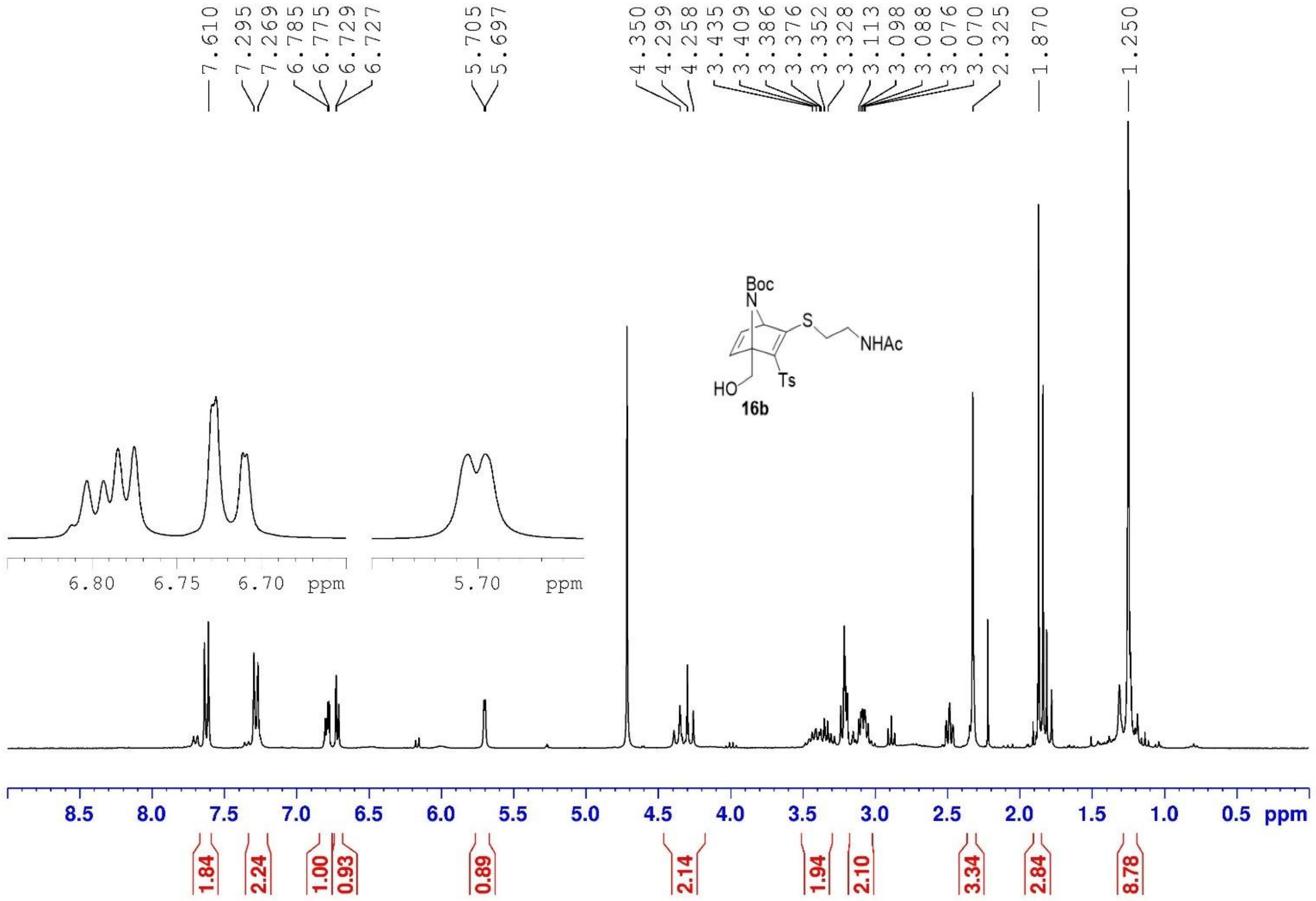

^{1}H-NMR (CD_3OD, 300 MHz) of compound **16b**

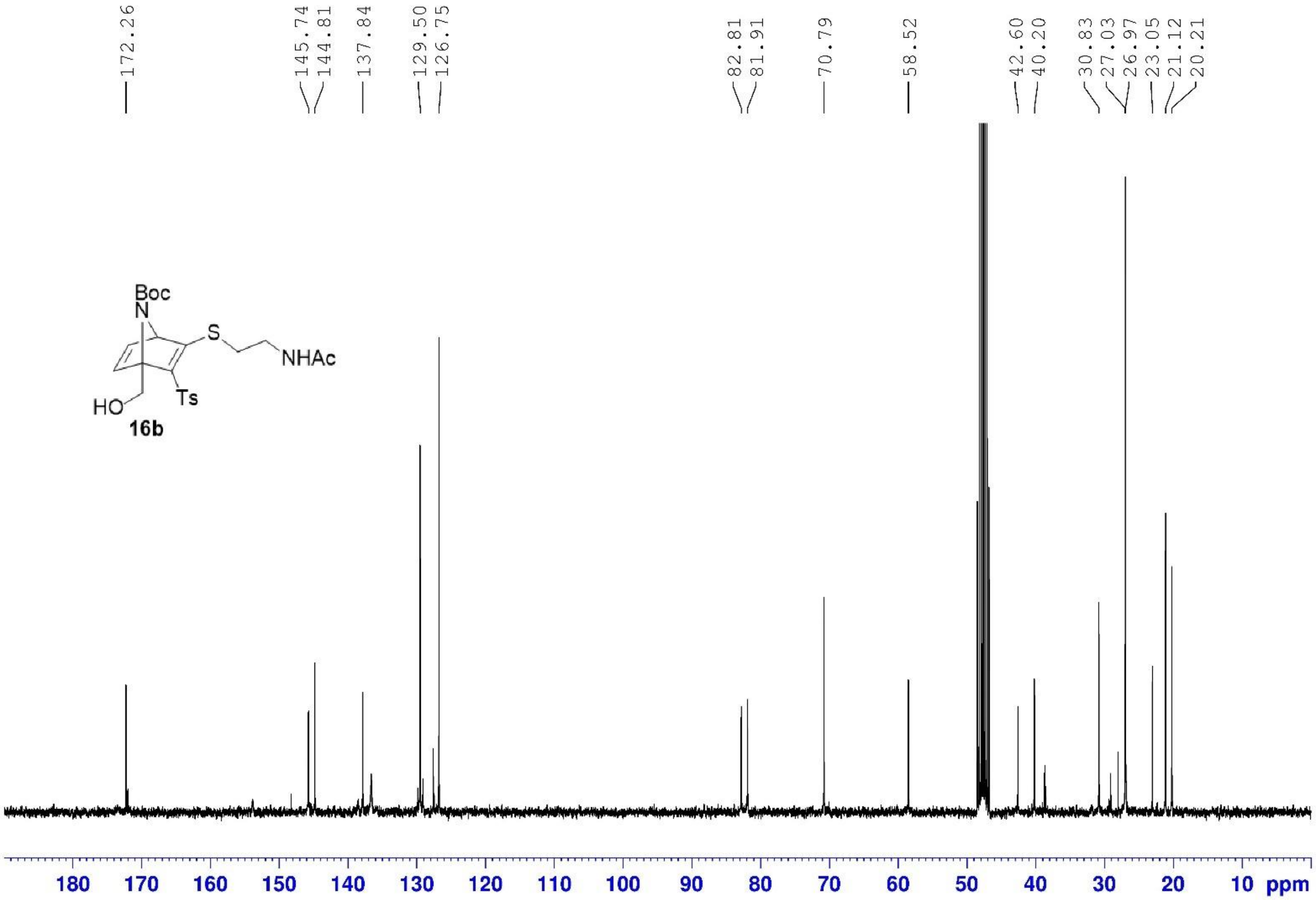

^{13}C-NMR (CD_3OD, 75 MHz) of compound **16b**

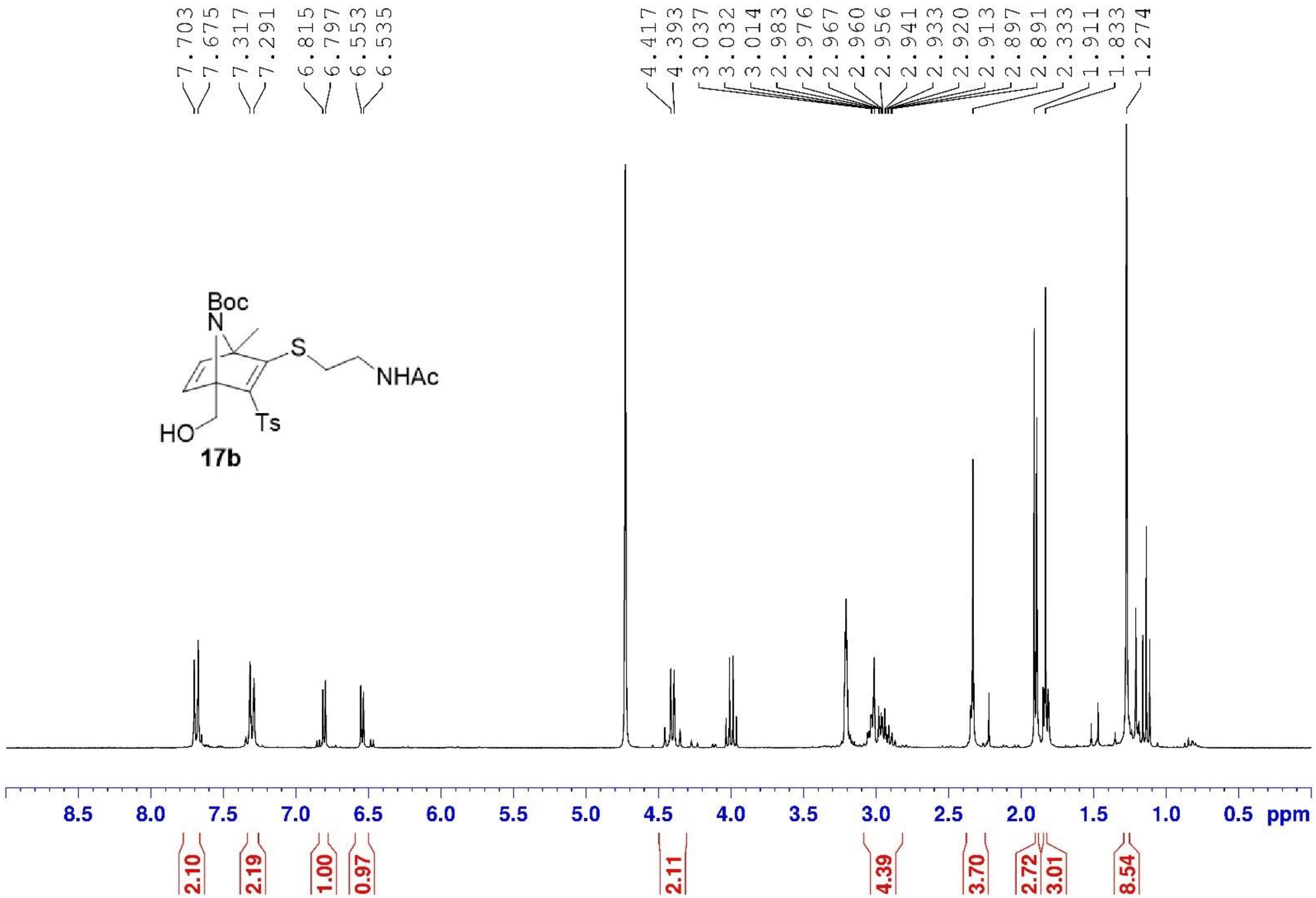

^{1}H-NMR (CD_3OD, 300 MHz) of compound **17b**

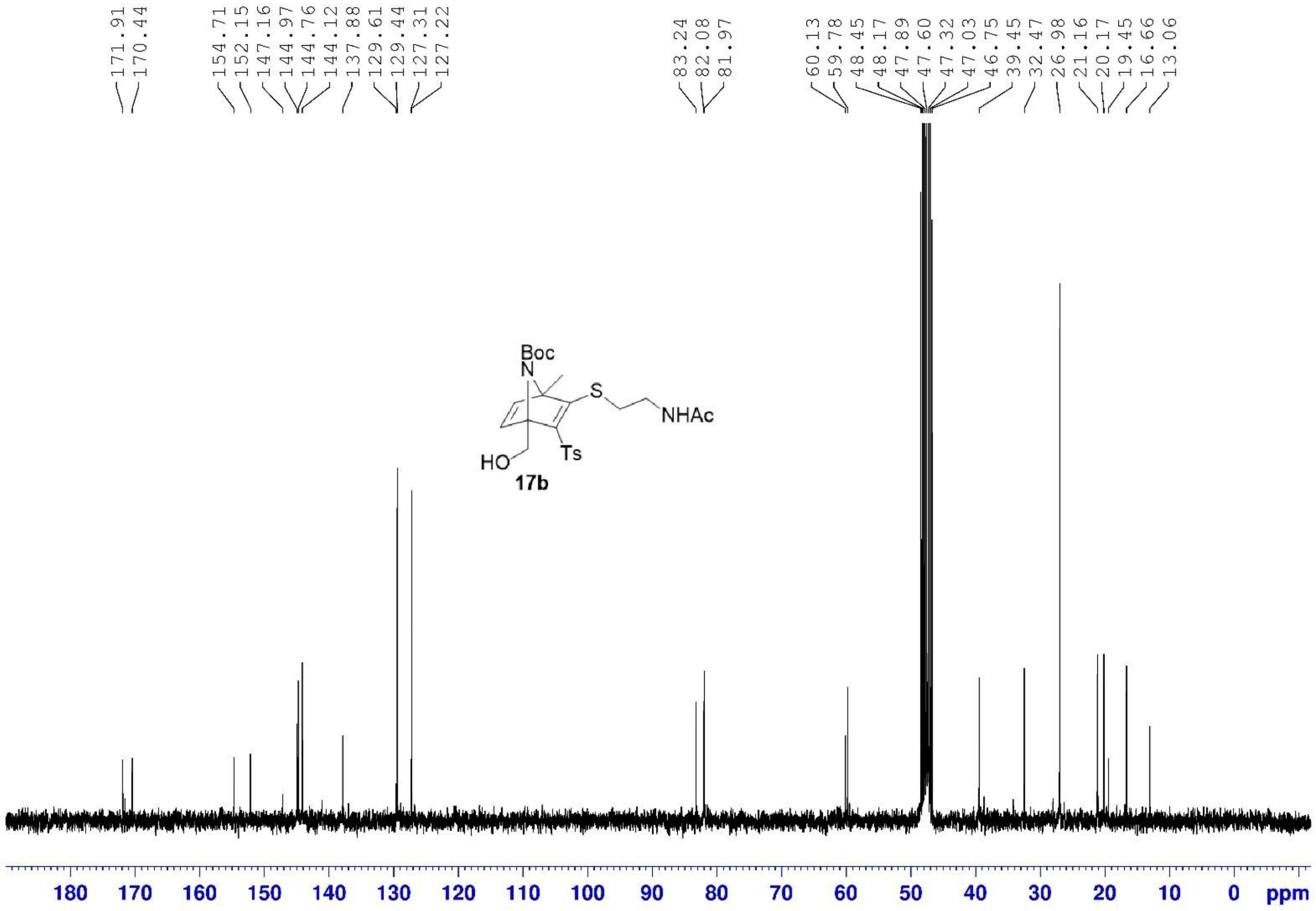

^{13}C-NMR (CD_3OD, 75 MHz) of compound **17b**

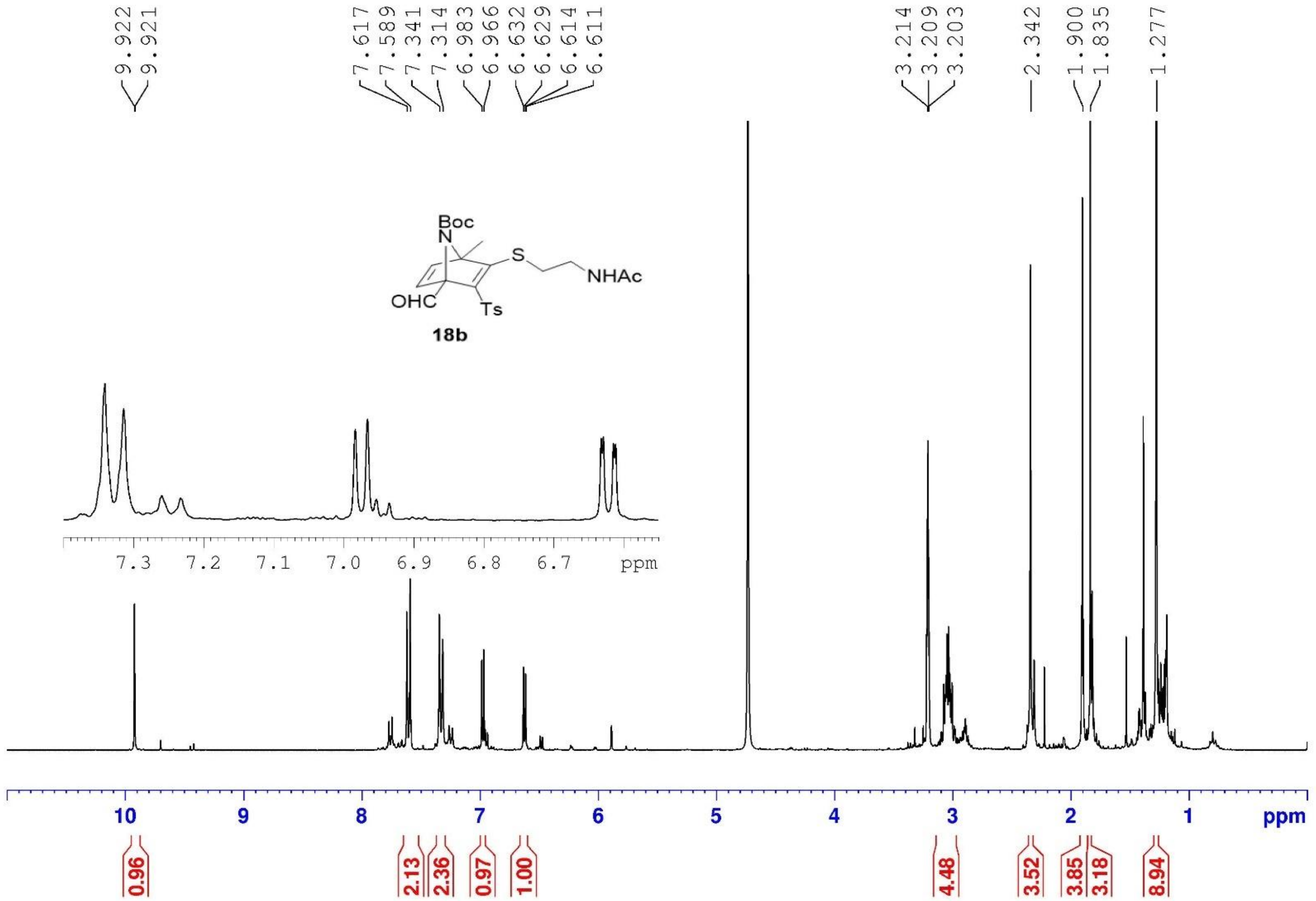

^{1}H-NMR (CD_3OD, 300 MHz) of compound **18b**

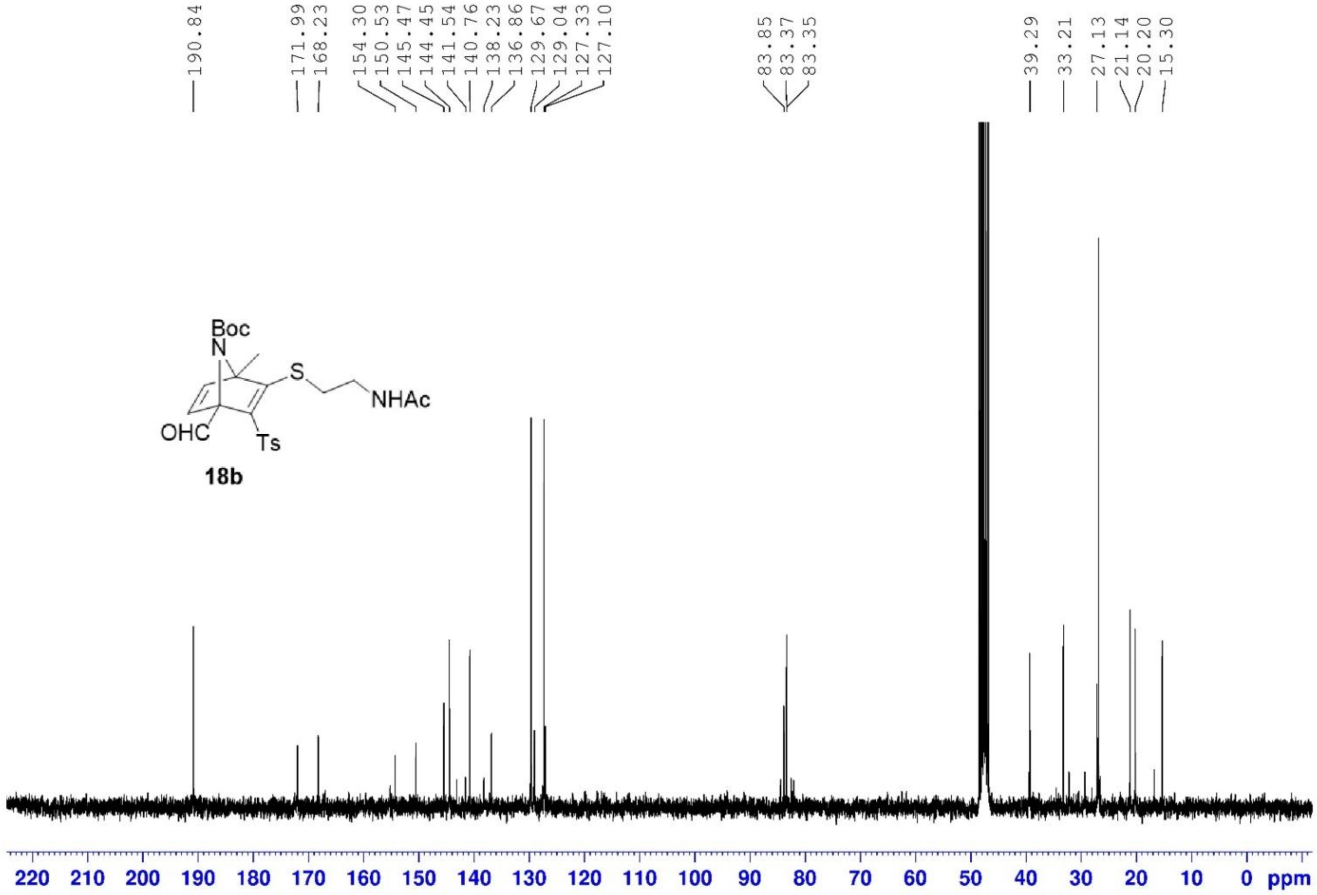

^{13}C-NMR (CD$_3$OD, 75 MHz) of compound **18b**

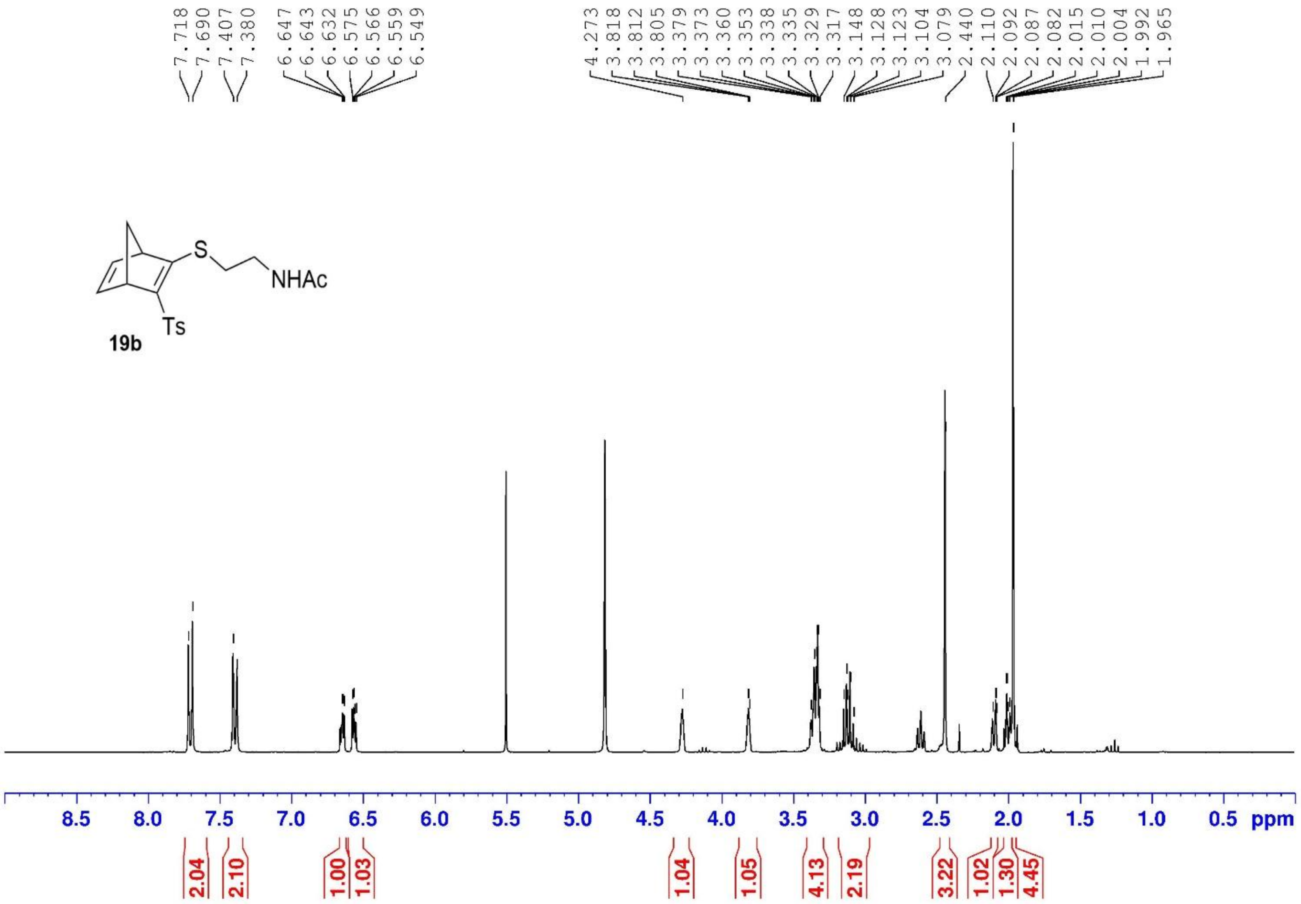

^{1}H-NMR (CD_3OD, 300 MHz) of compound **19b**

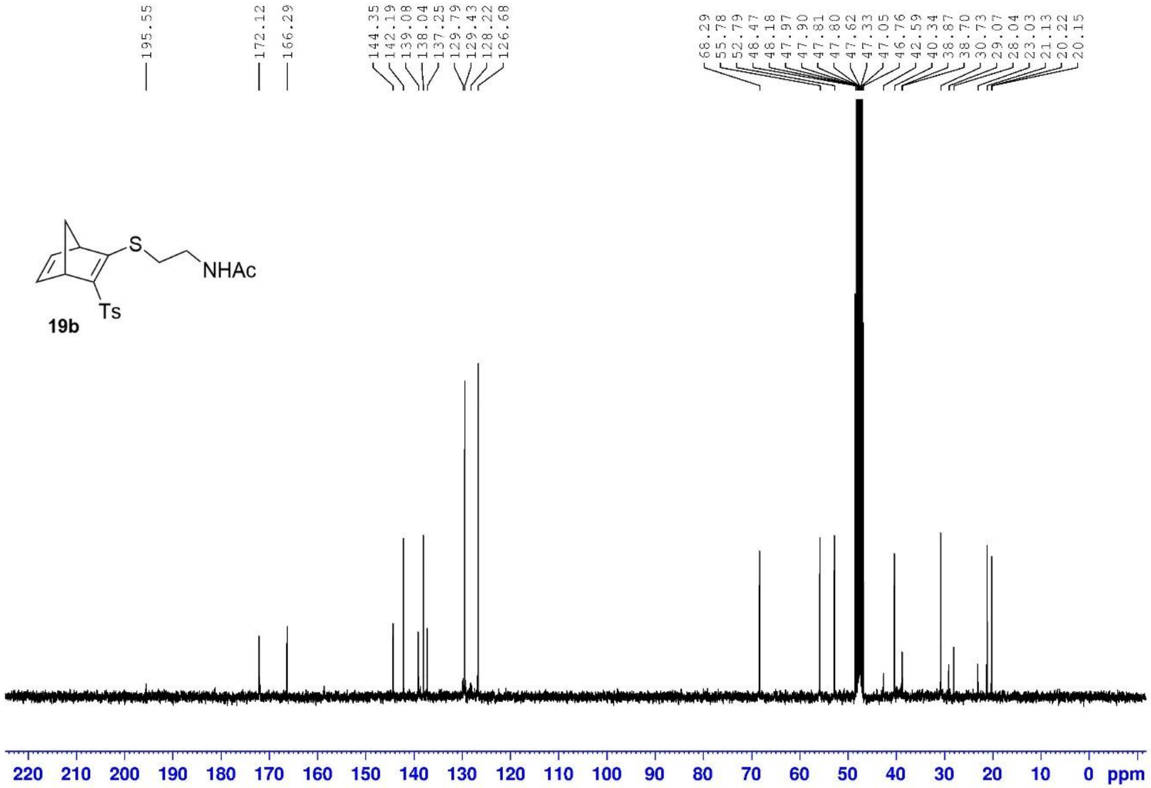

^{13}C-NMR (CD_3OD, 75 MHz) of compound **19b**

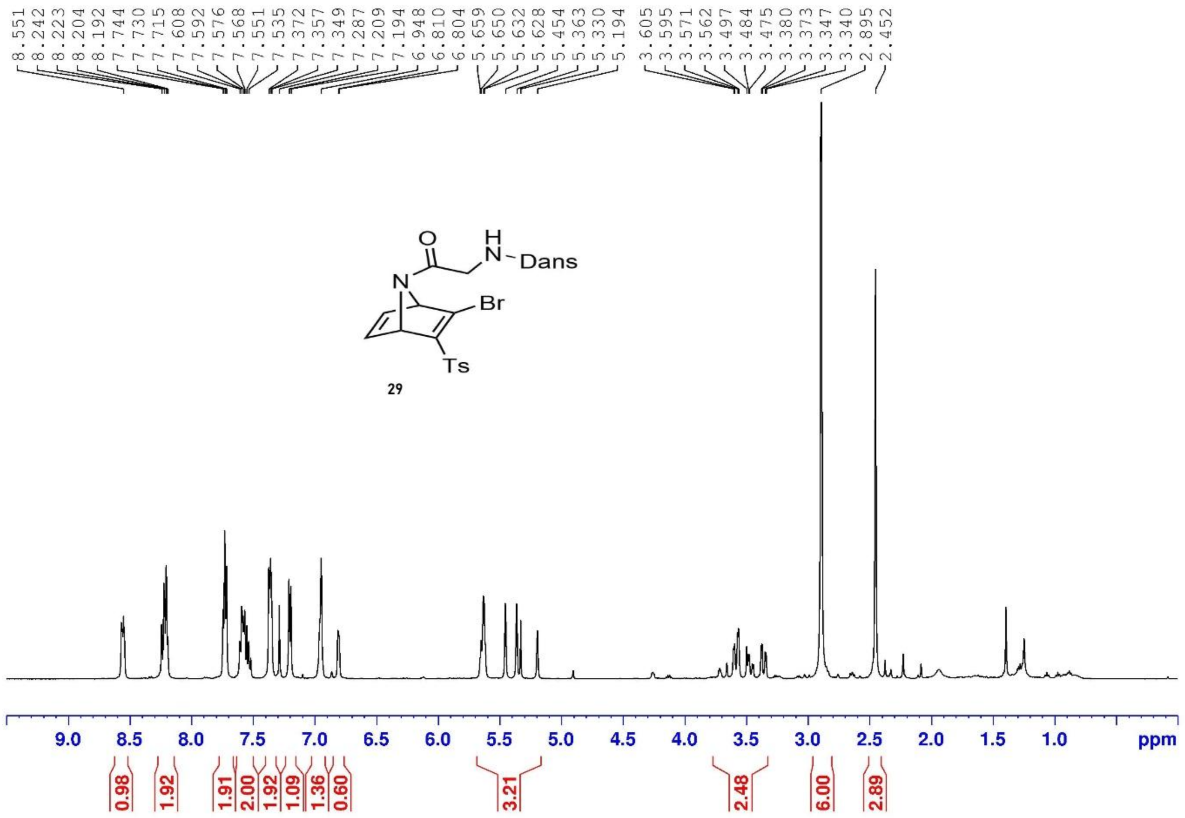

^{1}H-NMR ($CDCl_3$, 300 MHz) of compound **29**

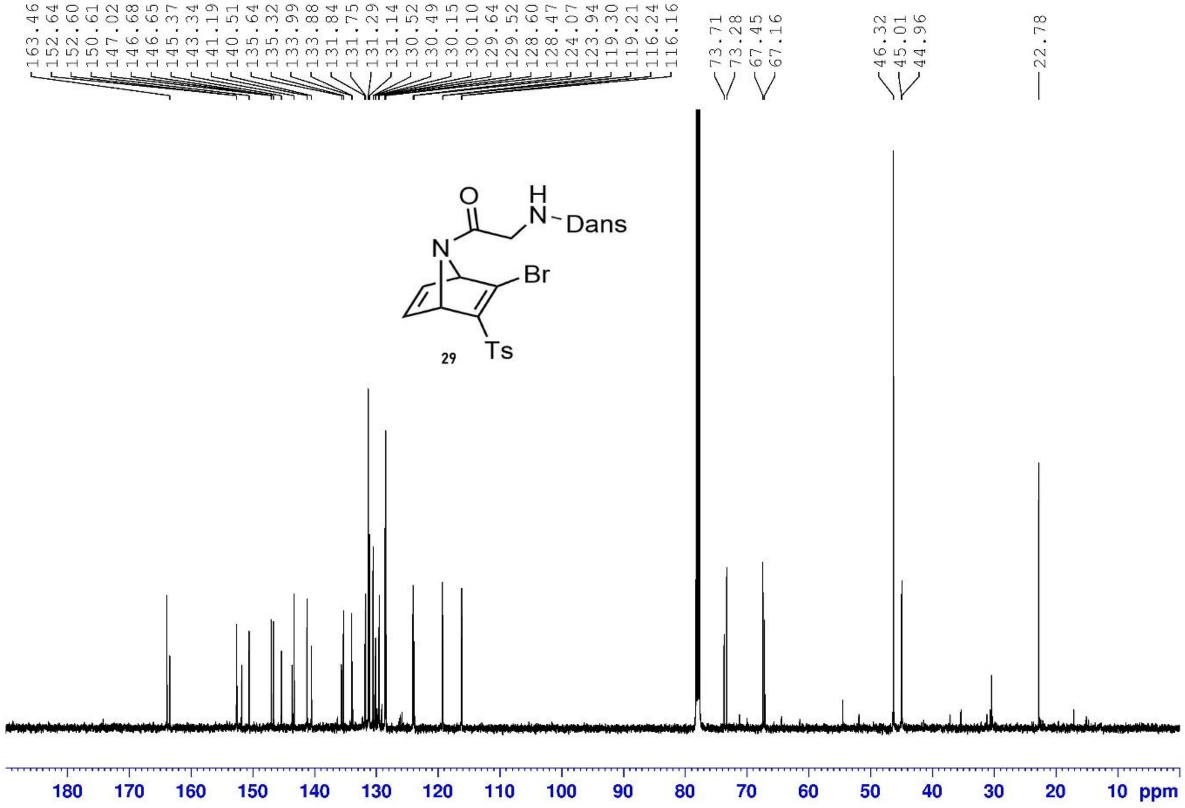

^{13}C-NMR ($CDCl_3$, 75 MHz) of compound **29**

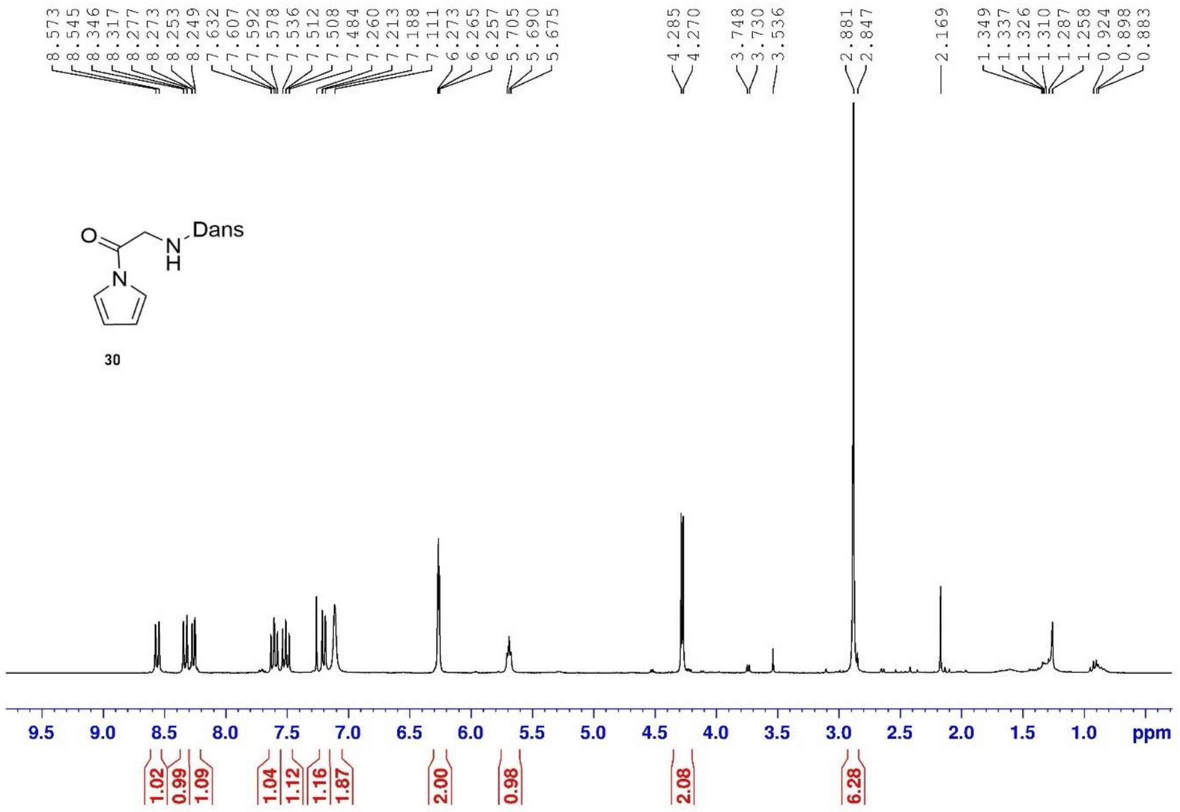

^{1}H-NMR ($CDCl_3$, 300 MHz) of compound **30**.

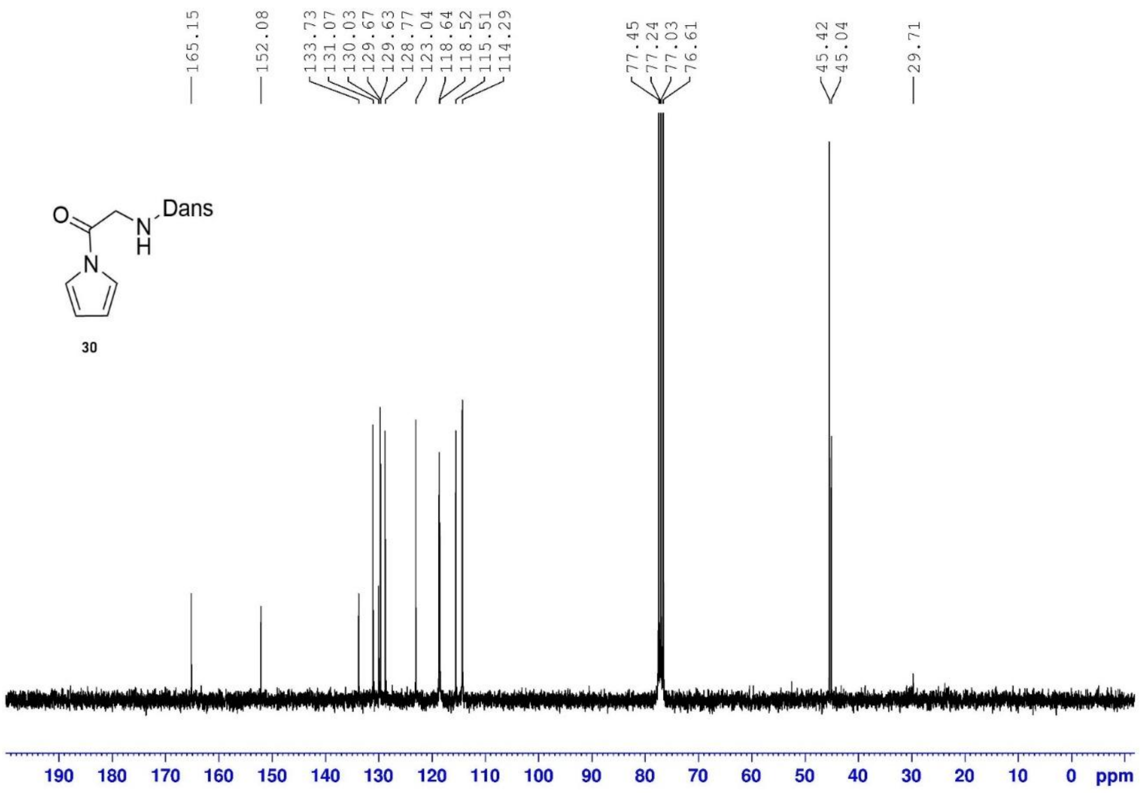

^{13}C-NMR ($CDCl_3$, 75 MHz) of compound **30.**

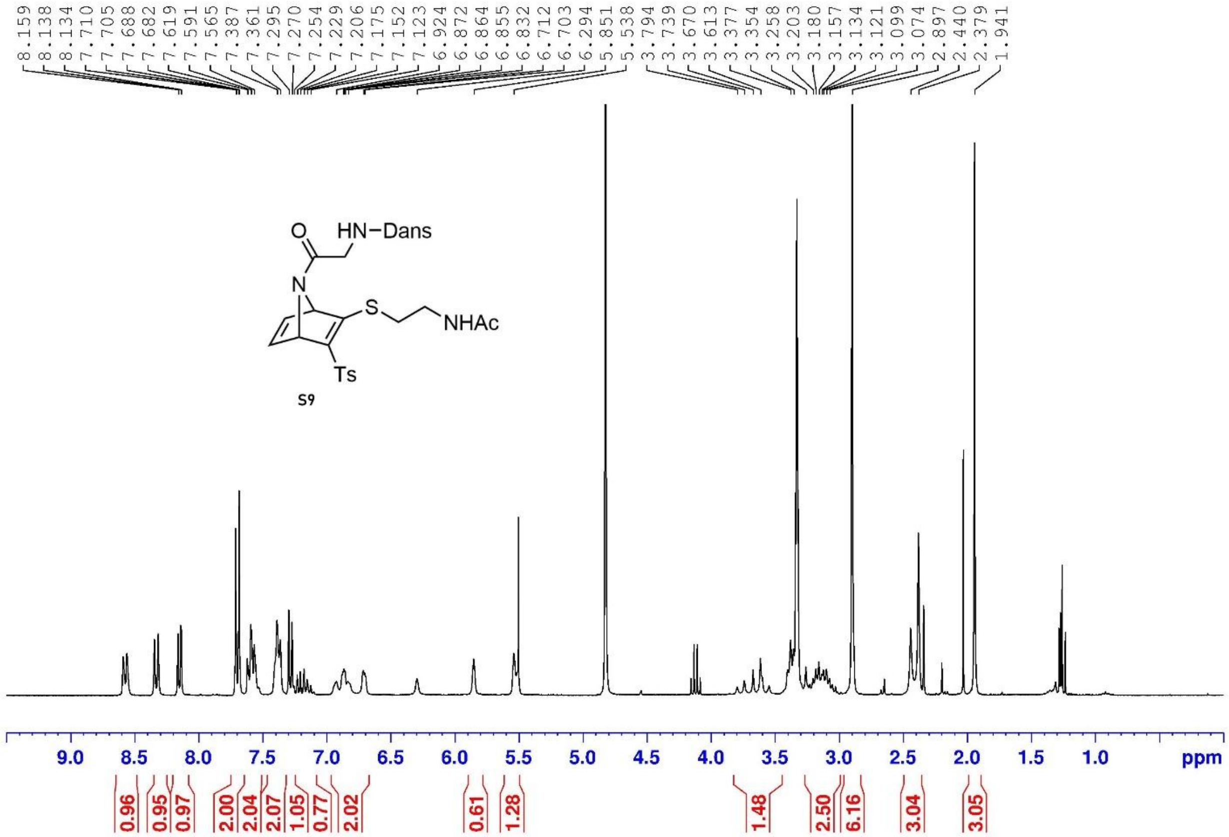

^{1}H-NMR (CD_3OD, 300 MHz) of compound **S9**.

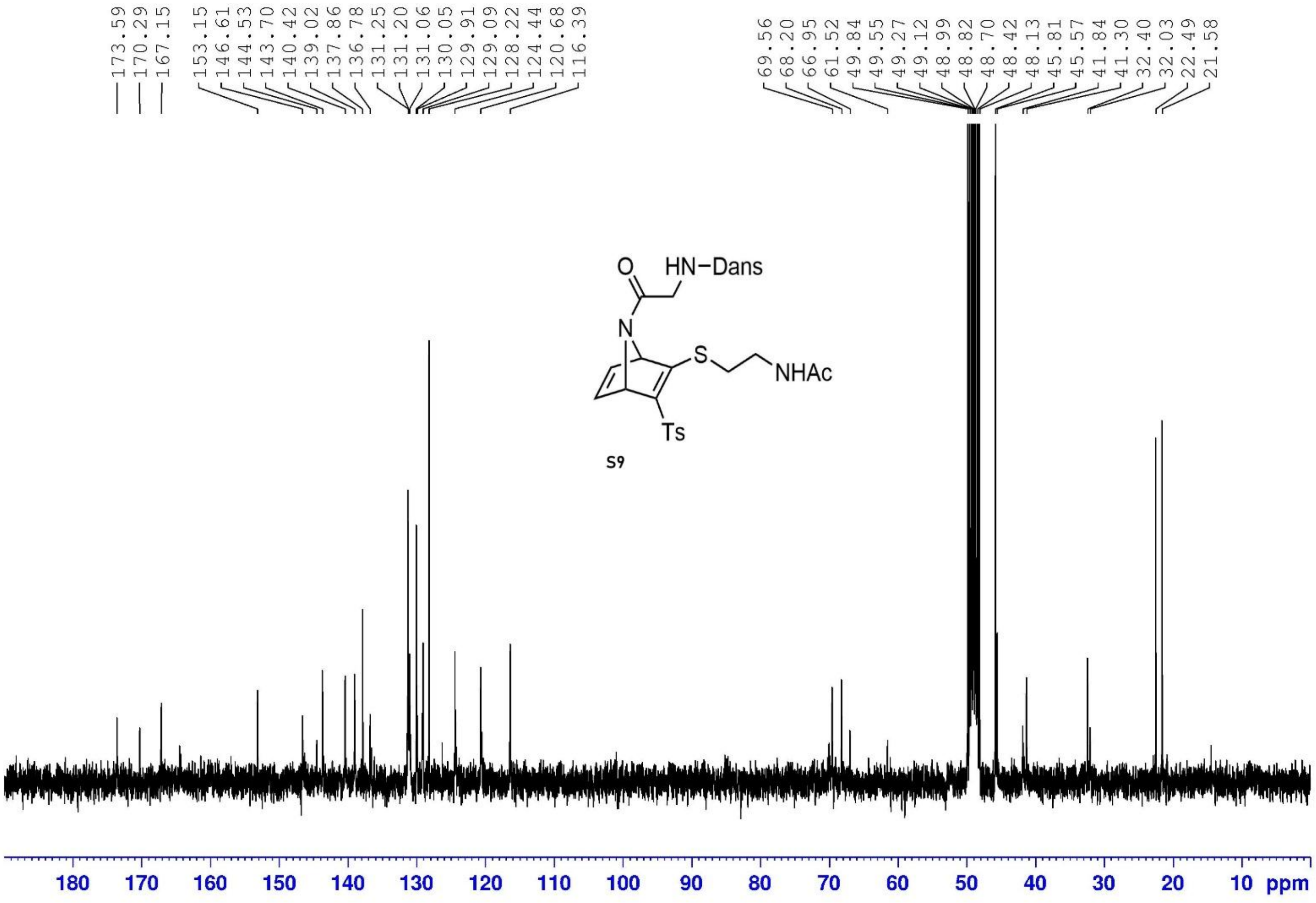

^{13}C-NMR (CD_3OD, 75 MHz) of compound **S9**.

11. References

[1] a) Bourhis, L.J.; Dolomanov, O.V.; Gildea, R.J.; Howard, J.A.K.; Puschmann, H. The anatomy of a comprehensive constrained, restrained refinement program for the modern computing environment - Olex2 dissected. *Acta Cryst.* **2015**, A71, 59-75. b) Dolomanov, O.V.; Bourhis, L.J.; Gildea, R.J; Howard, J.A.K.; Puschmann, H. OLEX2: a complete structure solution, refinement and analysis program. *J. Appl. Cryst.* **2009**, *42*, 339-341. c) Sheldrick, G.M. *Acta Cryst.* **2015**, C71, 3-8.

[2] Sheldrick, G. M. Crystal structure refinement with SHELXL. *Acta Cryst*. **2008,** *A64*, 112.

[3] Gil de Montes, E.; Jiménez-Moreno, E.; Oliveira, B. L.; Navo, C. D.; Cal, P. M. S. D.; Jiménez-Osés, G.; Robina, I.; Moreno-Vargas, A. J.; Bernardes, G. J. L. Azabicyclic vinyl sulfones for residue-specific dual protein labelling. *Chem. Sci.,* **2019**, *10*, 4515.

[4] Chen, Z.; Trudell, M. L. A Simplified Method for the Preparation of Ethynyl *p*-Tolyl Sulfone and Ethynyl Phenyl Sulfone. *Synth. Commun*., **1994**, *24*, 3149.

[5] Leroy, J. A Convenient Procedure for the Preparation of 3-Bromopropiolic Esters. *Synth. Commun.* **1992**, *22*, 567.

[6] Gil de Montes, E.; Tallarida, Matteo A.; Carmona, Ana T.; Navo, C.D.; Robina, I., Elias-Rodriguez, P., Jiménez-Osés, G.; Moreno-Vargas, A. J. Studies on the regioselective rearrangement of azanorbornanic aminyl radicals into 2,8-diazabicyclo[3.2.1]oct-2-ene systems. *J. Org. Chem.,* **2022**, *87*, 16483.

[7] Davies, H. M. L.; Matasi, J. J.; Ahmed, G. Divergent Pathways in the intramolecular reactions between Rhodium-stabilized vinylcarbenoids and pirroles: construction of fused tropanes and 7-azabicyclo[4.2.0]octadienes. *J. Org. Chem*. **1996**, *61*, 2305.

[8] a) For compound **4a**: Gil de Montes, E; Istrate, A.; Navo, C.D.; Jiménez-Moreno, E., Hoyt, E.A.; Corzana, F., Robina, I.; Jiménez-Oses, G.; Moreno-Vargas, A.J.; Bernardes, G.J.L. Stable Pyrrole-Linked Bioconjugates through Tetrazine-Triggered Azanorbornadiene Fragmentation. *Angew. Chem. Int.* **2020**, *59*, 6196-6200. b) for compound **20**: Moreno-Clavijo, E.; Kieffer, R.; Sigstam, T.; Carmona, A.T.; Moreno-Vargas, A.J.; Robina, I. *Org. Lett.,* **2011**, *23*, 6244-6247. c) for compound **21** and **22:** Moreno-Clavijo, E.; Carmona, A.T.; Moreno-Vargas, A.J.; Robina, I. Strain-promoted retro-Dieckmann-type condensation on [2.2.2]- and [2.2.1]bicyclic systems: a fragmentation reaction for the preparation of functionalized heterocycled and carbocycles. *Org. Biomol. Chem.* **2013**, *11*, 7016-7025.

[9] Murray, A. T.; Packard, E.; Nortcliffe, A.; Lewis, W.; Hamza, D.; Jones, G.; Moody, C.J. Synthesis of Epibatidine Analogues by Pyrrole Diels-Alder Reactions: Rapid Access to Azabicyclo[2.2.1] heptane and 3,8-diazabicyclo[3.2.1]octane Scaffolds for library synthesis. *Eur. J. Org. Chem.* **2017**, *1*, 138-148.

[10] Carranza, M.; Carmona, A. T.; Navo, C.D.; Robina, I.; Fratta, S.; Newburn, C.; Jiménez-Osés, G.; Moreno-Vargas, A.J. Experimental and theorical analysis of thiol-promoted fragmentation of 2-halo-3-tosyl-oxanorbornadienes. *Org. Lett.,* **2023***,* *25*, 7481-7485.

[11] García-Domínguez, J.; Carranza, M.; Jansons, E.; Carmona, A. T.; Robina, I.; Moreno-Vargas, A. J. Transferring Substituents from Alkynes to Furans and Pyrroles through Heteronorbornadienes as Intermediates: Synthesis of β-Substituted Pyrroles/Furans. *J. Org. Chem.* **2023**, *88*, 13331-13338.
